# Supplementary material for: Exploring and applying the substrate promiscuity of a C-glycosyltransferase in the chemo-enzymatic synthesis of bioactive C-glycosides
Source: Nat Commun. 2020 Oct 14;11:5162. doi: 10.1038/s41467-020-18990-9 (PMC7558026; doi:10.1038/s41467-020-18990-9)
Supplement: Supplementary file 1 — Supplementary Information [file 41467_2020_18990_MOESM1_ESM.pdf]

Supplementary Information

Exploring and applying the substrate promiscuity of a *C*-glycosyltransferase in the chemo-enzymatic synthesis of bioactive *C*-glycosides

Kebo Xie et al.

Chemical synthesis of substrates

Synthesis of 1-(3,4-dimethoxybenzyl)benzene-2,4,6-triol (20) (novel)

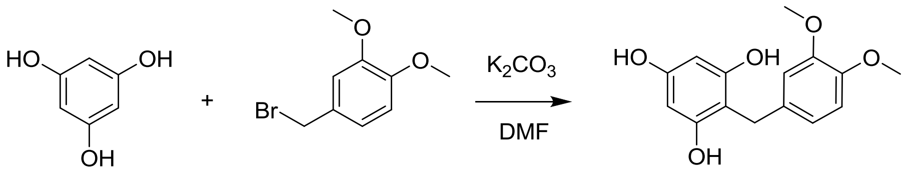

A solution of phloroglucinol (250 mg, 1.98 mmol) and K_2CO_3 (138 mg, 1.0 mmol in DMF (1.2 ml) was stirred vigorously at 0 ℃ for 10 min. 4-(Bromomethyl)-1,2-dimethoxybenzene (230 mg, 1.0 mmol) in DMF (1.2 ml) was added dropwisely. The reaction mixture was stirred at 0 ℃ for 18 h. The mixture was poured in to water (6 ml), and extracted with EtOAc (3 ml×6). The combined organic phases were washed with brine (3 ml) and water (3 ml) successively. Then, the organic layer was dried (Na_2SO_4) and concentrated. The residue was purified by semi-HPLC to obtain 1-(3,4-dimethoxybenzyl)benzene-2,4,6-triol as a brown solid (128 mg, 46.4%).

1-(3,4-dimethoxybenzyl)benzene-2,4,6-triol ^{1}H NMR (DMSO-d_6, 600 MHz): δ = 8.93 (2H, s, -OH), 8.79 (1H, s, -OH), 6.85 (1H, d, J = 1.9 Hz, H-2′), 6.75 (1H, d, J = 8.1 Hz, H-5′), 6.66 (1H, dd, J = 8.1, 1.9 Hz, H-6′), 5.79 (2H, s, H-3, H-5), 3.67 (6H, s, overlapped, -CH_3, -CH_3), 3.62 (2H, s, -CH_2-); ^{13}C NMR (DMSO-d_6, 150 MHz): δ = 156.9 (C-2), 156.9 (C-6), 156.7 (C-4), 148.6 (C-3′), 146.9 (C-4′), 135.8 (C-1′), 120.5, 113.1, 112.1, 105.8, 94.5 (C-3), 94.5 (C-5), 56.0 (-CH_3), 55.8 (-CH_3), 28.0 (-CH_2-). HRESIMS (m/z): $[M+H]^+$ calcd. for $C_{15}H_{17}O_5$, 277.1076; found, 277.1081.

Synthesis of 1-(4′-methoxyphenethyl)benzene-2,4,6-triol (21) (novel)

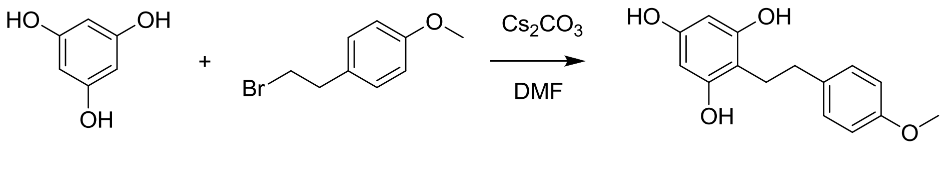

A solution of phloroglucinol (250 mg, 1.98 mmol) and Cs_2CO_3 (315 mg, 1.0 mmol) in DMF (1.2 ml) was stirred vigorously at 0 ℃ for 10 min. 4-Methoxyphenethyl bromide (215 mg, 1.0 mmol) in DMF (1.2 ml) was added dropwisely. The reaction mixture was stirred at 0 ℃ for 18 h. The mixture was poured in to water (6 ml), and extracted with EtOAc (6 ml×2). The combined organic phases were washed with

brine (3 ml) and water (3 ml) successively. Then, the organic layer was dried (Na_2SO_4) and concentrated. The residue was purified by semi-HPLC to obtain 1-(4′-methoxyphenethyl)benzene-2,4,6-triol as a light yellow powder (15.0 mg, 5.8%).

1-(4′-methoxyphenethyl)benzene-2,4,6-triol ^{1}H NMR (DMSO-d_6, 600 MHz): δ = 8.83 (3H, s, 2-OH, 4-OH, 6-OH), 7.08 (2H, d, J = 8.5 Hz, H-2′, H-6′), 6.80 (2H, d, J = 8.5 Hz, H-3′, H-5′), 5.75 (2H, s, H-3, H-5), 3.68 (3H, s, -CH_3), 2.55 (4H, m, -CH_2-, -CH_2-); ^{13}C NMR (DMSO-d_6, 150 MHz): δ = 157.6 (C-4′), 156.9 (C-2), 156.9 (C-6), 156.3 (C-4), 135.3 (C-1′), 129.4 (C-2′), 129.4 (C-6′), 114.0 (C-3′), 114.0 (C-5′), 106.0 (C-1), 94.5 (C-3), 94.5 (C-5), 55.4 (-CH_3), 34.8 (-CH_2-), 25.5 (-CH_2-). HRESIMS (m/z): $[M+H]^+$ calcd. for $C_{15}H_{17}O_4$, 261.1127; found, 261.1122.

Synthesis of 1-(2-ethylbutyl)benzene-2,4,6-triol (31) (novel)

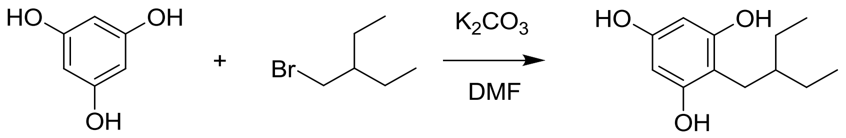

A solution of phloroglucinol (250 mg, 1.98 mmol) and K_2CO_3 (138 mg, 1.0 mmol) in DMF (1.2 ml) was stirred vigorously at 0 ℃ for 10 min. 1-Bromo-2-ethylbutane (164 mg, 1.0 mmol) in DMF (1.2 ml) was added dropwisely. The reaction mixture was stirred at 0 ℃ for 18 h. The mixture was poured in to water (6 ml), and extracted with EtOAc (6 ml×2). The combined organic phases were washed with brine (3 ml) and water (3 ml) successively. Then, the organic layer was dried (Na_2SO_4) and concentrated. The residue was purified by semi-HPLC to obtain 1-(2-ethylbutyl)benzene-2,4,6-triol as a yellow powder (7.5 mg, 3.6%).

1-(2-ethylbutyl)benzene-2,4,6-triol ^{1}H NMR (DMSO-d_6, 600 MHz): δ = 8.65 (3H, s, 2-OH, 4-OH, 6-OH), 5.71 (2H, s, H-3, H-5), 2.25 (2H, d, J = 7.2 Hz, -CH_2-), 1.44 (1H, m, -CH-), 1.18 (4H, m, -CH_2-, -CH_2-), 0.78 (6H, t, J = 7.4 Hz, -CH_3, -CH_3); ^{13}C NMR (DMSO-d_6, 150 MHz): δ = 157.2 (C-2), 157.2 (C-6), 156.0 (C-4), 105.7 (C-1), 94.4 (C-3), 94.4 (C-5), 40.42 (-CH-), 27.0 (-CH_2-), 25.2 (-CH_2-), 25.2 (-CH_2-), 11.3 (-CH_3), 11.3 (-CH_3). HRESIMS (m/z): $[M+H]^+$ calcd. for $C_{12}H_{19}O_3$, 211.1334; found, 211.1332.

Synthesis of 1-(5-methylhexyl)benzene-2,4,6-triol (32) (novel)

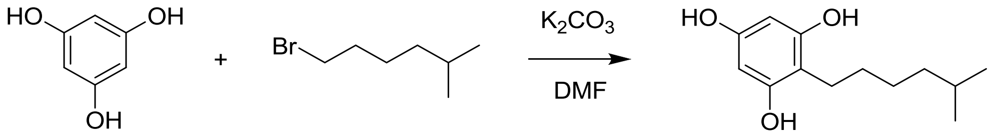

A solution of phloroglucinol (250 mg, 1.98 mmol) and K_2CO_3 (138 mg, 1.0 mmol) in DMF (1.2 ml) was stirred vigorously at 0 ℃ for 10 min. 1-Bromo-5-methylhexane (178 mg, 1.0 mmol) in DMF (1.2 ml) was added dropwisely. The reaction mixture was stirred at 0 ℃ for 18 h. The mixture was poured in to water (6 ml), and extracted with EtOAc (6 ml×2). The combined organic phases were washed with brine (3 ml) and water (3 ml) successively. Then, the organic layer was dried (Na_2SO_4) and concentrated. The residue was purified by semi-HPLC to obtain 1-(5-methylhexyl)benzene-2,4,6-triol as a brown solid (7.1 mg, 3.5%).

1-(5-methylhexyl)benzene-2,4,6-triol ^{1}H NMR (DMSO-d_6, 600 MHz): δ = 8.64–8.68 (3H, overlapped, 2-OH, 4-OH, 6-OH), 5.71 (2H, s, H-3, H-5), 2.31 (2H, dd, J = 7.4 Hz, -CH_2-), 1.46 (2H, m, -CH_2-), 1.30 (2H, m, -CH_2-), 1.21 (2H, m, -CH_2-), 1.12 (2H, m, -CH_2-), 0.81 (3H, s, -CH_3), 0.80 (3H, s, -CH_3); ^{13}C NMR (DMSO-d_6, 150 MHz): δ = 156.8 (C-2), 156.8 (C-6), 156.0 (C-4), 106.6 (C-1), 94.4 (C-3), 94.4 (C-5), 49.0 (-CH-), 39.0 (-CH_2-), 30.0 (-CH_2-), 27.9 (-CH_2-), 27.5 (-CH_2-), 23.0 (-CH_3), 23.0 (-CH_3). HRESIMS (m/z): $[M+H]^+$ calcd. for $C_{13}H_{21}O_3$, 225.1491; found, 225.1489.

Synthesis of 1-dodecyl-2,4,6-benzenetriol (33) (novel)

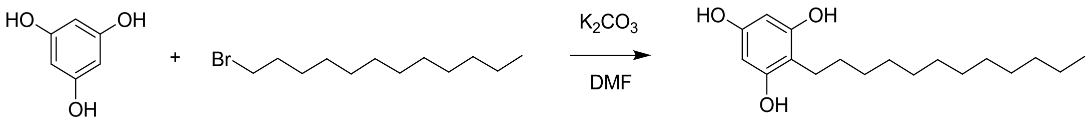

A solution of phloroglucinol (250 mg, 1.98 mmol) and K_2CO_3 (138 mg, 1.0 mmol) in DMF (1.2 ml) was stirred vigorously at 0 ℃ for 10 min. 1-Bromododecane (248 mg, 1.0 mmol) in DMF (1.2 ml) was added dropwisely. The reaction mixture was stirred at 0 ℃ for 18 h. The mixture was poured in to water (6 ml), and extracted with EtOAc (3 ml×2). The combined organic phases were washed with brine (3 ml) and water (3 ml) successively. Then, the organic layer was dried (Na_2SO_4) and concentrated. The residue was purified by semi-HPLC to obtain 1-dodecyl-2,4,6-benzenetriol as a brown

solid (5.1 mg, 1.7%).

1-dodecyl-2,4,6-benzenetriol ^{1}H NMR (DMSO-d_6, 600 MHz): δ = 8.70 (2H, s, -OH), 8.67 (1H, s, -OH), 5.74 (2H, s, H-3, H-5), 2.33 (2H, t, J = 7.2 Hz, -CH_2-), 1.33 (2H, m, -CH_2-), 1.23–1.28 (18H, m, overlapped, -CH_2-CH_2-CH_2-CH_2-CH_2-CH_2-CH_2-CH_2-CH_2-), 0.85 (3H, t, J = 6.8 Hz, -CH_3); ^{13}C NMR (DMSO-d_6, 150 MHz): δ = 156.8 (C-2), 156.8 (C-6), 156.0 (C-4), 106.6 (C-1), 94.4 (C-3), 94.4 (C-5), 37.1 (-CH_2-), 29.7 (-CH_2-), 29.6 (-CH_2-), 29.5 (-CH_2-), 29.5 (-CH_2-), 29.5 (-CH_2-), 29.5 (-CH_2-), 29.2 (-CH_2-), 22.8 (-CH_2-), 22.5 (-CH_2-), 22.5 (-CH_2-), 14.4 (-CH_3). HRESIMS (m/z): $[M+H]^+$ calcd. for $C_{18}H_{31}O_3$, 295.2273; found, 295.2269.

Synthesis of 1-(cyclopropylmethyl)benzene-2,4,6-triol (34) (novel)

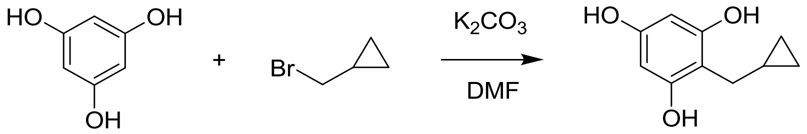

A solution of phloroglucinol (250 mg, 1.98 mmol) and K_2CO_3 (138 mg, 1.0 mmol) in DMF (1.2 ml) was stirred vigorously at 0 ℃ for 10 min. Bromomethylcyclopropane (134 mg, 1.0 mmol) in DMF (1.2 ml) was added dropwisely. The reaction mixture was stirred at 0 ℃ for 18 h. The mixture was poured in to water (6 ml), and extracted with EtOAc (6 ml×2). The combined organic phases were washed with brine (3 ml) and water (3 ml) successively. Then, the organic layer was dried (Na_2SO_4) and concentrated. The residue was purified by semi-HPLC to obtain 1-(cyclopropylmethyl)benzene-2,4,6-triol as a light yellow powder (15.6 mg, 8.7%).

1-(cyclopentylmethyl)benzene-2,4,6-triol ^{1}H NMR (DMSO-d_6, 600 MHz): δ = 8.72 (2H, s, 2-OH, 6-OH), 8.68 (1H, s, 4-OH), 5.73 (2H, s, H-3, H-5), 2.28 (2H, d, J = 6.5 Hz, -CH_2-), 0.87 (1H, m, -CH-), 0.20 (2H, m, -CH_2-), 0.07 (2H, m, -CH_2-); ^{13}C NMR (DMSO-d_6, 150 MHz): δ = 156.9 (C-2), 156.9 (C-6), 156.1 (C-4), 105.8 (C-1), 94.4 (C-3), 94.4 (C-5), 26.9 (-CH_2-), 11.6 (-CH-), 4.7 (-CH_2-), 4.7 (-CH_2-). HRESIMS (m/z): $[M+H]^+$ calcd. for $C_{10}H_{13}O_3$, 181.0865; found, 181.0863.

Synthesis of 1-(cyclobutylmethyl)benzene-2,4,6-triol (35) (novel)

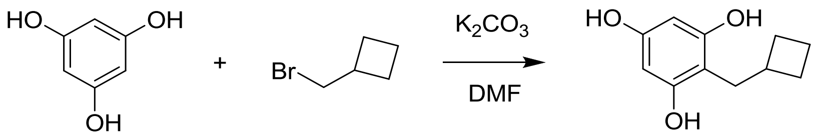

A solution of phloroglucinol (250 mg, 1.98 mmol) and K_2CO_3 (138 mg, 1.0 mmol) in DMF (1.2 ml) was stirred vigorously at 0 ℃ for 10 min. Bromomethylcyclobutane (148 mg, 1.0 mmol) in DMF (1.2 ml) was added dropwisely. The reaction mixture was stirred at 0 ℃ for 18 h. The mixture was poured in to water (6 ml), and extracted with EtOAc (6 ml×2). The combined organic phases were washed with brine (3 ml) and water (3 ml) successively. Then, the organic layer was dried (Na_2SO_4) and concentrated. The residue was purified by semi-HPLC to obtain 1-(cyclobutylmethyl)benzene-2,4,6-triol as a light yellow solid (5.6 mg, 2.9%).

1-(cyclobutylmethyl)benzene-2,4,6-triol ^{1}H NMR (DMSO-d_6, 600 MHz): δ = 8.70 (3H, s, 2-OH, 4-OH, 6-OH), 5.71 (2H, s, H-3, H-5), 2.44 (2H, d, J = 2.2 Hz, -CH_2-), 1.97 (1H, m, -CH-), 1.80 (2H, m, -CH_2-), 1.62–1.71 (4H, m, -CH_2-); ^{13}C NMR (DMSO-d_6, 150 MHz): δ = 157.1 (C-2), 157.1 (C-6), 156.0 (C-4), 105.0 (C-1), 94.4 (C-3), 94.4 (C-5), 36.0 (-CH-), 29.4 (-CH_2-), 27.9 (-CH_2-), 27.9 (-CH_2-), 18.2 (-CH_2-). HRESIMS (m/z): $[M+H]^+$ calcd. for $C_{11}H_{15}O_3$, 195.1021; found, 195.1021.

Synthesis of 1-(cyclopentylmethyl)benzene-2,4,6-triol (36) (novel)

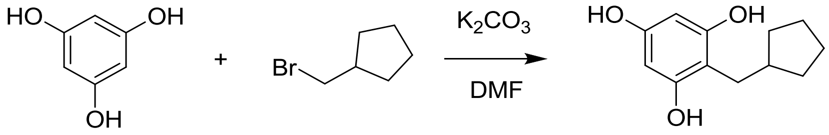

A solution of phloroglucinol (250 mg, 1.98 mmol) and K_2CO_3 (138 mg, 1.0 mmol) in DMF (1.2 ml) was stirred vigorously at 0 ℃ for 10 min. Bromomethylcyclopentane (162 mg, 1.0 mmol) in DMF (1.2 ml) was added dropwisely. The reaction mixture was stirred at 0 ℃ for 18 h. The mixture was poured in to water (6 ml), and extracted with EtOAc (6 ml×2). The combined organic phases were washed with brine (3 ml) and water (3 ml) successively. Then, the organic layer was dried (Na_2SO_4) and concentrated. The residue was purified by semi-HPLC to obtain 1-(cyclopentylmethyl)benzene-2,4,6-triol as a brown solid (10.4 mg, 5.0%).

1-(cyclopentylmethyl)benzene-2,4,6-triol ^{1}H NMR (DMSO-d_6, 600 MHz): δ = 8.67 (3H, s, 2-OH, 4-OH, 6-OH), 5.71 (2H, s, H-3, H-5), 2.31 (2H, d, J = 7.4 Hz, -CH_2-),

2.04 (1H, m, -CH-), 1.55 (2H, m, -CH_2-), 1.46 (2H, m, -CH_2-), 1.39 (2H, m, -CH_2-), 1.19 (2H, m, -CH_2-); ^{13}C NMR (DMSO-d_6, 150 MHz): δ = 157.0 (C-2), 157.0 (C-6), 156.0 (C-4), 106.2 (C-1), 94.4 (C-3), 94.4 (C-5), 40.1 (overlapped, -CH-), 32.2 (-CH_2-), 32.2 (-CH_2-), 28.4 (-CH_2-), 25.0 (-CH_2-), 25.0 (-CH_2-). HRESIMS (m/z): $[M+H]^+$ calcd. for $C_{12}H_{17}O_3$, 209.1178; found, 209.1179.

Synthesis of 1-(cyclohexylmethyl)benzene-2,4,6-triol (37) (novel)

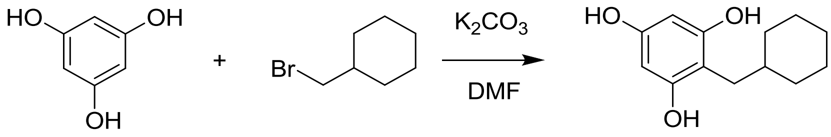

A solution of phloroglucinol (250 mg, 1.98 mmol) and K_2CO_3 (138 mg, 1.0 mmol) in DMF (1.2 ml) was stirred vigorously at 0 ℃ for 10 min. Bromomethylcyclohexane (264 mg, 1.5 mmol) in DMF (1.2 ml) was added dropwisely. The reaction mixture was stirred at 0 ℃ for 18 h. The mixture was poured in to water (6 ml), and extracted with EtOAc (6 ml×2). The combined organic phases were washed with brine (3 ml) and water (3 ml) successively. Then, the organic layer was dried (Na_2SO_4) and concentrated. The residue was purified by semi-HPLC to obtain 1-(cyclohexylmethyl)benzene-2,4,6-triol as a light yellow solid (8.9 mg, 2.7%).

1-(cyclohexylmethyl)benzene-2,4,6-triol HRESIMS: *m/z* 223.1321 $[M+H]^+$; ESI-MS *m/z* 223.21 $[M+H]^+$; ^{1}H NMR (DMSO-d_6, 600 MHz): δ = 8.69 (3H, overlapped, -OH), 5.75 (2H, s, H-2, H-3), 2.25 (2H, d, J = 7.1 Hz, -CH_2-), 1.62 (2H, m, -CH_2-), 1.56 (2H, m, -CH_2-), 1.44 (1H, m, H-1′), 1.10 (4H, m, -CH_2-, -CH_2-), 0.93 (2H, m, -CH_2-); ^{13}C NMR (DMSO-d_6, 150 MHz): δ = 157.2 (C-2), 157.2 (C-6), 156.0 (C-4), 105.2 (C-1), 94.4 (C-3), 94.4 (C-5), 38.0 (C-1′), 33.4 (C-2′), 33.4 (C-6′), 30.3 (-CH_2-), 26.8 (C-4′), 26.4 (C-3′), 26.4 (C-5′). HRESIMS (m/z): $[M+H]^+$ calcd. for $C_{13}H_{19}O_3$, 223.1334; found, 223.1331.

Synthesis of 1-benzylbenzene-2,4,6-triol (38)

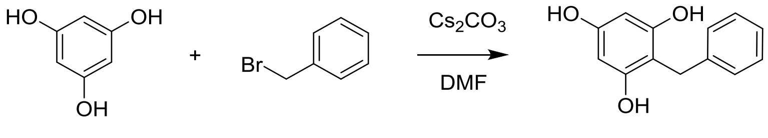

A solution of phloroglucinol (250 mg, 1.98 mmol) and Cs_2CO_3 (315 mg, 1.0 mmol) in

DMF (1.2 ml) was stirred vigorously at 0 ℃ for 10 min. Benzyl bromide (171 mg, 1.0 mmol) in DMF (1.2 ml) was added dropwisely. The reaction mixture was stirred at 0 ℃ for 20 h. The mixture was poured in to water (6 ml), and extracted with EtOAc (6 ml×2). The combined organic phases were washed with brine (3 ml) and water (3 ml) successively. Then, the organic layer was dried (Na_2SO_4) and concentrated. The residue was purified by semi-HPLC to obtain 1-benzylbenzene-2,4,6-triol as a brown solid (138 mg, 63.9%).

1-benzylbenzene-2,4,6-triol ^{1}H NMR (DMSO-d_6, 600 MHz): δ = 8.91 (2H, s, -OH, -OH), 8.78 (1H, s, -OH), 7.14–7.15 (4H, m, H-2′, H-3′, H-5′, H-6′), 7.03 (1H, m, H-4′), 5.77 (2H, s, H-3, H-5), 3.67 (2H, s, -CH_2-); ^{13}C NMR (DMSO-d_6, 150 MHz): δ = 156.9 (C-2), 156.9 (C-6), 156.7 (C-3), 143.2 (C-1′), 128.7 (C-3′), 128.7 (C-5′), 128.1 (C-2′), 128.1 (C-6′), 125.4 (C-4′), 105.4 (C-1), 94.5 (C-3), 94.5 (C-5), 28.5 (-CH_2-). ESI-MS *m/z* 217.15 $[M+H]^+$.

Synthesis of 1-phenethylbenzene-2,4,6-triol (39)

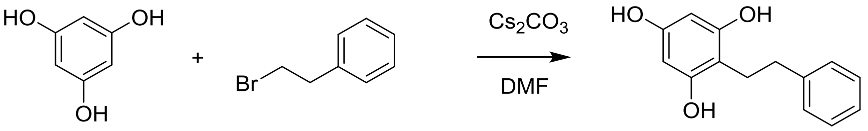

A solution of phloroglucinol (250 mg, 1.98 mmol) and Cs_2CO_3 (315 mg, 1.0 mmol) in DMF (1.2 ml) was stirred vigorously at 0 ℃ for 10 min. (2-Bromoethyl)benzene (185 mg, 1.0 mmol) in DMF (1.2 ml) was added dropwisely. The reaction mixture was stirred at 0 ℃ for 18 h. The mixture was poured in to water (6 ml), and extracted with EtOAc (6 ml×2). The combined organic phases were washed with brine (3 ml) and water (3 ml) successively. Then, the organic layer was dried (Na_2SO_4) and concentrated. The residue was purified by semi-HPLC to obtain 1-phenethylbenzene-2,4,6-triol as a light yellow solid (6.1 mg, 2.7%).

1-phenethylbenzene-2,4,6-triol ^{1}H NMR (DMSO-d_6, 600 MHz): δ = 8.81 (2H, s, -OH, -OH), 8.76 (1H, s, -OH), 7.22 (2H, d, J = 7.3 Hz, H-2′, H-6′), 7.14 (2H, t, J = 7.3 Hz, H-3′, H-5′), 7.02 (2H, t, J = 7.3 Hz, H-4′), 1.54 (2H, s, -CH_2-), 1.53 (2H, s, -CH_2-); ^{13}C NMR (DMSO-d_6, 150 MHz): δ = 157.0 (C-2), 157.0 (C-6), 156.5 (C-3), 147.5 (C-1′), 127.8 (C-3′), 127.8 (C-5′), 127.6 (C-2′), 127.6 (C-6′), 125.0 (C-4′), 110.1 (C-1), 94.9 (C-3), 94.9 (C-5), 33.2 (-CH_2-), 18.6 (-CH_2-). ESI-MS *m/z* 231.03

$[M+H]^+$.

Synthesis of 1-(4-phenylbutyl)benzene-2,4,6-triol (40) (novel)

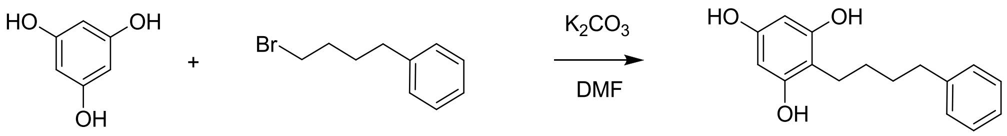

A solution of phloroglucinol (250 mg, 1.98 mmol) and K_2CO_3 (138 mg, 1.0 mmol) in DMF (1.2 ml) was stirred vigorously at 0 ℃ for 10 min. 1-Bromo-4-phenylbutane (212 mg, 1.0 mmol) in DMF (1.2 ml) was added dropwisely. The reaction mixture was stirred at 0 ℃ for 18 h. The mixture was poured in to water (6 ml), and extracted with EtOAc (3 ml×2). The combined organic phases were washed with brine (3 ml) and water (3 ml) successively. Then, the organic layer was dried (Na_2SO_4) and concentrated. The residue was purified by semi-HPLC to obtain 1-(4-phenylbutyl)benzene-2,4,6-triol as a brown solid (5.9 mg, 2.3%).

1-(4-phenylbutyl)benzene-2,4,6-triol ^{1}H NMR (DMSO-d_6, 600 MHz): δ = 8.74 (2H, s, -OH), 8.68 (1H, s, -OH), 7.24 (2H, t, J = 7.6 Hz, H-3′, H-5′), 7.16 (2H, d, J = 7.6 Hz, H-2′, H-6′), 7.14 (1H, t, J = 7.6 Hz, H-4′), 5.74 (2H, s, H-3, H-5), 2.56 (2H, t, J = 7.7 Hz, $-CH_2-$), 2.39 (2H, t, J = 7.5 Hz, $-CH_2-$), 1.52 (2H, m, $-CH_2-$), 1.39 (2H, m, $-CH_2-$); ^{13}C NMR (DMSO-d_6, 150 MHz): δ = 156.9 (C-2), 156.9 (C-6), 156.0 (C-4), 143.1 (C-1′), 128.7 (C-3′), 128.7 (C-5′), 128.6 (C-2′), 128.6 (C-6′), 125.9 (C-4′), 106.4 (C-4), 94.4 (C-3), 94.4 (C-5), 35.7 ($-CH_2-$), 31.8 ($-CH_2-$), 29.3 ($-CH_2-$), 22.6 ($-CH_2-$). HRESIMS (m/z): $[M+H]^+$ calcd. for $C_{16}H_{19}O_3$, 259.1334; found, 259.1330.

Synthesis of 1-(6-phenylhexyl)benzene-2,4,6-triol (41) (novel) and 1-(6-phenylhexyloxy)benzene-3,5-diol (22) (novel)

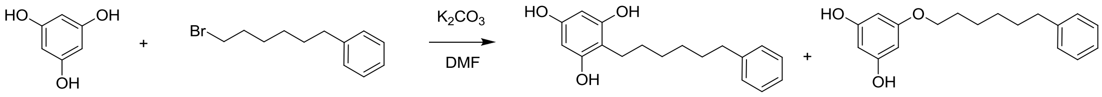

41 22

A solution of phloroglucinol (250 mg, 1.98 mmol) and K_2CO_3 (138 mg, 1.0 mmol) in DMF (1.2 ml) was stirred vigorously at 0 ℃ for 10 min. (6-bromohexyl)benzene (240 mg, 1.0 mmol) in DMF (1.2 ml) was added dropwisely. The reaction mixture was stirred at 0 ℃ for 18 h. The mixture was poured in to water (6 ml), and extracted with EtOAc (3 ml×3). The combined organic phases were washed with brine (3 ml) and

water (3 ml) successively. Then, the organic layer was dried (Na_2SO_4) and concentrated. The residue was purified by semi-HPLC to obtain 1-(6-phenylhexyl)benzene-2,4,6-triol as brown solid (5.3 mg, 1.9%) and 1-(6-phenylhexyloxy)benzene-3,5-diol as a light yellow solid (20.2 mg, 7.1%).

1-(6-phenylhexyl)benzene-2,4,6-triol ^{1}H NMR (DMSO-d_6, 600 MHz): δ = 8.71 (2H, s, -OH), 8.67 (1H, s, -OH), 7.25 (2H, t, J = 7.6 Hz, H-3′, H-5′), 7.17 (2H, d, J = 7.6 Hz, H-2′, H-6′), 7.14 (1H, m, overlapped, H-4′), 5.74 (2H, s, H-3, H-5), 2.54 (2H, t, J = 7.7 Hz, -CH_2-), 2.34 (2H, t, J = 7.3 Hz, -CH_2-), 1.54 (2H, m, -CH_2-), 1.34 (2H, m, -CH_2-), 1.27–1.29 (2H, m, -CH_2-, -CH_2-); ^{13}C NMR (DMSO-d_6, 150 MHz): δ = 156.8 (C-2), 156.8 (C-6), 156.0 (C-4), 142.8 (C-1′), 128.7 (C-3′), 128.7 (C-5′), 128.6 (C-2′), 128.6 (C-6′), 126.0 (C-4′), 106.6 (C-4), 94.5 (C-3), 94.5 (C-5), 35.6 (-CH_2-), 31.5 (-CH_2-), 29.6 (-CH_2-), 29.4 (-CH_2-), 29.1 (-CH_2-), 22.8 (-CH_2-); HRESIMS (m/z): $[M+H]^+$ calcd. for $C_{18}H_{23}O_3$, 287.1647; found, 287.1642.

1-(6-phenylhexyloxy)benzene-3,5-diol ^{1}H NMR (DMSO-d_6, 600 MHz): δ = 9.12 (1H, s, overlapped, -OH), 9.12 (1H, s, overlapped, -OH), 7.23 (2H, t, J = 7.6 Hz, H-3′, H-5′), 7.15 (2H, d, overlapped, J = 7.9 Hz, H-2′, H-6′), 7.13 (1H, m, overlapped, H-4′), 5.78 (1H, d, J = 2.0 Hz, H-4), 5.74 (2H, d, J = 2.0 Hz, H-2, H-6), 3.76 (2H, t, J = 6.5 Hz, -CH_2-), 2.53 (2H, m, -CH_2-), 1.60 (2H, m, -CH_2-), 1.54 (2H, m, -CH_2-), 1.37 (2H, m, -CH_2-), 1.29 (2H, m, -CH_2-); ^{13}C NMR (DMSO-d_6, 150 MHz): δ = 160.9 (C-1), 159.4 (C-3), 159.4 (C-5), 142.7 (C-1′), 128.7 (C-3′), 128.7 (C-5′), 128.6 (C-2′), 128.6 (C-6′), 126.0 (C-4′), 95.8 (C-4), 93.5 (C-2), 93.5 (C-6), 67.4 (-CH_2-), 35.5 (-CH_2-), 31.4 (-CH_2-), 29.1 (-CH_2-), 28.8 (-CH_2-), 25.8 (-CH_2-). HRESIMS (m/z): $[M+H]^+$ calcd. for $C_{18}H_{23}O_3$, 287.1647; found, 287.1642.

Synthesis of 1-(thiophen-1′-ylmethyl)benzene-2,4,6-triol (42) (novel)

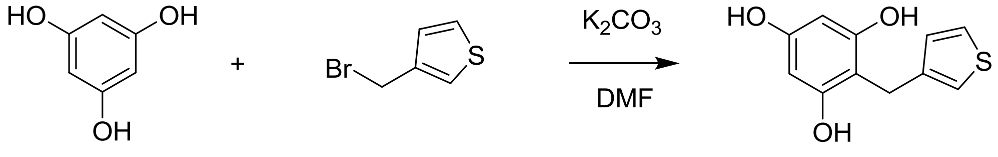

A solution of phloroglucinol (250 mg, 1.98 mmol) and K_2CO_3 (138 mg, 1.0 mmol) in DMF (1.2 ml) was stirred vigorously at 0 ℃ for 10 min. 3-(Bromomethyl)thiophene (178 mg, 1.0 mmol) in DMF (1.2 ml) was added dropwisely. The reaction mixture was stirred at 0 ℃ for 18 h. The mixture was poured in to water (6 ml), and extracted

with EtOAc (6 ml×2). The combined organic phases were washed with brine (3 ml) and water (3 ml) successively. Then, the organic layer was dried (Na_2SO_4) and concentrated. The residue was purified by semi-HPLC to obtain 1-(thiophen-1′-ylmethyl)benzene-2,4,6-triol as a dark brown solid (122 mg, 55.0%).

1-(thiophen-1′-ylmethyl)benzene-2,4,6-triol ^{1}H NMR (Methanol-*d₄*, 600 MHz): δ = 7.31 (1H, dd, *J* = 2.9, 4.9 Hz, H-4′), 6.98 (1H, dd, *J* = 1.0, 4.9 Hz, H-5′), 6.87 (1H, dd, *J* = 1.0, 2.9 Hz, H-2′), 5.85(2H, s, H-3, H-5), 3.79 (2H, s, -CH_2-); ^{13}C NMR (Methanol-d_4, 150 MHz): δ = 156.3 (C-2), 156.3 (C-6), 156.0 (C-4), 142.8 (C-1′), 128.4, 123.4, 119.2, 106.2 (C-1), 93.9 (C-3), 93.9 (C-5), 22.7 (-CH_2-). HRESIMS (m/z): $[M+H]^+$ calcd. for $C_{11}H_{11}O_3S$, 223.0429; found, 223.0425.

Synthesis of 1-(2-(1*H*-indol-3-yl)ethyl)benzene-2,4,6-triol (43) (novel)

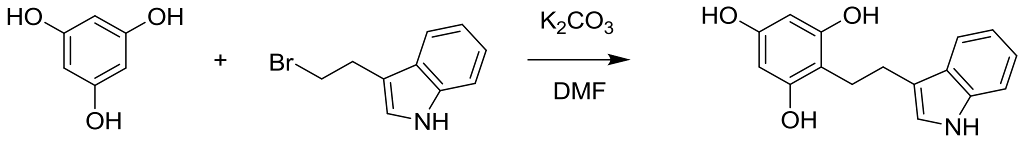

A solution of phloroglucinol (250 mg, 1.98 mmol) and K_2CO_3 (138 mg, 1.0 mmol) in DMF (1.2 ml) was stirred vigorously at 0 ℃ for 10 min. 3-(2-Bromoethyl)-1*H*-indole (223 mg, 1.0 mmol) in DMF (1.2 ml) was added dropwisely. The reaction mixture was stirred at 0 ℃ for 18 h. The mixture was poured in to water (6 ml), and extracted with EtOAc (6 ml×2). The combined organic phases were washed with brine (3 ml) and water (3 ml) successively. Then, the organic layer was dried (Na_2SO_4) and concentrated. The residue was purified by semi-HPLC to obtain 1-(2-(1*H*-indol-3-yl)ethyl)benzene-2,4,6-triol as an orange solid (137 mg, 50.9%).

1-(2-(1*H*-indol-3-yl)ethyl)benzene-2,4,6-triol ^{1}H NMR (DMSO-d_6, 600 MHz): δ = 10.74 (1H, s, -NH-), 7.47 (1H, d, *J* = 7.8 Hz, H-3′), 7.29 (1H, d, *J* = 8.0 Hz, H-6′), 7.09 (1H, d, *J* = 2.1 Hz, H-8′), 7.01 (1H, t, *J* = 8.0 Hz, H-4′), 6.93 (1H, t, *J* = 7.9 Hz, H-5′), 3.61 (2H, m, -CH_2-), 2.80 (2H, t, *J* = 7.4, -CH_2-); ^{13}C NMR (DMSO-d_6, 150 MHz): δ = 136.6, 136.6, 127.8, 123.2, 123.2, 121.2, 121.2, 118.7, 118.7, 118.5, 118.5, 112.0, 111.7, 111.7, 62.1, 29.3. HRESIMS (m/z): $[M+H]^+$ calcd. for $C_{16}H_{16}NO_3$, 270.1130; found, 270.1125.

Synthesis of 1-(quinoxalin-6-ylmethyl)benzene-2,4,6-triol (44) (novel)

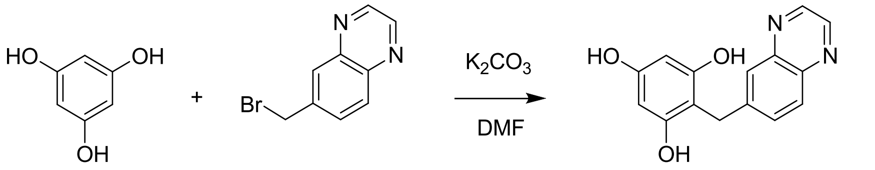

A solution of phloroglucinol (100 mg, 0.79 mmol) and K_2CO_3 (69 mg, 0.5 mmol) in DMF (0.6 ml) was stirred vigorously at 0 ℃ for 10 min. 6-(Bromomethyl)quinoxaline (90 mg, 0.41 mmol) in DMF (0.6 ml) was added dropwisely. The reaction mixture was stirred at 0 ℃ for 12 h. The mixture was poured in to water (3 ml), and extracted with EtOAc (2 ml×2). The combined organic phases were washed with brine (2 ml) and water (2 ml) successively. Then, the organic layer was dried (Na_2SO_4) and concentrated. The residue was purified by semi-HPLC to obtain 1-(quinoxalin-6-ylmethyl)benzene-2,4,6-triol as a brown powder (30 mg, 27.6%).

1-(quinoxalin-6-ylmethyl)benzene-2,4,6-triol ^{1}H NMR (DMSO-d_6, 600 MHz): δ = 9.14 (2H, s, -OH), 8.93 (1H, s, -OH), 8.84 (2H, d, J = 13.5 Hz, H-5′, H-7′), 7.95 (1H, d, J = 9.3 Hz, H-9′), 7.75–7.77 (2H, overlapped, H-2′, H-10′), 5.84 (2H, s, H-3, H-5), 3.96 (2H, s, -CH_2-); ^{13}C NMR (DMSO-d_6, 150 MHz): δ = 157.1 (C-4), 157.1 (C-2), 157.1 (C-6), 145.8, 145.8, 144.9, 142.7, 141.3, 132.3, 128.7, 127.3, 104.6 (C-1), 94.6 (C-3), 94.6 (C-5), 28.8 (-CH_2-). HRESIMS (m/z): $[M+H]^+$ calcd. for $C_{15}H_{13}N_2O_3$, 269.0926; found, 269.0919.

Synthesis of 1-((5'-(2*H*-tetrazol-11′-yl)biphenyl-1′-yl)methyl)benzene-2,4,6-triol (45p) (novel)

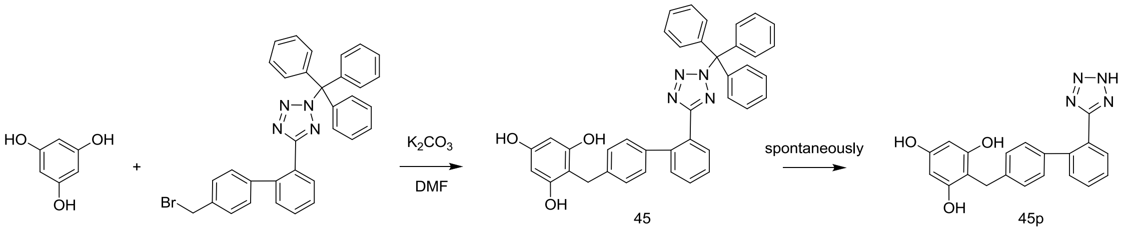

A solution of phloroglucinol (250 mg, 1.98 mmol) and K_2CO_3 (138 mg, 1.0 mmol) in DMF (1.2 ml) was stirred vigorously at 0 ℃ for 10 min. 5-[4'-(Bromomethyl)-1,1'-biphenyl-2-yl]-2- triphenylmethyl-2*H*-tetrazole (556 mg, 1.0 mmol) in DMF (1.2 ml) was added dropwisely. The reaction mixture was stirred at 0 ℃ for 18 h. The mixture was poured in to water (6 ml), and extracted with EtOAc (6 ml×2). The combined organic phases were washed with brine (3 ml) and water (3

ml) successively. Then, the organic layer was dried (Na_2SO_4) and concentrated. The residue was purified by semi-HPLC to obtain 1-((5'-(2*H*-tetrazol-11′-yl)biphenyl-1′-yl)methyl)benzene-2,4,6-triol as a brown solid (31 mg, 8.6%).

1-((5'-(2H-tetrazol-11′-yl)biphenyl-1′-yl)methyl)benzene-2,4,6-triol ^{1}H NMR (DMSO-d_6, 600 MHz): δ = 8.95 (2H, s, 2-OH, 4-OH), 8.80 (1H, s, 4-OH), 7.59 (2H, m, H-8′, H-9′), 7.47 (2H, m, H-7′, H-10′), 7.07 (2H, d, J = 8.2 Hz, H-2′/H-3′, H-6′/H-5′), 6.88 (2H, d , J = 8.2 Hz, H-2′/H-3′, H-6′/H-5′), 5.77 (2H, s, H-3, H-5), 3.66 (2H, s, -CH_2-); ^{13}C NMR (DMSO-d_6, 150 MHz): δ = 157.0 (C-2), 157.0 (C-6), 156.8 (C-4), 142.3, 141.9, 136.5, 131.2, 131.0, 131.0, 131.0, 131.0, 128.6, 128.6, 128.6, 128.6, 128.6, 128.6, 127.1 (C-11′), 105.1 (C-1), 94.5 (C-3), 94.5 (C-5), 28.1 (-CH_2-). HRESIMS (m/z): $[M+H]^+$ calcd. for $C_{20}H_{17}N_4O_3$, 361.1301; found, 361.1291.

Synthesis of 2-(4-methylbenzyl)benzene-1,3,5-triol (46) (novel)

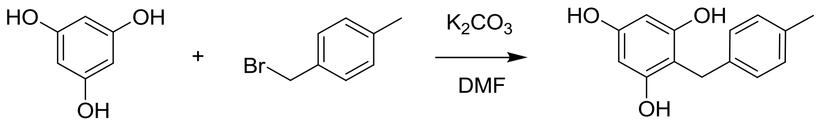

A solution of phloroglucinol (250 mg, 1.98 mmol) and K_2CO_3 (138 mg, 1.0 mmol) in DMF (1.2 ml) was stirred vigorously at 0 ℃ for 10 min. 4-Methylbenzyl bromide (185 mg, 1.0 mmol) in DMF (1.2 ml) was added dropwisely. The reaction mixture was stirred at 0 ℃ for 12 h. The mixture was poured in to water (6 ml), and extracted with EtOAc (3 ml×2). The combined organic phases were washed with brine (3 ml) and water (3 ml) successively. Then, the organic layer was dried (Na_2SO_4) and concentrated. The residue was purified by semi-HPLC to obtain 2-(4-methylbenzyl)benzene-1,3,5-triol as a light yellow powder (151 mg, 65.7%).

1-(4-methylbenzyl)benzene-2,4,6-triol ^{1}H NMR (DMSO-d_6, 600 MHz): δ = 8.87 (2H, s, -OH), 8.76 (1H, s, -OH), 7.04 (2H, d, J = 8.0 Hz, H-3′, H-5′), 6.95 (2H, d, J = 8.0 Hz, H-2′, H-6′), 5.77 (2H, brs, H-3, H-5), 3.63 (2H, s, -CH_2-), 2.18 (3H, s, -CH_3); ^{13}C NMR (DMSO-d_6, 150 MHz): δ = 156.9 (C-2), 156.9 (C-6), 156.6 (C-4), 140.1 (C-1′), 134.05 (C-4′), 128.7 (C-3′), 128.7 (C-5′), 128.6 (C-2′), 128.6 (C-6′), 105.7 (C-1), 94.5 (C-3), 94.5 (C-5), 28.1 (-CH_2-), 21.0 (-CH_3). HRESIMS (m/z): $[M+H]^+$ calcd. for $C_{14}H_{15}O_3$, 231.1021; found, 231.1018.

Synthesis of 1-(4-butylbenzyl)benzene-2,4,6-triol (47) (novel)

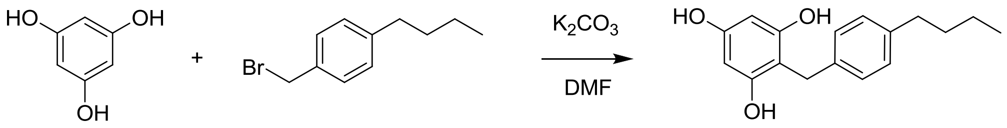

A solution of phloroglucinol (250 mg, 1.98 mmol) and K_2CO_3 (138 mg, 1.0 mmol) in DMF (1.2 ml) was stirred vigorously at 0 ℃ for 10 min. 4-Butylbenzylbromide (226 mg, 1.0 mmol) in DMF (1.2 ml) was added dropwisely. The reaction mixture was stirred at 0 ℃ for 18 h. The mixture was poured in to water (6 ml), and extracted with EtOAc (3 ml×2). The combined organic phases were washed with brine (3 ml) and water (3 ml) successively. Then, the organic layer was dried (Na_2SO_4) and concentrated. The residue was purified by semi-HPLC to obtain 1-(4-butylbenzyl)benzene-2,4,6-triol as a yellow solid (104 mg, 38.2%).

1-(4-butylbenzyl)benzene-2,4,6-triol ^{1}H NMR (DMSO-d_6, 600 MHz): δ = 8.91 (2H, s, -OH), 8.79 (1H, s, -OH), 7.07 (2H, d, J = 7.9 Hz, H-2′, H-6′), 6.98 (2H, d, J = 7.9 Hz, H-3′, H-5′), 5.79 (2H, s, H-3, H-5), 3.65 (2H, s, -CH_2-), 2.47 (2H, d, J = 7.6 Hz, -CH_2-), 1.49 (2H, m, -CH_2-), 1.27 (2H, m, -CH_2-), 0.87 (3H, t, J = 7.3 Hz, -CH_3); ^{13}C NMR (DMSO-d_6, 150 MHz): δ = 156.9 (C-2), 156.9 (C-6), 156.6 (C-4), 140.3, 139.2, 128.6, 128.6, 128.0, 128.0, 105.6 (C-1), 94.4 (C-3), 94.4 (C-5), 34.5 (-CH_2-), 33.8 (-CH_2-), 28.1 (-CH_2-), 22.2 (-CH_2-), 14.2 (-CH_3). HRESIMS (m/z): $[M+H]^+$ calcd. for $C_{17}H_{21}O_3$, 273.1491; found, 273.1484.

Synthesis of 1-(4-fluorobenzyl)benzene-2,4,6-triol (48) (novel)

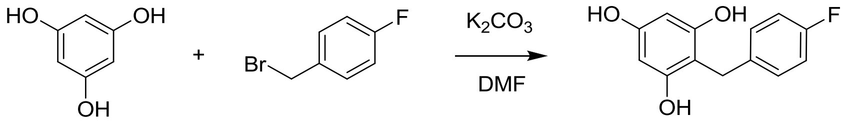

A solution of phloroglucinol (250 mg, 1.98 mmol) and K_2CO_3 (138 mg, 1.0 mmol) in DMF (1.2 ml) was stirred vigorously at 0 ℃ for 10 min. 4-fluorobenzyl bromide (185 mg, 0.98 mmol) in DMF (1.2 ml) was added dropwisely. The reaction mixture was stirred at 0 ℃ for 18 h. The mixture was poured in to water (6 ml), and extracted with EtOAc (3 ml×2). The combined organic phases were washed with brine (3 ml) and water (3 ml) successively. Then, the organic layer was dried (Na_2SO_4) and

concentrated. The residue was purified by semi-HPLC to obtain 1-(4-fluorobenzyl)benzene-2,4,6-triol as a yellow solid (110 mg, 48.0%).

1-(4-fluorobenzyl)benzene-2,4,6-triol ^{1}H NMR (DMSO-d_6, 600 MHz): δ = 8.99 (2H, s, -OH), 8.83 (1H, s, -OH), 7.19 (2H, m, H-3′, H-5′), 6.99 (2H, t, J = 8.9 Hz, H-2′, H-6′), 5.80 (2H, s, H-3, H-5), 3.67 (2H, s, -CH_2-); ^{13}C NMR (DMSO-d_6, 150 MHz): δ = 161.4 (C-4′), 156.9 (C-2), 156.9 (C-6), 156.8 (C-4), 139.2 (C-1′), 130.2 (C-2′), 130.2 (C-6′), 114.8 (C-3′), 114.8 (C-5′), 105.4 (C-1), 94.5 (C-3), 94.5 (C-5), 27.7 (-CH_2-). HRESIMS (m/z): $[M+H]^+$ calcd. for $C_{13}H_{12}FO_3$, 235.0770; found, 235.0766.

Synthesis of 2-(4-chlorobenzyl)benzene-1,3,5-triol (49) (novel)

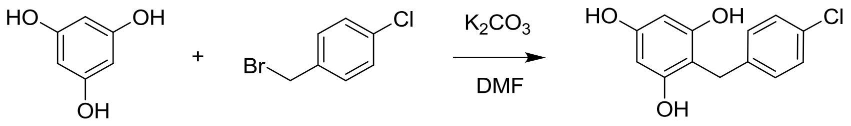

A solution of phloroglucinol (250 mg, 1.98 mmol) and K_2CO_3 (138 mg, 1.0 mmol) in DMF (1.2 ml) was stirred vigorously at 0 ℃ for 10 min. 4-Chlorobenzyl bromide (206 mg, 1.0 mmol) in DMF (1.2 ml) was added dropwisely. The reaction mixture was stirred at 0 ℃ for 12 h. The mixture was poured in to water (6 ml), and extracted with EtOAc (3 ml×2). The combined organic phases were washed with brine (3 ml) and water (3 ml) successively. Then, the organic layer was dried (Na_2SO_4) and concentrated. The residue was purified by semi-HPLC to obtain 2-(4-chlorobenzyl)benzene-1,3,5-triol as a brown solid (92 mg, 36.8%).

1-(4-chlorobenzyl)benzene-2,4,6-triol ^{1}H NMR (DMSO-d_6, 600 MHz): δ = 9.01 (2H, s, -OH), 8.85 (1H, s, -OH), 7.23 (2H, d, J = 8.5 Hz, H-3′, H-5′), 7.18 (2H, d, J = 8.5 Hz, H-2′, H-6′), 5.80 (2H, s, H-3, H-5), 3.68 (2H, s, -CH_2-); ^{13}C NMR (DMSO-d_6, 150 MHz): δ = 156.9 (C-2), 156.9 (C-6), 156.9 (C-4), 142.2 (C-1′), 130.5 (C-2′), 130.5 (C-6′), 129.9 (C-4′), 128.1 (C-3′), 128.1 (C-5′), 105.0 (C-1), 94.5 (C-3), 94.5 (C-5), 27.9 (-CH_2-). HRESIMS (m/z): $[M+H]^+$ calcd. for $C_{13}H_{12}ClO_3$, 251.0475; found, 251.0471.

Synthesis of 1-(biphenyl-4-ylmethyl)benzene-2,4,6-triol (50) (novel)

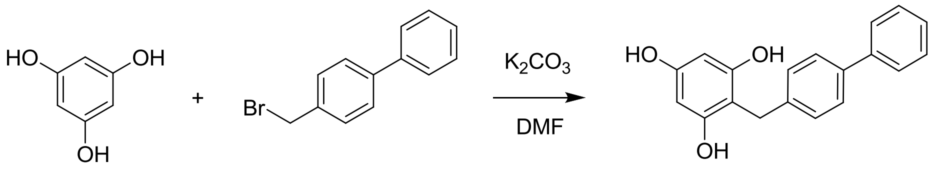

A solution of phloroglucinol (250 mg, 1.98 mmol) and K_2CO_3 (138 mg, 1.0 mmol) in DMF (1.2 ml) was stirred vigorously at 0 ℃ for 10 min. 4-(Bromomethyl)biphenyl (246 mg, 1.0 mmol) in DMF (1.2 ml) was added dropwisely. The reaction mixture was stirred at 0 ℃ for 18 h. The mixture was poured in to water (6 ml), and extracted with EtOAc (3 ml×2). The combined organic phases were washed with brine (3 ml) and water (3 ml) successively. Then, the organic layer was dried (Na_2SO_4) and concentrated. The residue was purified by semi-HPLC to obtain 1-(biphenyl-4-ylmethyl)benzene-2,4,6-triol as a yellow oil (90 mg, 30.8%).

1-(biphenyl-4-ylmethyl)benzene-2,4,6-triol ^{1}H NMR (DMSO-d_6, 600 MHz): δ = 9.02 (2H, s, -OH), 8.86 (1H, s, -OH), 7.58 (2H, dd, J = 1.3, 7.8 Hz, H-2″, H-6″), 7.47 (2H, d, J = 8.3 Hz, H-3′, H-5′), 7.42 (2H, t, J = 7.8 Hz, H-3″, H-5″), 7.31 (1H, t, J = 7.8 Hz, H-4″), 7.26 (2H, d, J = 8.3 Hz, H-2′, H-6′), 5.81 (2H, s, H-3, H-5), 3.74 (2H, s, -CH_2-); ^{13}C NMR (DMSO-d_6, 150 MHz): δ = 160.0 (C-2), 160.0 (C-6), 156.8 (C-4), 142.5, 140.9, 137.5, 129.3, 129.3, 129.2, 129.2, 127.4, 126.9, 126,9, 126.5, 126.5, 105.3 (C-1), 94.5 (C-3), 94.5 (C-5), 28.2 (-CH_2-). HRESIMS (m/z): $[M+H]^+$ calcd. for $C_{19}H_{17}O_3$, 293.1178; found, 293.1172.

Synthesis of 1-(4′-hydroxyphenethyl)benzene-2,4,6-triol (51) (novel)

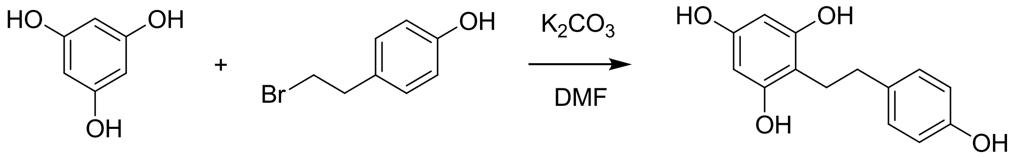

A solution of phloroglucinol (250 mg, 1.98 mmol) and K_2CO_3 (138 mg, 1.0 mmol) in DMF (1.2 ml) was stirred vigorously at 0 ℃ for 10 min. 4-(2-Bromoethyl)phenol (200 mg, 1.0 mmol) in DMF (1.2 ml) was added dropwisely. The reaction mixture was stirred at 0 ℃ for 18 h. The mixture was poured in to water (6 ml), and extracted with EtOAc (6 ml×2). The combined organic phases were washed with brine (3 ml) and water (3 ml) successively. Then, the organic layer was dried (Na_2SO_4) and concentrated. The residue was purified by semi-HPLC to obtain

1-(4′-hydroxyphenethyl)benzene-2,4,6-triol as a light brown powder (119 mg, 48.4%).

1-(4′-hydroxyphenethyl)benzene-2,4,6-triol ^{1}H NMR (Methanol-*d₄*, 600 MHz): δ = 7.03 (2H, dd, J = 2.0, 6.5 Hz, H-2′, H-6′), 6.65 (2H, dd, J = 2.0, 6.5 Hz, H-3′, H-5′), 5.84 (2H, s, H-3, H-5), 2.68 (2H, m, -CH_2-), 2.61 (2H, m, -CH_2-); ^{13}C NMR (Methanol-d_4, 150 MHz): δ = 156.3 (C-2), 156.3 (C-6), 155.5 (C-4), 154.6 (C-4′), 134.3 (C-1′), 128.9 (C-2′), 128.9 (C-6′), 107.0 (C-1), 94.0 (C-3), 94.0 (C-5), 34.5 (-CH_2-), 25.3 (-CH_2-). HRESIMS (m/z): $[M+H]^+$ calcd. for $C_{14}H_{15}O_4$, 247.0970; found, 247.0968.

Synthesis of 1-((3′,4′-dihydrobenzofuran-1′-yl)ethyl)benzene-2,4,6-triol (52) (novel)

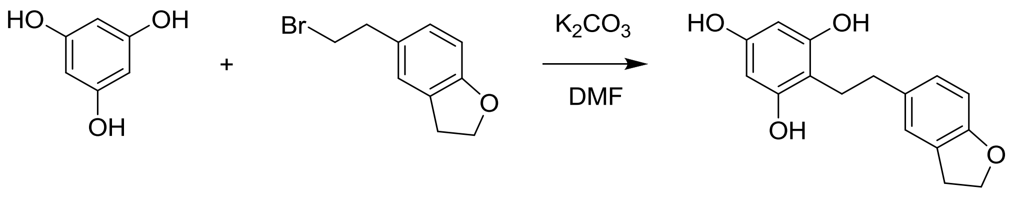

A solution of phloroglucinol (250 mg, 1.98 mmol) and K_2CO_3 (138 mg, 1.0 mmol) in DMF (1.2 ml) was stirred vigorously at room temperature for 10 min. 5-(2-Bromoethyl)-2,3-dihydrobenzofuran (226 mg, 1.0 mmol) in DMF (1.2 ml) was added dropwisely. The reaction mixture was stirred at room temperature for 18 h. The mixture was poured in to water (6 ml), and extracted with EtOAc (6 ml×2). The combined organic phases were washed with brine (3 ml) and water (3 ml) successively. Then, the organic layer was dried (Na_2SO_4) and concentrated. The residue was purified by semi-HPLC to obtain 1-((3′,4′-dihydrobenzofuran-1′-yl)ethyl)benzene-2,4,6-triol as a brown solid (13.9 mg, 5.1%).

1-((3′,4′-dihydrobenzofuran-1′-yl)ethyl)benzene-2,4,6-triol ^{1}H NMR (Methanol-d_4, 600 MHz): δ = 7.06 (1H, s, H-2′), 6.91 (1H, d, J = 8.1 Hz, H-6′), 6.57 (1H, d, J = 8.1 Hz, H-5′), 5.83 (2H, s, H-3, H-5), 4.46 (2H, t, J = 8.6, -CH_2-), 3.13 (2H, t, J = 8.6, -CH_2-), 2.68 (2H, m, -CH_2-), 2.62 (2H, m, -CH_2-); ^{13}C NMR (Methanol-d_4, 150 MHz): δ = 157.8, 156.3, 156.3, 155.5, 135.5, 127.2, 126.5, 124.5, 108.0, 107.0, 94.0 (C-3), 94.0 (C-5), 70.6 (-CH_2-), 34.8 (-CH_2-), 29.3 (-CH_2-), 25.5 (-CH_2-). HRESIMS (m/z): $[M+H]^+$ calcd. for $C_{16}H_{17}O_4$, 273.1127; found, 273.1127.

Synthesis of 1-(4′-fluorophenethyl)benzene-2,4,6-triol (53) (novel)

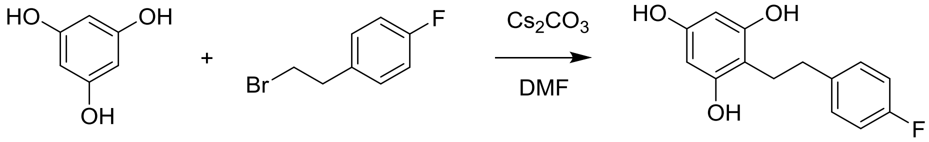

A solution of phloroglucinol (250 mg, 1.98 mmol) and Cs_2CO_3 (315 mg, 1.0 mmol) in DMF (1.2 ml) was stirred vigorously at 0 ℃ for 10 min. 4-Fluorophenethyl bromide (203 mg, 1.0 mmol) in DMF (1.2 ml) was added dropwisely. The reaction mixture was stirred at 0 ℃ for 18 h. The mixture was poured in to water (6 ml), and extracted with EtOAc (6 ml×2). The combined organic phases were washed with brine (3 ml) and water (3 ml) successively. Then, the organic layer was dried (Na_2SO_4) and concentrated. The residue was purified by semi-HPLC to obtain 1-(4′-fluorophenethyl)benzene-2,4,6-triol as a brown solid (25.3 mg, 10.2%).

1-(4′-fluorophenethyl)benzene-2,4,6-triol ^{1}H NMR (DMSO-d_6, 600 MHz): δ = 8.86 (3H, s, 2-OH, 4-OH, 6-OH), 7.17 (2H, dd, J = 8.3 Hz, H-2′/H-3′, H-6′/H-5′), 7.04 (2H, d, J = 8.3 Hz, H-3′/H-2′, H-5′/H-6′), 5.75 (2H, s, H-3, H-5), 2.59 (4H, brs, -CH_2-, -CH_2-); ^{13}C NMR (DMSO-d_6, 150 MHz): δ = 161.7 (C-4′), 156.9 (C-2), 156.9 (C-6), 156.4 (C-4), 139.4 (C-1′), 130.2, 130.2, 115.2, 115.1, 105.6 (C-1), 94.5 (C-3), 94.5 (C-5), 34.8 (-CH_2-), 25.2 (-CH_2-). HRESIMS (m/z): $[M+H]^+$ calcd. for $C_{14}H_{14}FO_3$, 249.0927; found, 249.0927.

Synthesis of 1-(4-chlorophenethyl)benzene-2,4,6-triol (54) (novel)

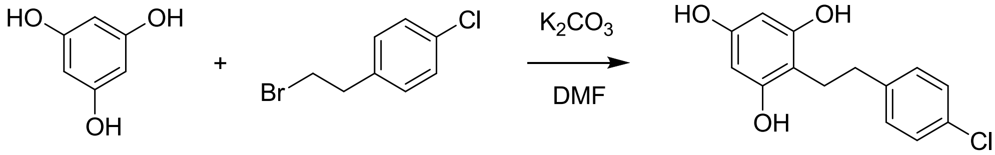

A solution of phloroglucinol (500 mg, 3.96 mmol) and K_2CO_3 (276 mg, 2.0 mmol) in DMF (2.4 ml) was stirred vigorously at 0 ℃ for 10 min. 1-(2-Bromoethyl)-4-chlorobenzene (440 mg, 2.0 mmol) in DMF (2.4 ml) was added dropwisely. The reaction mixture was stirred at 0 ℃ for 18 h. The mixture was poured in to water (12 ml), and extracted with EtOAc (12 ml×2). The combined organic phases were washed with brine (6 ml) and water (6 ml) successively. Then, the organic layer was dried (Na_2SO_4) and concentrated. The residue was purified by

semi-HPLC to obtain 1-(4-chlorophenethyl)benzene-2,4,6-triol as a yellow solid (11.6 mg, 2.2%).

1-(4-chlorophenethyl)benzene-2,4,6-triol ^{1}H NMR (DMSO-d_6, 600 MHz): δ = 8.90 (3H, s, -OH), 7.30 (2H, d, J = 8.2 Hz, H-3′, H-5′), 7.19 (2H, d, J = 8.2 Hz, H-2′, H-6′), 5.77 (2H, s, H-3, H-5), 2.63 (4H, m, -CH_2-, -CH_2-); ^{13}C NMR (DMSO-d_6, 150 MHz): δ = 156.9 (C-2), 156.9 (C-6), 156.4 (C-4), 142.3 (C-1′), 130.5 (C-2′), 130.5 (C-6′), 130.4 (C-4′), 128.4 (C-3′), 128.4 (C-5′), 105.5 (C-1), 94.5 (C-3), 94.5 (C-5), 34.9 (-CH_2-), 25.0 (-CH_2-). HRESIMS (m/z): $[M+H]^+$ calcd. for $C_{14}H_{14}ClO_3$, 265.0631; found, 265.0629.

Isolation protocol for the gram scale experiments

For the gram scale experiments catalyzed by whole cells, the cells were removed by centrifugation at 10,000 *g* for 30 min and washed twice with water. The clear supernatant containing product **46a** was passed through an Amberlite XAD-16 macroporous resin column (100 mL, Rohm & Haas Corp., Philadelphia, USA). Aliquots (500 mL) of ultrapure water, 50% (v/v) ethanol and 100% (v/v) ethanol were sequentially loaded into the column and eluted at a flow rate of 1 mL/min. The target glycoside was eluted with 50% (v/v) ethanol, and this fraction was concentrated under reduced pressure. The residue containing **46a** was dissolved in methanol and further purified by reversed-phase semipreparative HPLC (solvent A: H_2O containing 0.1% formic acid; solvent B: methanol; flow rate: 3 ml/min; gradient: 40−100% B, 20 min; 100% B, 5 min; 40% B, 5 min) to afford **46a**. The obtained product **46a** was analyzed by HRMS, ^{1}H NMR and ^{13}C NMR.

MS, ^{1}H NMR, ^{13}C NMR data of representative glucosylated products

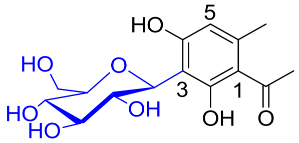

3-*C*-β-D-glucosyl-2,4-dihydroxy-6-methylacetophenone (9a) (novel)**:** ^{1}H NMR (DMSO-d_6, 600 MHz): δ = 6.22 (1H, s, H-5), 4.66 (1H, d, J = 9.8 Hz, Glc-H_1), 3.64 (2H, m, Glc-H), 3.52 (2H, dd, J = 11.9, 4.5 Hz, Glc-H), 3.24 (2H, m, Glc-H), 2.48 (3H, s, -CH_3), 2.24 (3H, s, -CH_3); ^{13}C NMR (DMSO-d_6, 150 MHz): δ = 204.2 (C=O), 159.7 (C-2), 159.6, 138.8, 118.9, 111.1, 110.3, 81.6 (Glc-5), 78.8 (Glc-3), 74.9 (Glc-1), 72.1 (Glc-2), 70.1 (Glc-4), 60.9 (Glc-6), 33.0 (-CH_3), 22.1 (-CH_3). HRESIMS (m/z): $[M+Na]^+$ calcd. for $C_{15}H_{20}NaO_8$, 351.1056; found, 351.1047.

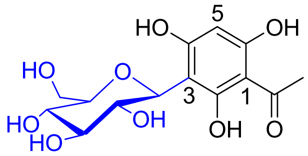

3-*C*-β-D-glucosyl-2,4,6-trihydroxyacetophenone (11a): ^{1}H NMR (DMSO-d_6, 600 MHz): δ = 5.93 (1H, s, H-5), 4.84 (1H, brs, Glc-OH), 4.80 (1H, brs, Glc-OH), 4.51 (1H, d, J = 9.8 Hz, Glc-H_1), 4.46 (1H, brs, Glc-OH), 3.87 (1H, t, J = 8.9 Hz, Glc-OH), 3.65 (1H, d, J = 9.8 Hz Glc-H), 3.41 (1H, m, Glc-H), 3.11–3.15 (4H, m, Glc-H), 2.56 (3H, s, -CH_3); ^{13}C NMR (DMSO-d_6, 150 MHz): δ = 203.0 (C=O), 165.3 (C-4), 164.3 (C-2), 162.4 (C-6), 104.4 (C-1), 104.4 (C-3), 94.9 (C-5), 81.8 (Glc-5), 79.4 (Glc-3), 74.0 (Glc-1), 71.1 (Glc-2), 70.9 (Glc-4), 61.7 (Glc-6), 33.0 (-CH_3). ESI-MS *m/z* 331.09 $[M+H]^+$.

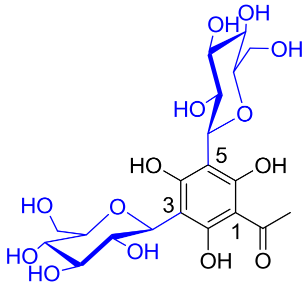

3,5-di-*C*-β-D-glucosyl-2,4,6-trihydroxyacetophenone (11aa): ^{1}H NMR (DMSO-d_6, 600 MHz): δ = 4.98 (2H, brs, -OH), 4.93 (1H, brs, -OH), 4.69 (2H, d, J = 9.8 Hz, Glc-H_1, Glc-$H_{1'}$), 3.63 (2H, d, J = 11.8 Hz, Glc-H), 3.57 (2H, dd, J = 11.8, 4.3 Hz, Glc-H), 3.51 (2H, t, J = 9.5 Hz, Glc-H), 3.22–3.29 (6H, m, Glc-H), 2.57 (3H, s, -CH_3); ^{13}C NMR (DMSO-d_6, 150 MHz): δ = 203.1 (C=O), 164.1 (C-4), 163.8 (C-2), 163.8 (C-6), 105.3 (C-1), 103.9 (C-3), 103.9 (C-5), 81.5 (Glc-5), 81.5 (Glc-5′), 78.5 (Glc-3),

78.5 (Glc-3′), 75.3 (Glc-1), 75.3 (Glc-1′), 72.7 (Glc-2), 72.7 (Glc-2′), 69.7 (Glc-4), 69.7 (Glc-4′), 60.5 (Glc-6), 60.5 (Glc-6′), 33.1 (-CH_3). ESI-MS *m/z* 491.46 $[M–H]^-$.

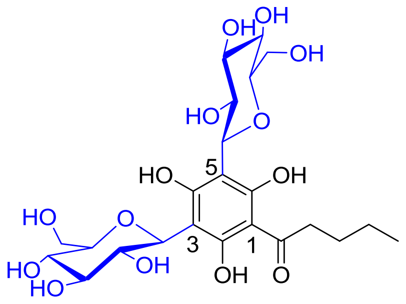

1-(3,5-di-*C*-β-D-glucosyl-2,4,6-trihydroxyphenyl)pentan-1′-one (16aa): ^{1}H NMR (DMSO-d_6, 600 MHz): δ = 9.11 (1H, s, -OH), 5.02 (2H, d, J = 5.3 Hz, Glc-OH), 4.97 (2H, d, J = 4.7 Hz, Glc-OH), 4.77 (2H, d, J = 5.2 Hz, Glc-OH), 4.71 (2H, overlapped, Glc-OH), 4.71 (2H, overlapped, J = 10.0 Hz Glc-H_1, Glc-$H_{1'}$), 3.57–3.65 (4H, m, Glc-H), 3.47 (2H, m, Glc-H), 3.04 (2H, m, -CH_2-), 1.58 (2H, m, -CH_2-), 1.33 (2H, m, -CH_2-), 0.90 (3H, t, J = 7.3 Hz, -CH_3); ^{13}C NMR (DMSO-d_6, 150 MHz): δ = 206.1 (C=O), 161.0 (C-4), 160.9 (C-2), 160.9 (C-6), 104.6 (C-1), 103.9 (C-3), 103.9 (C-5), 81.0 (Glc-5), 81.0 (Glc-5′), 77.7 (Glc-3), 77.7 (Glc-3′), 74.5 (Glc-1), 74.5 (Glc-1′), 71.9 (Glc-2), 71.9 (Glc-2′), 69.1 (Glc-4), 69.1 (Glc-4′), 59.8 (Glc-6), 59.8 (Glc-6′), 43.2 (-CH_2-), 26.3 (-CH_2-), 21.8 (-CH_2-), 13.8 (-CH_3). ESI-MS *m/z* 535.13 $[M+H]^+$.

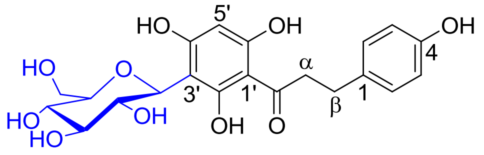

phloretin-3′-*C*-β-D-glucoside (17a): ^{1}H NMR (DMSO-d_6, 600 MHz): δ = 13.78 (1H, s, -OH), 11.04 (1H, s, -OH), 10.07 (1H, s, -OH), 9.14 (1H, s, -OH), 7.03 (2H, d, J = 8.4 Hz, H-2, H-6), 6.67 (2H, d, J = 8.4 Hz, H-3, H-5), 5.95 (1H, s, H-5′), 4.84 (1H, brs, Glc-OH), 4.80 (1H, brs, Glc-OH), 4.52 (1H, d, J = 9.8 Hz, Glc-H_1), 4.50 (1H, d, J = 5.3 Hz, Glc-OH), 4.46 (1H, t, J = 5.7 Hz, Glc-OH), 3.88 (1H, m, Glc-H), 3.65 (1H, dd, J = 4.7, 11.6 Hz, Glc-H), 3.42 (1H, m, Glc-H), 3.23 (2H, t, J = 7.6 Hz, H-α), 3.15–3.17 (3H, m, Glc-H), 2.77 (2H, t, J = 7.6 Hz, H-β); ^{13}C NMR (DMSO-d_6, 150 MHz): δ = 204.9 (C=O), 165.2 (C-4′), 164.1 (C-2′), 162.0 (C-6′), 155.8 (C-4), 132.1 (C-1), 129.6 (C-2), 129.6 (C-6), 115.5 (C-3), 115.5 (C-5), 104.5 (C-3′), 104.0 (C-1′), 95.0 (C-5′), 81.8 (Glc-5), 79.4 (Glc-3), 74.0 (Glc-1), 71.1 (Glc-2), 70.9 (Glc-4), 61.7 (Glc-6), 46.1 (C-α), 30.0 (C-β). ESI-MS *m/z* 437.08 $[M+H]^+$.

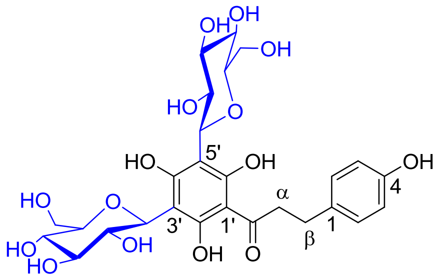

phloretin-3′,5′-di-*C*-β-D-glucoside (17aa): ^{1}H NMR (DMSO-d_6, 600 MHz): δ = 9.14 (2H, s, -OH), 7.03 (2H, d, J = 8.4 Hz, H-2, H-6), 6.67 (2H, d, J = 8.4 Hz, H-3, H-5), 5.02 (2H, d, J = 5.3 Hz, Glc-OH), 4.98 (2H, brs, Glc-OH), 4.77 (2H, brs, Glc-OH), 4.72 (2H, d, J = 9.5 Hz, Glc-H_1), 4.71 (2H, overlapped, Glc-OH), 3.57–3.63 (4H, m, Glc-H), 3.47 (2H, m, Glc-H), 3.26–3.29 (6H, m, Glc-H), 3.17 (2H, m, H-α), 2.79 (2H, t, J = 7.7 Hz, H-β); ^{13}C NMR (DMSO-d_6, 150 MHz): δ = 205.6 (C=O), 161.6 (C-4′), 161.5 (C-2′), 161.5 (C-6′), 155.9 (C-4), 132.0 (C-1), 129.7 (C-2), 129.6 (C-6), 115.5 (C-3), 115.5 (C-5), 105.0 (C-1), 104.5 (C-3′), 104.5 (C-5′), 81.6 (Glc-5), 81.6 (Glc-5′), 78.3 (Glc-3), 78.3 (Glc-3′), 75.1 (Glc-1), 75.1 (Glc-1′), 72.5 (Glc-2), 72.5 (Glc-2′), 69.6 (Glc-4), 69.6 (Glc-4′), 60.4 (Glc-6), 60.4 (Glc-6′), 46.7 (C-α), 29.7 (C-β). ESI-MS *m/z* 599.09 $[M+H]^{+}$.

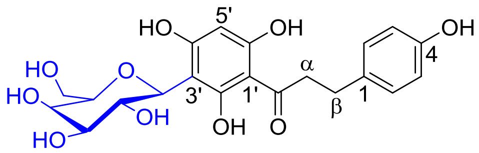

phloretin-3′-*C*-β-D-galactoside (17b): ^{1}H NMR (DMSO-d_6, 600 MHz): δ =7.02 (2H, d, J = 8.4 Hz, H-2, H-6), 6.65 (2H, d, J = 8.4 Hz, H-3, H-5), 5.80 (1H, s, H-5′), 4.58 (1H, d, J = 9.7 Hz, Gal-H_1), 3.88 (1H, m, Gal-H), 3.74 (1H, d, J = 2.8 Hz, Gal-H), 3.47 (1H, m, Gal-H), 3.43 (1H, m, Gal-H), 3.33–3.35 (2H, m, Gal-H), 3.23 (2H, t, J = 7.9 Hz, H-α), 2.77 (2H, t, J = 7.9 Hz, H-β); ^{13}C NMR (DMSO-d_6, 150 MHz): δ = 204.6 (C=O), 165.5 (C-4′), 163.8 (C-2′), 163.2 (C-6′), 155.8 (C-4), 132.2 (C-1), 129.6 (C-2), 129.6 (C-6), 115.5 (C-3), 115.5 (C-5), 105.0 (C-3′), 104.4 (C-1′), 94.7 (C-5′), 79.6 (Gal-5), 75.5 (Gal-3), 74.9 (Gal-1), 70.2 (Gal-2), 69.1 (Gal-4), 61.2 (Gal-6), 46.0 (C-α), 30.0 (C-β). ESI-MS *m/z* 435.49 $[M–H]^{-}$.

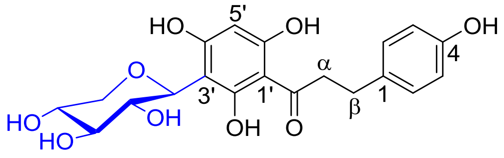

phloretin-3′-*C*-β-D-xyloside (17c): ^{1}H NMR (DMSO-d_6, 600 MHz): δ =7.00 (2H, d, J = 8.4 Hz, H-2, H-6), 6.63 (2H, d, J = 8.4 Hz, H-3, H-5), 5.91 (1H, s, H-5′), 4.37 (1H, d, J = 9.8 Hz, Xyl-H$_1$), 3.93 (1H, t, J = 9.2 Hz Xyl-H), 3.67 (1H, dd, J = 5.4, 10.9 Hz, Xyl-H), 3.31 (1H, overlapped, Xyl-H), 3.19 (2H, t, J = 7.7 Hz, H-α), 3.05 (1H, t, J = 8.8 Hz, Xyl-H), 2.97 (1H, t, J = 10.7 Hz, Xyl-H), 2.73 (2H, t, J = 7.7 Hz, H-β); ^{13}C NMR (DMSO-d_6, 150 MHz): δ = 204.8 (C=O), 165.8 (C-4′), 164.3 (C-2′), 161.9 (C-6′), 155.8 (C-4), 132.1 (C-1), 129.6 (C-2), 129.6 (C-6), 115.5 (C-3), 115.5 (C-5), 104.4 (C-3′), 103.9 (C-1′), 94.8 (C-5′), 79.7 (Xyl-2), 74.7 (Xyl), 70.7 (Xyl), 70.7 (Xyl), 70.4 (Xyl), 46.0 (C-α), 30.0 (C-β). ESI-MS *m/z* 405.33 [M–H]$^{-}$.

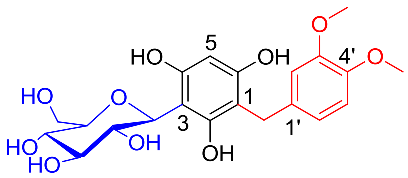

3-*C*-β-D-glucosyl-1-(3,4-dimethoxybenzyl)benzene-2,4,6-triol (20a) (novel) ^{1}H NMR (DMSO-d_6, 600 MHz): δ = 9.05 (1H, s, -OH), 8.78 (1H, s, -OH), 7.96 (1H, s, -OH), 6.88 (1H, d, J = 1.9 Hz, H-2′), 6.75 (1H, d, J = 8.2 Hz, H-5′), 6.68 (1H, dd, J = 8.2, 1.9 Hz, H-6′), 5.93 (1H, s, H-5), 4.96 (1H, s, Glc-OH), 4.89 (1H, s, Glc-OH), 4.77 (1H, s, Glc-OH), 4.67 (1H, s, Glc-OH), 4.61 (1H, d, J = 9.7 Hz, Glc-H$_1$), 3.67 (8H, overlapped, -CH$_3$, -CH$_3$, -CH$_2$-), 3.62 (1H, m, Glc-H), 3.57 (1H, m, Glc-H), 3.20–3.32 (4H, m, Glc-H); ^{13}C NMR (DMSO-d_6, 150 MHz): δ = 155.9, 155.3, 154.4, 148.7 (C-3′), 147.0 (C-4′), 135.5 (C-1′), 120.6, 113.1, 112.1, 107.1, 103.7 (C-3), 95.3 (C-5), 81.3 (Glc-5), 78.6 (Glc-3), 75.9 (Glc-1), 73.1 (Glc-2), 69.7 (Glc-4), 60.5 (Glc-6), 56.0 (-CH$_3$), 55.8 (-CH$_3$), 28.1 (-CH$_2$-). HRESIMS (m/z): [M+H]$^{+}$ calcd. for $C_{21}H_{27}O_{10}$, 439.1604; found, 439.1599.

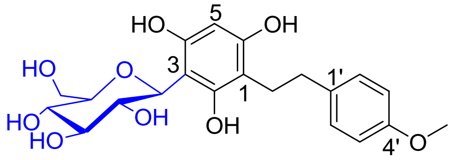

3-*C*-β-D-glucosyl-1-(4′-methoxyphenethyl)benzene-2,4,6-triol (21a) (novel) ^{1}H NMR (DMSO-d_6, 600 MHz): δ = 8.94 (1H, s, -OH), 8.71 (1H, s, -OH), 7.91 (1H, s, -OH), 7.10 (2H, d, J = 8.5 Hz, H-2′, H-6′), 6.81 (2H, d, J = 8.5 Hz, H-3′, H-5′), 5.89 (1H, s, H-5), 4.59 (1H, d, J = 9.8 Hz, Glc-H$_1$), 3.69 (3H, s, -CH$_3$), 3.55–3.63 (2H, m, Glc-H), 3.39 (1H, t, J = 9.1 Hz, Glc-H), 3.18–3.22 (3H, m, Glc-H), 2.54–2.61 (4H, m,

-CH_2-, -CH_2-); ^{13}C NMR (DMSO-d_6, 150 MHz): δ = 157.7 (C-4′), 155.9, 155.3, 154.2, 135.3 (C-1′), 129.4 (C-2′), 129.4 (C-6′), 114.1 (C-3′), 114.1 (C-5′), 106.0 (C-1), 103.7 (C-3), 95.3 (C-5), 81.4 (Glc-5), 78.6 (Glc-3), 75.9 (Glc-1), 73.1 (Glc-2), 69.7 (Glc-4), 60.6 (Glc-6), 55.4 (-CH_3), 34.9 (-CH_2-), 25.8 (-CH_2-). HRESIMS (m/z): $[M+H]^+$ calcd. for $C_{21}H_{27}O_9$, 423.1655; found, 423.1649.

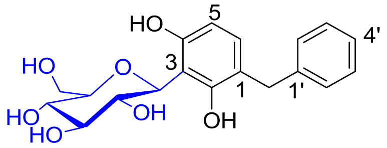

3-*C*-β-D-glucosyl-1-benzylbenzene-2,4-diol (28a) (novel) ^{1}H NMR (Methanol-d_4, 600 MHz): δ = 7.17–7.20 (4H, m, overlapped, H-2′, H-3′, H-5′, H-6′), 7.09 (1H, m, H-4′), 6.78 (1H, d, *J* = 8.3 Hz, H-6), 6.28 (1H, d, *J* = 8.3 Hz, H-5), 4.94 (1H, d, *J* = 9.9 Hz, Glc-H_1), 3.85 (1H, dd, *J* = 2.3, 12.1 Hz, Glc-H), 3.82 (2H, d, *J* = 6.6 Hz, -CH_2-), 3.78 (1H, dd, *J* = 4.5, 12.1 Hz, Glc-H), 3.65 (1H, dd, *J* = 8.8 Hz, Glc-H), 3.50 (2H, m, Glc-H), 3.41 (1H, m, Glc-H); ^{13}C NMR (Methanol-d_4, 150 MHz): δ = 154.3, 154.0, 141.8 (C-1′), 129.9, 128.4 (C-3′), 128.4 (C-5′), 127.6 (C-2′), 127.6 (C-6′), 125.1, 120.3, 111.0, 106.9, 81.1 (Glc-5), 78.2 (Glc-3), 76.2 (Glc-1), 73.1 (Glc-2), 69.6 (Glc-4), 60.6 (Glc-6), 34.7 (-CH_2-). HRESIMS (m/z): $[M+H]^+$ calcd. for $C_{19}H_{23}O_7$, 363.1444; found, 363.1438.

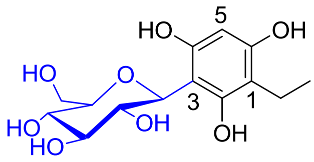

ethyl phloroglucinol-3-*C*-β-D-glucoside (30a) ^{1}H NMR (DMSO-d_6, 600 MHz): δ = 8.86 (1H, s, -OH), 8.65 (1H, s, -OH), 7.86 (1H, s, -OH), 5.88 (1H, s, H-5), 4.95 (1H, d, *J* = 4.6 Hz, Glc-OH), 4.76 (1H, d, *J* = 5.5 Hz, Glc-OH), 4.67 (1H, t, *J* = 5.3 Hz, Glc-OH), 4.60 (1H, d, *J* = 9.8 Hz, Glc-H_1), 3.63 (1H, m, Glc-H), 3.57 (1H, m, Glc-H), 3.39 (1H, m, Glc-H), 3.28 (1H, m, Glc-H), 3.21 (2H, m, Glc-H), 2.41 (2H, m, -CH_2-), 0.96 (3H, t, *J* = 7.3 Hz), ^{13}C NMR (DMSO-d_6, 150 MHz): δ = 155.7, 155.1, 153.9, 109.2, 103.6, 95.2 (C-5), 81.3 (Glc-5), 78.6 (Glc-3), 75.9 (Glc-1), 73.1 (Glc-2), 69.7 (Glc-4), 60.6 (Glc-6), 16.1 (-CH_2-), 14.7 (-CH_3). ESI-MS *m/z* 315.03 $[M–H]^-$.

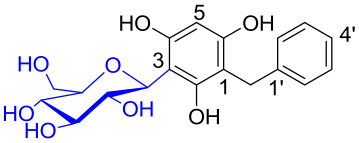

3-*C*-β-D-glucosyl-1-benzylbenzene-2,4,6-triol (38a) (novel) ^{1}H NMR (DMSO-d_6, 600 MHz): δ = 9.04 (1H, s, -OH), 8.77 (1H, s, -OH), 7.95 (1H, s, -OH), 7.14–7.18 (4H, m, H-2′, H-3′, H-5′, H-6′), 7.06 (1H, m, H-4′), 5.91 (1H, s, H-5), 4.58 (1H, d, *J* = 9.7 Hz, Glc-H_1), 3.70 (2H, s, -CH_2-), 3.53–3.60 (2H, m, Glc-H), 3.16–3.21 (4H, m, overlapped, Glc-H); ^{13}C NMR (DMSO-d_6, 150 MHz): δ = 156.0, 155.4, 154.6, 142.9 (C-1′), 128.8 (C-3′), 128.8 (C-5′), 128.2 (C-2′), 128.2 (C-6′), 125.4 (C-4′), 106.7 (C-1), 103.6 (C-3), 95.2 (C-5), 81.4 (Glc-5), 78.6 (Glc-3), 75.9 (Glc-1), 73.1 (Glc-2), 69.7 (Glc-4), 60.5 (Glc-6), 25.6 (-CH_2-). ESI-MS *m/z* 377.33 $[M–H]^-$. HRESIMS (m/z): $[M+H]^+$ calcd. for $C_{19}H_{23}O_8$, 379.1393; found, 379.1383.

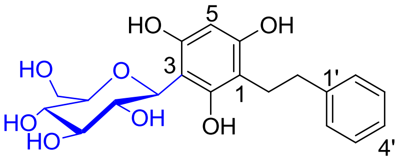

3-*C*-β-D-glucosyl-1-phenethylbenzene-2,4,6-triol (39a) (novel) ^{1}H NMR (DMSO-d_6, 600 MHz): δ = 8.91 (1H, s, -OH), 8.79 (1H, s, -OH), 7.94 (1H, s, -OH), 7.22–7.26 (2H, m, H-3′, H-5′), 7.15 (2H, t, *J* = 7.5 Hz, H-2′, H-6′), 7.03 (1H, *J* = 7.5 Hz, H-4′), 5.85 (1H, s, H-5), 4.59 (1H, d, *J* = 9.7 Hz, Glc-H_1), 3.53–3.60 (2H, m, Glc-H), 3.17–3.21 (4H, m, overlapped, Glc-H), 1.55–1.56 (4H, m, -CH_2-, -CH_2-); ^{13}C NMR (DMSO-d_6, 150 MHz): δ = 156.3, 155.3, 154.4, 147.3 (C-1′), 127.9 (C-3′), 127.9 (C-5′), 127.8 (C-2′), 127.8 (C-6′), 125.1 (C-4′), 111.4 (C-1), 103.6 (C-3), 94.9 (C-5), 81.4 (Glc-5), 78.6 (Glc-3), 76.0 (Glc-1), 73.1 (Glc-2), 69.7 (Glc-4), 60.5 (Glc-6), 33.3 (-CH_2-), 18.7 (-CH_2-). HRESIMS (m/z): $[M+H]^+$ calcd. for $C_{20}H_{25}O_8$, 393.1549; found, 393.1538.

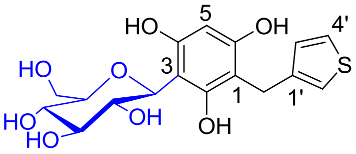

3-*C*-β-D-glucosyl-1-(thiophen-1′-ylmethyl)benzene-2,4,6-triol (42a) (novel) ^{1}H NMR (Methanol-d_4, 600 MHz): δ = 7.13 (1H, dd, *J* = 2.9, 4.9 Hz, H-4′), 7.00 (1H, dd, *J* = 0.9, 4.9 Hz, H-5′), 6.91 (1H, dd, *J* = 0.9, 2.9 Hz, H-2′), 5.96 (1H, s, H-5), 4.82 (1H, d, *J* = 9.8 Hz, Glc-H_1), 3.84 (1H, dd, *J* = 2.2, 12.1 Hz, Glc-H), 3.82 (2H, d, *J* = 2.3 Hz,

-CH_2-), 3.76 (1H, dd, *J* = 4.7, 12.1 Hz, Glc-H), 3.61 (1H, dd, *J* = 9.6 Hz, Glc-H), 3.45 (2H, m, overlapped, Glc-H), 3.38 (1H, m, Glc-H); ^{13}C NMR (Methanol-d_4, 150 MHz): δ = 155.8, 154.9, 154.2, 142.6, 128.6, 123.5, 119.5, 107.1, 102.8 (C-3), 94.8 (C-5), 81.1 (Glc-5), 78.1 (Glc-3), 76.2 (Glc-1), 73.3 (Glc-2), 69.7 (Glc-4), 60.7 (Glc-6), 22.7 (-CH_2-). HRESIMS (m/z): $[M+H]^+$ calcd. for $C_{17}H_{21}O_8S$, 385.0957; found, 385.0946.

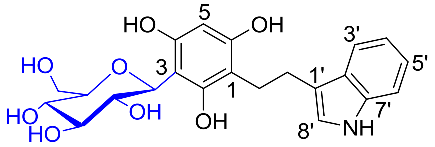

3-*C*-β-D-glucosyl-1-(2-(1*H*-indol-3-yl)ethyl)benzene-2,4,6-triol (43a) (novel) ^{1}H NMR (DMSO-d_6, 600 MHz): δ = 10.67 (1H, s, -NH-), 9.01 (1H, s, -OH), 8.72 (1H, s, -OH), 7.94 (1H, s, -OH), 7.60 (1H, d, *J* = 7.3 Hz, H-3′), 7.29 (1H, d, *J* = 7.3 Hz, H-6′), 7.09 (1H, d, *J* = 1.8 Hz, H-8′), 7.02 (1H, t, *J* = 7.3 Hz, H-4′/ H-5′), 6.93 (1H, t, *J* = 7.3 Hz, H-5′/ H-4′), 5.92 (1H, d, *J* = 9.8 Hz, Glc-H_1), 3.59 (2H, m, Glc-H), 3.41 (2H, m, overlapped, Glc-H), 3.21 (2H, m, overlapped, Glc-H), 2.72 (4H, m, -CH_2-, -CH_2-); ^{13}C NMR (DMSO-d_6, 150 MHz): δ = 156.0, 155.4, 154.1, 136.7, 127.8, 122.1, 121.2, 119.0, 118.4, 116.1, 111.7, 108.0, 103.7, 95.3 (C-5), 81.4 (Glc-5), 78.7 (Glc-3), 76.0 (Glc-1), 73.2 (Glc-2), 69.7 (Glc-4), 60.6 (Glc-6), 29.5 (-CH_2-), 25.7 (-CH_2-). HRESIMS (m/z): $[M+H]^+$ calcd. for $C_{22}H_{25}NNaO_8$, 454.1478; found, 454.1474.

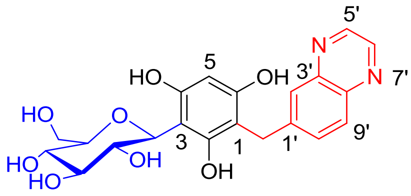

3-*C*-β-D-glucosyl-1-(quinoxalin-6-ylmethyl)benzene-2,4,6-triol (44a) (novel) ^{1}H NMR (DMSO-d_6, 600 MHz): δ = 9.14 (1H, s, -OH), 8.87 (1H, s, -OH), 8.82 (2H, d, *J* = 13.8 Hz, H-5′, H-7′), 8.07 (1H, s, -OH), 7.93 (1H, d, *J* = 8.6 Hz, H-9′), 7.78 (1H, brs, H-2′), 7.75 (1H, dd, *J* = 1.9, 8.6 Hz, H-10′), 5.96 (1H, s, H-5), 4.93 (1H, brs, Glc-OH), 4.85 (1H, brs, Glc-OH), 4.63 (1H, brs, Glc-OH), 4.61 (1H, d, *J* = 9.7 Hz, Glc-H_1), 4.00 (2H, m, -CH_2-), 3.60 (2H, m, Glc-H), 3.25-3.41(4H, m, Glc-H); ^{13}C NMR (DMSO-d_6, 150 MHz): δ = 156.1, 155.5, 155.0, 145.8, 145.5, 145.0, 142.7, 141.3, 132.4, 128.8, 127.4, 105.8 (C-1), 103.8 (C-3), 95.3 (C-5), 81.4 (Glc-5), 78.6 (Glc-3), 75.9 (Glc-1), 73.2 (Glc-2), 69.7 (Glc-4), 60.5 (Glc-6), 28.9 (-CH_2-). HRESIMS (m/z): $[M+H]^+$ calcd. for $C_{21}H_{23}N_2O_8$, 431.1454; found, 431.1447.

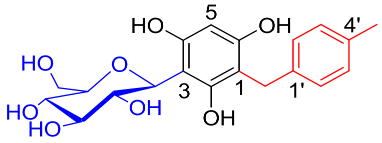

3-*C*-β-D-glucosyl-1-(4-methylbenzyl)benzene-2,4,6-triol (46a) (novel) (6.1 mg, isolated yield: 88%) ^{1}H NMR (DMSO-d_6, 600 MHz): δ = 9.03 (1H, s, -OH), 8.77 (1H, s, -OH), 7.95 (1H, s, -OH), 7.08 (2H, d, J = 7.9 Hz, H-3′, H-5′), 6.98 (2H, d, J = 7.9 Hz, H-2′, H-6′), 5.93 (1H, s, H-5), 4.95 (1H, s, Glc-OH), 4.87 (1H, s, Glc-OH), 4.77 (1H, s, Glc-OH), 4.67 (1H, s, Glc-OH), 4.61 (1H, d, J = 9.7 Hz, Glc-H$_1$), 3.64 (2H, s, -CH$_2$-), 3.56–3.64 (2H, m, Glc-H), 3.17–3.39 (4H, m, Glc-H), 2.21 (3H, s, -CH$_3$); ^{13}C NMR (DMSO-d_6, 150 MHz): δ = 155.9 (C-6), 155.7 (C-6), 154.5 (C-4), 139.8 (C-1′), 134.14 (C-4′), 128.7 (C-2′), 128.7 (C-3′), 128.7 (C-5′), 128.7 (C-6′), 107.0 (C-1), 103.6 (C-3), 95.2 (C-5), 81.4 (Glc-5), 78.6 (Glc-3), 75.9 (Glc-1), 73.1 (Glc-2), 69.7 (Glc-4), 60.5 (Glc-6), 28.2 (-CH$_2$-), 21.0 (-CH$_3$). HRESIMS (m/z): [M+H]$^+$ calcd. for $C_{20}H_{25}O_8$, 393.1549; found, 393.1540.

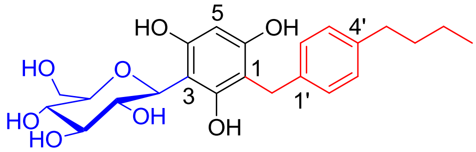

3-*C*-β-D-glucosyl-1-(4-butylbenzyl)benzene-2,4,6-triol (47a) (novel) ^{1}H NMR (DMSO-d_6, 600 MHz): δ = 9.02 (1H, s, -OH), 8.76 (1H, s, -OH), 7.93 (1H, s, -OH), 7.07 (2H, d, J = 7.8 Hz, H-2′, H-6′), 6.96 (2H, d, J = 7.8 Hz, H-3′, H-5′), 5.90 (1H, s, H-5), 4.58 (1H, d, J = 9.7 Hz, Glc-H$_1$), 3.66 (2H, s, -CH$_2$-), 3.60 (1H, d , J = 10.7 Hz, Glc-H), 3.54 (1H, dd, J = 11.7, 4.3 Hz, Glc-H), 3.27 (2H, m, overlapped, Glc-H), 3.16–3.21 (2H, m, Glc-H), 2.45 (2H, overlapped, -CH$_2$-), 1.46 (2H, m, -CH$_2$-), 1.25 (2H, m, -CH$_2$-), 0.84 (3H, t, J = 7.4 Hz, -CH$_3$); ^{13}C NMR (DMSO-d_6, 150 MHz): δ = 155.9, 155.3, 154.5, 140.0, 139.2, 128.7, 128.7, 128.1, 128.1, 106.9 (C-1), 103.6 (C-3), 95.2 (C-5), 81.4 (Glc-5), 78.6 (Glc-3), 75.9 (Glc-1), 73.1 (Glc-2), 69.7 (Glc-4), 60.5 (Glc-6), 34.9 (-CH$_2$-), 33.8 (-CH$_2$-), 28.2 (-CH$_2$-), 22.2 (-CH$_2$-), 14.2 (-CH$_3$). HRESIMS (m/z): [M+H]$^+$ calcd. for $C_{23}H_{31}O_8$, 435.2019; found, 435.2007.

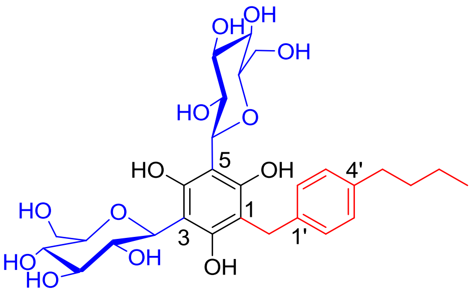

3,5-di-*C*-β-D-glucosyl-1-(4-butylbenzyl)benzene-2,4,6-triol (47aa) (novel) ^{1}H NMR (DMSO-d_6, 600 MHz): δ = 8.33 (1H, s, -OH), 8.17 (2H, s, -OH), 7.07 (2H, d, J = 7.9 Hz, H-2′, H-6′), 6.96 (2H, d, J = 7.9 Hz, H-3′, H-5′), 4.67 (2H, d, J = 9.8 Hz, Glc-H_1), 3.72 (2H, d, J = 6.8 Hz, -CH_2-), 3.58 (4H, m, Glc-H), 3.21–3.25 (8H, m, overlapped, Glc-H), 2.45 (2H, overlapped, -CH_2-), 1.47 (2H, m, -CH_2-), 1.25 (2H, m, -CH_2-), 0.84 (3H, t, J = 7.4 Hz, -CH_3); ^{13}C NMR (DMSO-d_6, 150 MHz): δ = 154.3 (C-2), 154.3 (C-4), 154.3 (C-6), 139.6, 139.4, 128.7, 128.7, 128.1, 128.1, 108.5 (C-1), 105.1 (C-3), 105.1 (C-5), 81.4 (Glc-5), 81.4 (Glc-5′), 78.3 (Glc-3), 78.3 (Glc-3′), 76.3 (Glc-1), 76.3 (Glc-1′), 73.6 (Glc-2), 73.6 (Glc-2′), 69.5 (Glc-4), 69.5 (Glc-4′), 60.3 (Glc-6), 60.3 (Glc-6′), 34.9 (-CH_2-), 33.7 (-CH_2-), 28.2 (-CH_2-), 22.2 (-CH_2-), 14.2 (-CH_3). HRESIMS (m/z): $[M+H]^+$ calcd. for $C_{29}H_{41}O_{13}$, 597.2547; found, 597.2545.

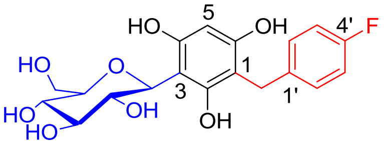

3-*C*-β-D-glucosyl-1-(4-fluorobenzyl)benzene-2,4,6-triol (48a) (novel) ^{1}H NMR (DMSO-d_6, 600 MHz): δ = 9.15 (1H, s, -OH), 8.85 (1H, s, -OH), 8.00 (1H, s, -OH), 7.21 (2H, m, H-3′, H-5′), 7.00 (2H, t, J = 8.9 Hz, H-2′, H-6′), 5.94 (1H, s, H-5), 4.61 (1H, d, J = 9.6 Hz, Glc-H_1), 3.71 (2H, s, -CH_2-), 3.62 (1H, m, Glc-H), 3.57 (1H, m, Glc-H), 3.20–3.39 (4H, m, Glc-H); ^{13}C NMR (DMSO-d_6, 150 MHz): δ = 161.4, 155.9, 155.3, 154.7, 139.0 (C-1′), 130.4 (C-2′), 130.4 (C-6′), 114.8 (C-3′), 114.8 (C-5′), 106.6 (C-1), 103.7 (C-3), 95.3 (C-5), 81.3 (Glc-5), 78.5 (Glc-3), 75.9 (Glc-1), 73.1 (Glc-2), 69.7 (Glc-4), 60.5 (Glc-6), 27.8 (-CH_2-). HRESIMS (m/z): $[M+H]^+$ calcd. for $C_{19}H_{22}FO_8$, 397.1299; found, 397.1289.

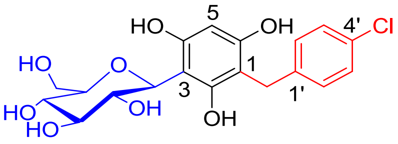

3-*C*-β-D-glucosyl-1-(4-chlorobenzyl)benzene-2,4,6-triol (49a) (novel) ^{1}H NMR

(DMSO-d_6, 600 MHz): δ = 9.12 (1H, s, -OH), 8.82 (1H, s, -OH), 8.00 (1H, s, -OH), 7.24 (2H, d, J = 8.5 Hz, H-3′, H-5′), 7.20 (2H, d, J = 8.5 Hz, H-2′, H-6′), 5.94 (1H, s, H-5), 4.95 (1H, s, Glc-OH), 4.95 (1H, d, J = 5.3 Hz, Glc-OH), 4.87 (1H, brs, Glc-OH), 4.78 (1H, d, J = 5.5 Hz, Glc-OH), 4.66 (1H, t, J = 5.0 Hz, Glc-OH), 4.61 (1H, d, J = 9.7 Hz, Glc-H_1), 3.71 (2H, s, -CH_2-), 3.62 (1H, m, Glc-H), 3.56 (1H, m, Glc-H), 3.20-3.39 (4H, m, Glc-H); ^{13}C NMR (DMSO-d_6, 150 MHz): δ = 155.9, 155.3, 154.7, 141.9 (C-1′), 130.6 (C-2′), 130.6 (C-6′), 130.0 (C-6′), 128.1 (C-3′), 128.1 (C-5′), 106.2 (C-1), 103.7 (C-3), 95.3 (C-5), 81.3 (Glc-5), 78.5 (Glc-3), 75.9 (Glc-1), 73.1 (Glc-2), 69.7 (Glc-4), 60.5 (Glc-6), 28.0 (-CH_2-). HRESIMS (m/z): $[M+H]^+$ calcd. for $C_{19}H_{22}ClO_8$, 413.1003; found, 413.0991.

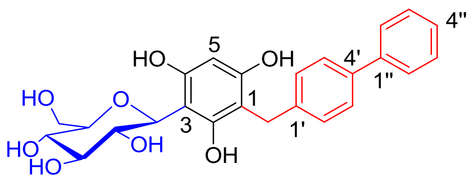

3-*C*-β-D-glucosyl-1-(biphenyl-4-ylmethyl)benzene-2,4,6-triol (50a) (novel) ^{1}H NMR (DMSO-d_6, 600 MHz): δ = 9.11 (1H, s, -OH), 8.81 (1H, s, -OH), 8.01 (1H, s, -OH), 7.59 (2H, d, J = 7.6 Hz, H-2″, H-6″), 7.48 (2H, d, J = 7.9 Hz, H-3′, H-5′), 7.42 (2H, t, J = 7.6 Hz, H-3″, H-5″), 7.31 (1H, overlapped, H-4″), 7.29 (2H, d, J = 7.9 Hz, H-2′, H-6′), 5.96 (1H, s, H-5), 4.96 (1H, brs, Glc-OH), 4.89 (1H, brs, Glc-OH), 4.80 (1H, brs, Glc-OH), 4.68 (1H, brs, Glc-OH), 4.63 (1H, d, J = 9.7 Hz, Glc-H_1), 3.78 (2H, s, -CH_2-), 3.56–3.64 (2H, m, Glc-H), 3.39 (2H, m, Glc-H), 3.22 (2H, m, Glc-H); ^{13}C NMR (DMSO-d_6, 150 MHz): δ = 156.0, 155.4, 154.6, 142.3, 140.8, 137.5, 129.4, 129.4, 129.3, 129.3 127.4, 126.9, 126.9, 126.6, 126.6, 106.6 (C-1), 103.7 (C-3), 95.3 (C-5), 81.4 (Glc-5), 78.6 (Glc-3), 75.9 (Glc-1), 73.1 (Glc-2), 69.7 (Glc-4), 60.5 (Glc-6), 28.3 (-CH_2-). HRESIMS (m/z): $[M+H]^+$ calcd. for $C_{25}H_{27}O_8$, 455.1706; found, 455.1691.

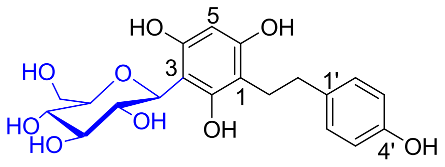

3-*C*-β-D-glucosyl-1-(4′-hydroxyphenethyl)benzene-2,4,6-triol (51a) ^{1}H NMR (Methanol-d_4, 600 MHz): δ = 7.03 (2H, dd, J = 2.0, 6.4 Hz, H-2′, H-6′), 6.66 (2H, dd, J = 2.0, 6.4 Hz, H-3′, H-5′), 5.94 (1H, s, H-5), 4.83 (1H, d, J = 7.4 Hz, Glc-H_1), 3.86

(1H, dd, J = 2.3, 12.5 Hz, Glc-H), 3.78 (1H, dd, J = 4.6, 12.5 Hz, Glc-H), 3.64 (1H, dd, J = 8.9 Hz, Glc-H), 3.48 (2H, m, Glc-H), 3.40 (1H, m, Glc-H), 2.71 (2H, m, -CH_2-), 2.59 (2H, m, -CH_2-); ^{13}C NMR (Methanol-d_4, 150 MHz): δ = 155.8, 154.9, 154.6, 153.8, 134.3 (C-1′), 128.8 (C-2′), 128.8 (C-6′), 114.7 (C-3′), 114.7 (C-5′), 108.5 (C-1), 102.8 (C-3), 94.8 (C-5), 81.1 (Glc-5), 78.2 (Glc-3), 76.3 (Glc-1), 73.3 (Glc-2), 69.7 (Glc-4), 60.7 (Glc-6), 34.5 (-CH_2-), 25.4 (-CH_2-). HRESIMS (m/z): $[M+H]^+$ calcd. for $C_{20}H_{25}O_9$, 409.1499; found, 409.1488.

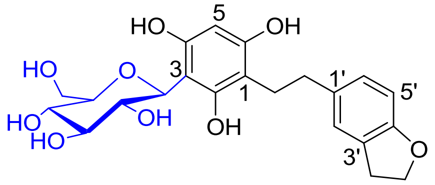

3-*C*-β-D-glucosyl-1-((3′,4′-dihydrobenzofuran-1′-yl)ethyl)benzene-2,4,6-triol (52a) (novel) HRESIMS: *m/z* 435.1635 $[M+H]^+$; ESI-MS *m/z* 433.37 $[M–H]^-$; ^{1}H NMR (Methanol-d_4, 600 MHz): δ = 7.07 (1H, s, H-2′), 6.92 (1H, d, J = 8.1 Hz, H-6′), 6.58 (1H, d, J = 8.1 Hz, H-5′), 5.94 (1H, s, H-5), 4.84 (1H, d, J = 9.5 Hz, Glc-H_1), 4.47 (2H, t, J = 8.6 Hz, -CH_2-), 3.86 (1H, dd, J = 2.2, 12.1 Hz, Glc-H), 3.78 (1H, dd, J = 4.7, 12.1 Hz, Glc-H), 3.64 (1H, dd, J = 9.7 Hz, Glc-H), 3.49 (2H, m, Glc-H), 3.40 (1H, m, Glc-H), 3.14 (2H, dd, J = 8.6 Hz, -CH_2-), 2.71 (2H, m, -CH_2-), 2.61 (2H, m, -CH_2-); ^{13}C NMR (Methanol-d_4, 150 MHz): δ = 157.8, 155.8, 154.9, 153.8, 135.4, 127.2, 126.6, 124.5, 108.5, 108.0, 102.8 (C-3), 94.8 (C-5), 81.0 (G-5), 78.2 (G-3), 76.3 (G-1), 73.3 (G-2), 70.6 (-CH_2-), 69.7 (G-4), 60.7 (G-6), 34.9 (-CH_2-), 29.3 (-CH_2-), 25.6 (-CH_2-). HRESIMS (m/z): $[M+H]^+$ calcd. for $C_{22}H_{27}O_9$, 435.1655; found, 435.1645.

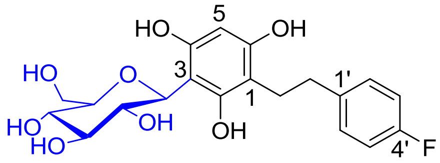

3-*C*-β-D-glucosyl-1-(4′-fluorophenethyl)benzene-2,4,6-triol (53a) (novel) ^{1}H NMR (DMSO-d_6, 600 MHz): δ = 8.99 (1H, s, -OH), 8.74 (1H, s, -OH), 7.96 (1H, s, -OH), 7.23 (2H, m, H-3′, H-5′), 7.20 (2H, dd, J = 8.9 Hz, H-2′, H-6′), 5.92 (1H, s, H-5), 4.62 (1H, d, J = 9.8 Hz, Glc-H_1), 3.64 (1H, d, J = 11.7 Hz, Glc-H), 3.58 (1H, dd, J = 11.7, 4.3 Hz, Glc-H), 3.42 (1H, t, J = 9.2 Hz, Glc-H), 3.31 (1H, t, J = 9.2 Hz, Glc-H),

3.20–3.25 (2H, m, Glc-H), 2.62–2.66 (4H, m, -CH_2-, -CH_2-); ^{13}C NMR (DMSO-d_6, 150 MHz): δ = 161.7 (C-4′), 155.9, 155.3, 154.3, 139.4 (C-1′), 130.2 (C-2′), 130.4 (C-6′), 115.2 (C-3′), 115.2 (C-5′), 107.0 (C-1), 103.7 (C-3), 95.3 (C-5), 81.4 (Glc-5), 78.6 (Glc-3), 75.9 (Glc-1), 73.1 (Glc-2), 69.7 (Glc-4), 60.6 (Glc-6), 34.9 (-CH_2-), 25.5 (-CH_2-). HRESIMS (m/z): $[M+H]^+$ calcd. for $C_{20}H_{24}FO_8$, 411.1455; found, 411.1447.

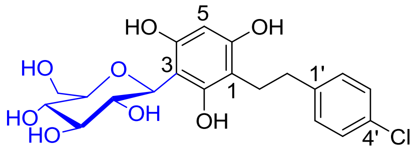

3-*C*-β-D-glucosyl-1-(4-chlorophenethyl)benzene-2,4,6-triol (54a) (novel) ^{1}H NMR (DMSO-d_6, 600 MHz): δ = 8.97 (1H, s, -OH), 8.72 (1H, s, -OH), 7.94 (1H, s, -OH), 7.29 (2H, d, J = 8.4 Hz, H-3′, H-5′), 7.20 (2H, d, J = 8.4 Hz, H-2′, H-6′), 5.89 (1H, s, H-5), 4.59 (1H, d, J = 9.8 Hz, Glc-H_1), 3.61 (1H, d, J = 11.0 Hz, Glc-H), 3.55 (1H, dd, J = 11.7, 4.1 Hz, Glc-H), 3.39 (1H, t, J = 9.4 Hz, Glc-H), 3.27 (1H, overlapped, Glc-H), 3.17–3.22 (2H, m, Glc-H), 2.61 (4H, m, -CH_2-, -CH_2-); ^{13}C NMR (DMSO-d_6, 150 MHz): δ = 155.9 (C-2), 155.3 (C-6), 154.3 (C-4), 142.3 (C-1′), 130.5 (C-4′), 130.4 (C-2′), 130.4 (C-6′), 128.5 (C-3′), 128.5 (C-5′), 106.9 (C-1), 103.7 (C-3), 95.3 (C-5), 81.4 (Glc-5), 78.6 (Glc-3), 75.9 (Glc-1), 73.1 (Glc-2), 69.7 (Glc-4), 60.6 (Glc-6), 35.0 (-CH_2-), 25.3 (-CH_2-). HRESIMS (m/z): $[M+H]^+$ calcd. for $C_{20}H_{24}ClO_8$, 427.1160; found, 427.1153.

Experimental Procedures of Anti-influenza A Virus Activity Assays[1, 2]

Cells and viruses

293T-Gluc cells were generated by transfection of plasmid DNA pLenti6-Gluc constitutively expressing the negative-strand RNA of *Gaussia luciferase* (Gluc) gene, which is converted into the positive strand upon IAV infection, and expresses the Gluc enzyme. The (Gluc) gene was amplified with forward primer (5′–TATGAATTCGGAAAAACGCCAGCAAC-3′) and reverse primer (5′–ATAAGGGCCCAAAATCTTCTTTCATCCGC-3′). PCR products were cloned into pLenti6/V5-DEST vector (Invitrogen) generating pLenti6-Gluc. 293T cells were transfected with pLenti6-Gluc using lipofecta-mine2000 in accordance with the manufacturer's protocol, and then were selected with 10 μg/mL Blasticidin 24 h post-transfection. Under antibiotic selective pressure, several clonal colonies were

obtained and tested for luciferase expression. One clonal cell line demonstrated high-level expression of luciferase; this cell line was named 293T-Gluc and was used for subsequent experiments. MDCK and 293T cells were maintained in Dulbecco′s modified Eagle′s medium with 10% (v/v) fetal bovine serum (FBS; Invitrogen). The cells were cultured at 37 ℃ in a humidified atmosphere of 5% CO_2. Influenza A/WSN/33 (H1N1) was rescued from eight plasmids using cocultured MDCK and 293T cells.

Gluc reporter assay

293T-Gluc cells were cultured to 90% confluence, released with 0.25% trypsin EDTA and resuspended in DMEM with 10% FBS at 7×10^5 cells/mL. Cells were then seeded in the wells of 96-well plate at 100 μL/well. After an overnight incubation at 37 ℃ and 5% CO_2, cells were infected with the indicated influenza viruses, which were contained in 10 μL serum-free DMEM culture medium. For evaluation assay of antivirals and high-throughput screening, 1 μL of each tested compound (final concentration 100 μ M and 20 μM) was added to cells and incubated for 2 h prior to infection, after which cells were infected with influenza A/WSN/33 virus at an MOI of 0.3. After a further incubation for 24 h at 37 ℃, the cell supernatant was collected and measured for Gluc activity and the inhibition rate of the tested compounds was calculated. In each 96-well plate ribavirin and DMSO were used as positive and negative controls, respectively.

Cell viability assay

Cell viability was evaluated by Cell Counting Kit-8 (CCK-8) assay. Briefly, 293T cells were cultured to 80% confluence in a 96-well plate with 1×10^4 cells/well and incubated with compounds. Six concentrations of each compound, ranging from 12.5 μM to 200 μM, were used to treat cells for 48 h. Cells cultured in DMSO only were used as the control. After a 48 h incubation, 10 μL CCK-8 solution was added to each well and incubated for an additional 1–2 h at 37 ℃. The absorbance at 450 nm was subsequently measured using EnSpire 2300 Multilable Reader (PerkinElmer).

Supplementary Table 1 The characteristic MS/MS fragment ions of *C*-glycosylated products generated by AbCGT with different sugar donors

Sugar donors	MS/MS fragment ions of *C*-glycosylated products
UDP-Glc	$[M–H–120]^-$ and $[M–H–90]^-$
UDP-Gal	$[M–H–120]^-$ and $[M–H–90]^-$
UDP-Xyl	$[M–H–90]^-$ and $[M–H–60]^-$
UDP-GlcA	$[M–H–134]^-$ and $[M–H–104]^-$

Supplementary Table 2. SGLT2 inhitory activity of phloretin-3′-*C*-glycosides with different sugar moieties

Glucosylated Products	Inhibitory Rates (%) [a]
17a	52.2
17aa	2.8
17b	–3.1
17c	1.7
Positive control	100

a. The inhibitory rates were obtained with *C*-glycosides under the final concentration of 10 μM and with dapagliflozin (positive control) under the final concentration of 10 μM.

Supplementary Table 3. Anomeric coupling constants of glucosylated products

Glucosylated Products	Anomeric Coupling Constants (Hz)
9a	9.8
11a	9.8
11aa	9.8, 9.8
16aa	10.0, 10.0
17a	9.8
17aa	9.5, 9.5
17b	9.7
17c	9.8
20a	9.7
21a	9.8
28a	9.9
30a	9.8
38a	9.7
39a	9.7
42a	9.8
43a	9.8
44a	9.7
46a	9.7
47a	9.7
47aa	9.8, 9.8
48a	9.6
49a	9.7
50a	9.7
51a	7.4
52a	9.5
53a	9.8
54a	9.4

Supplementary Table 4. Anti-influenza A virus activity of aglycon **44** and *C*-glycoside **44a**

Glucosylated Products	Inhibitory Rates (%) [a]	IC_{50} (M)	CC_{50} (M)
44	—	—	—
44a	82.8	9.78×10^{-6}	8.86×10^{-5}
Ribavirin	85.6	\	\

a. The inhibitory rates were obtained with *C*-glycosides under the final concentration of 20 μM and with ribavirin (positive control) under the final concentration of 30 μM.

—: represented no inhibition activity was determined. \: represented not detected.

Supplementary Table 5. PCR primers used in this study

Primers	Sequences (5′ to 3′)
AbCGT-F	ATGTCAAACTCCGGCAGGCCTCACGTAGCCCT
AbCGT-R	TTAGCTGCCCATCATTATTCTACTAATGAACT
AbCGT-F$_{pET28a}$	GTGCCGCGCGGCAGCCATATGTCAAACTCCGGCA
AbCGT-R$_{pET28a}$	GCGGCCGCAAGCTTGTCGTTAGCTGCCCATCATTAT
AbCGT-V183A-F	AGCCGCGTGGGCCCCGCCGCCGCTCCACGTCCCC
AbCGT-V183A-R	GCGGCGGCGGGGCCCACGCGGCTGGCACGGGGTG
AbCGT-V183C-F	AGCCGCGTGGTGCCCGCCGCCGCTCCACGTCCCC
AbCGT-V183C-R	GCGGCGGCGGGCACCACGCGGCTGGCACGGGGTG
AbCGT-V183D-F	AGCCGCGTGGGACCCGCCGCCGCTCCACGTCCCC
AbCGT-V183D-R	GCGGCGGCGGGTCCCACGCGGCTGGCACGGGGTG
AbCGT-V183E-F	AGCCGCGTGGGAGCCGCCGCCGCTCCACGTCCCC
AbCGT-V183E-R	GCGGCGGCGGCTCCCACGCGGCTGGCACGGGGTG
AbCGT-V183F-F	AGCCGCGTGGTTCCCGCCGCCGCTCCACGTCCCC
AbCGT-V183F-R	GCGGCGGCGGGAACCACGCGGCTGGCACGGGGTG
AbCGT-V183G-F	AGCCGCGTGGGGCCCGCCGCCGCTCCACGTCCCC
AbCGT-V183G-R	GCGGCGGCGGGCCCCACGCGGCTGGCACGGGGTG
AbCGT-V183H-F	AGCCGCGTGGCACCCGCCGCCGCTCCACGTCCCC
AbCGT-V183H-R	GCGGCGGCGGGTGCCACGCGGCTGGCACGGGGTG
AbCGT-V183I-F	AGCCGCGTGGATCCCGCCGCCGCTCCACGTCCCC
AbCGT-V183I-R	GCGGCGGCGGGATCCACGCGGCTGGCACGGGGTG
AbCGT-V183K-F	AGCCGCGTGGAAGCCGCCGCCGCTCCACGTCCCC
AbCGT-V183K-R	GCGGCGGCGGCTTCCACGCGGCTGGCACGGGGTG
AbCGT-V183L-F	AGCCGCGTGGCTCCCGCCGCCGCTCCACGTCCCC
AbCGT-V183L-R	GCGGCGGCGGGAGCCACGCGGCTGGCACGGGGTG
AbCGT-V183M-F	AGCCGCGTGGATGCCGCCGCCGCTCCACGTCCCC
AbCGT-V183M-R	GCGGCGGCGGCATCCACGCGGCTGGCACGGGGTG
AbCGT-V183N-F	AGCCGCGTGGAACCCGCCGCCGCTCCACGTCCCC
AbCGT-V183N-R	GCGGCGGCGGGTTCCACGCGGCTGGCACGGGGTG
AbCGT-V183P-F	AGCCGCGTGGCCCCCGCCGCCGCTCCACGTCCCC
AbCGT-V183P-R	GCGGCGGCGGGGGGCCACGCGGCTGGCACGGGGTG
AbCGT-V183Q-F	AGCCGCGTGGCAGCCGCCGCCGCTCCACGTCCCC
AbCGT-V183Q-R	GCGGCGGCGGCTGCCACGCGGCTGGCACGGGGTG
AbCGT-V183R-F	AGCCGCGTGGCGCCCGCCGCCGCTCCACGTCCCC
AbCGT-V183R-R	GCGGCGGCGGGCGCCACGCGGCTGGCACGGGGTG
AbCGT-V183S-F	AGCCGCGTGGTCCCCGCCGCCGCTCCACGTCCCC
AbCGT-V183S-R	GCGGCGGCGGGGACCACGCGGCTGGCACGGGGTG
AbCGT-V183T-F	AGCCGCGTGGACCCCGCCGCCGCTCCACGTCCCC
AbCGT-V183T-R	GCGGCGGCGGGGTCCACGCGGCTGGCACGGGGTG
AbCGT-V183W-F	AGCCGCGTGGTGGCCGCCGCCGCTCCACGTCCCC
AbCGT-V183W-R	GCGGCGGCGGCCACCACGCGGCTGGCACGGGGTG
AbCGT-V183Y-F	AGCCGCGTGGTACCCGCCGCCGCTCCACGTCCCC
AbCGT-V183Y-R	GCGGCGGCGGGTACCACGCGGCTGGCACGGGGTG

Supplementary Table 6. HPLC methods used in this study

Method	Solvent A	Solvent B	Gradient	Analysis (Substrates)
1	0.1% formic acid aqueous solution	Methanol	15–100% B, 30 min; 100% B, 5 min	1–8, 10–15, 17, 18, 20, 23–26, 30, 42–44, 46, 49, 51
2	0.1% formic acid aqueous solution	Methanol	10–100% B, 30 min; 100% B, 5 min	9, 16
3	0.1% formic acid aqueous solution	Methanol	5–100% B, 30 min; 100% B, 5 min	19
4	0.1% formic acid aqueous solution	Methanol	30–100% B, 30 min; 100% B, 5 min	21, 22, 27–29, 31, 32, 34–41, 47, 48, 50, 52–54
5	0.1% formic acid aqueous solution	Methanol	60–100% B, 30 min; 100% B, 5 min	33
6	0.1% formic acid aqueous solution	Methanol	55–100% B, 30 min; 100% B, 5 min	45

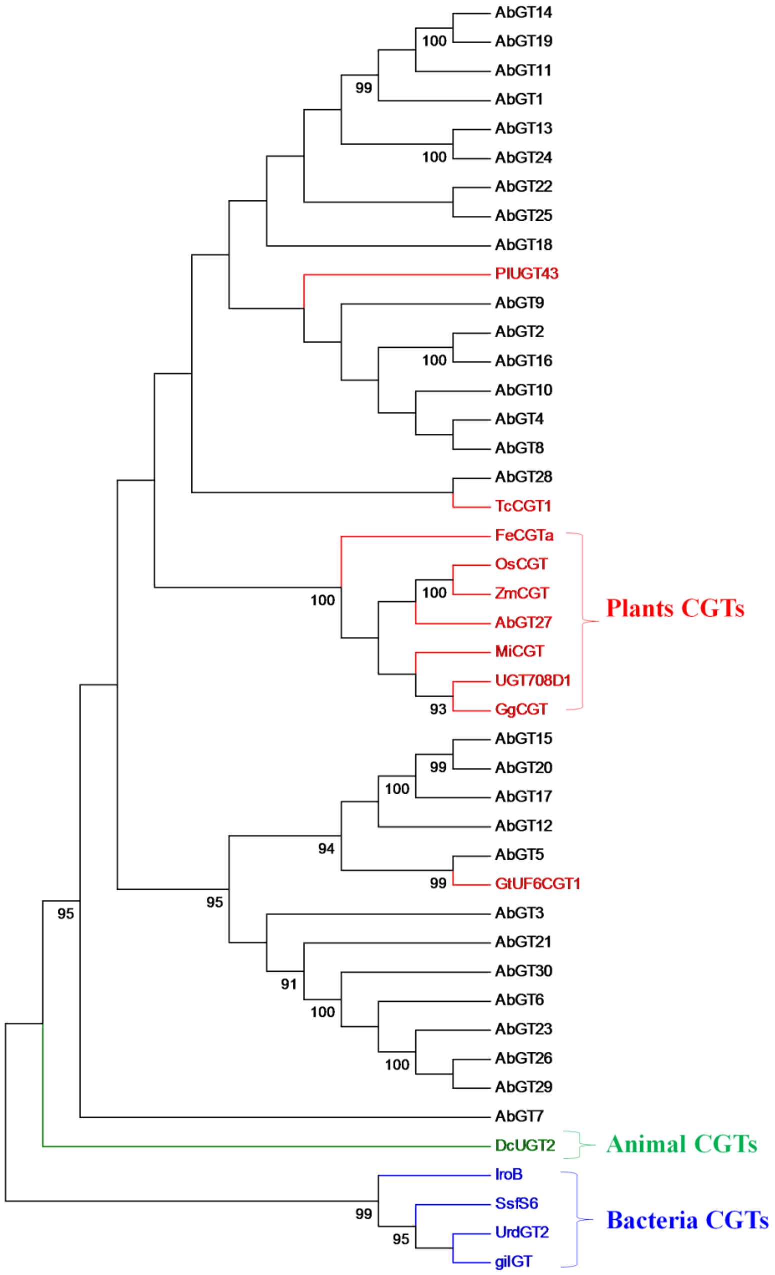

Supplementary Figure 1. Phylogenetic analyses of AbGTs with CGTs of plants, animals and bacteria. The phylogenetic tree was constructed by the neighbor-joining method using Clustal W (MEGA 5.0). The lengths of lines indicate the relative distance between nodes. Plant CGTs (red): OsCGT (Genbank accession No. FM179712), ZmCGT (NP_001132650), AbGT27 (AbCGT, in this work), UGT708D1 (BAR73279), MiCGT (KT200208), FeCGTa (AB909375), GtUF6CGT1 (A0A0B6VIJ5), PIUGT43 (KU317801), TcCGT1 (MK644229) and GgCGT (QGL05036.1). Animal CGTs (green): DcUGT2 (ATL15304.1). Bacteria CGTs (blue): gilGT (AAP69578.2), UrdGT2 (AAF00209), IroB (CAE55724) and SsfS6 (ADE34512).

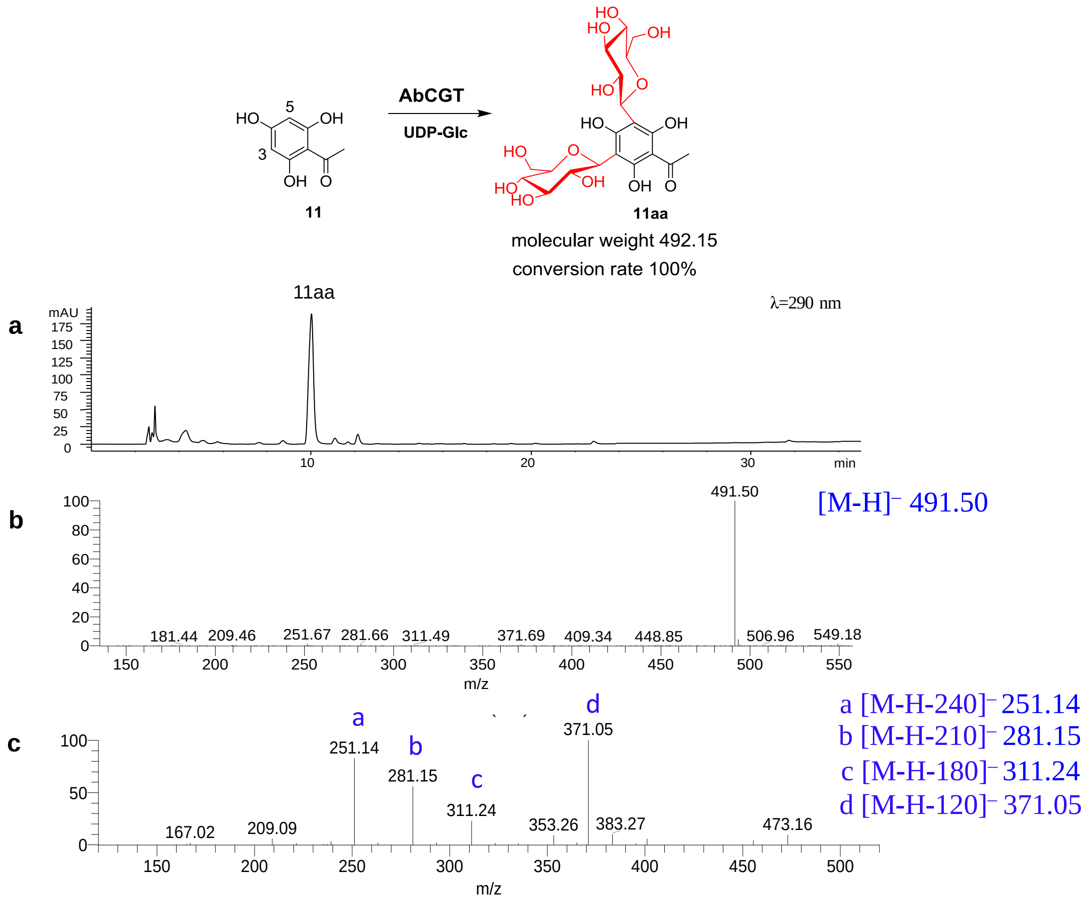

Supplementary Figure 2. HPLC-UV/ESI-MS analysis of AbCGT enzyme product using phloracetophene (**11**) and UDPG as substrates. a) HPLC-UV analysis of the AbCGT catalyzing reaction; b) Typical negative ion MS for the peak of **11aa**; c) Typical negative MS^2 for peak of **11aa**.

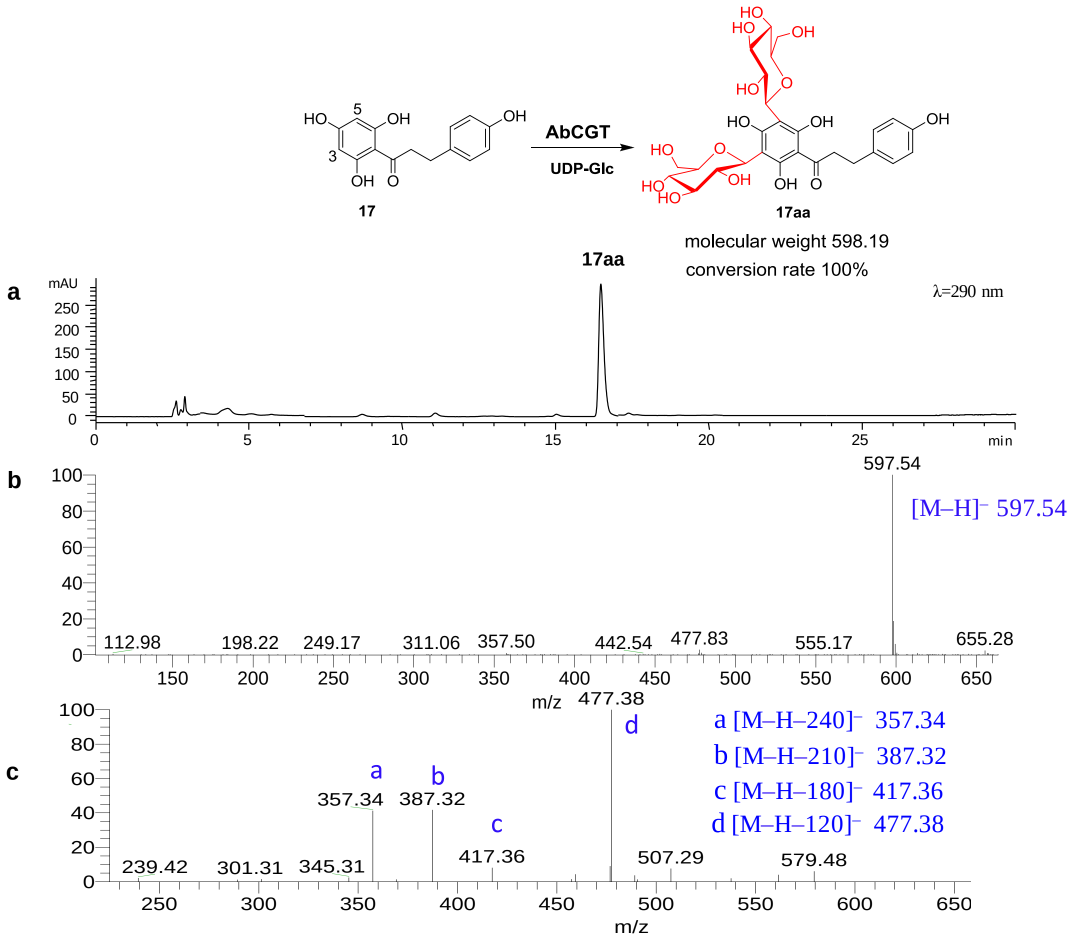

Supplementary Figure 3. HPLC-UV/ESI-MS analysis of AbCGT enzyme product using phloretin (**17**) and UDPG as substrates. a) HPLC-UV analysis of the AbCGT catalyzing reaction; b) Typical negative ion MS for the peak of **17aa**; c) Typical negative MS^2 for peak of **17aa**.

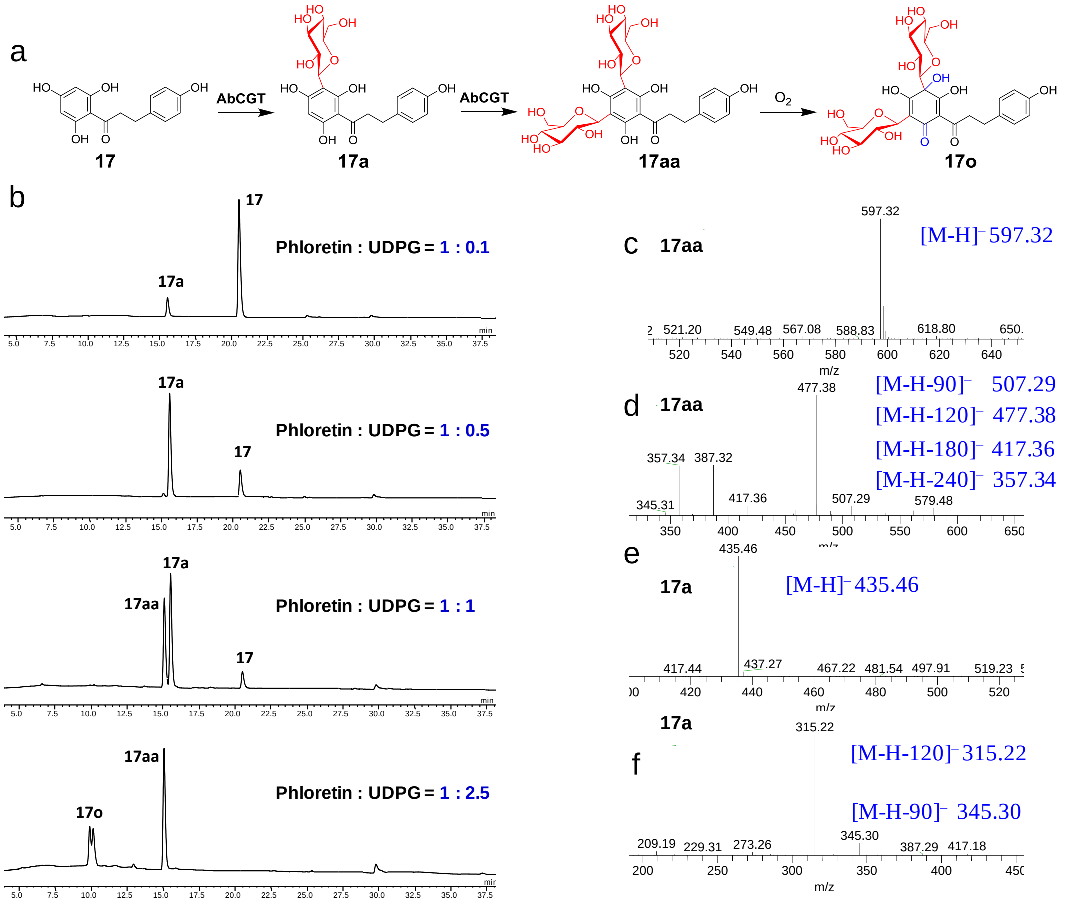

Supplementary Figure 4. HPLC-UV/ESI-MS analysis of the AbCGT enzyme products with different ratios of acceptor (**17**) and sugar donor (UDPG). a) The reactions catalyzed by AbCGT using phloretin (**17**) as the acceptor and excess UDPG as the sugar donor; b) HPLC-UV analysis of the AbCGT catalyzing reactions with the ratios of phloretin and UDPG from 1 : 0.1 to 1 : 2.5; c) Typical negative ion MS for the peak of **17aa**; d) Typical negative MS^2 for peak of **17aa**; e) Typical negative ion MS for the peak of **17a**; f) Typical negative MS^2 for peak of **17a**.

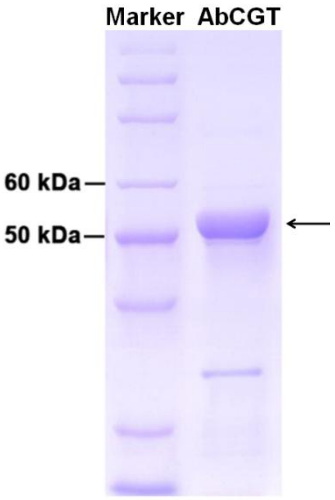

Supplementary Figure 5. SDS-PAGE analysis of purified recombinant His$_6$-AbCGT (AbGT27). This experiment was repeated independently three times with similar results.

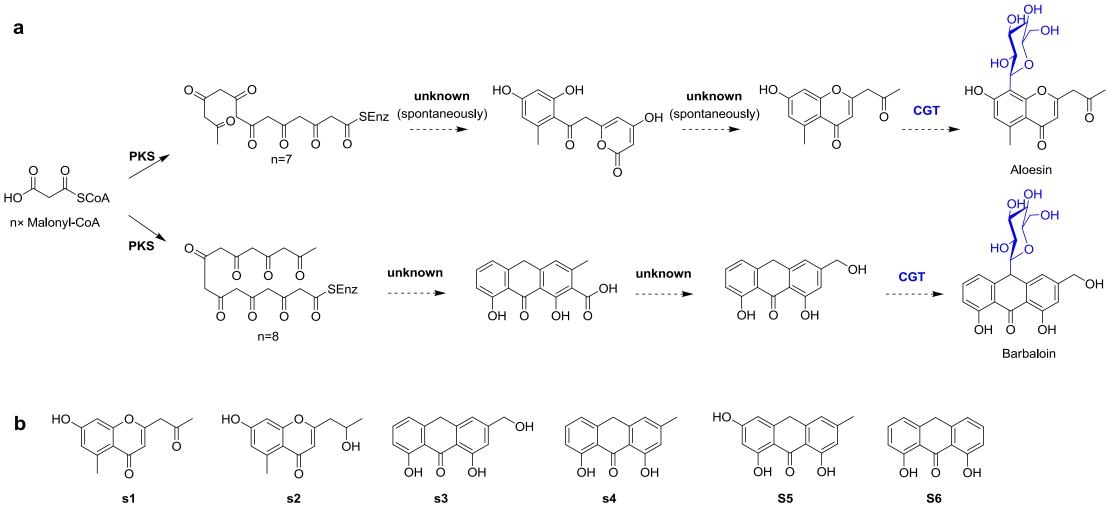

Supplementary Figure 6. Exploring the native function of AbCGT. a) Proposed biosynthesis pathway of aloesin and barbaloin. b) Substrates used in the reactions catalyzed by AbCGT. (AbCGT was unable to catalyze the *C*-glycosylation of substrates **s1**–**s6**)

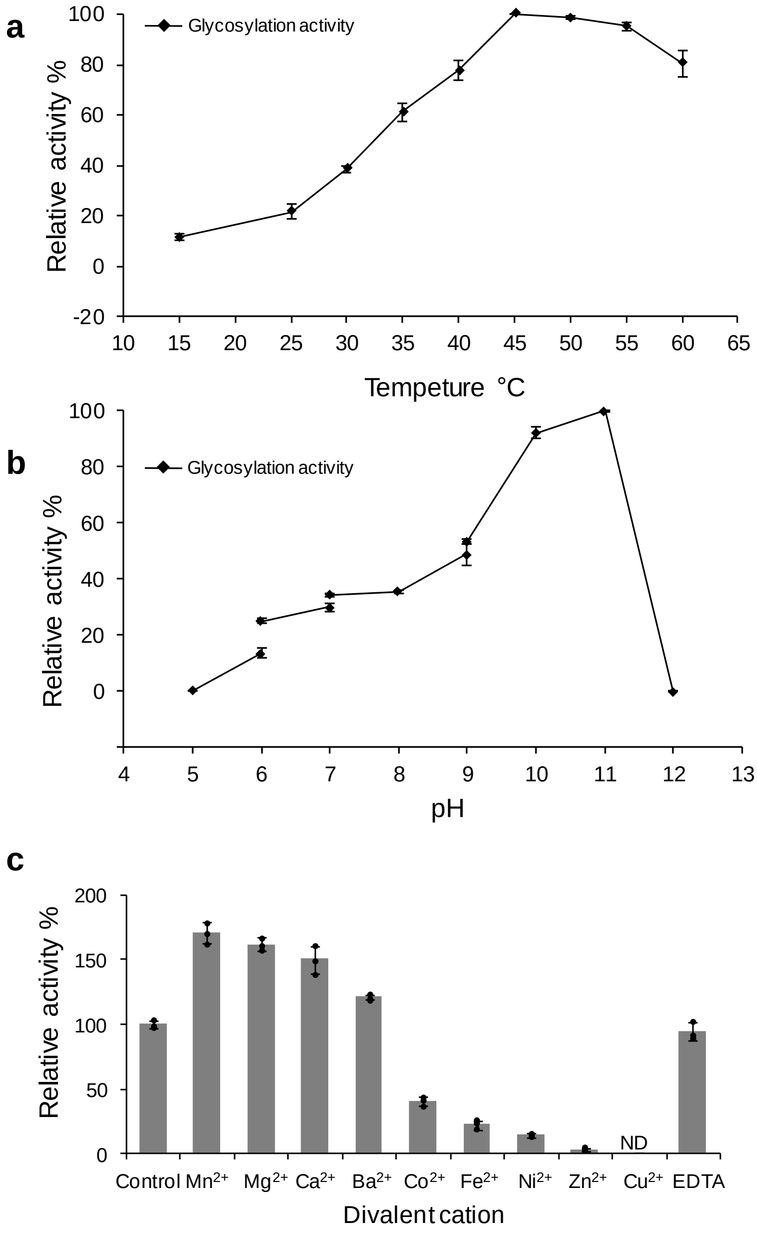

Supplementary Figure 7. Biochemical properties of recombinant AbCGT. a) Effects of temperature on the enzymatic glycosylation activity of AbCGT. b) pH dependency of AbCGT. c) Effects of various divalent metal ions on AbCGT activities. Phloretin (**17**) and UDPG were used as an acceptor and a sugar donor, respectively. Enzyme activity values represent mean ± SD of three independent replicates (n = 3). ND: not detected.

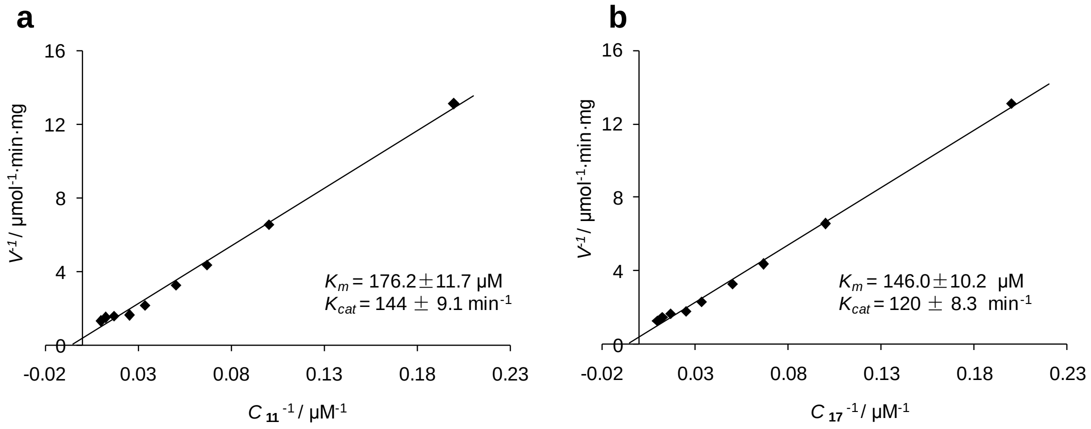

Supplementary Figure 8. Determination of kinetic parameters for AbCGT. The apparent K_m values were detected with phloracetophene (**11**) (a) and phloretin (**17**) (b) as acceptors, respectively and UDP-Glc as a donor. K_m and k_{cat} values represent mean ±standard deviation (s.d.) of three independent replicates.

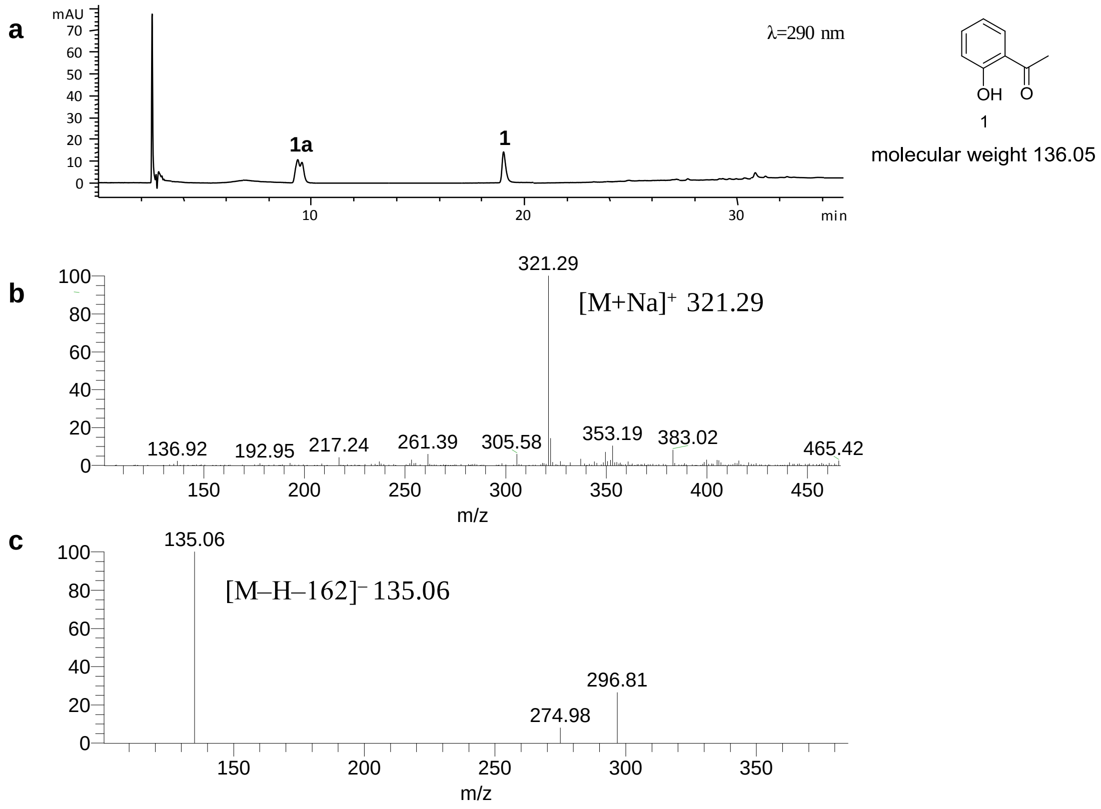

Supplementary Figure 9. HPLC-UV/ESI-MS analysis of AbCGT enzyme product using aglycon **1** and UDPG as substrates. a) HPLC-UV analysis of the AbCGT catalyzing reaction; b) Typical positive ion MS for the peak of **1a**; c) Typical negative MS^2 for peak of **1a**.

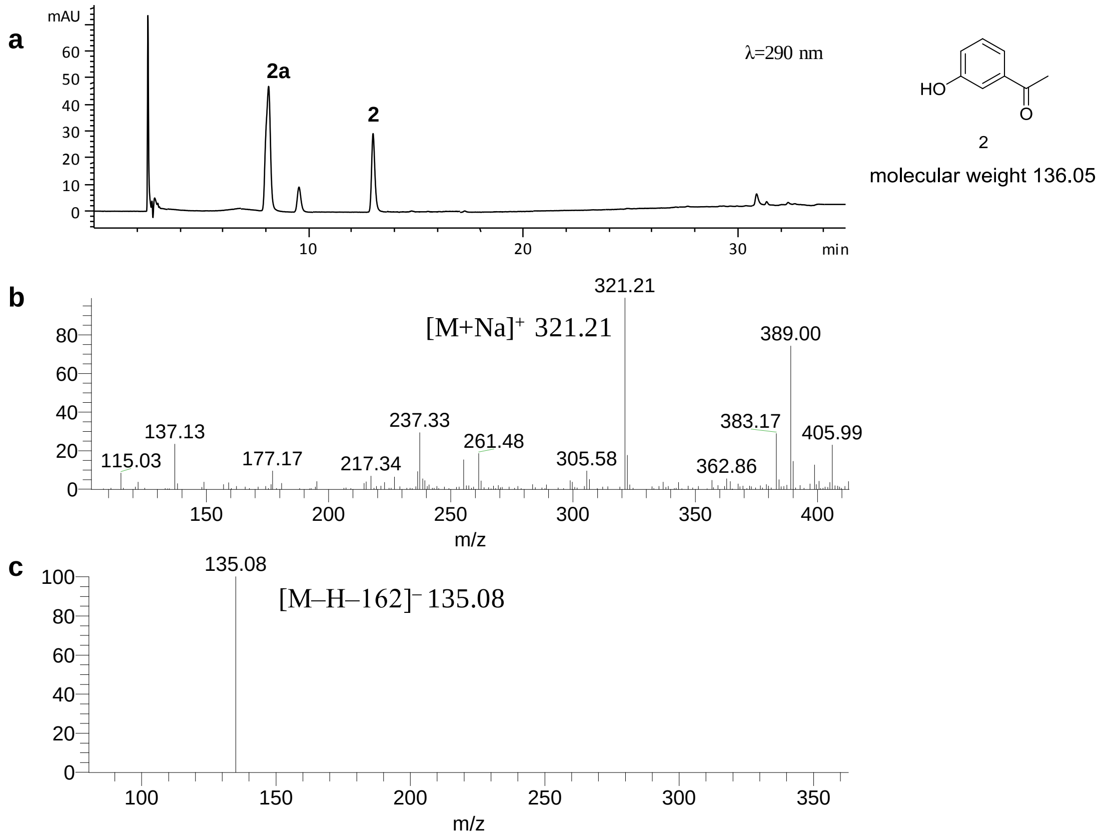

Supplementary Figure 10. HPLC-UV/ESI-MS analysis of AbCGT enzyme product using aglycon **2** and UDPG as substrates. a) HPLC-UV analysis of the AbCGT catalyzing reaction; b) Typical positive ion MS for the peak of **2a**; c) Typical negative MS^2 for peak of **2a**.

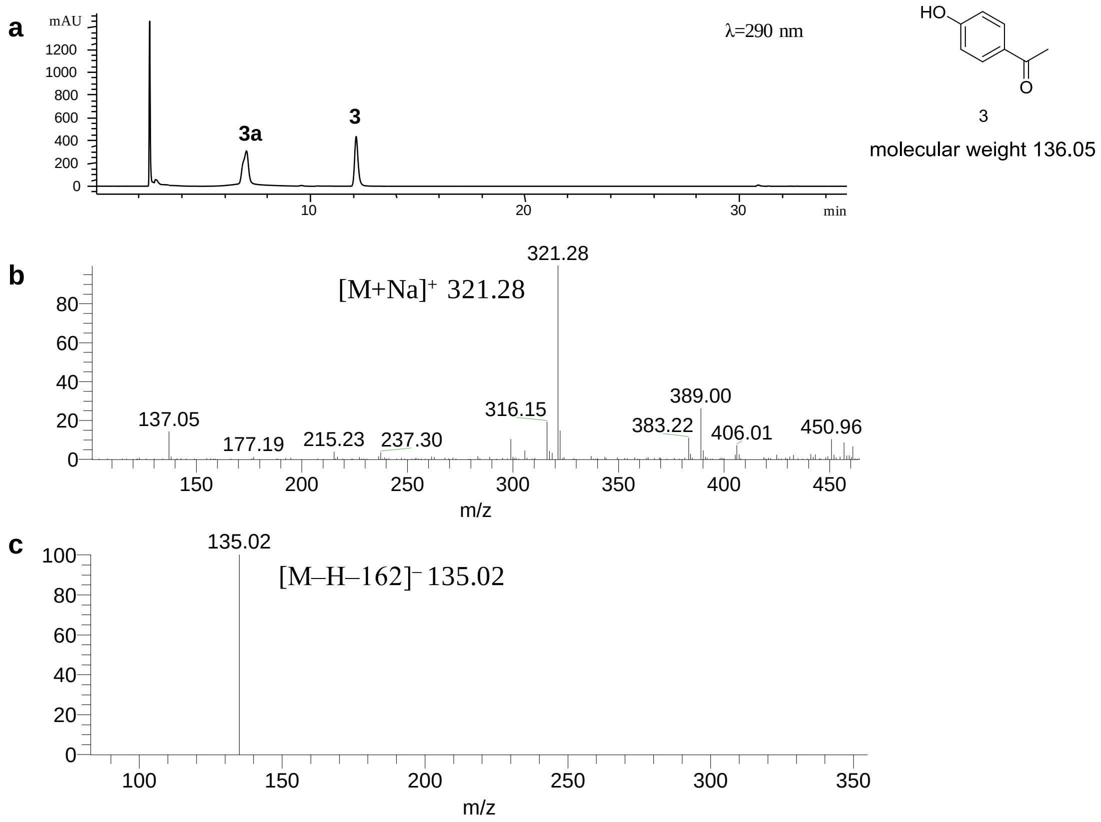

Supplementary Figure 11. HPLC-UV/ESI-MS analysis of AbCGT enzyme product using aglycon **3** and UDPG as substrates. a) HPLC-UV analysis of the AbCGT catalyzing reaction; b) Typical positive ion MS for the peak of **3a**; c) Typical negative MS^2 for peak of **3a**.

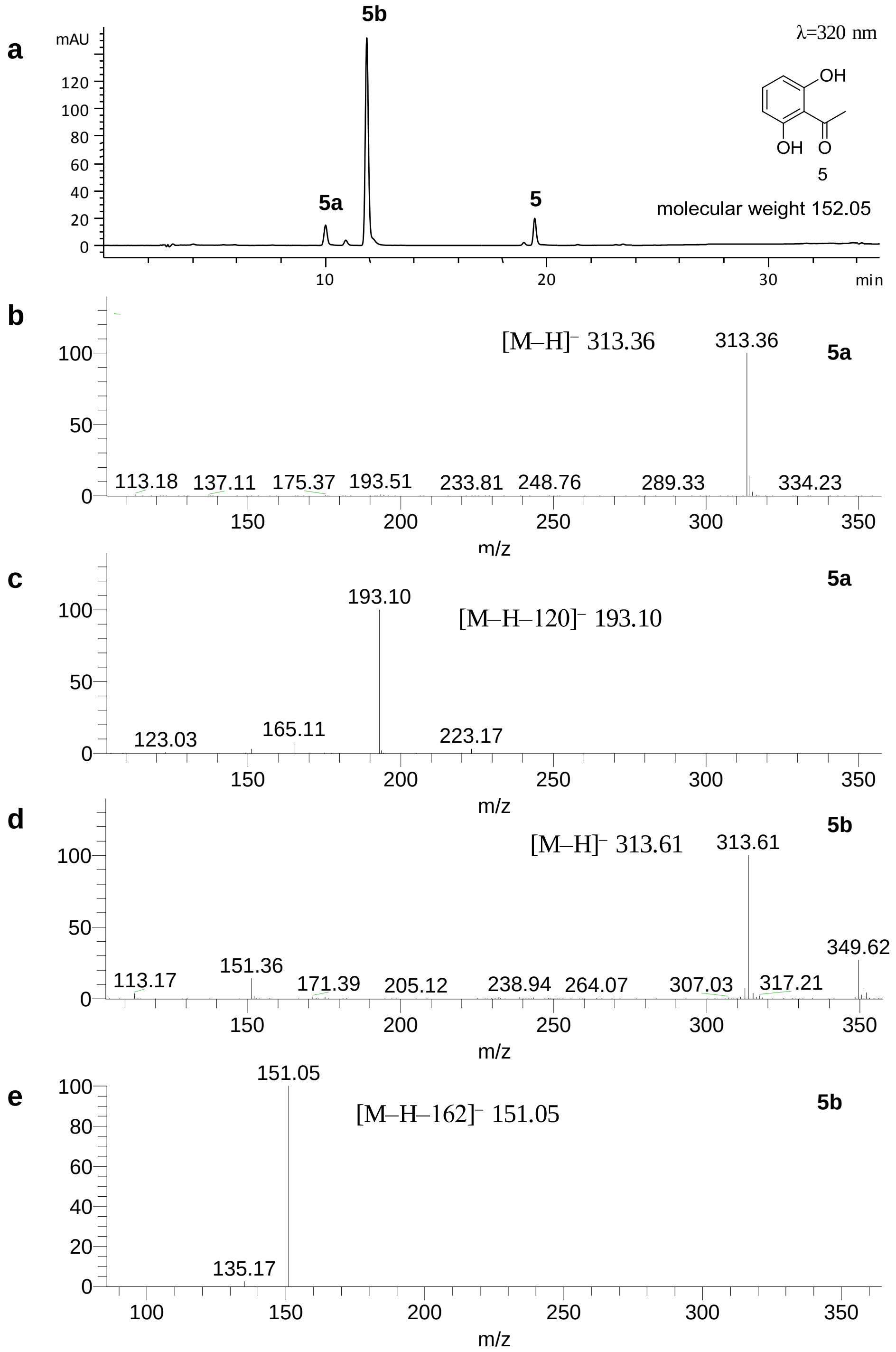

Supplementary Figure 12. HPLC-UV/ESI-MS analysis of AbCGT enzyme products using aglycon **5** and UDPG as substrates. a) HPLC-UV analysis of the AbCGT catalyzing reaction; b) Typical negative ion MS for the peak of **5a**; c) Typical negative MS^2 for peak of **5a**; d) Typical negative ion MS for the peak of **5b**; e) Typical negative MS^2 for peak of **5b**.

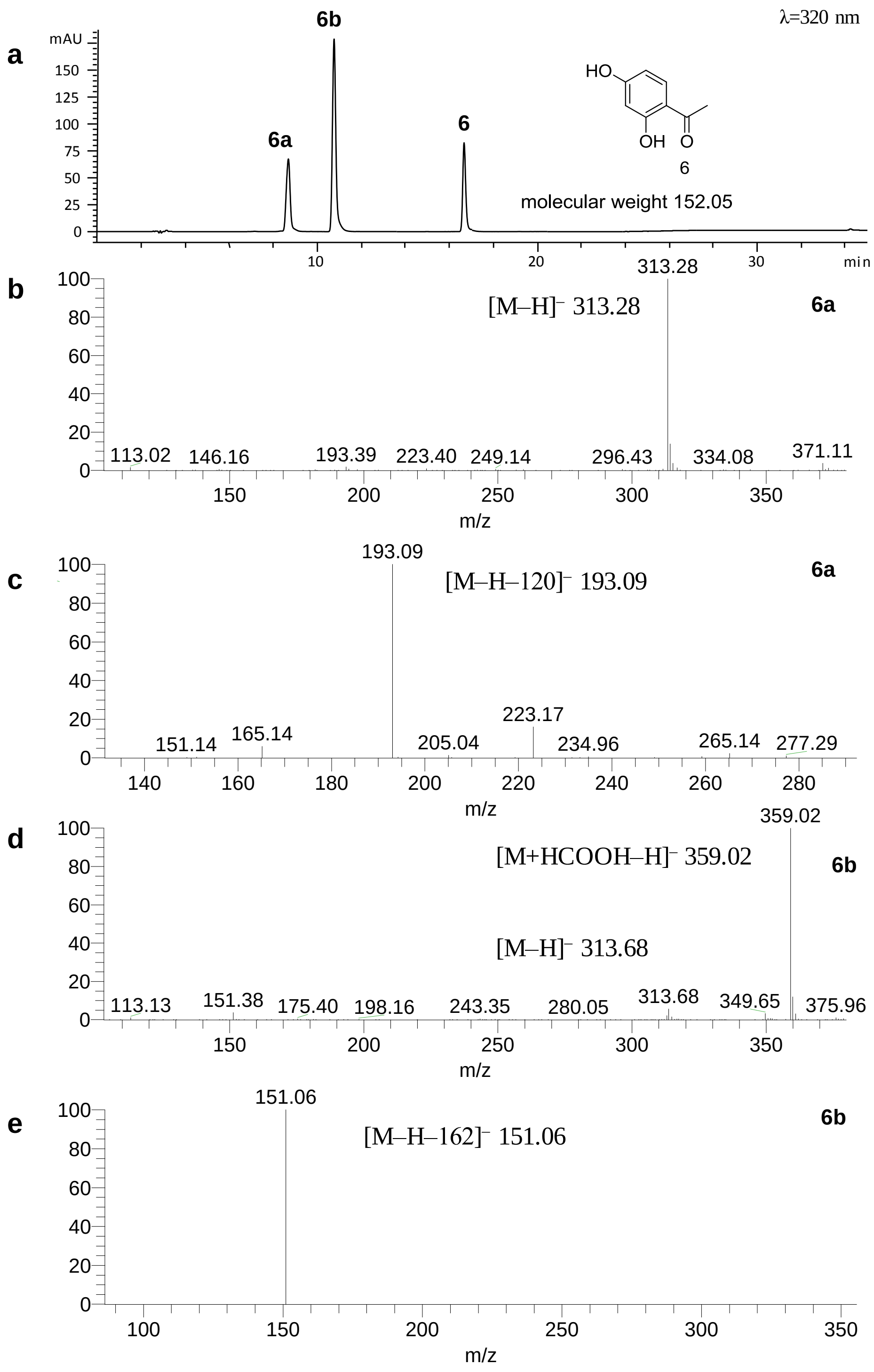

Supplementary Figure 13. HPLC-UV/ESI-MS analysis of AbCGT enzyme products using aglycon **6** and UDPG as substrates. a) HPLC-UV analysis of the AbCGT catalyzing reaction; b) Typical negative ion MS for the peak of **6a**; c) Typical negative MS^2 for peak of **6a**; d) Typical negative ion MS for the peak of **6b**; e) Typical negative MS^2 for peak of **6b**.

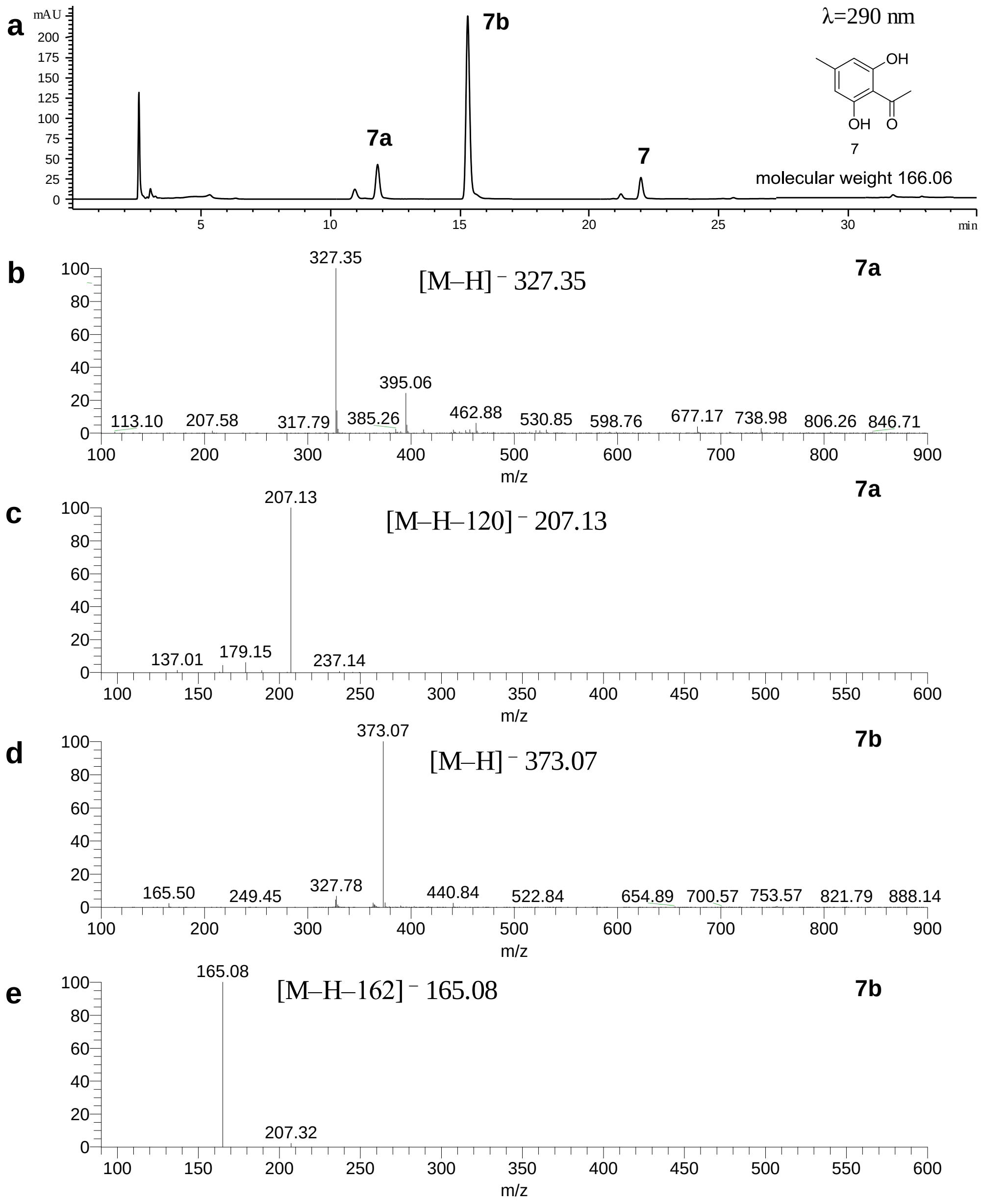

Supplementary Figure 14. HPLC-UV/ESI-MS analysis of AbCGT enzyme products using aglycon **7** and UDPG as substrates. a) HPLC-UV analysis of the AbCGT catalyzing reaction; b) Typical negative ion MS for the peak of **7a**; c) Typical negative MS^2 for peak of **7a**; d) Typical negative ion MS for the peak of **7b**; e) Typical negative MS^2 for peak of **7b**.

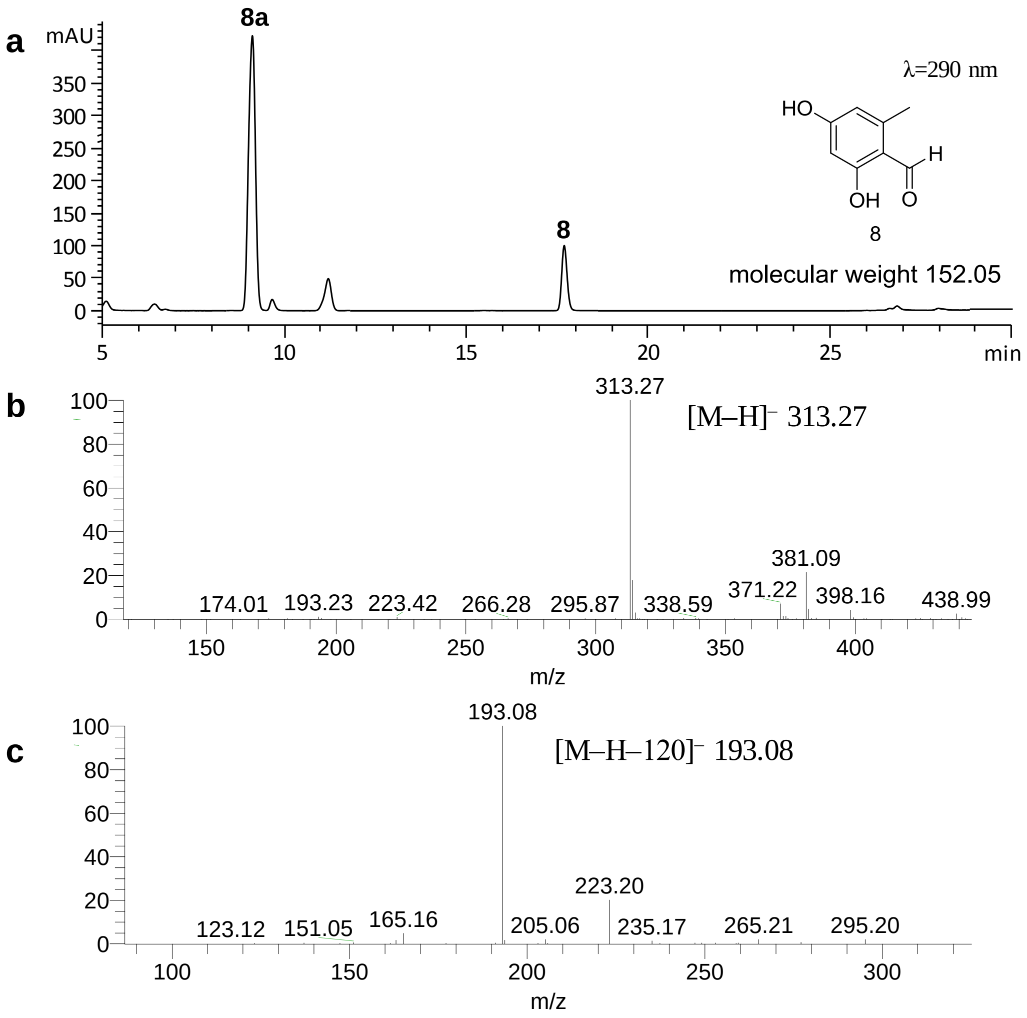

Supplementary Figure 15. HPLC-UV/ESI-MS analysis of AbCGT enzyme product using aglycon **8** and UDPG as substrates. a) HPLC-UV analysis of the AbCGT catalyzing reaction; b) Typical negative ion MS for the peak of **8a**; c) Typical negative MS^2 for peak of **8a**.

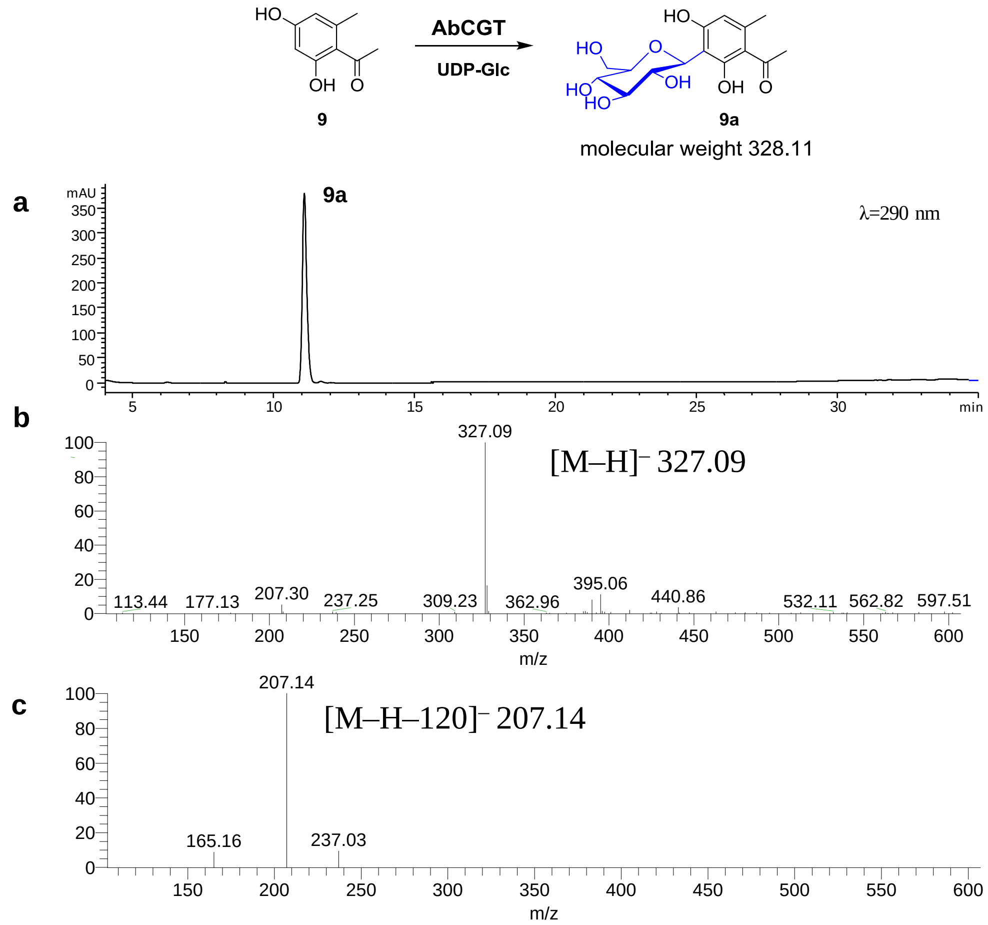

Supplementary Figure 16. HPLC-UV/ESI-MS analysis of AbCGT enzyme product using aglycon **9** and UDPG as substrates. a) HPLC-UV analysis of the AbCGT catalyzing reaction; b) Typical negative ion MS for the peak of **9a**; c) Typical negative MS^2 for peak of **9a**.

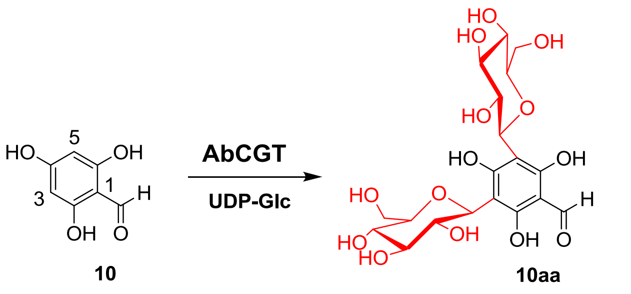

molecular weight 478.13

conversion rate 100%

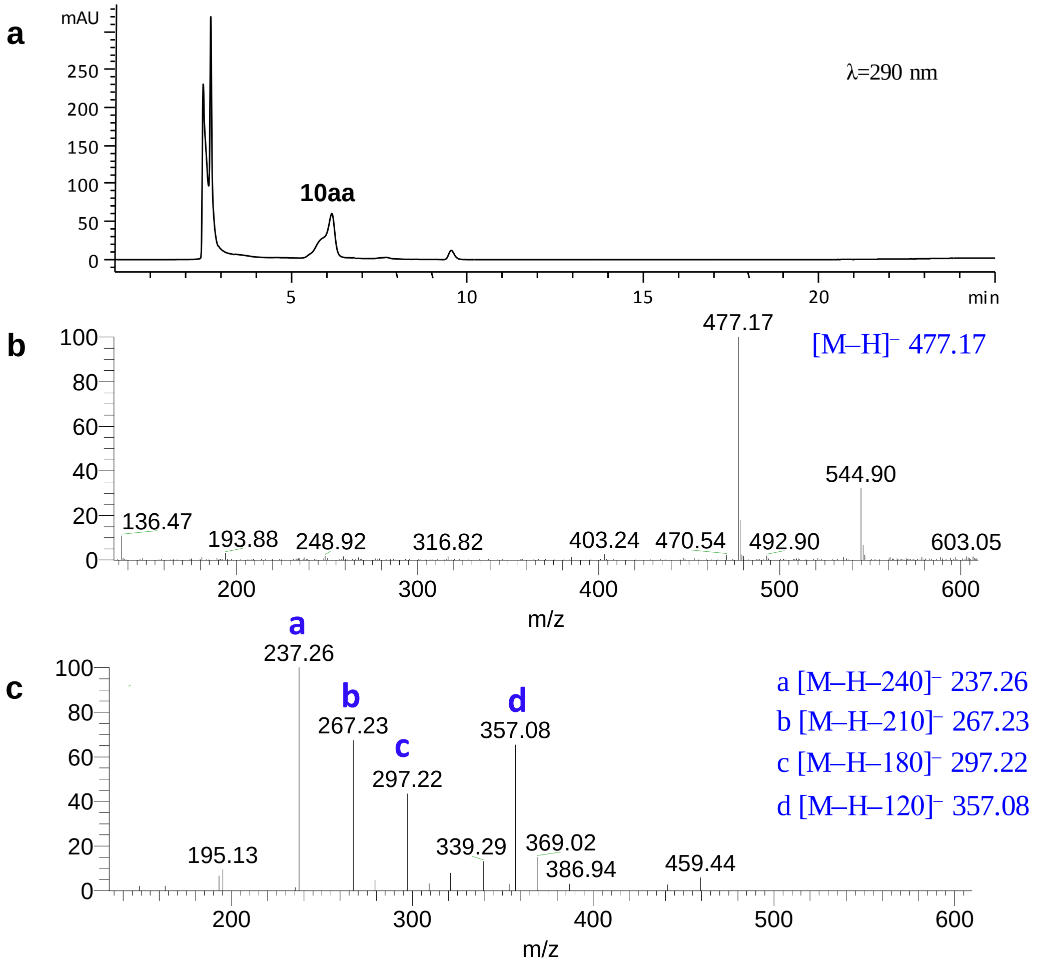

Supplementary Figure 17. HPLC-UV/ESI-MS analysis of AbCGT enzyme product using aglycon **10** and UDPG as substrates. a) HPLC-UV analysis of the AbCGT catalyzing reaction; b) Typical negative ion MS for the peak of **10aa**; c) Typical negative MS^2 for peak of **10aa**.

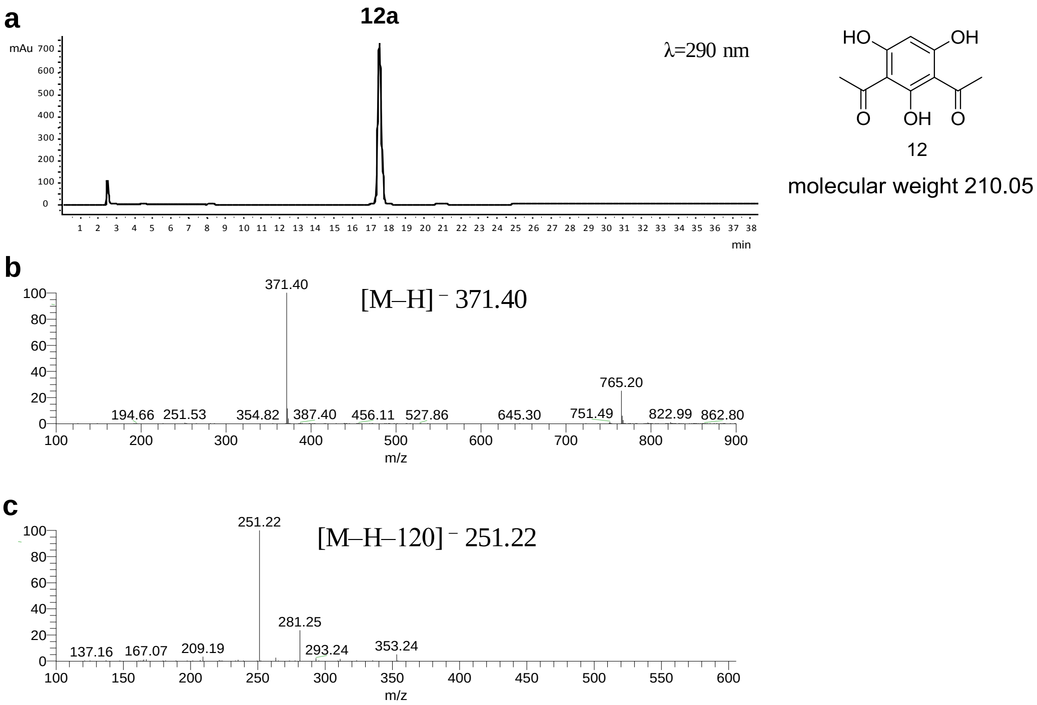

Supplementary Figure 18. HPLC-UV/ESI-MS analysis of AbCGT enzyme product using aglycon **12** and UDPG as substrates. a) HPLC-UV analysis of the AbCGT catalyzing reaction; b) Typical negative ion MS for the peak of **12a**; c) Typical negative MS^2 for peak of **12a**.

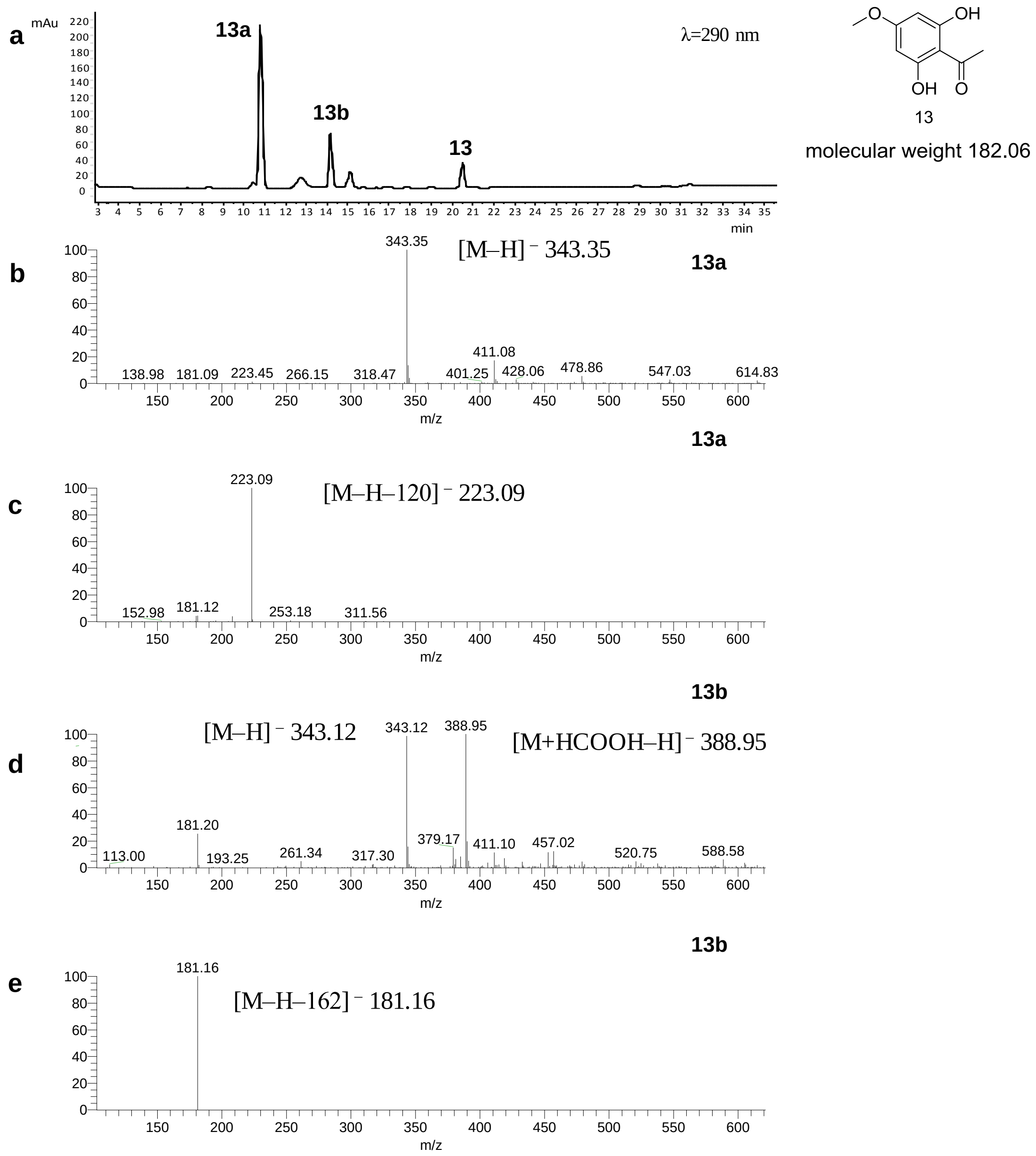

Supplementary Figure 19. HPLC-UV/ESI-MS analysis of AbCGT enzyme product using aglycon **13** and UDPG as substrates. a) HPLC-UV analysis of the AbCGT catalyzing reaction; b) Typical negative ion MS for the peak of **13a**; c) Typical negative MS^2 for peak of **13a**; d) Typical negative ion MS for the peak of **13b**; e) Typical negative MS^2 for peak of **13b**.

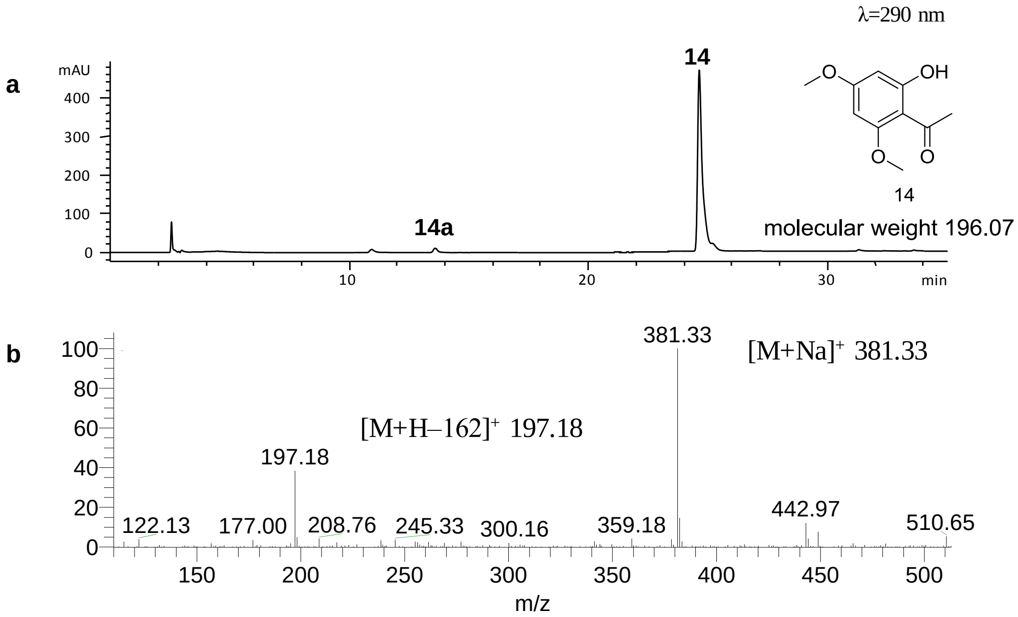

Supplementary Figure 20. HPLC-UV/ESI-MS analysis of AbCGT enzyme product using aglycon **14** and UDPG as substrates. a) HPLC-UV analysis of the AbCGT catalyzing reaction; b) Typical positive ion MS for the peak of **14a**.

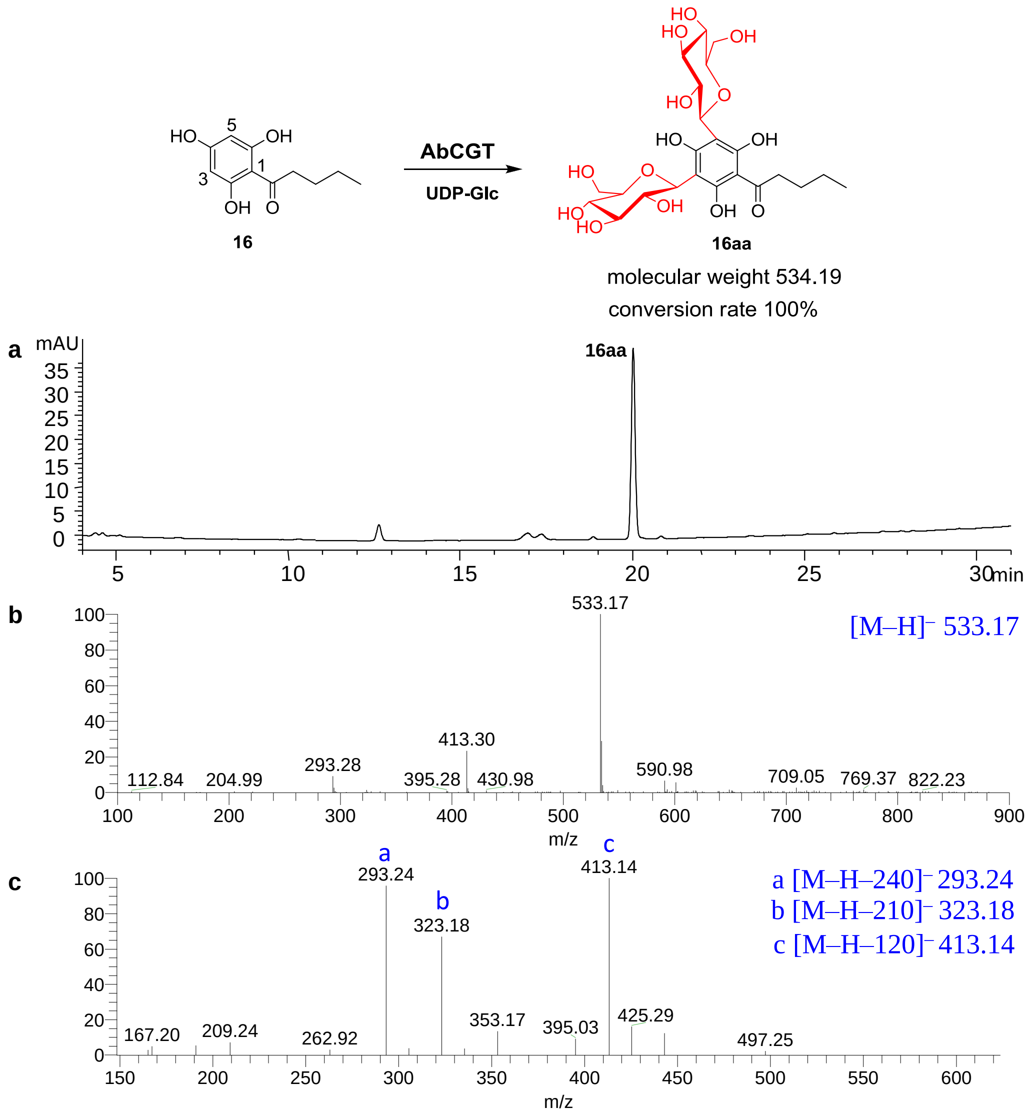

Supplementary Figure 21. HPLC-UV/ESI-MS analysis of AbCGT enzyme product using aglycon **16** and UDPG as substrates. a) HPLC-UV analysis of the AbCGT catalyzing reaction; b) Typical negative ion MS for the peak of **16aa**; c) Typical negative ion MS^2 for the peak of **16aa**.

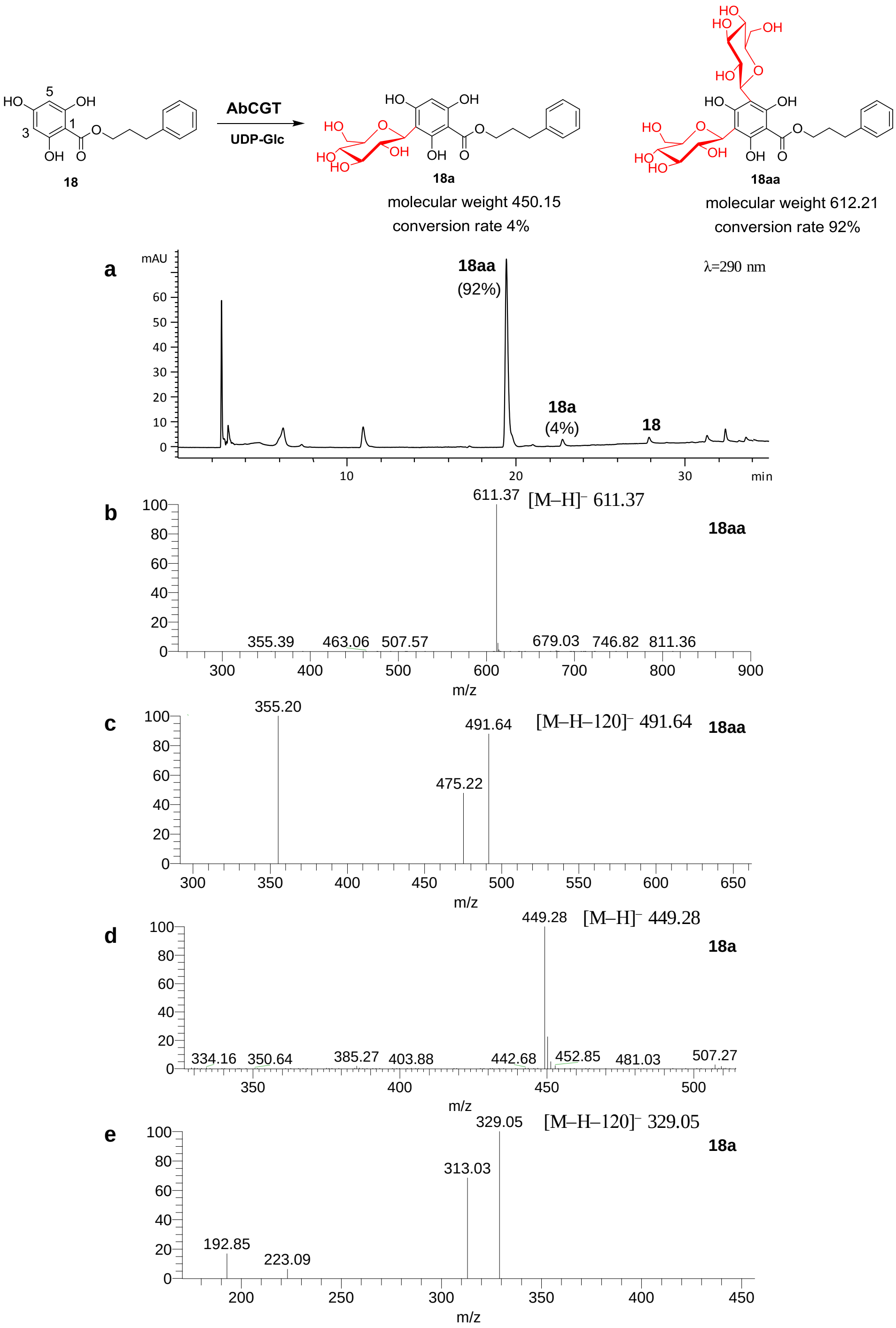

Supplementary Figure 22. HPLC-UV/ESI-MS analysis of AbCGT enzyme products using aglycon **18** and UDPG as substrates. a) HPLC-UV analysis of the AbCGT catalyzing reaction. The total conversion rate and the yields of mono-*C*-glycoside (**18a**) and di-*C*-glycoside (**18aa**) are shown, respectively; b) Typical negative ion MS for the peak of **18aa**; c) Typical negative MS^2 for peak of **18aa**; d) Typical negative ion MS for the peak of **18a**; e) Typical negative MS^2 for peak of **18a**.

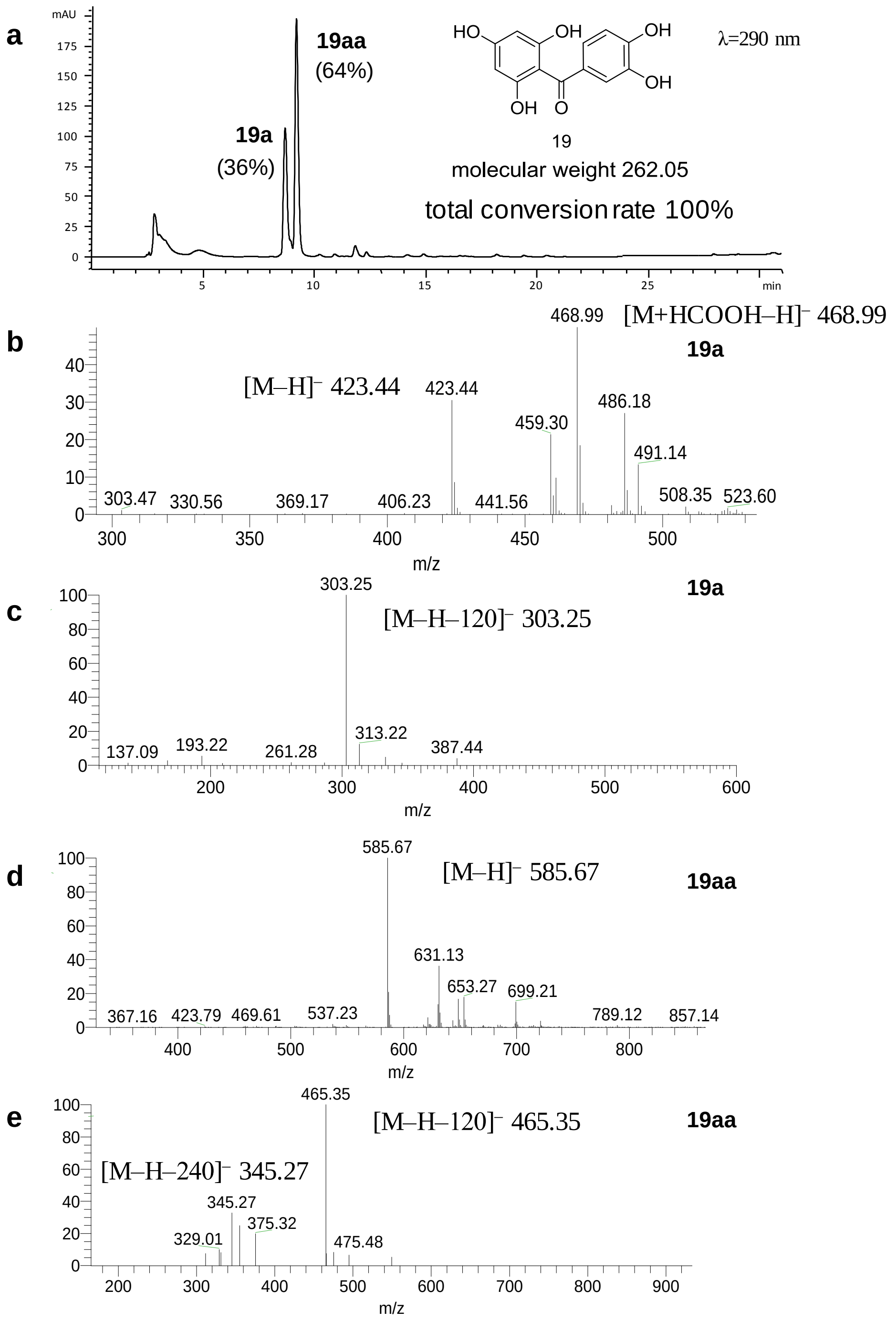

Supplementary Figure 23. HPLC-UV/ESI-MS analysis of AbCGT enzyme products using aglycon **19** and UDPG as substrates. a) HPLC-UV analysis of the AbCGT catalyzing reaction. The total conversion rate and the yields of mono-*C*-glycoside (**19a**) and di-*C*-glycoside (**19aa**) are shown; b) Typical negative ion MS for the peak of **19a**; c) Typical negative MS^2 for peak of **19a**; d) Typical negative ion MS for the peak of **19aa**; e) Typical negative MS^2 for peak of **19aa**.

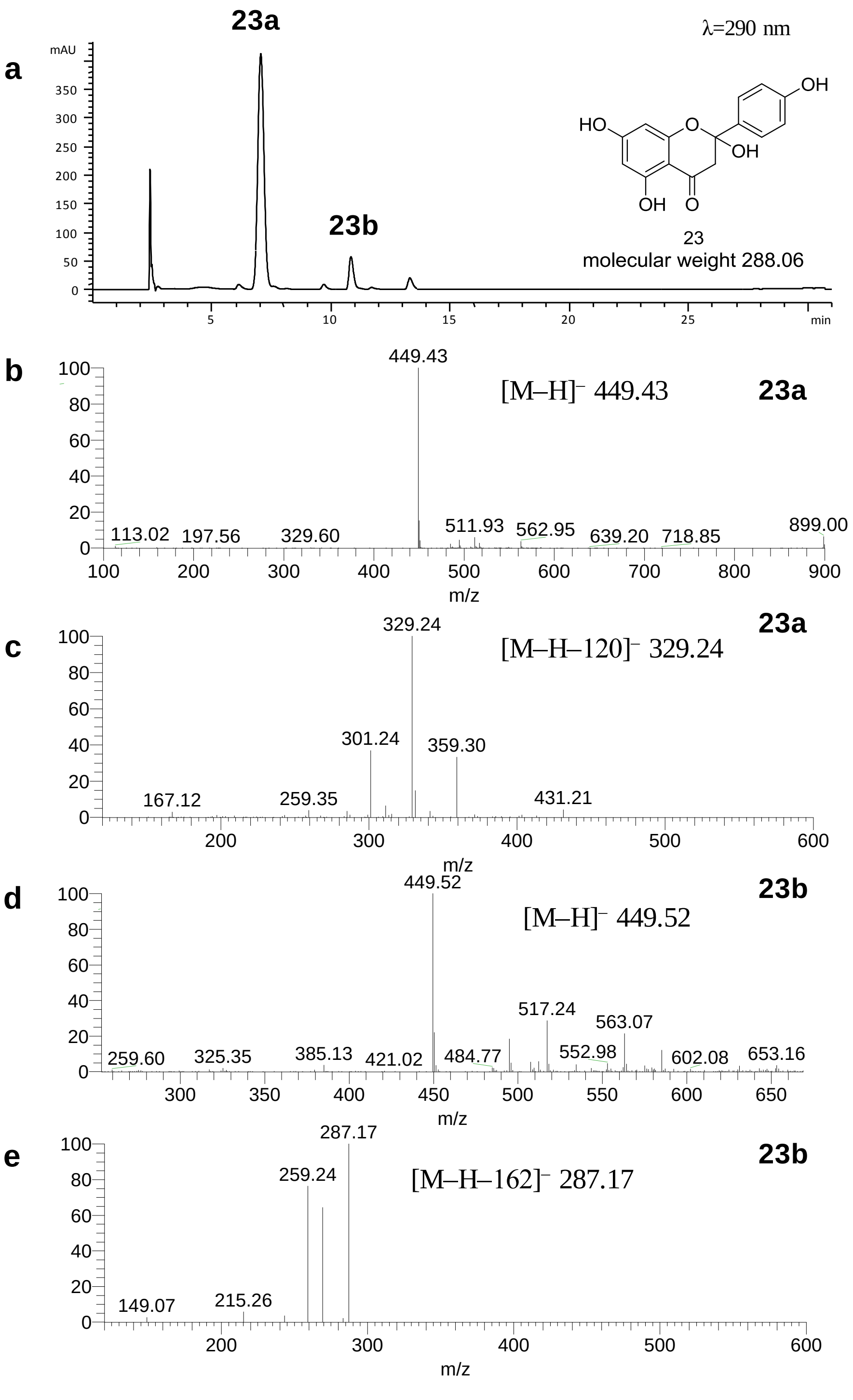

Supplementary Figure 24. HPLC-UV/ESI-MS analysis of AbCGT enzyme products using aglycon **23** and UDPG as substrates. a) HPLC-UV analysis of the AbCGT catalyzing reaction; b) Typical negative ion MS for the peak of **23a**; c) Typical negative MS^2 for peak of **23a** (*C*-glycoside); d) Typical negative ion MS for the peak of **23b**; e) Typical negative MS^2 for peak of **23b** (*O*-glycoside).

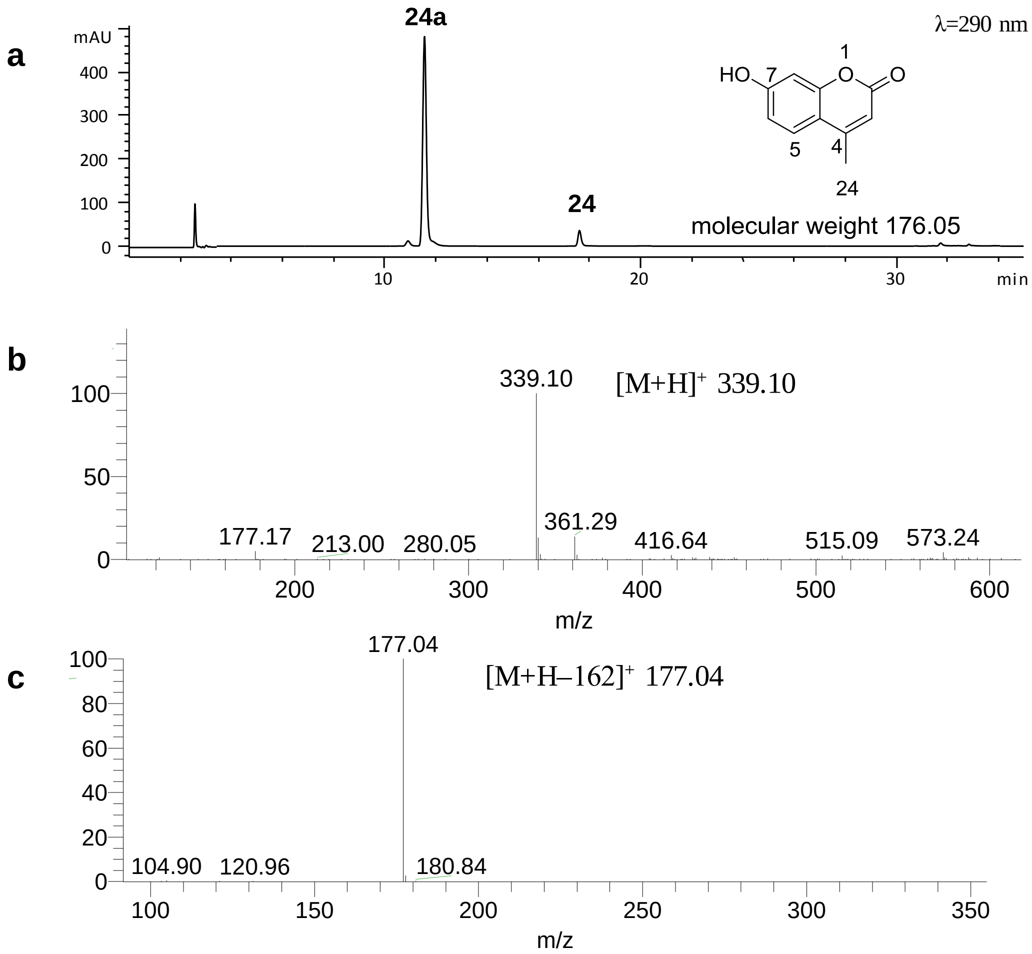

Supplementary Figure 25. HPLC-UV/ESI-MS analysis of AbCGT enzyme product using aglycon **24** and UDPG as substrates. A) HPLC-UV analysis of the AbCGT catalyzing reaction; B) Typical positive ion MS for the peak of **24a**; C) Typical positive MS^2 for peak of **24a**.

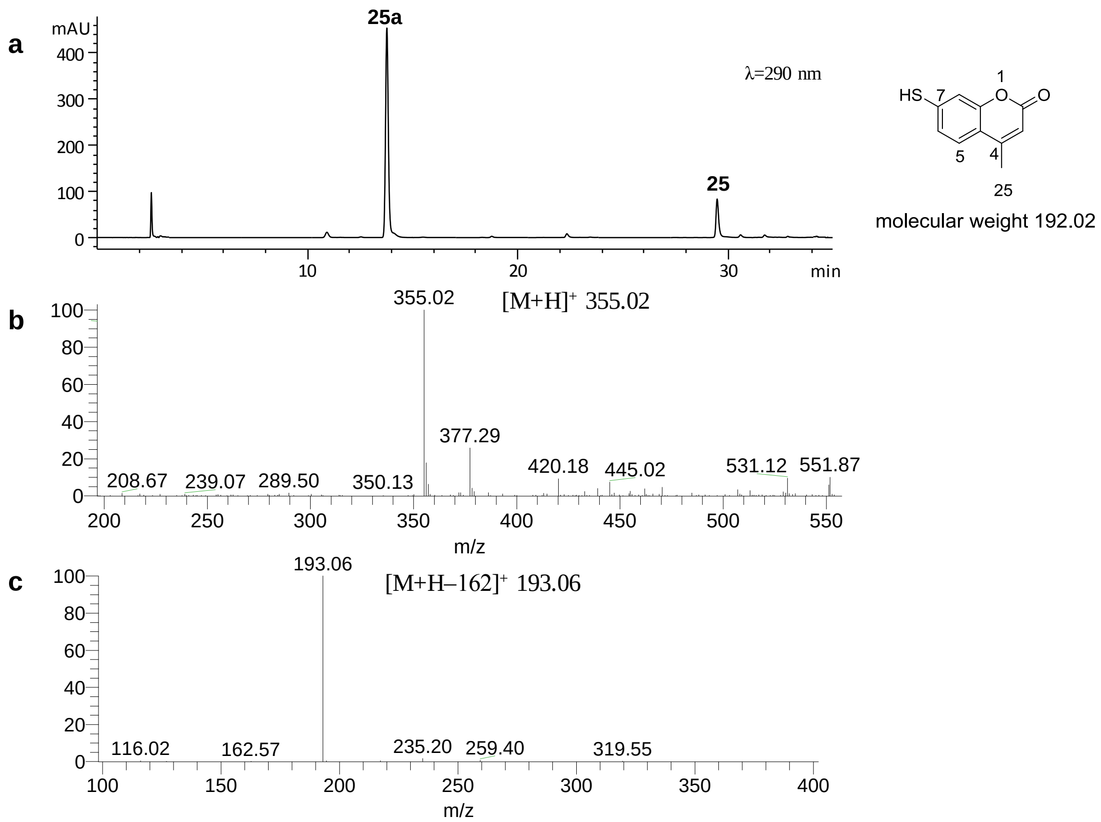

Supplementary Figure 26. HPLC-UV/ESI-MS analysis of AbCGT enzyme product using aglycon **25** and UDPG as substrates. a) HPLC-UV analysis of the AbCGT catalyzing reaction; b) Typical positive ion MS for the peak of **25a**; c) Typical positive MS^2 for peak of **25a**.

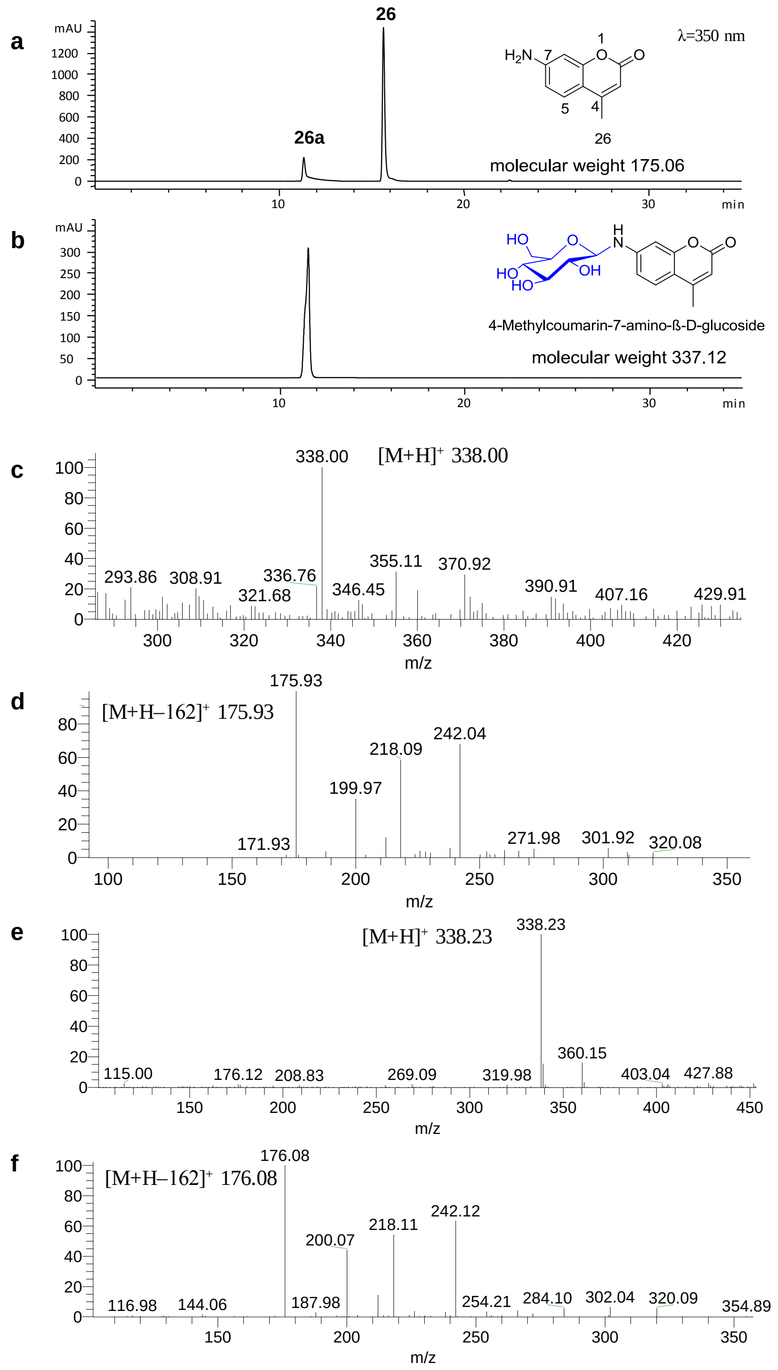

Supplementary Figure 27. HPLC-UV/ESI-MS analysis of AbCGT enzyme product using aglycon **26** and UDPG as substrates. a) HPLC-UV analysis of the AbCGT catalyzing reaction; b) HPLC-UV analysis of the standard 4-Methylcoumarin-7-amino-β-D-glucoside; c) Typical positive ion MS for the peak of **26a**; d) Typical positive MS^2 for peak of **26a**. e) Typical positive ion MS for the peak of 4-Methylcoumarin-7-amino-β-D-glucoside; f) Typical positive MS^2 for peak of 4-Methylcoumarin-7-amino-β-D-glucoside.

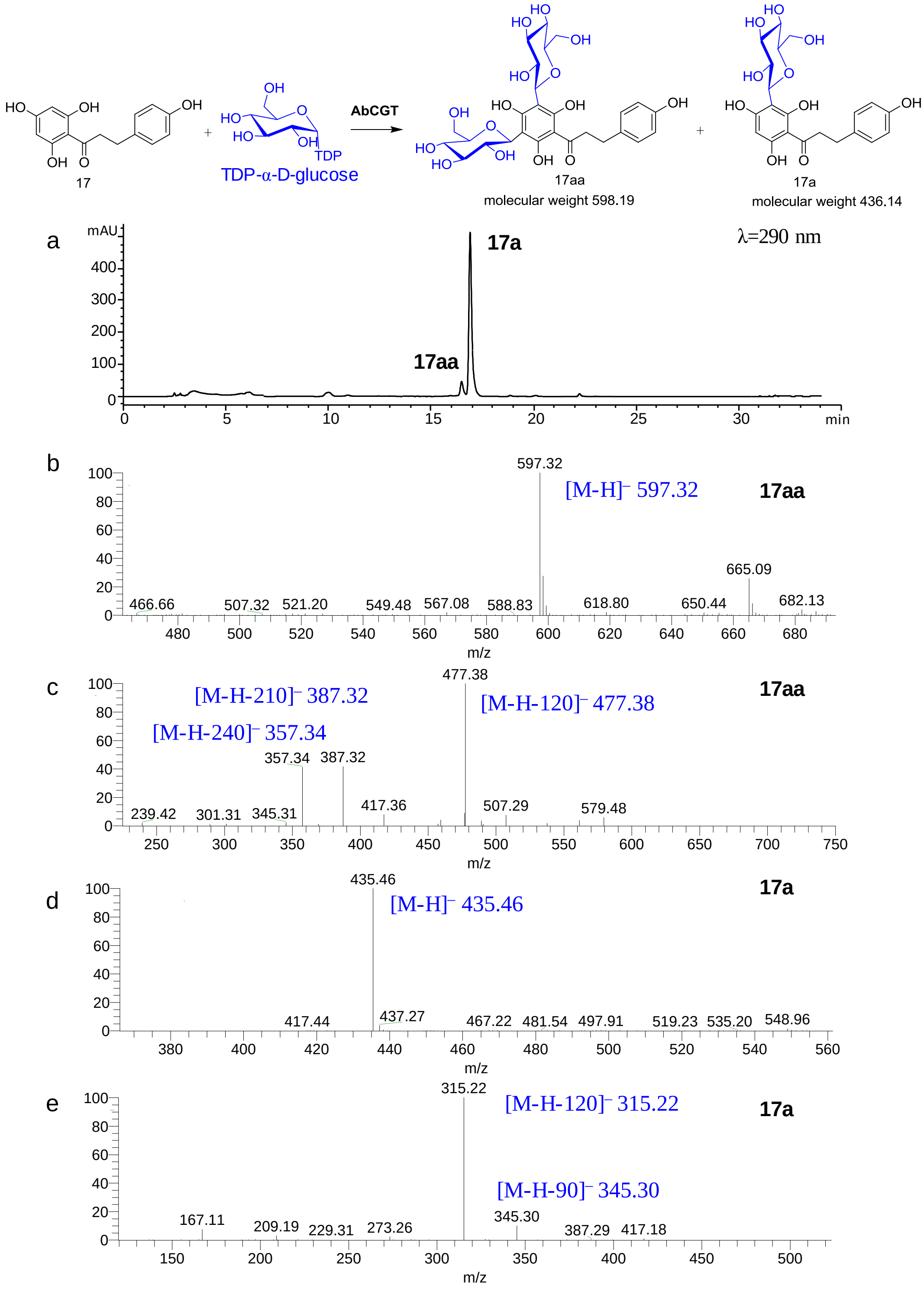

Supplementary Figure 28. HPLC-UV/ESI-MS analysis of AbCGT enzyme products using phloretin (**17**) and TDP-α-D-glucose as substrates. a) HPLC-UV analysis of the AbCGT catalyzing reaction; b) Typical negative ion MS for the peak of **17aa**; c) Typical negative MS^2 for peak of **17aa**; d) Typical negative ion MS for the peak of **17a** $[M–H]^-$; E) Typical negative MS^2 for peak of **17a**.

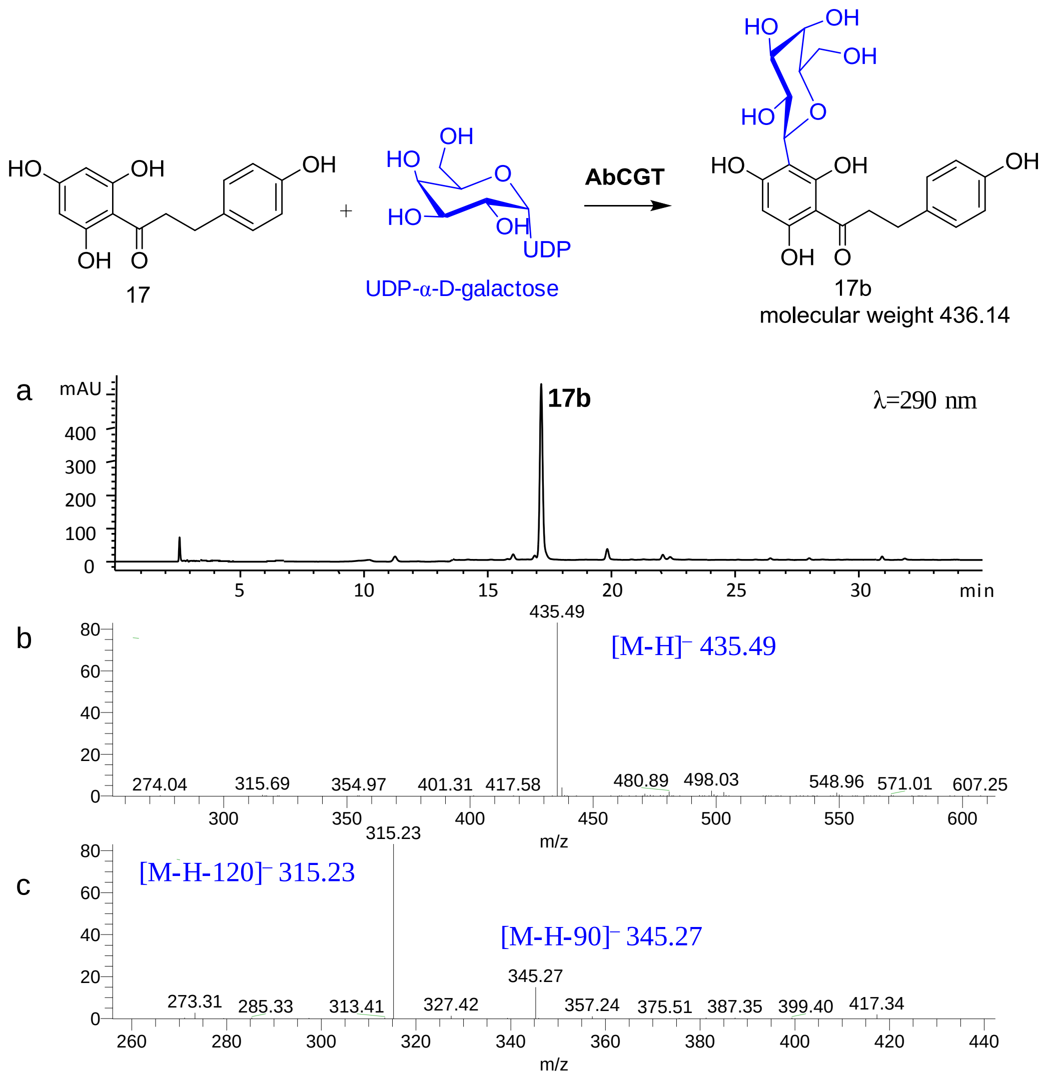

Supplementary Figure 29. HPLC-UV/ESI-MS analysis of AbCGT enzyme product using phloretin (**17**) and UDP-α-D-galactose as substrates. a) HPLC-UV analysis of the AbCGT catalyzing reaction; b) Typical negative ion MS for the peak of **17b**; c) Typical negative MS^2 for peak of **17b**.

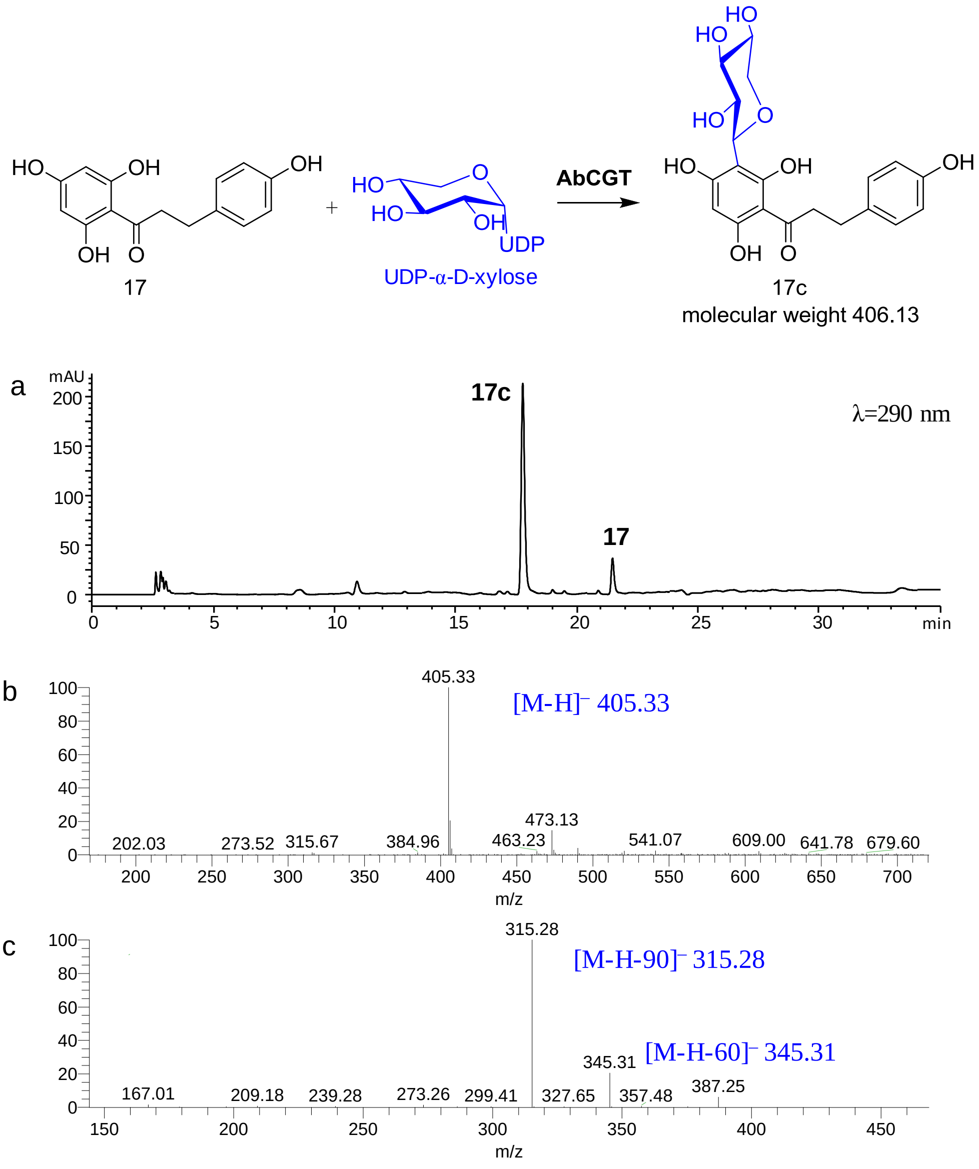

Supplementary Figure 30. HPLC-UV/ESI-MS analysis of AbCGT enzyme product using phloretin (**17**) and UDP-α-D-xylose as substrates. a) HPLC-UV analysis of the AbCGT catalyzing reaction; b) Typical negative ion MS for the peak of **17c**; c) Typical negative MS^2 for peak of **17c**.

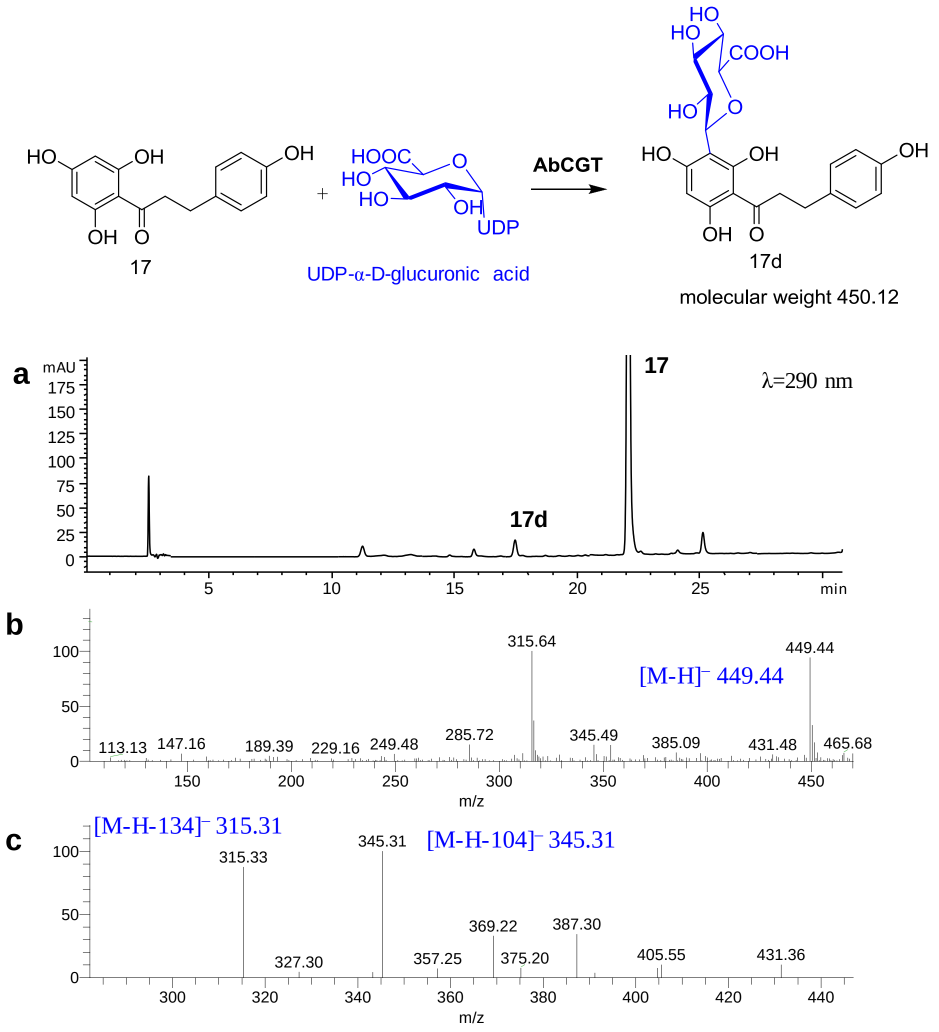

Supplementary Figure 31. HPLC-UV/ESI-MS analysis of AbCGT enzyme product using phloretin (**17**) and UDP-α-D-glucuronic acid as substrates. a) HPLC-UV analysis of the AbCGT catalyzing reaction; b) Typical negative ion MS for the peak of **17d**; c) Typical negative MS^2 for peak of **17d**.

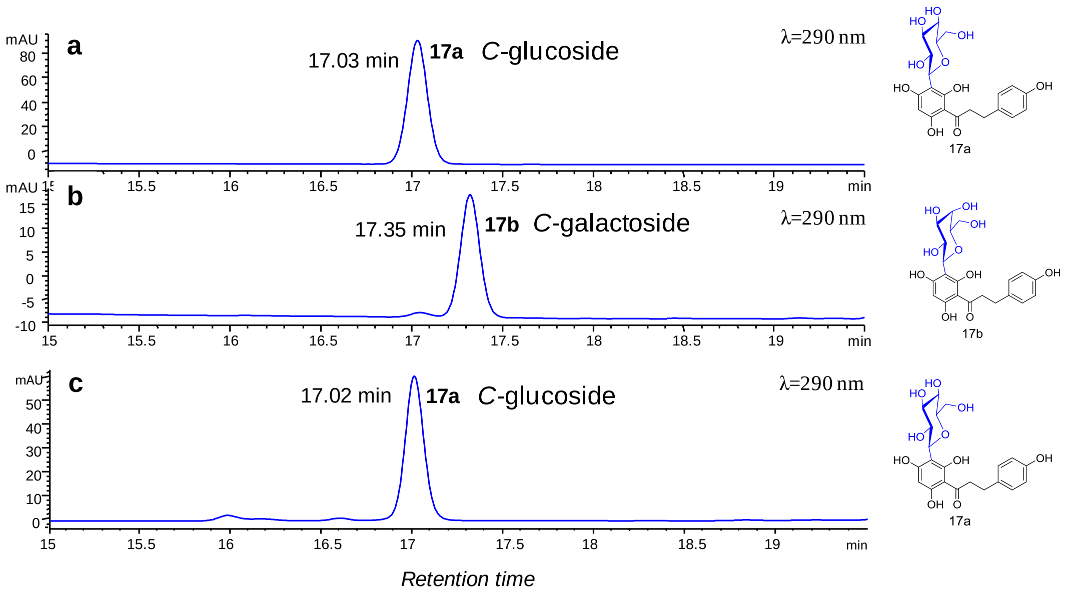

Supplementary Figure 32. HPLC analysis of AbCGT catalyzing reactions *in vitro* and *in vivo*. a) AbCGT catalyzing reactions *in vitro* with UDP-Glc as the sugar donor; b) AbCGT catalyzing reactions *in vitro* with UDP-Gal as the sugar donor; c) whole cell catalyzing reactions *in vivo*. Phloretin (**17**) was used as an acceptor.

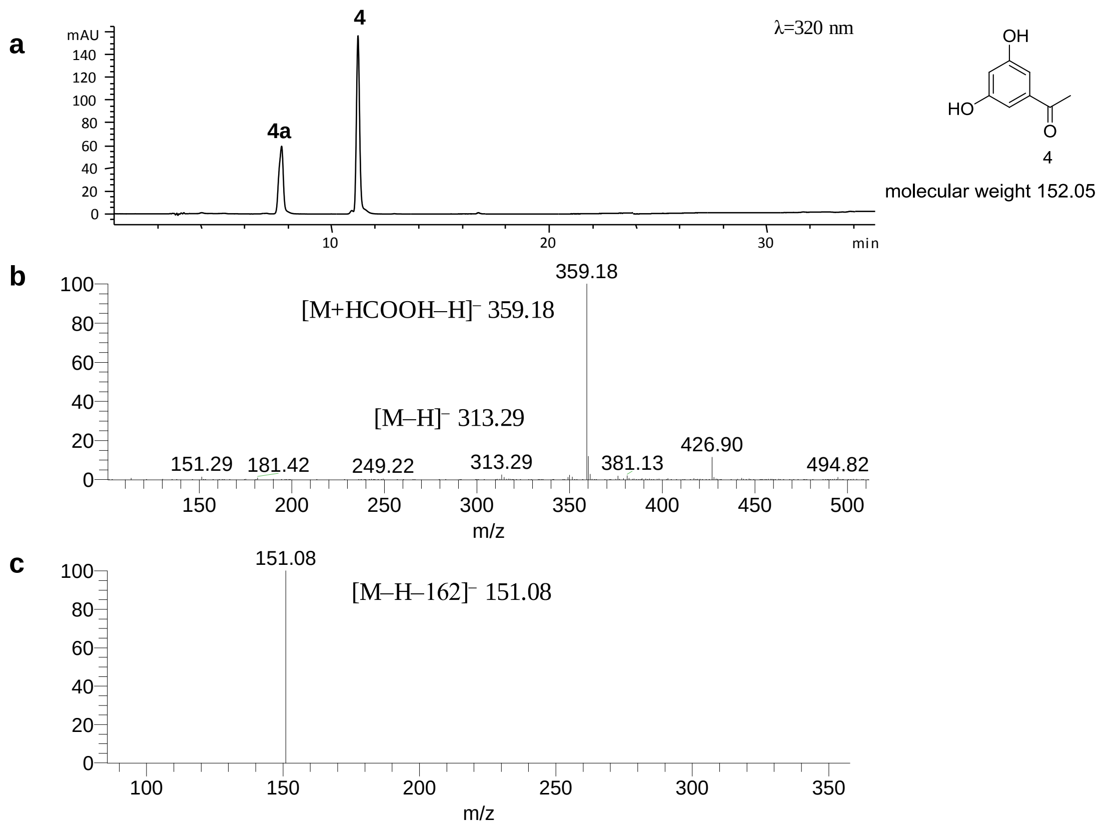

Supplementary Figure 33. HPLC-UV/ESI-MS analysis of AbCGT enzyme product using aglycon **4** and UDPG as substrates. a) HPLC-UV analysis of the AbCGT catalyzing reaction; b) Typical negative ion MS for the peak of **4a**; c) Typical negative MS^2 for peak of **4a**.

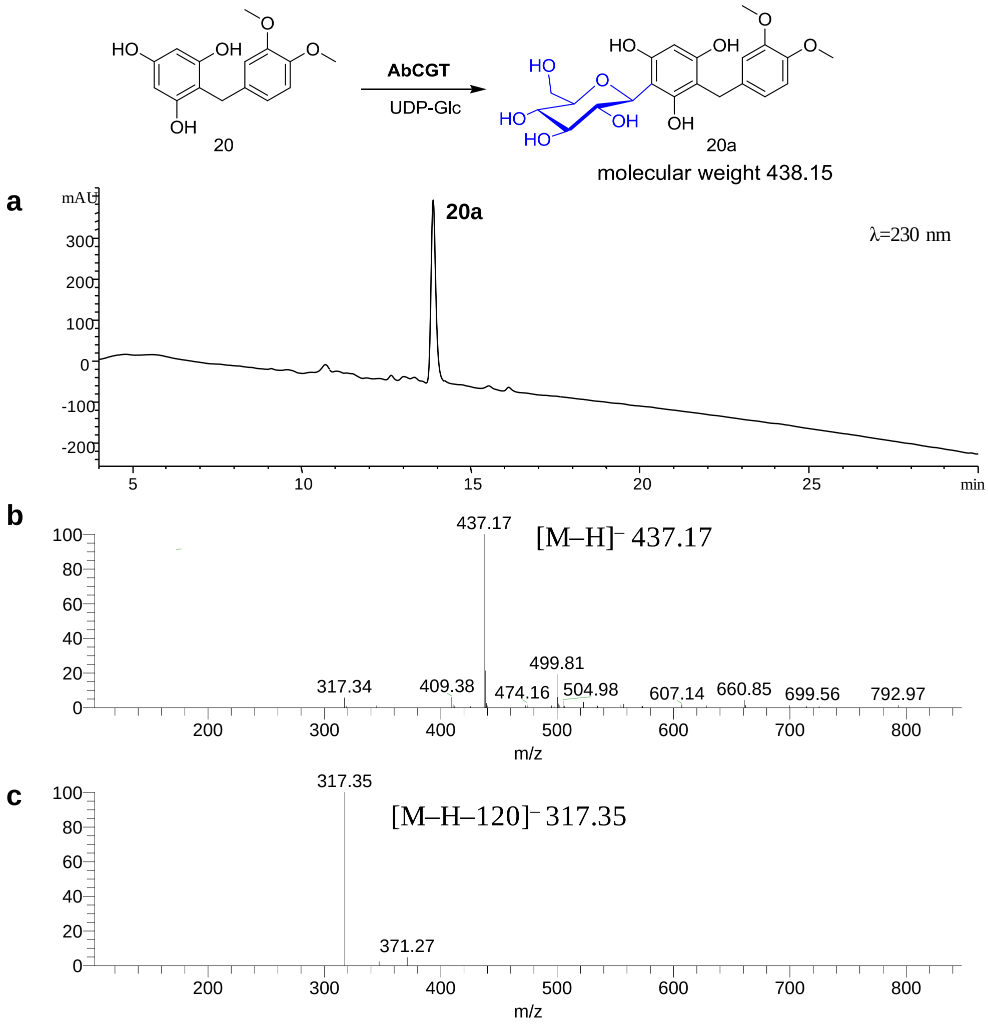

Supplementary Figure 34. HPLC-UV/ESI-MS analysis of AbCGT enzyme product using aglycon **20** and UDPG as substrates. a) HPLC-UV analysis of the AbCGT catalyzing reaction; b) Typical negative ion MS for the peak of **20a**; c) Typical negative MS^2 for peak of **20a**.

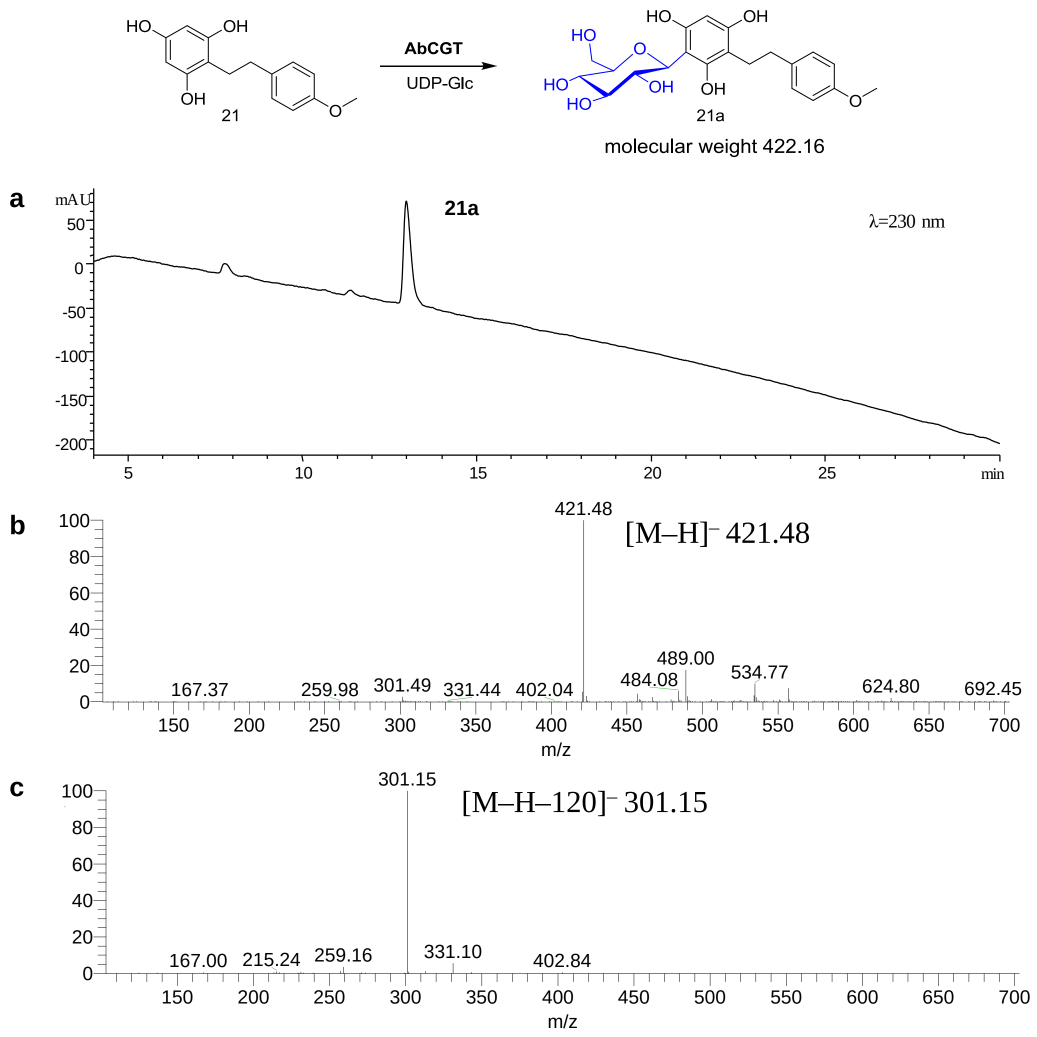

Supplementary Figure 35. HPLC-UV/ESI-MS analysis of AbCGT enzyme product using aglycon **21** and UDPG as substrates. a) HPLC-UV analysis of the AbCGT catalyzing reaction; b) Typical negative ion MS for the peak of **21a**; c) Typical negative MS^2 for peak of **21a**.

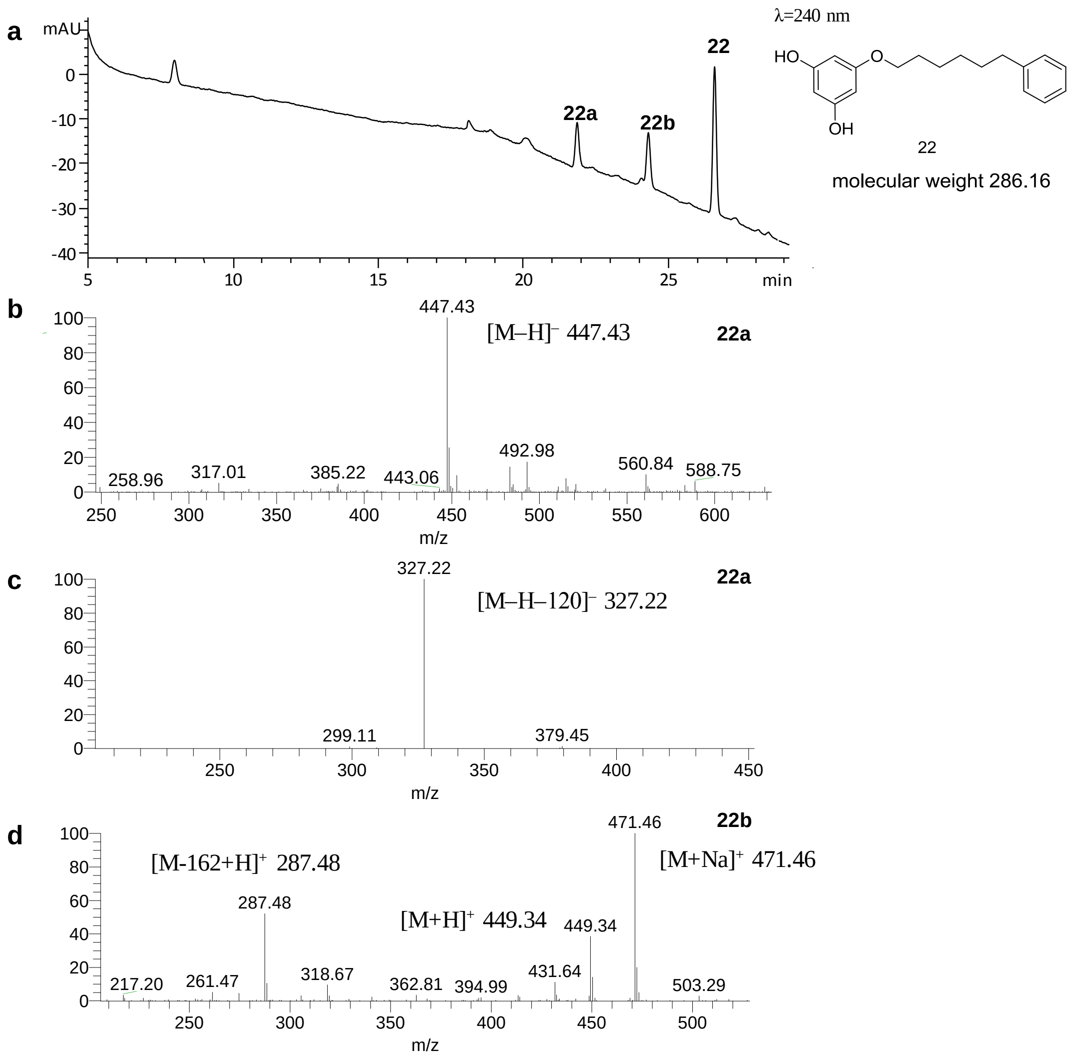

Supplementary Figure 36. HPLC-UV/ESI-MS analysis of AbCGT enzyme products using aglycon **22** and UDPG as substrates. a) HPLC-UV analysis of the AbCGT catalyzing reaction; b) Typical negative ion MS for the peak of **22a**; c) Typical negative MS^2 for peak of **22a**; d) Typical negative ion MS for the peak of **22b**; e) Typical negative MS^2 for peak of **22b**.

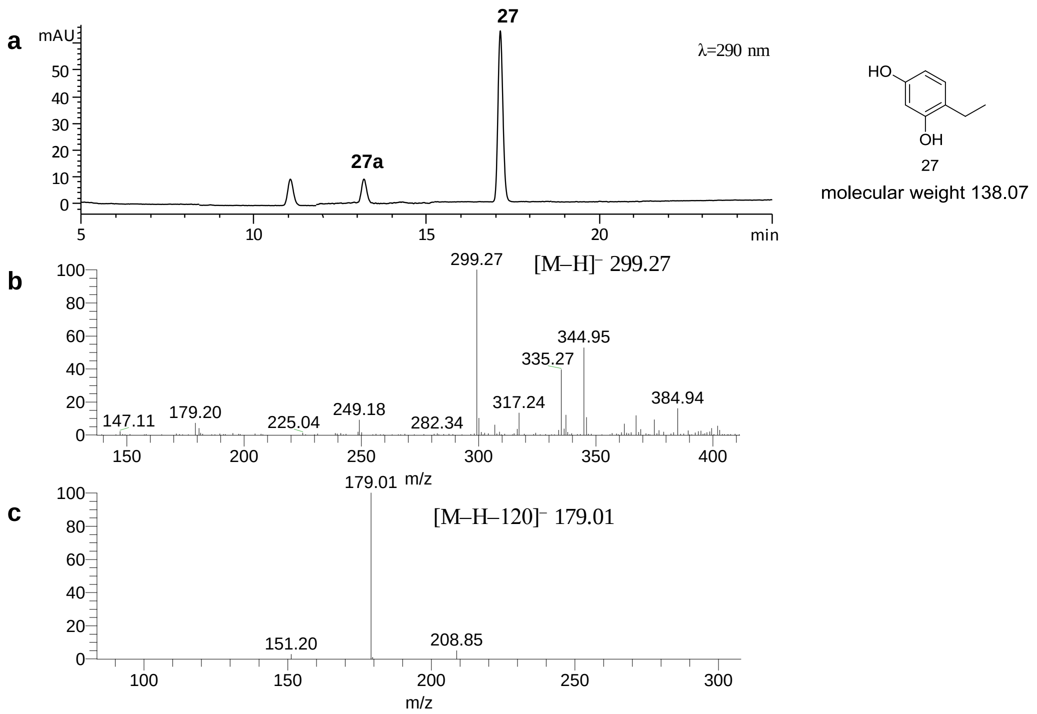

Supplementary Figure 37. HPLC-UV/ESI-MS analysis of AbCGT enzyme product using aglycon **27** and UDPG as substrates. a) HPLC-UV analysis of the AbCGT catalyzing reaction; b) Typical negative ion MS for the peak of **27a**; c) Typical negative MS^2 for peak of **27a**.

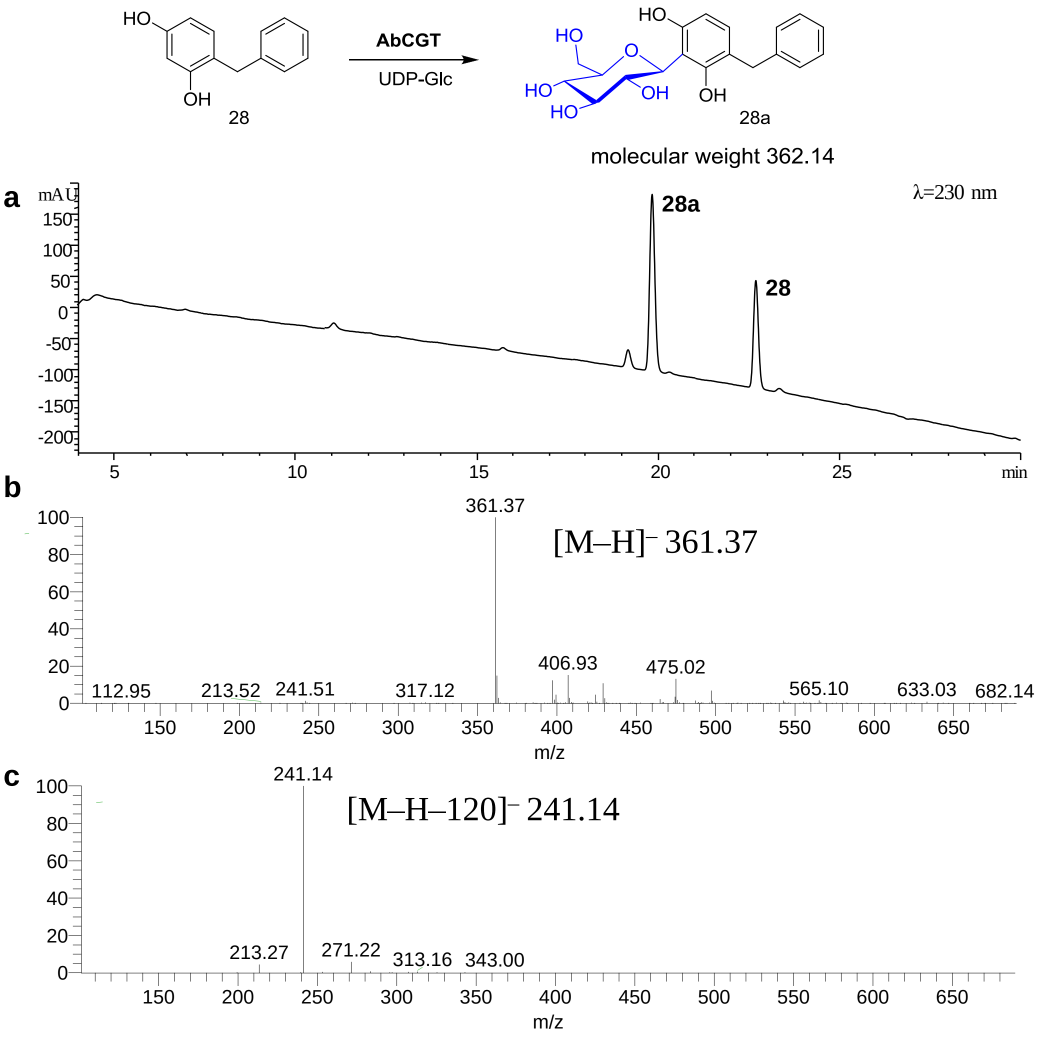

Supplementary Figure 38. HPLC-UV/ESI-MS analysis of AbCGT enzyme product using aglycon **28** and UDPG as substrates. a) HPLC-UV analysis of the AbCGT catalyzing reaction; b) Typical negative ion MS for the peak of **28a**; c) Typical negative MS^2 for peak of **28a**.

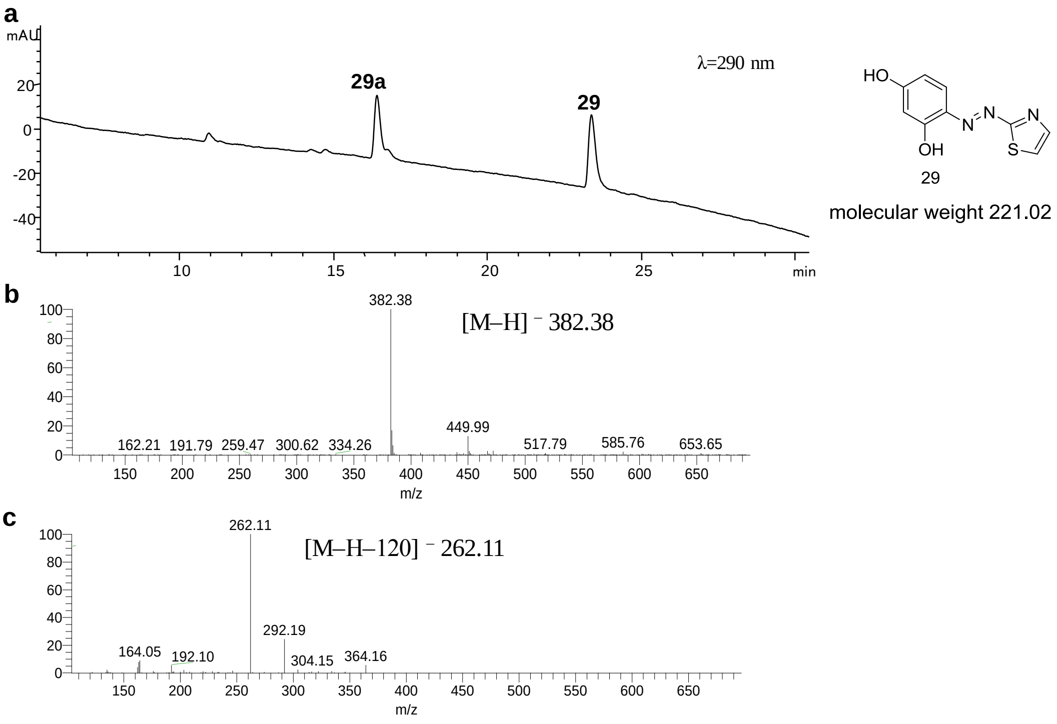

Supplementary Figure 39. HPLC-UV/ESI-MS analysis of AbCGT enzyme product using aglycon **29** and UDPG as substrates. a) HPLC-UV analysis of the AbCGT catalyzing reaction; b) Typical negative ion MS for the peak of **29a**; c) Typical negative MS^2 for peak of **29a**.

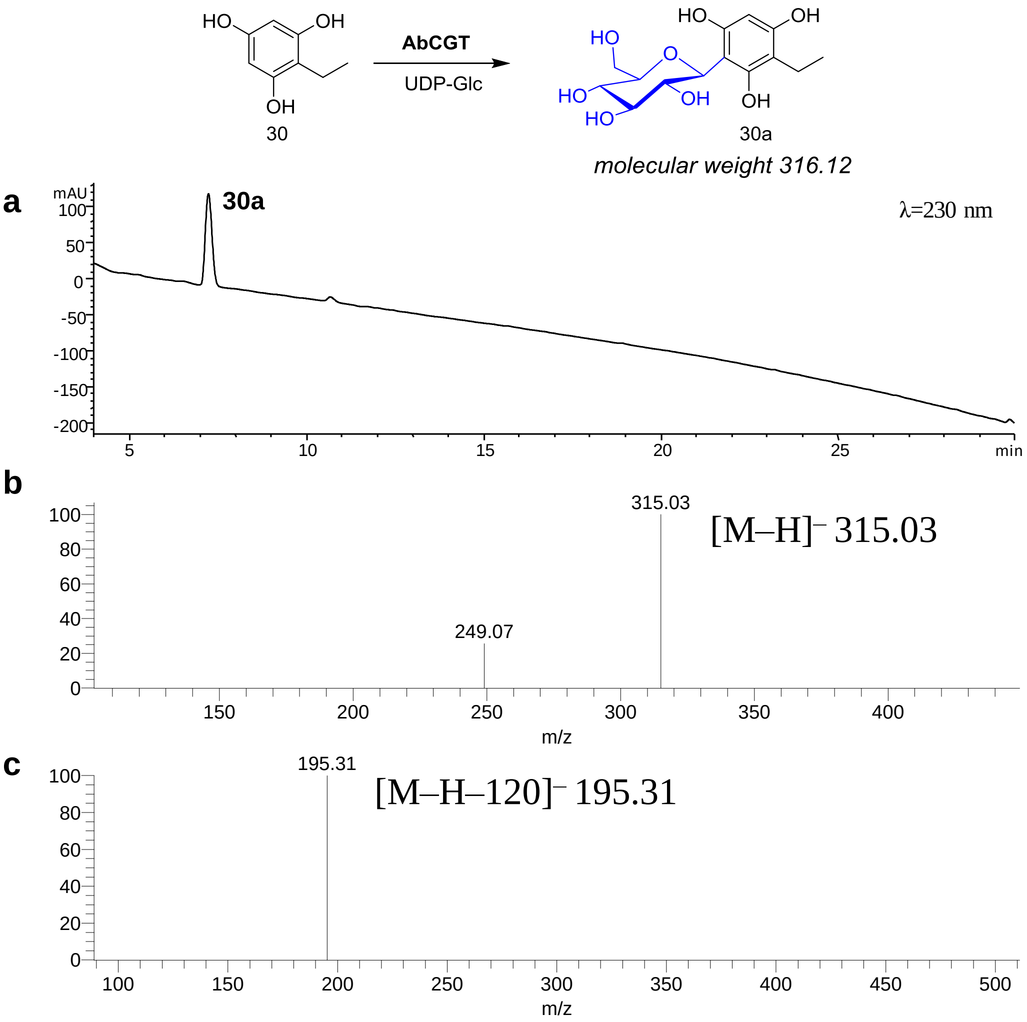

Supplementary Figure 40 HPLC-UV/ESI-MS analysis of AbCGT enzyme product using aglycon **30** and UDPG as substrates. a) HPLC-UV analysis of the AbCGT catalyzing reaction; b) Typical negative ion MS for the peak of **30a**; c) Typical negative MS^2 for peak of **30a**.

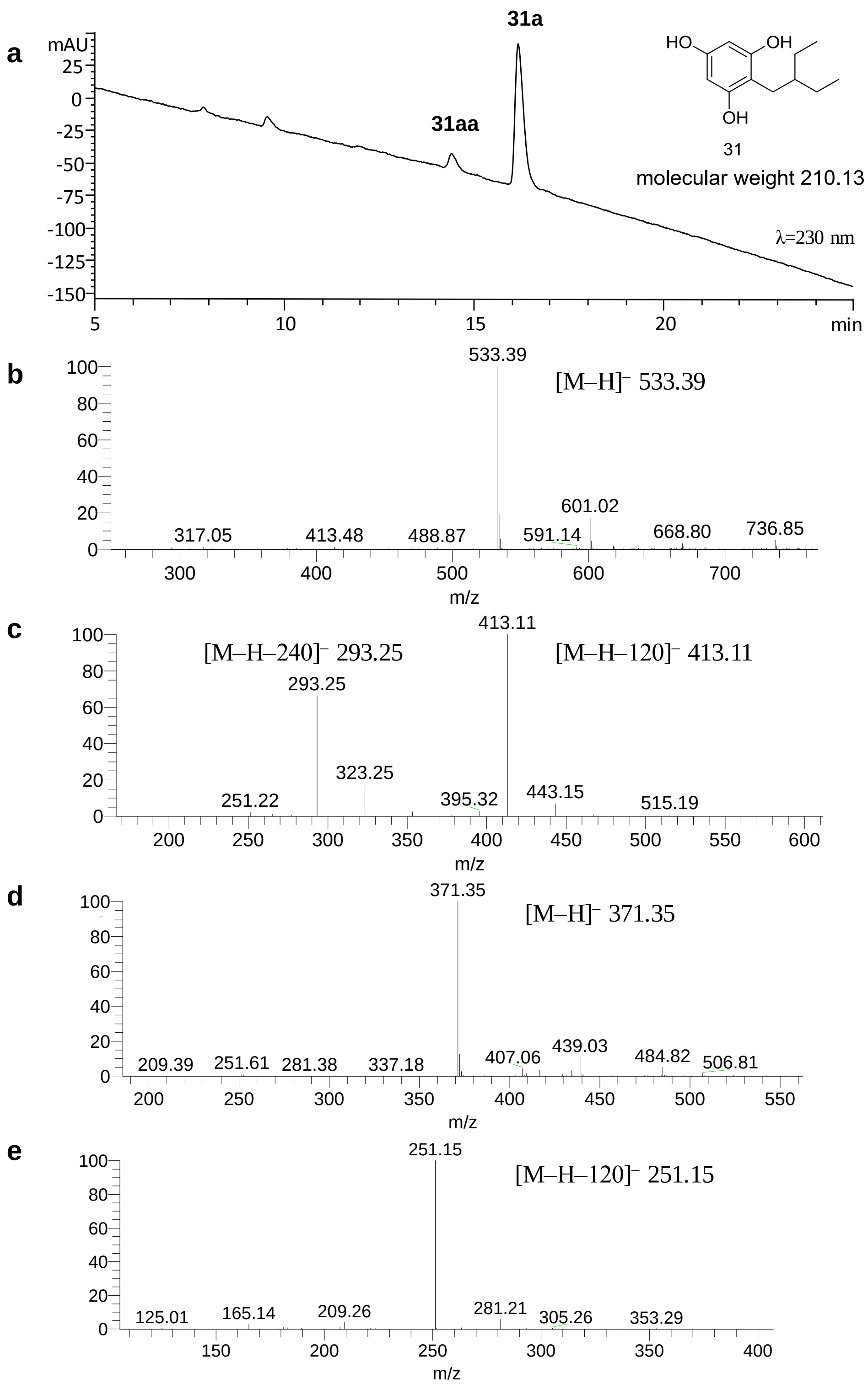

Supplementary Figure 41. HPLC-UV/ESI-MS analysis of AbCGT enzyme products using aglycon **31** and UDPG as substrates. a) HPLC-UV analysis of the AbCGT catalyzing reaction; b) Typical negative ion MS for the peak of **31aa**; c) Typical negative MS^2 for peak of **31aa**; d) Typical negative ion MS for the peak of **31a**; e) Typical negative MS^2 for peak of **31a**.

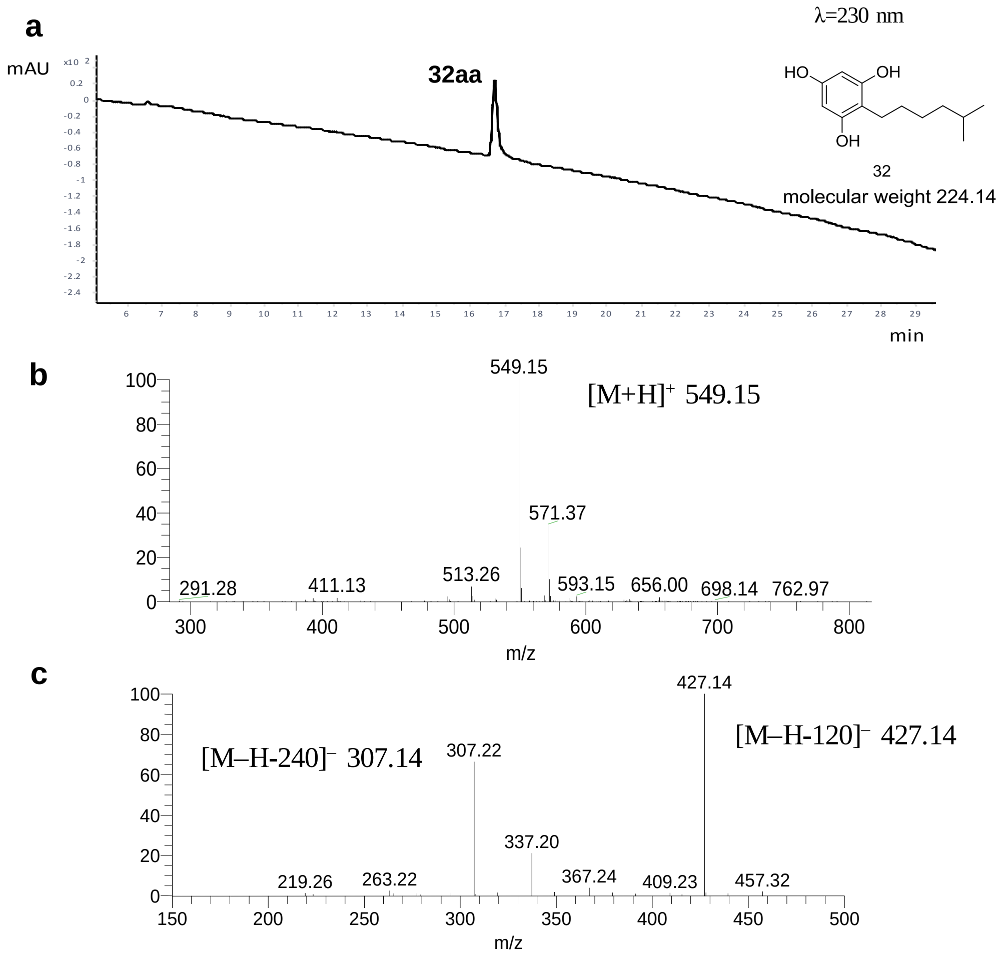

Supplementary Figure 42. HPLC-UV/ESI-MS analysis of AbCGT enzyme product using aglycon **32** and UDPG as substrates. a) HPLC-UV analysis of the AbCGT catalyzing reaction; b) Typical positive ion MS for the peak of **32aa**; c) Typical negative MS^2 for peak of **32aa**.

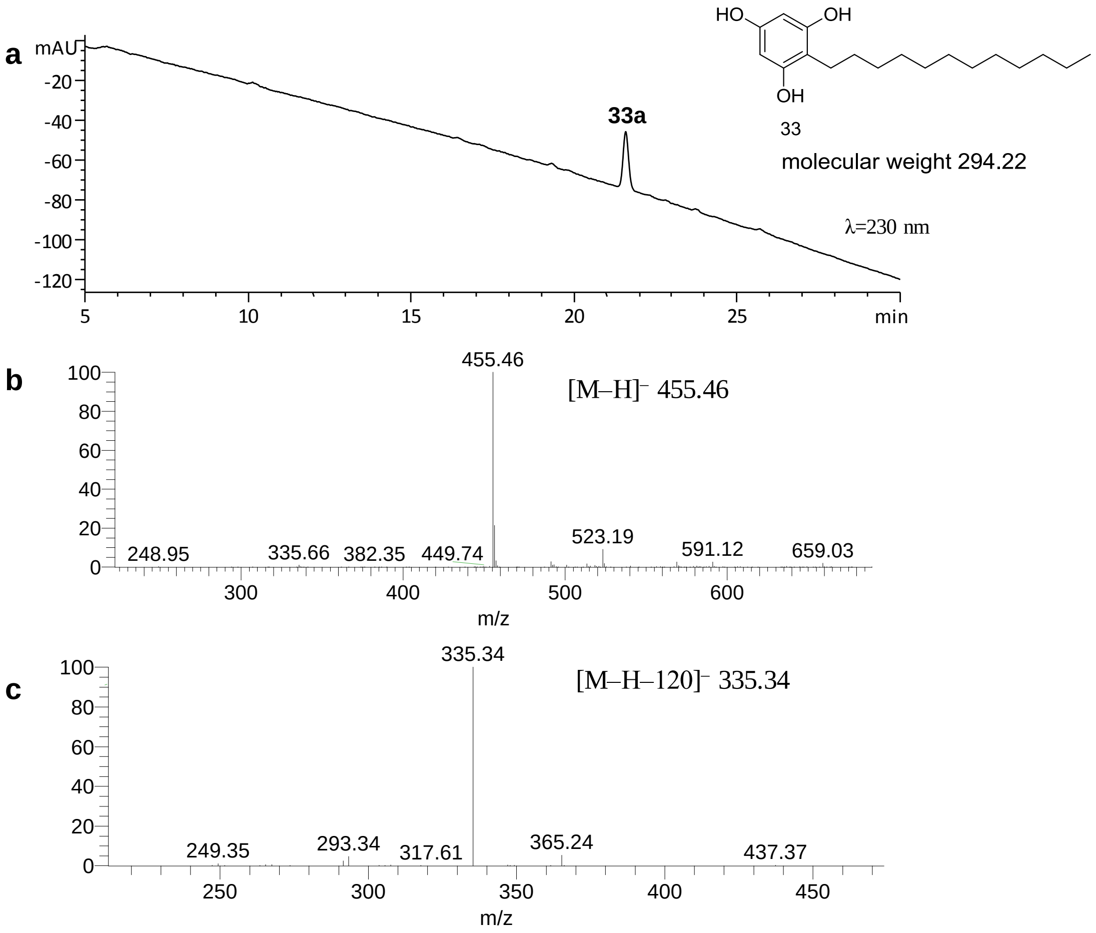

Supplementary Figure 43. HPLC-UV/ESI-MS analysis of AbCGT enzyme product using aglycon **33** and UDPG as substrates. a) HPLC-UV analysis of the AbCGT catalyzing reaction; b) Typical negative ion MS for the peak of **33a**; c) Typical negative MS^2 for peak of **33a**.

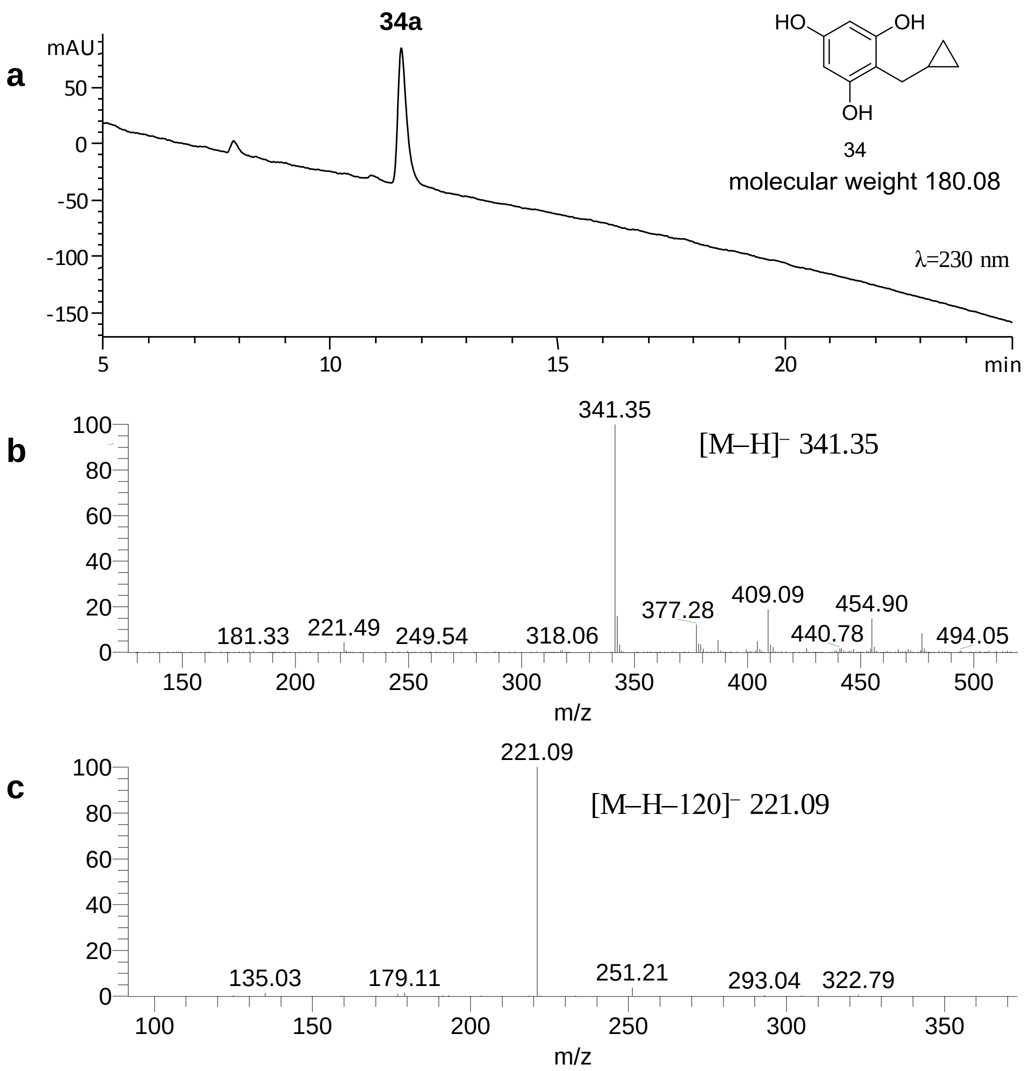

Supplementary Figure 44. HPLC-UV/ESI-MS analysis of AbCGT enzyme product using aglycon **34** and UDPG as substrates. a) HPLC-UV analysis of the AbCGT catalyzing reaction; b) Typical negative ion MS for the peak of **34a**; c) Typical negative MS^2 for peak of **34a**.

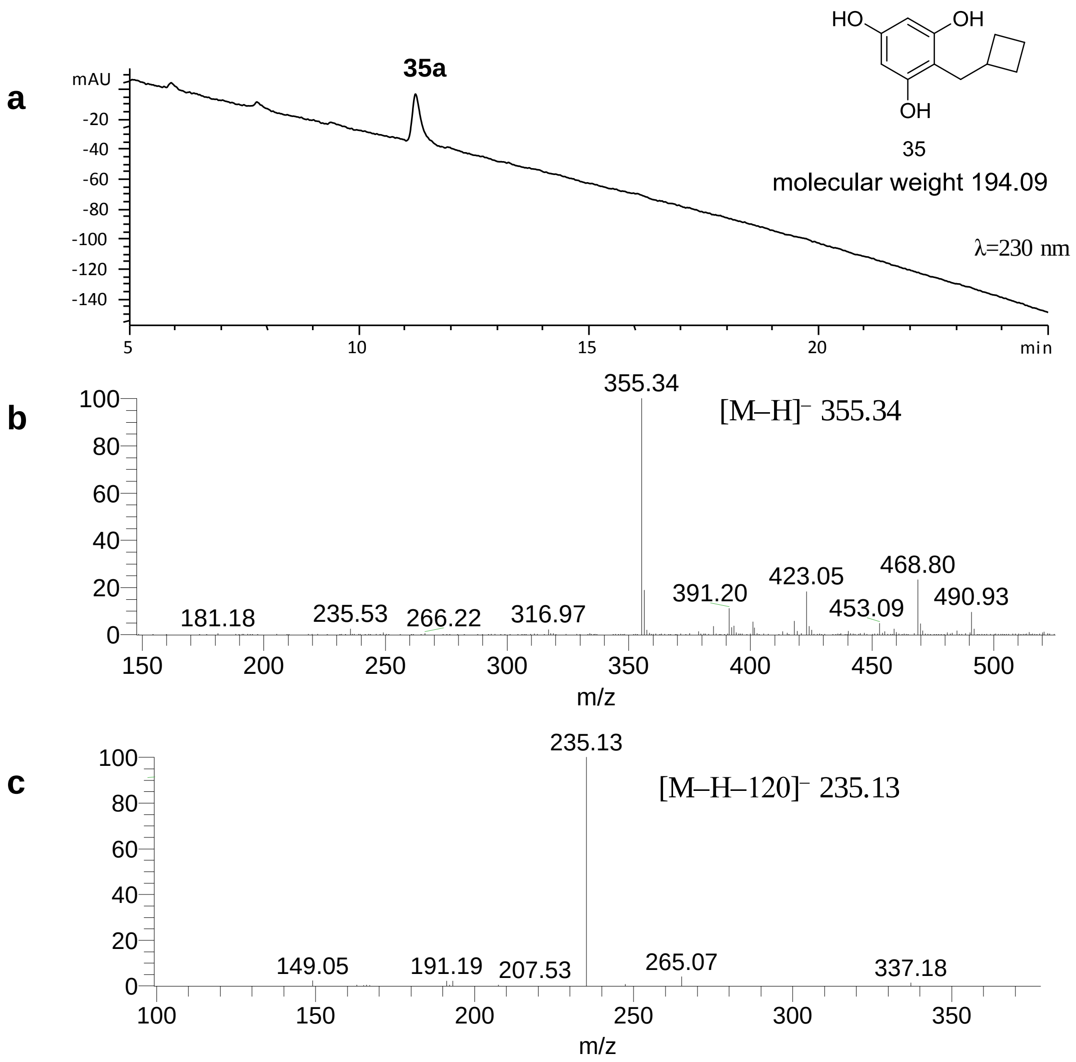

Supplementary Figure 45. HPLC-UV/ESI-MS analysis of AbCGT enzyme product using aglycon **35** and UDPG as substrates. a) HPLC-UV analysis of the AbCGT catalyzing reaction; b) Typical negative ion MS for the peak of **35a**; c) Typical negative MS^2 for peak of **35a**.

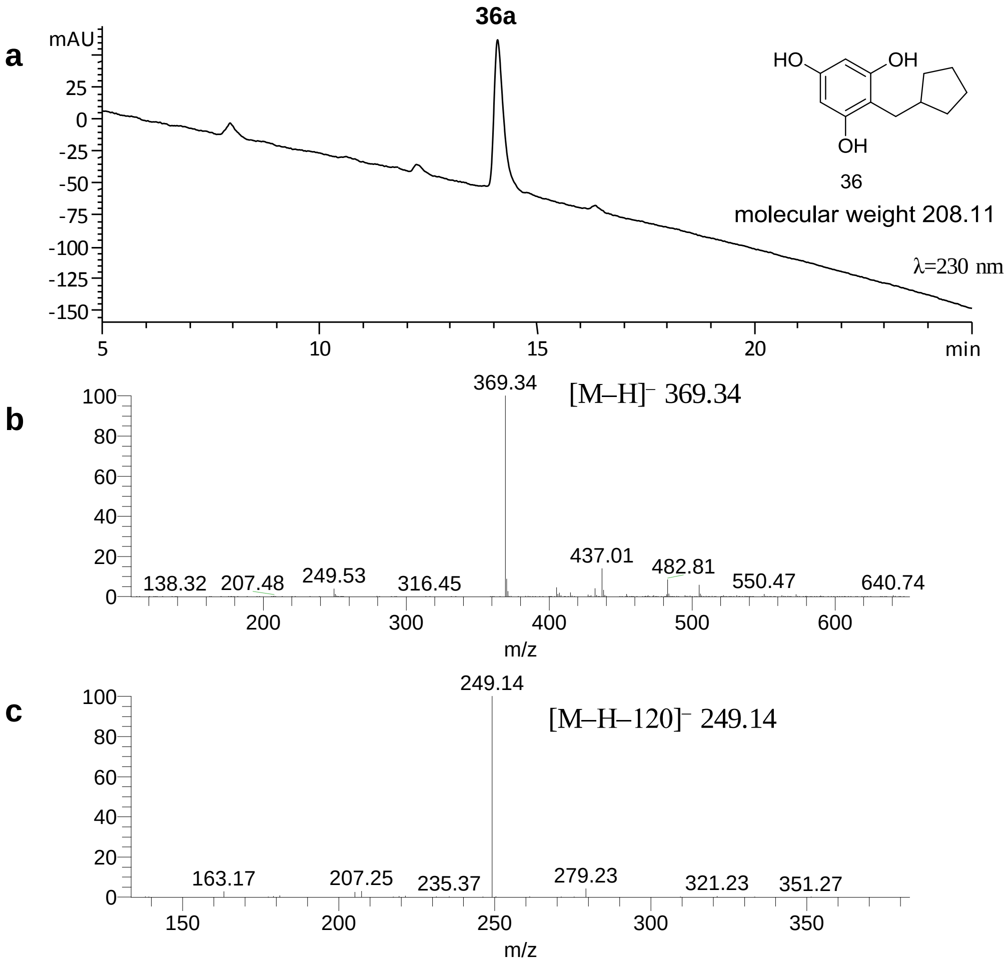

Supplementary Figure 46. HPLC-UV/ESI-MS analysis of AbCGT enzyme product using aglycon **36** and UDPG as substrates. a) HPLC-UV analysis of the AbCGT catalyzing reaction; b) Typical negative ion MS for the peak of **36a**; c) Typical negative MS^2 for peak of **36a**.

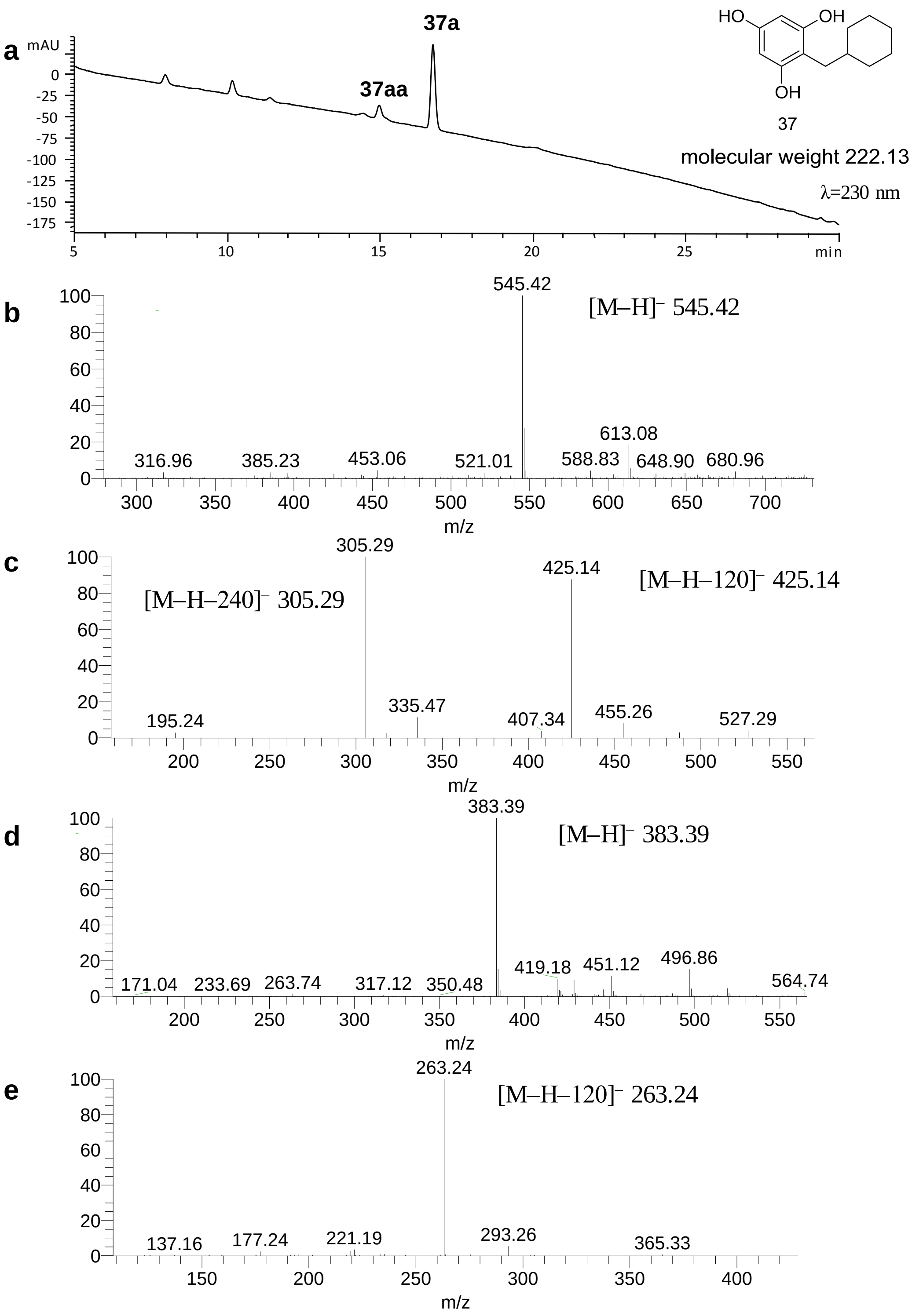

Supplementary Figure 47. HPLC-UV/ESI-MS analysis of AbCGT enzyme products using aglycon **37** and UDPG as substrates. a) HPLC-UV analysis of the AbCGT catalyzing reaction; b) Typical negative ion MS for the peak of **37aa**; c) Typical negative MS^2 for peak of **37aa**; d) Typical negative ion MS for the peak of **37a**; e) Typical negative MS^2 for peak of **37a**.

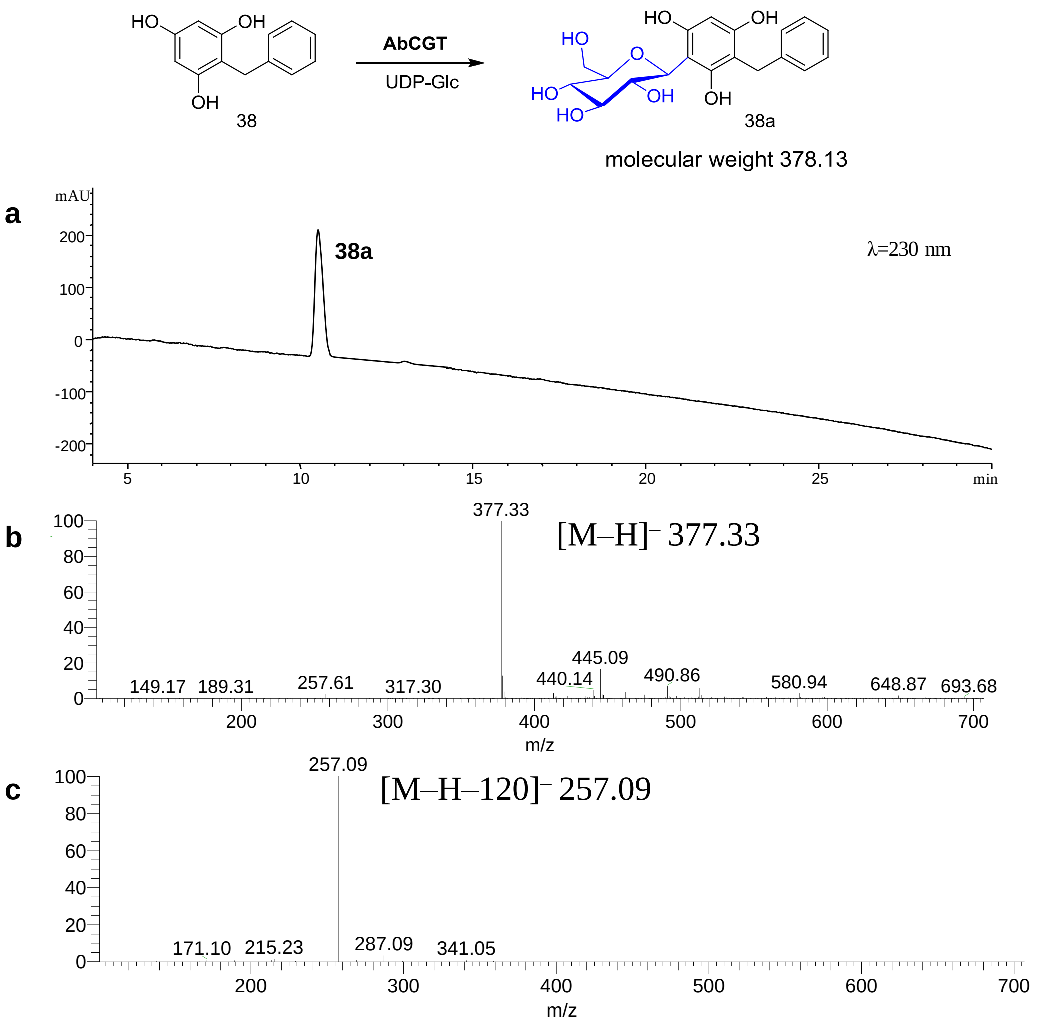

Supplementary Figure 48. HPLC-UV/ESI-MS analysis of AbCGT enzyme product using aglycon **38** and UDPG as substrates. a) HPLC-UV analysis of the AbCGT catalyzing reaction; b) Typical negative ion MS for the peak of **38a**; c) Typical negative MS^2 for peak of **38a**.

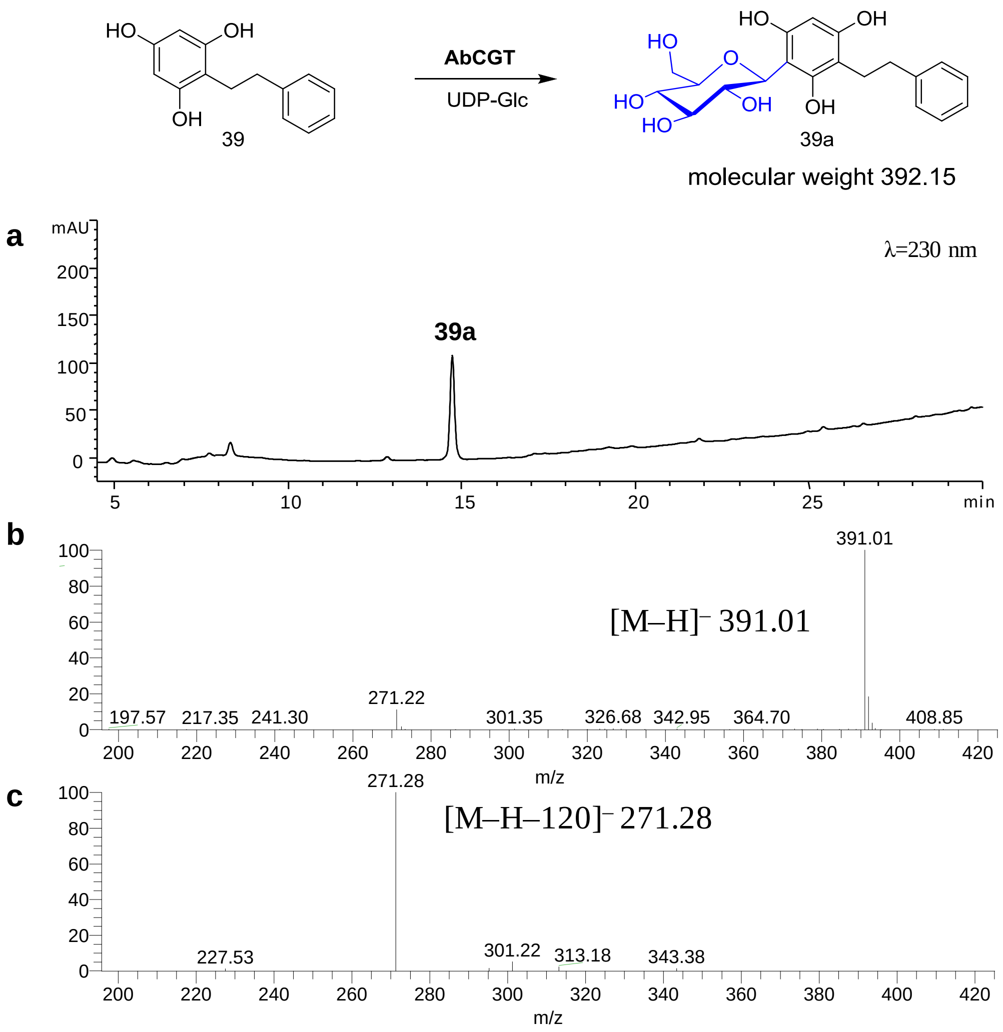

Supplementary Figure 49. HPLC-UV/ESI-MS analysis of AbCGT enzyme product using aglycon **39** and UDPG as substrates. a) HPLC-UV analysis of the AbCGT catalyzing reaction; b) Typical negative ion MS for the peak of **39a**; c) Typical negative MS^2 for peak of **39a**.

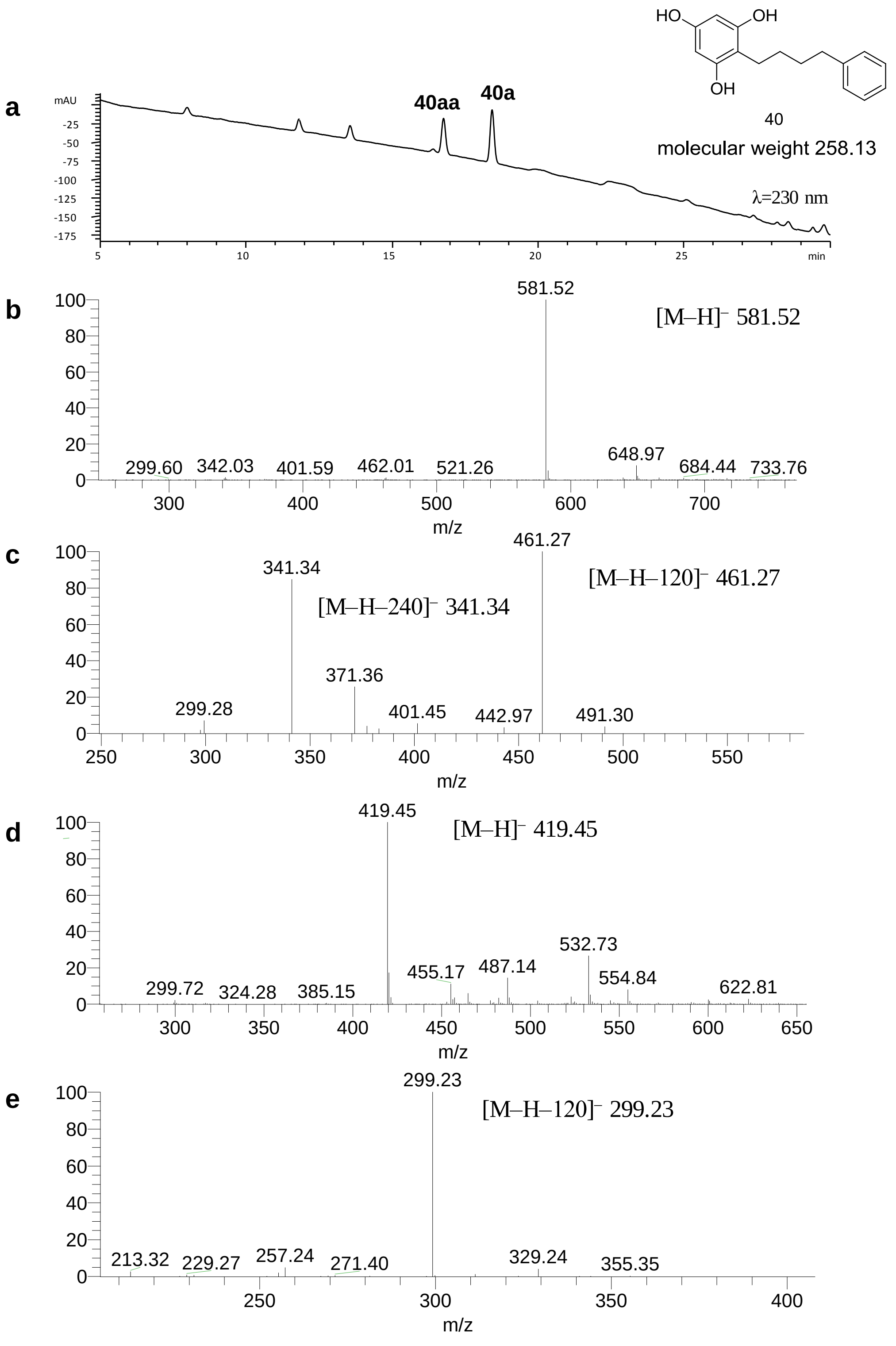

Supplementary Figure 50. HPLC-UV/ESI-MS analysis of AbCGT enzyme products using aglycon **40** and UDPG as substrates. a) HPLC-UV analysis of the AbCGT catalyzing reaction; b) Typical negative ion MS for the peak of **40aa**; c) Typical negative MS^2 for peak of **40aa**; d) Typical negative ion MS for the peak of **40a**; e) Typical negative MS^2 for peak of **40a**.

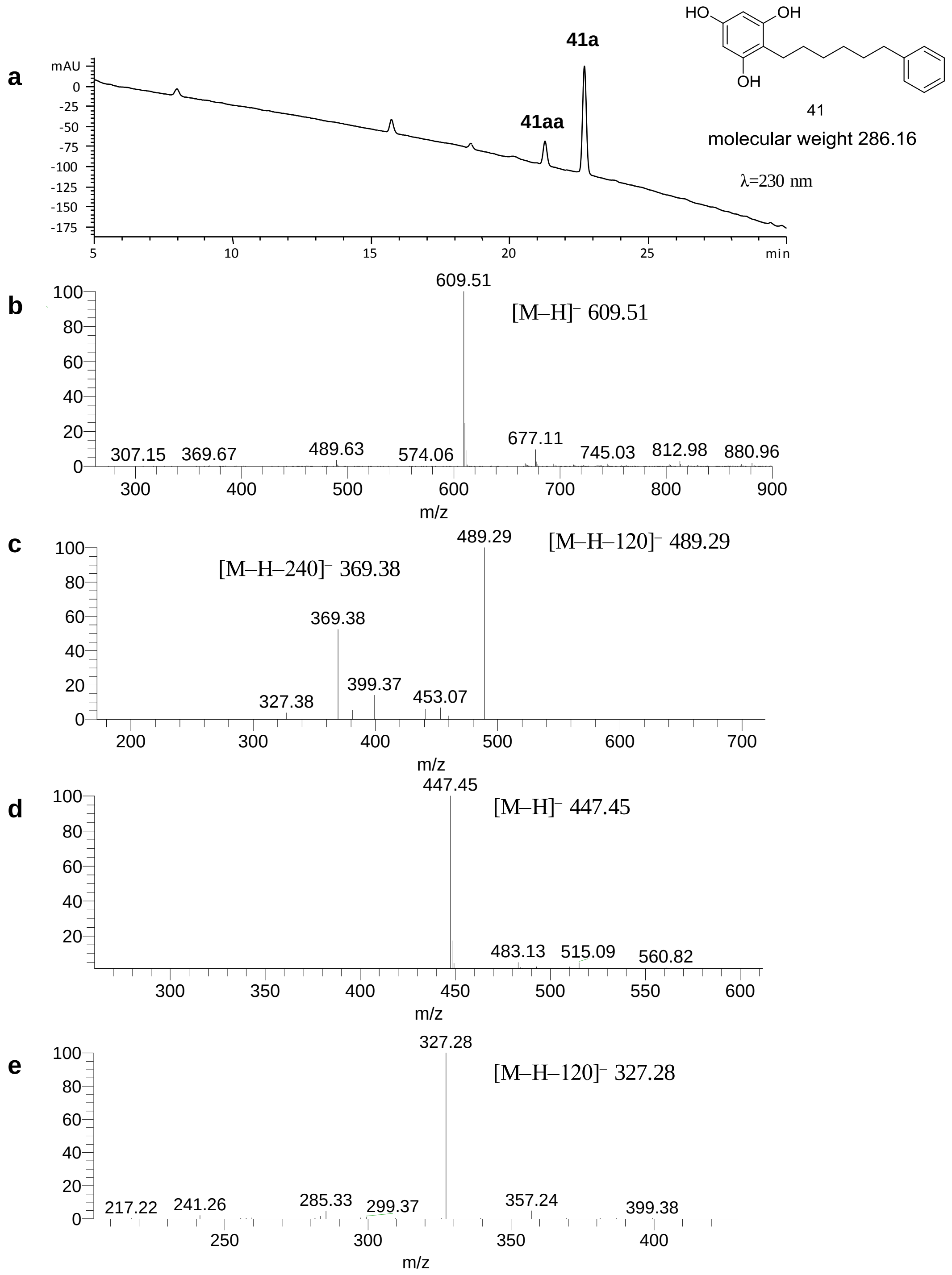

Supplementary Figure 51. HPLC-UV/ESI-MS analysis of AbCGT enzyme products using aglycon **41** and UDPG as substrates. a) HPLC-UV analysis of the AbCGT catalyzing reaction; b) Typical negative ion MS for the peak of **41aa**; c) Typical negative MS^2 for peak of **41aa**; d) Typical negative ion MS for the peak of **41a**; e) Typical negative MS^2 for peak of **41a**.

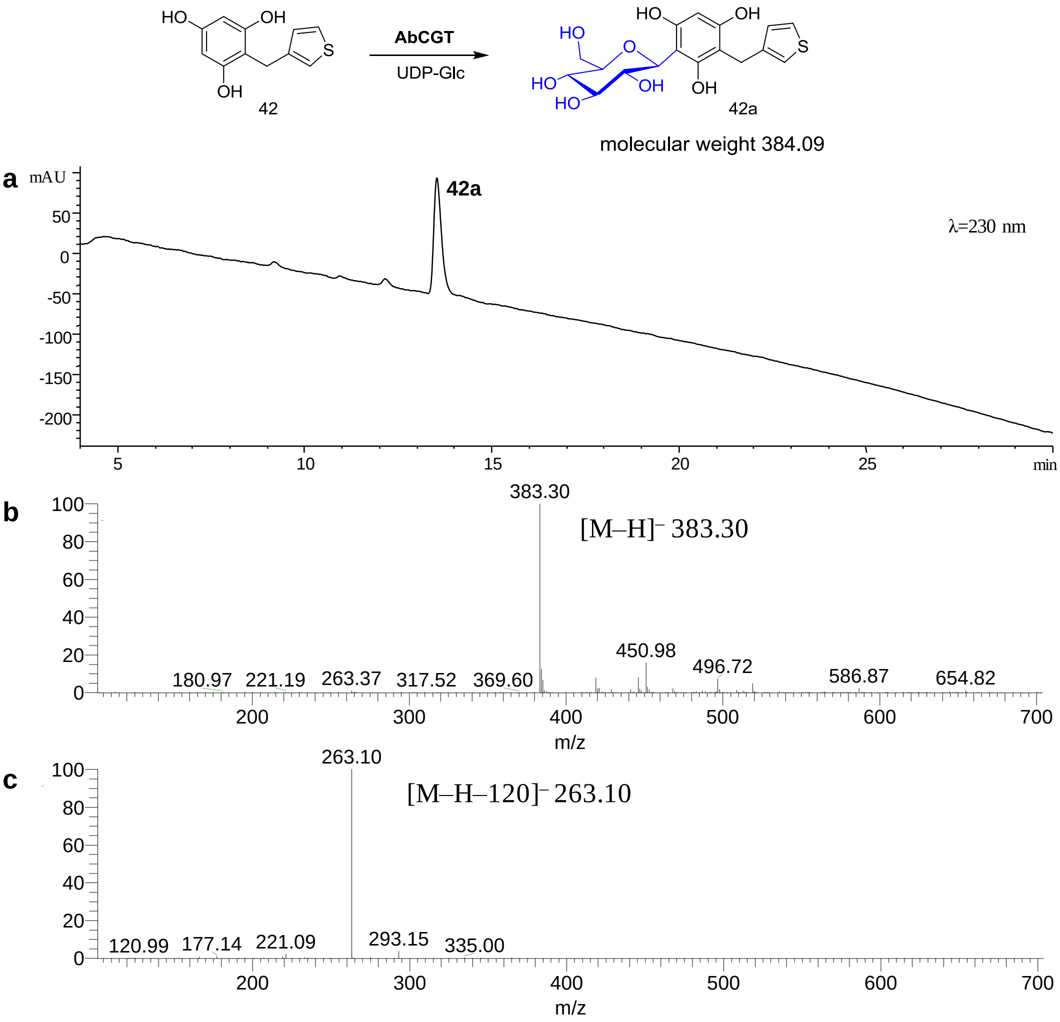

Supplementary Figure 52. HPLC-UV/ESI-MS analysis of AbCGT enzyme product using aglycon **42** and UDPG as substrates. a) HPLC-UV analysis of the AbCGT catalyzing reaction; b) Typical negative ion MS for the peak of **42a**; c) Typical negative MS^2 for peak of **42a**.

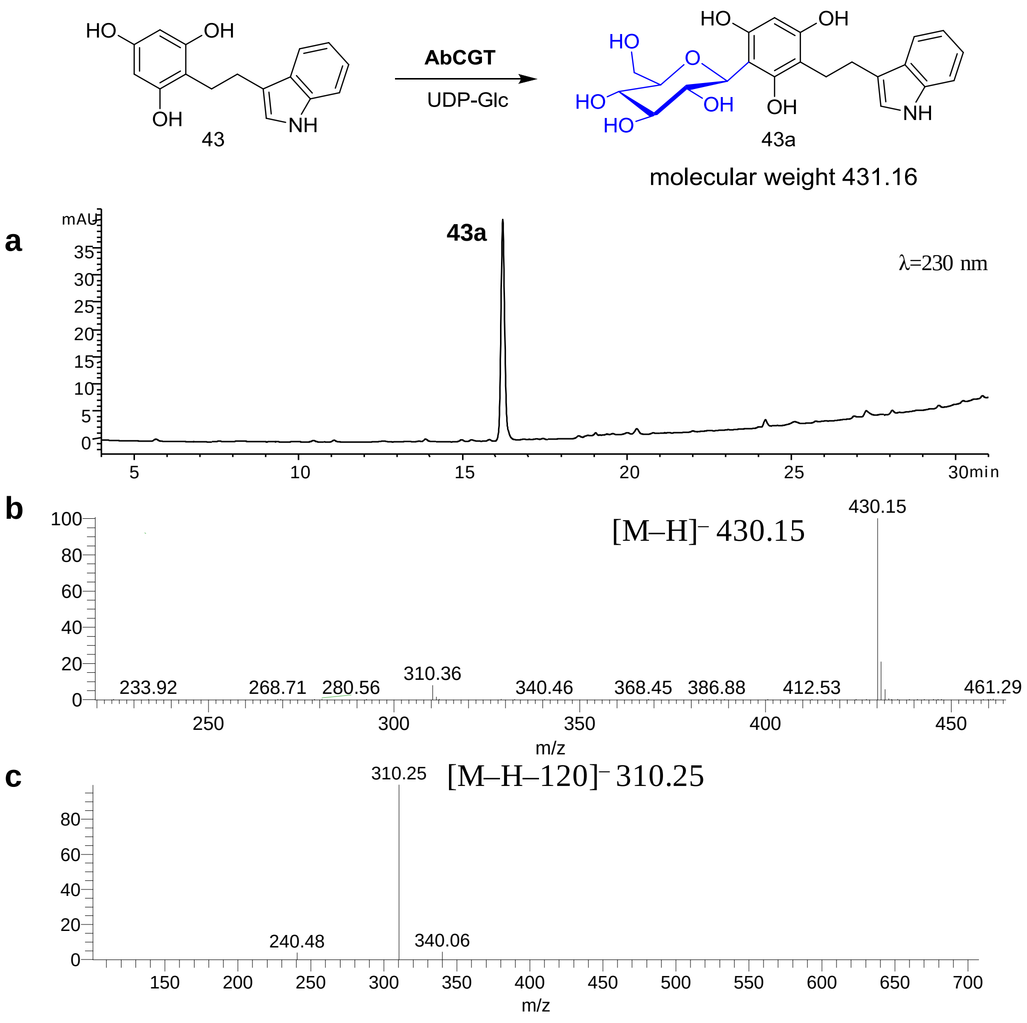

Supplementary Figure 53. HPLC-UV/ESI-MS analysis of AbCGT enzyme product using aglycon **43** and UDPG as substrates. a) HPLC-UV analysis of the AbCGT catalyzing reaction; b) Typical negative ion MS for the peak of **43a**; c) Typical negative MS^2 for peak of **43a**.

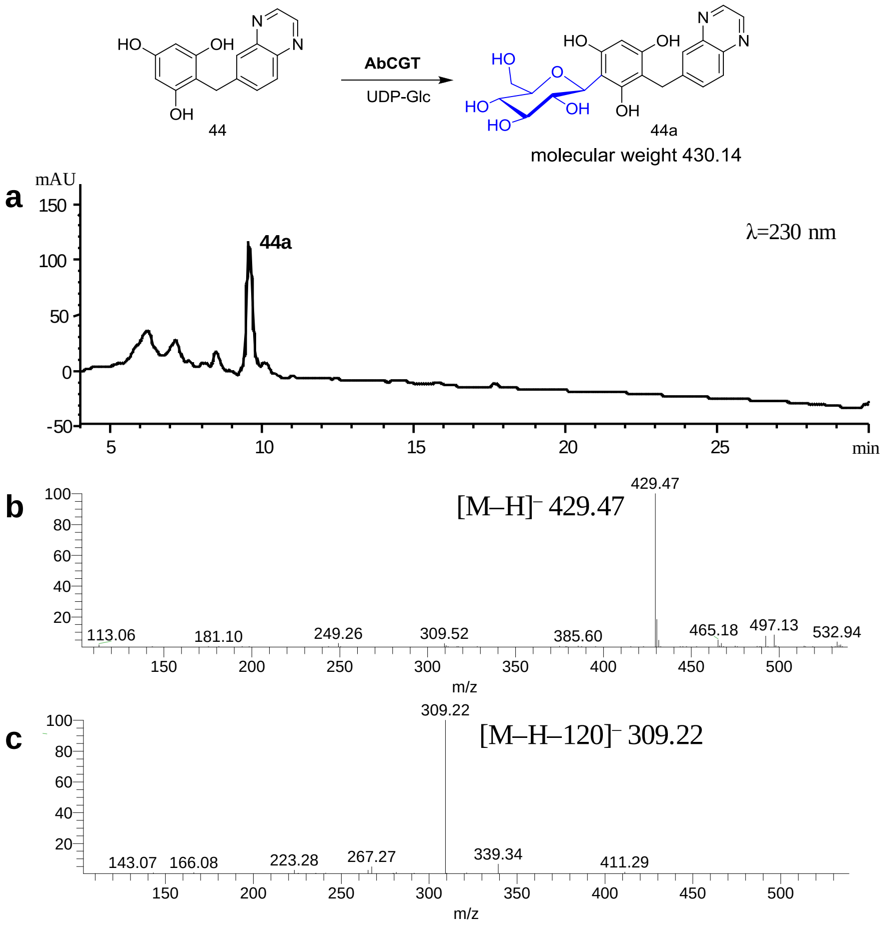

Supplementary Figure 54. HPLC-UV/ESI-MS analysis of AbCGT enzyme product using aglycon **44** and UDPG as substrates. a) HPLC-UV analysis of the AbCGT catalyzing reaction; b) Typical negative ion MS for the peak of **44a**; c) Typical negative MS^2 for peak of **44a**.

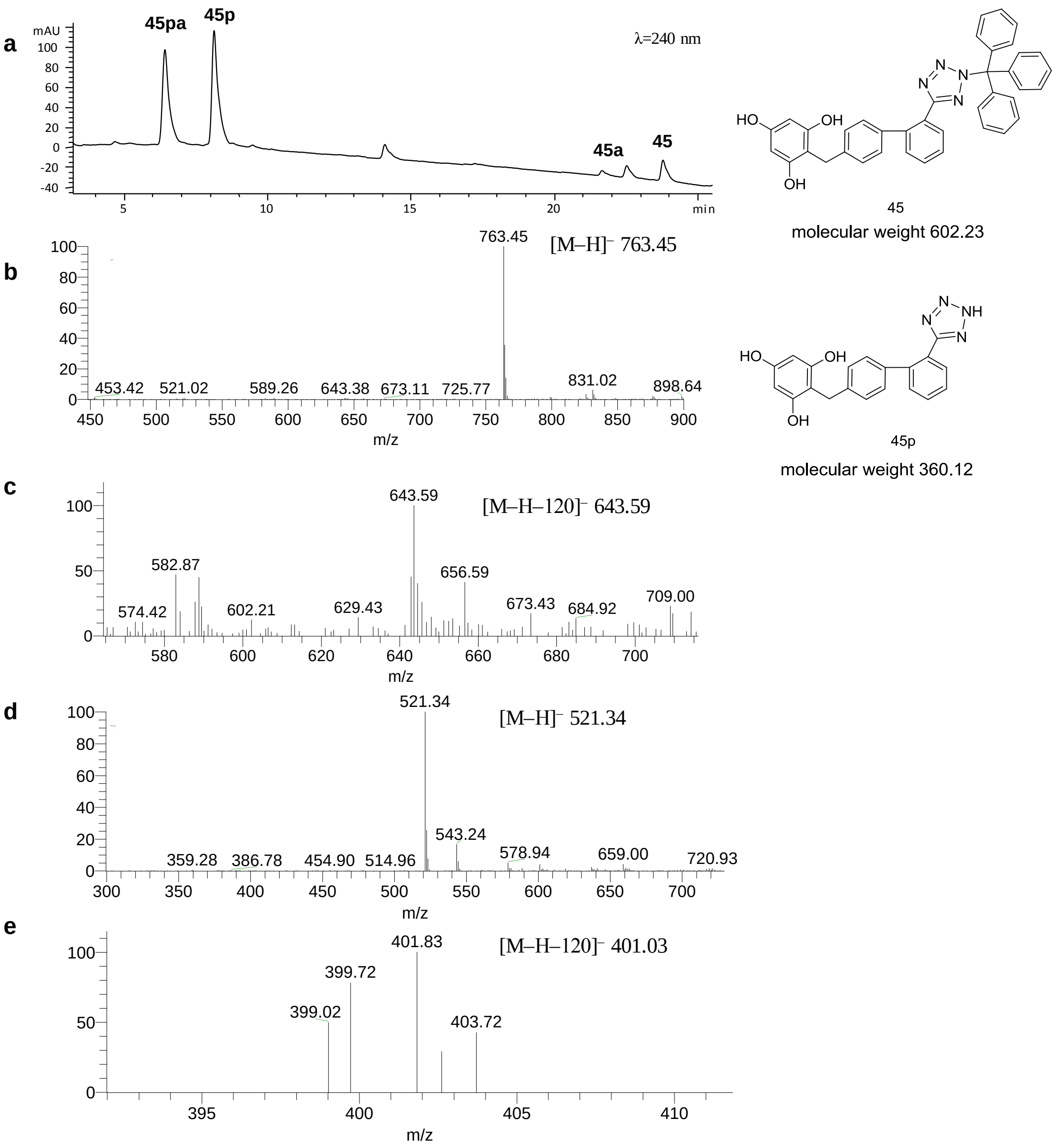

Supplementary Figure 55. HPLC-UV/ESI-MS analysis of AbCGT enzyme products using aglycon **45** and UDPG as substrates. a) HPLC-UV analysis of the AbCGT catalyzing reaction; b) Typical negative ion MS for the peak of **45a**; c) Typical negative MS^2 for peak of **45a**; d) Typical negative ion MS for the peak of **45pa**; e) Typical negative MS^2 for peak of **45pa**.

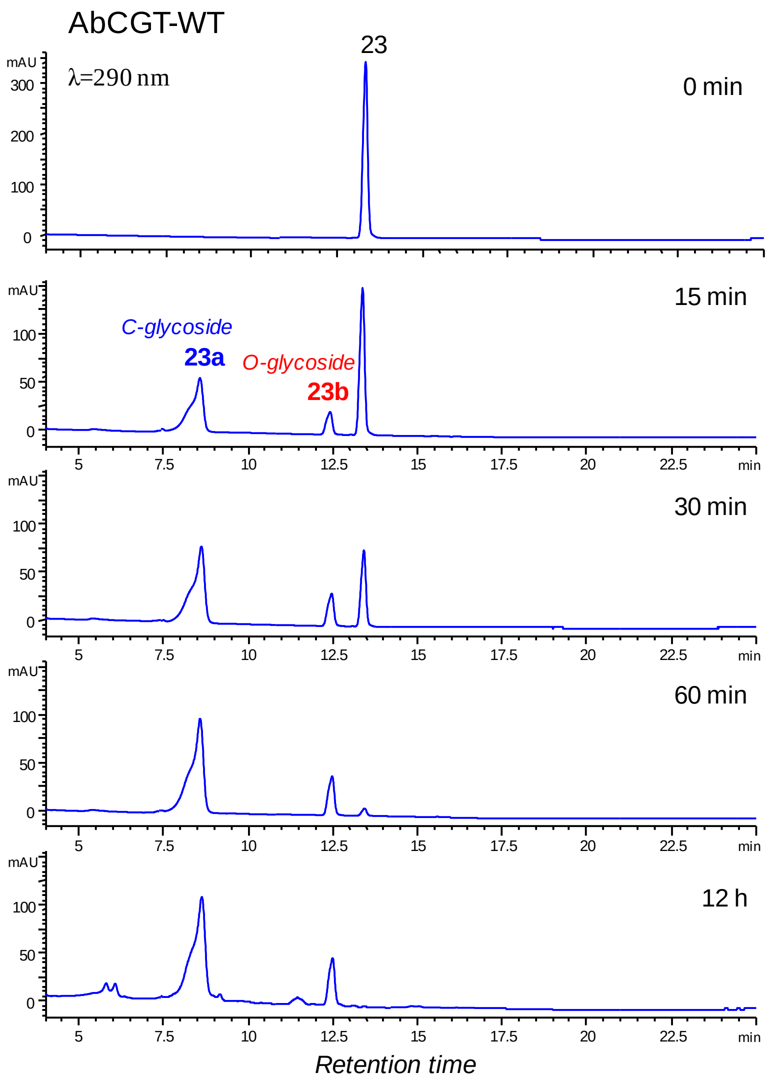

Supplementary Figure 56. HPLC analysis of reactions catalyzed by wild type AbCGT at different time intervals. 2-hydroxynaringenin (**23**) was used as the acceptor and UDP-Glc was used as the sugar donor. Reactions were performed at pH 7.4 and 30 ℃.

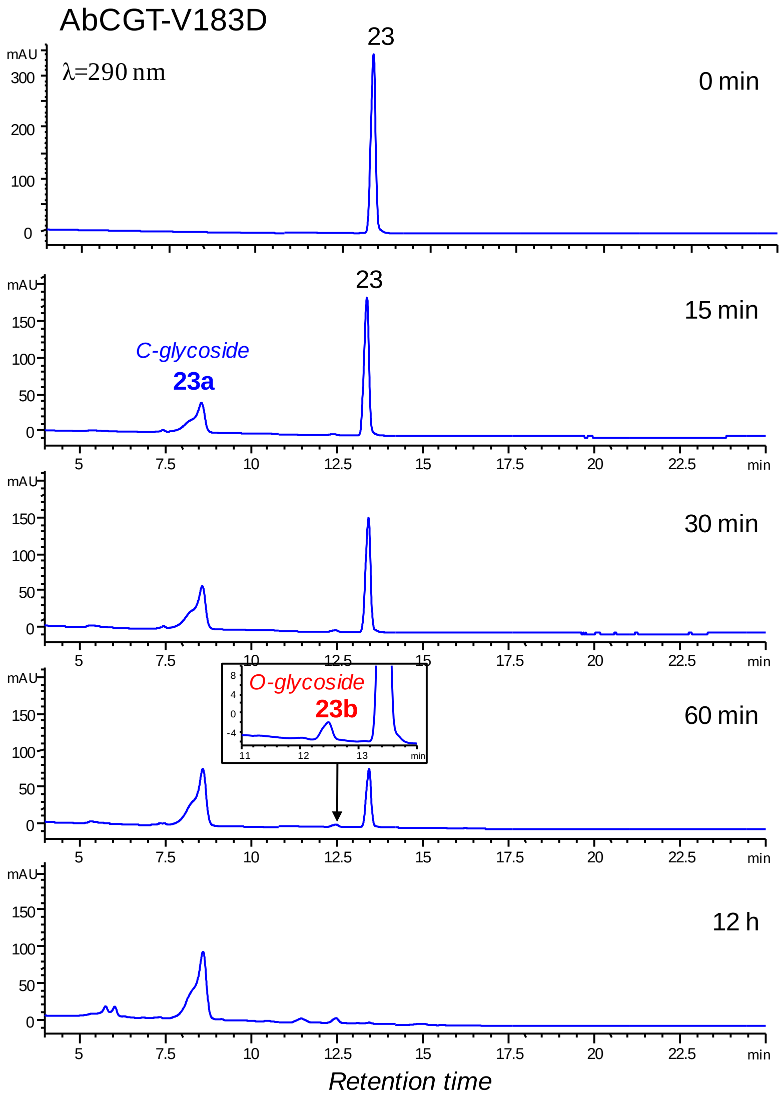

Supplementary Figure 57. HPLC analysis of reactions catalyzed by the mutant V183D-AbCGT at different time intervals. 2-hydroxynaringenin (**23**) was used as the acceptor and UDP-Glc was used as the sugar donor. Reactions were performed at pH 7.4 and 30 ℃. (The *O*-glycoside was greatly decreased and only <1% of *O*-glycoside was observed.)

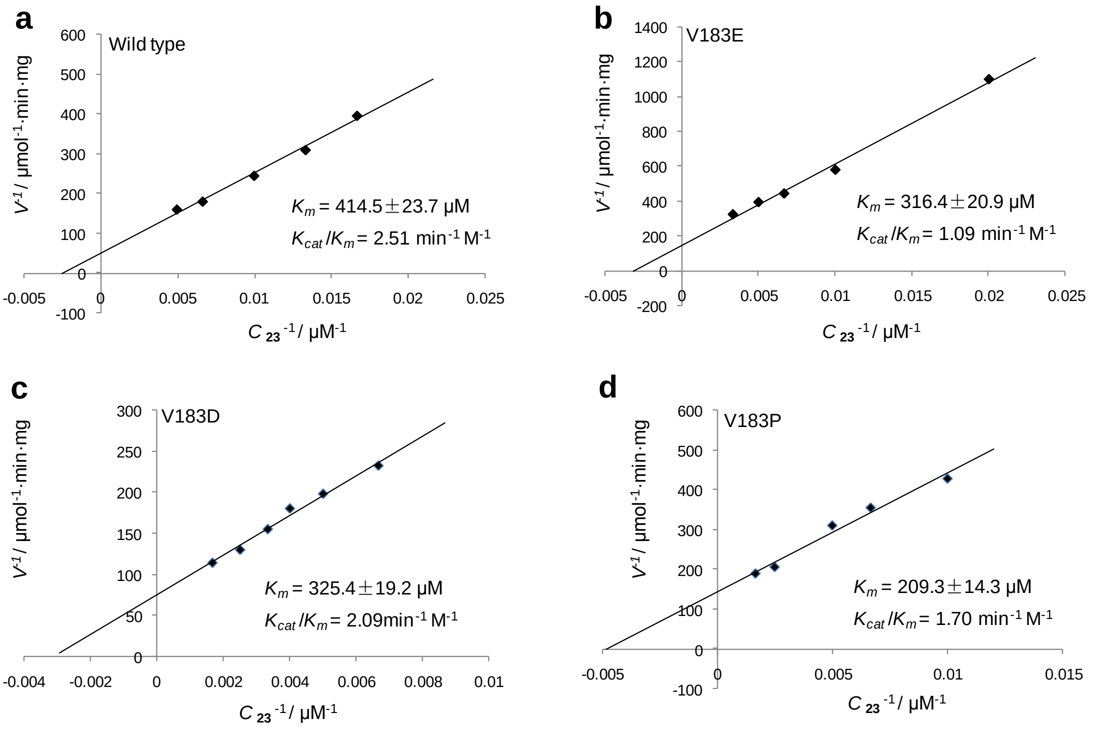

Supplementary Figure 58. The kinetic parameters of wild type AbCGT and V183E, V183D and V183P for 2-hydroxynaringenin (**23**). a) The kinetic parameters of wild type AbCGT; b) The kinetic parameters of V183E; c)The kinetic parameters of V183D; d)The kinetic parameters of V183P; UDP-Glc was used as the sugar donor.

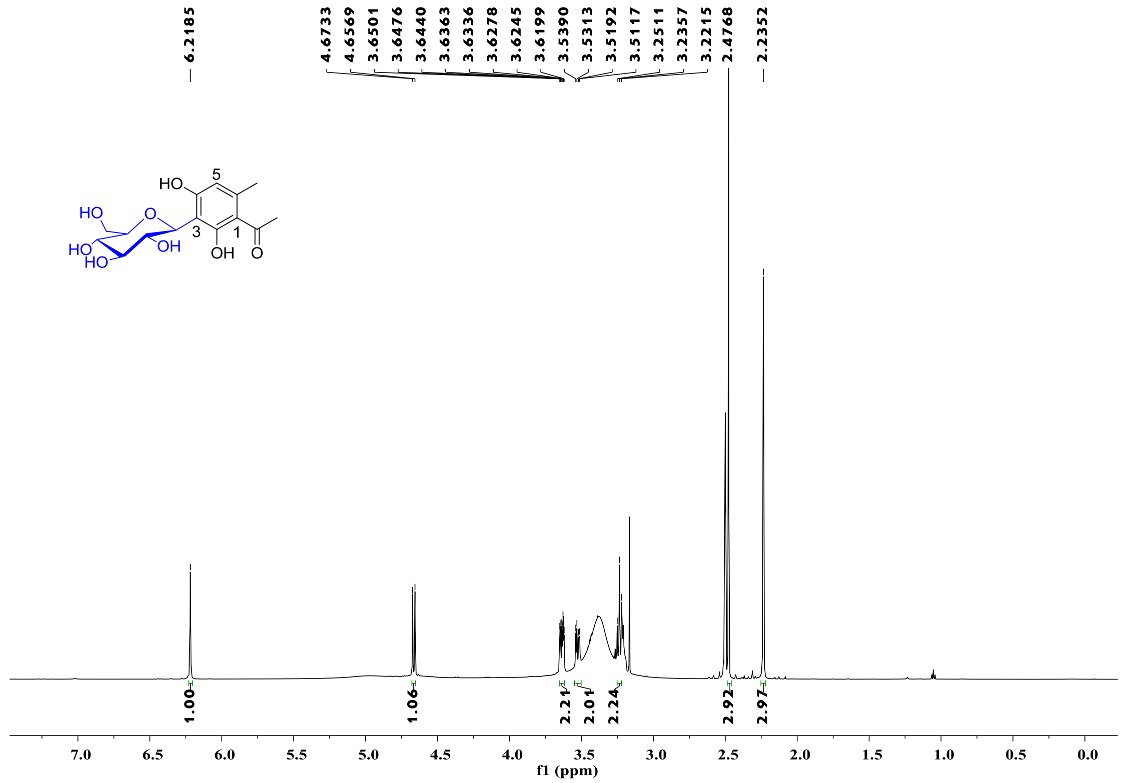

Supplementary Figure 59 ^{1}H NMR spectrum of **9a** (DMSO-d_6, 600 MHz)

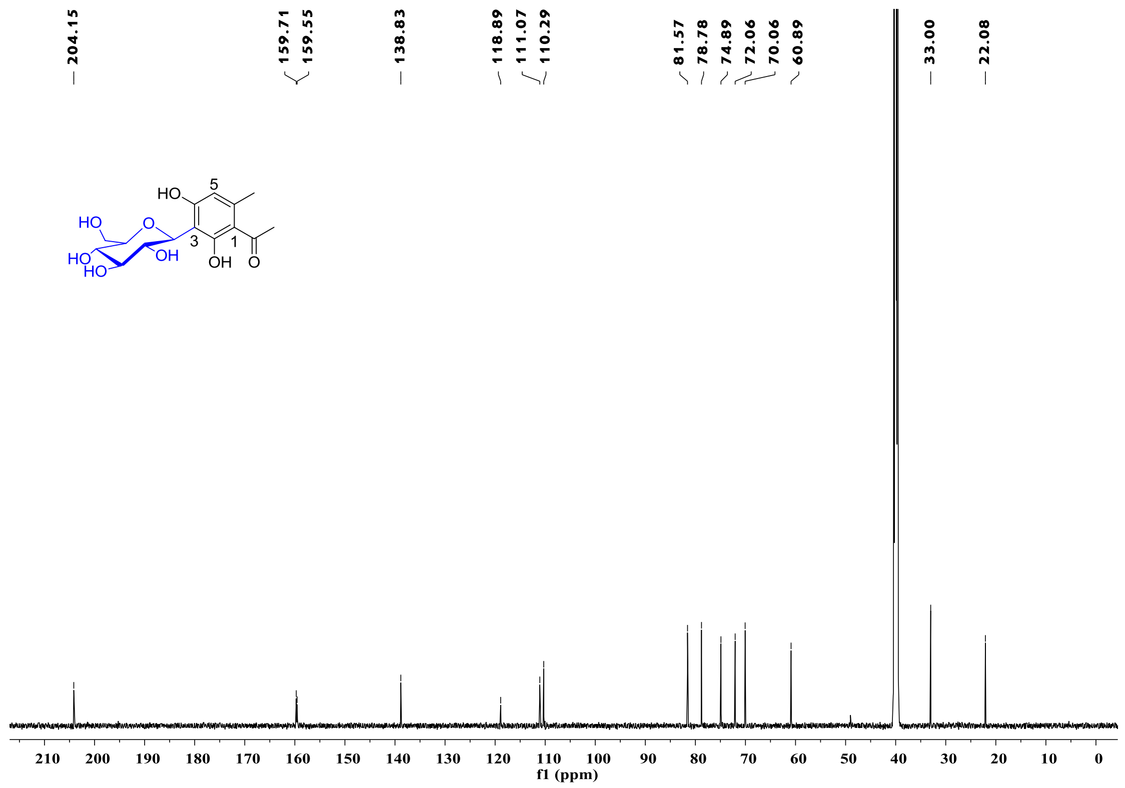

Supplementary Figure 60 ^{13}C NMR spectrum of **9a** (DMSO-d_6, 150 MHz)

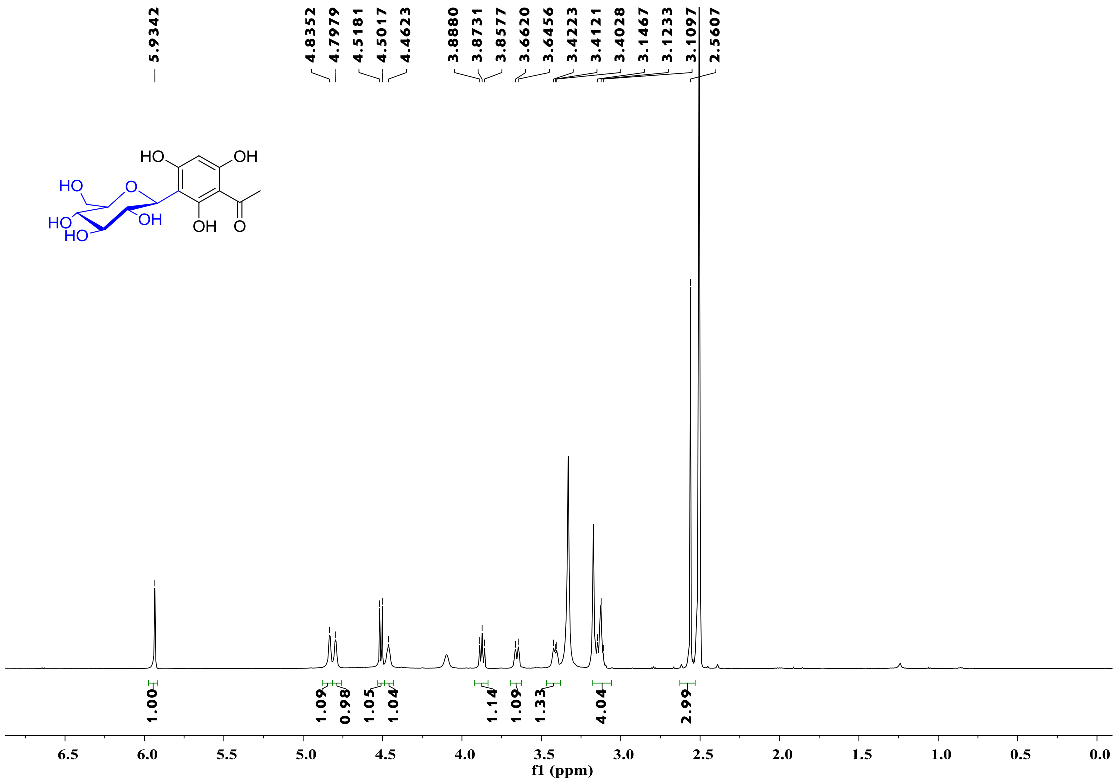

Supplementary Figure 61 ^{1}H NMR spectrum of **11a** (DMSO-d_6, 600 MHz)

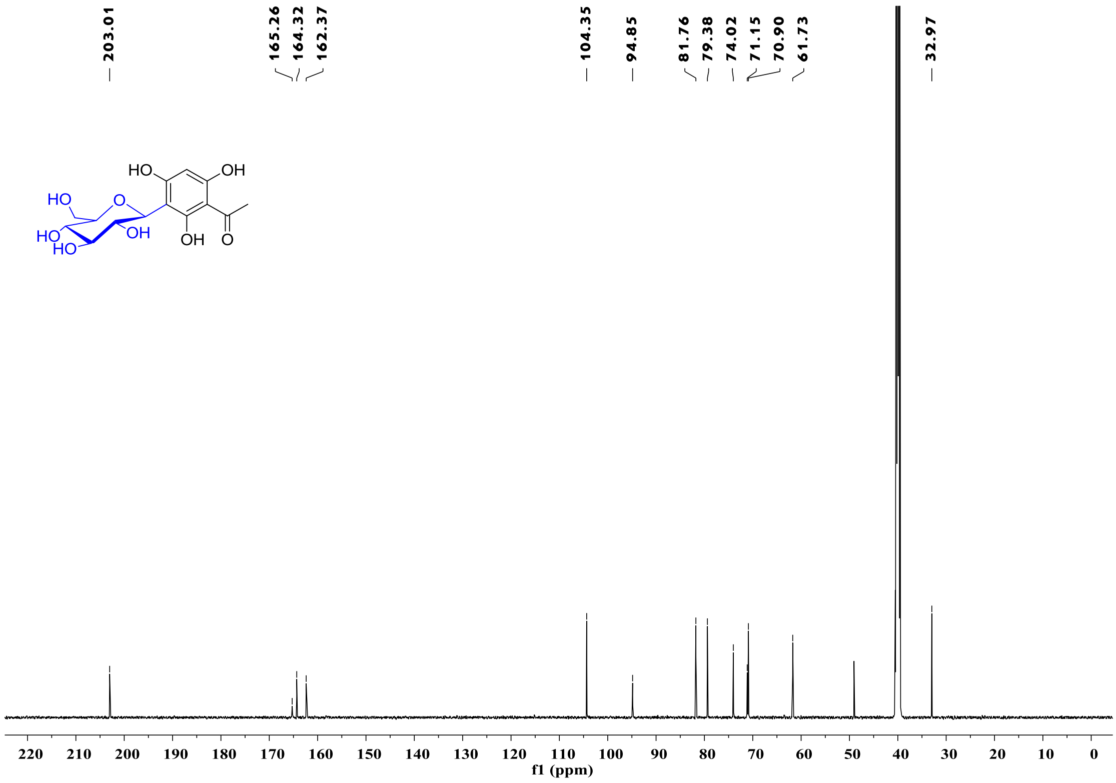

Supplementary Figure 62 ^{13}C NMR spectrum of **11a** (DMSO-d_6, 150 MHz)

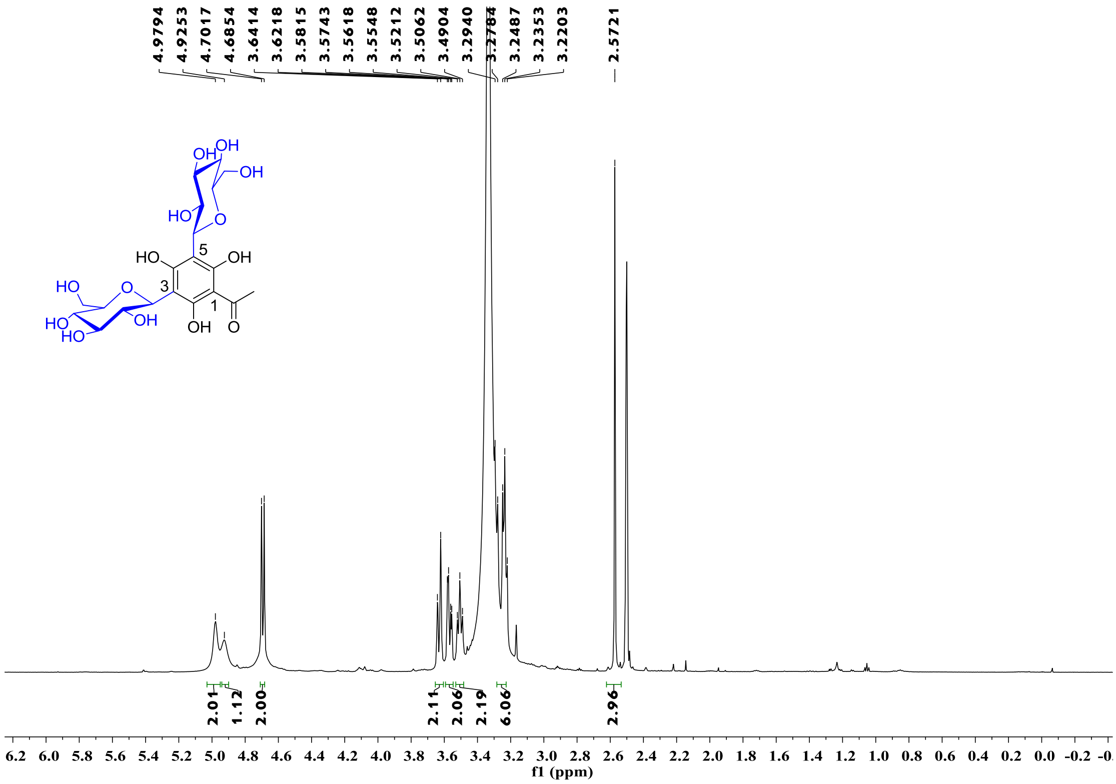

Supplementary Figure 63 ^{1}H NMR spectrum of **11aa** (DMSO-d_6, 600 MHz)

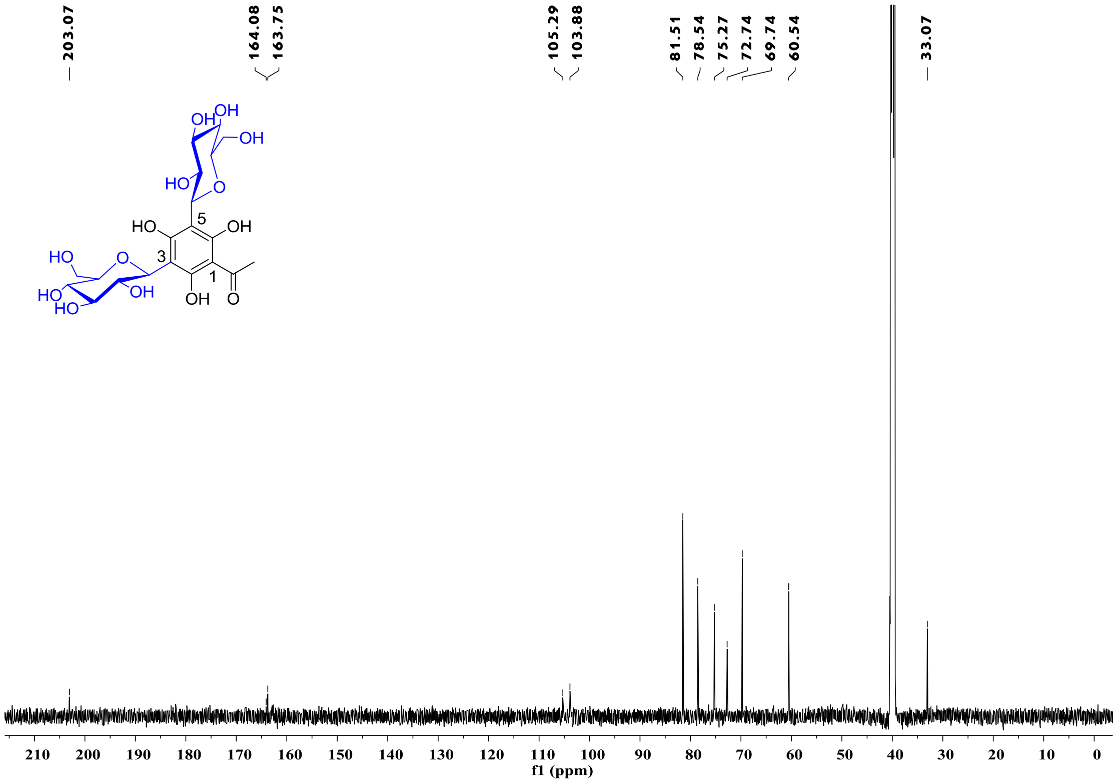

Supplementary Figure 64 ^{13}C NMR spectrum of **11aa** (DMSO-d_6, 150 MHz)

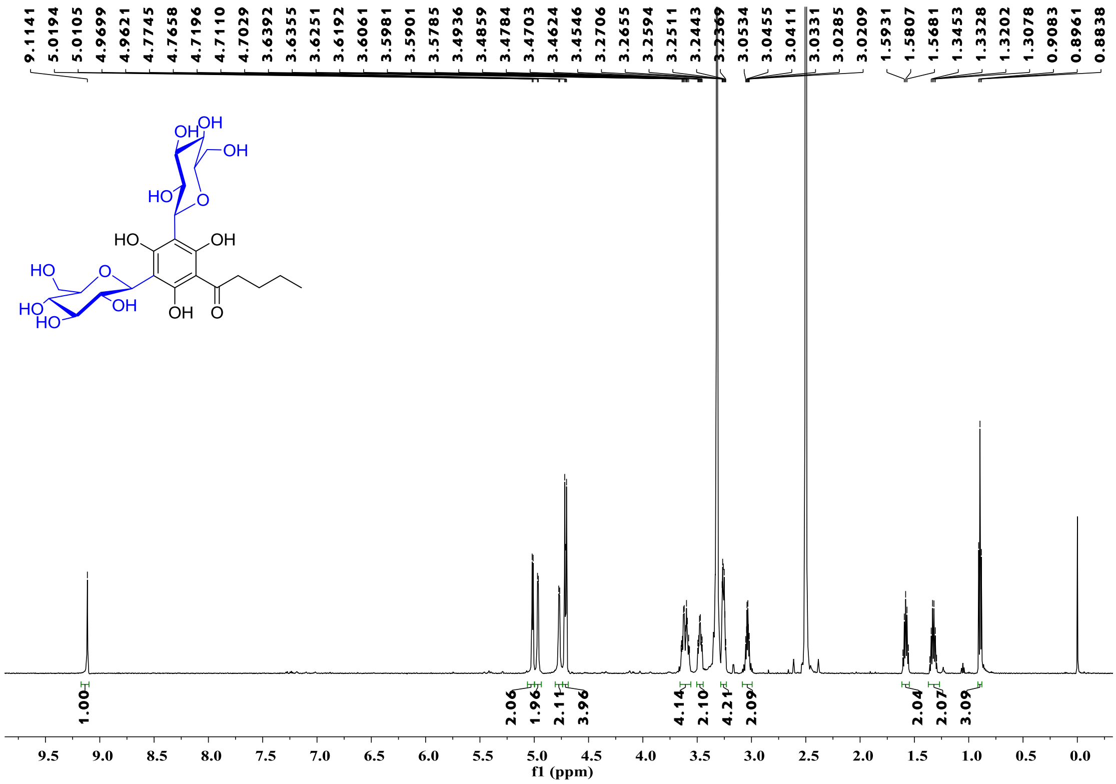

Supplementary Figure 65 ^{1}H NMR spectrum of **16aa** (DMSO-d_6, 600 MHz)

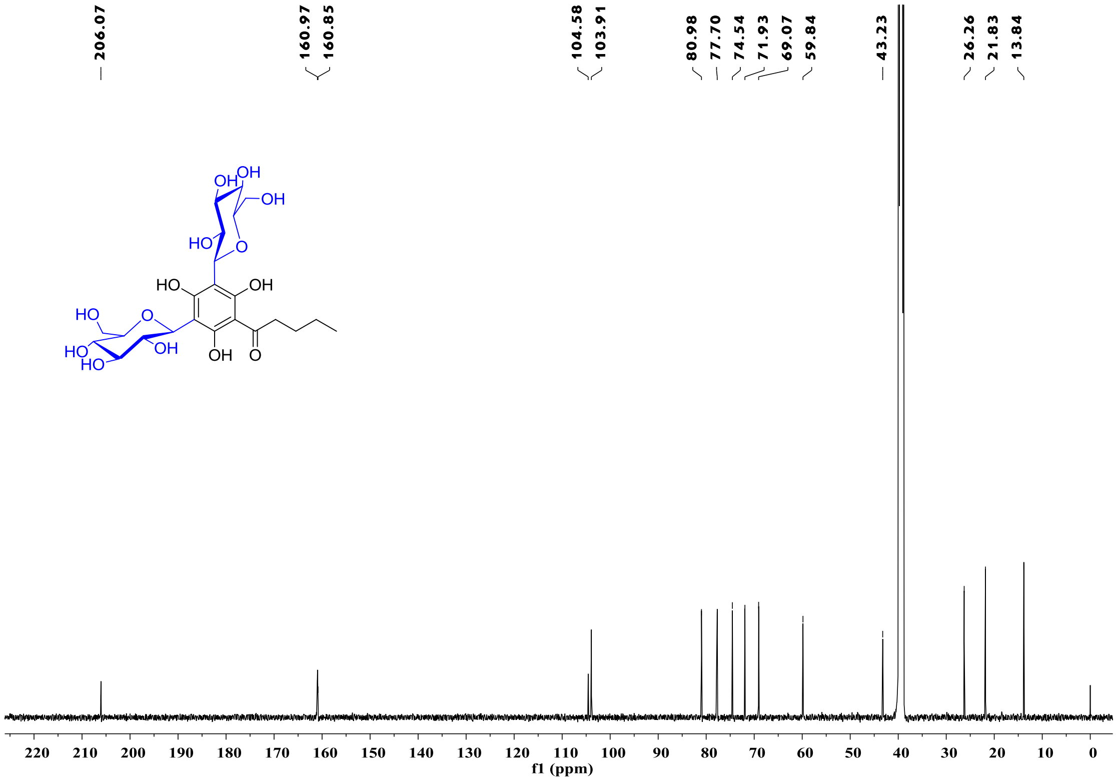

Supplementary Figure 66 ^{13}C NMR spectrum of **16aa** (DMSO-d_6, 150 MHz)

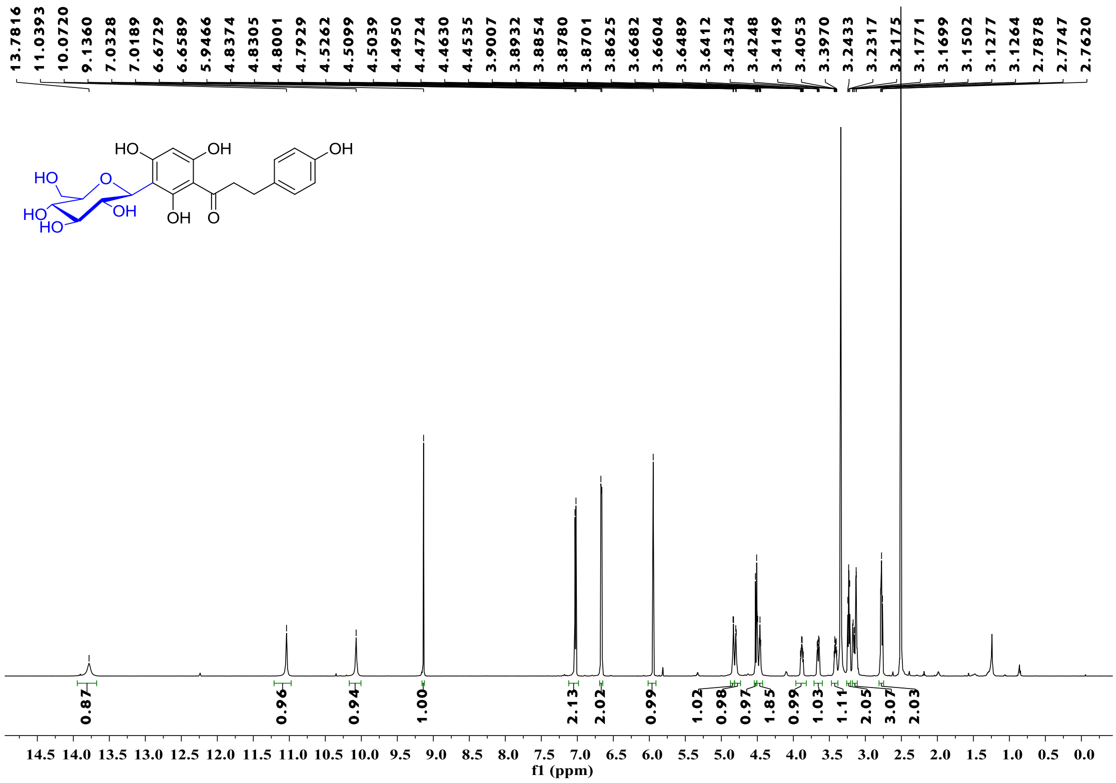

Supplementary Figure 67 ^{1}H NMR spectrum of **17a** (DMSO-d_6, 600 MHz)

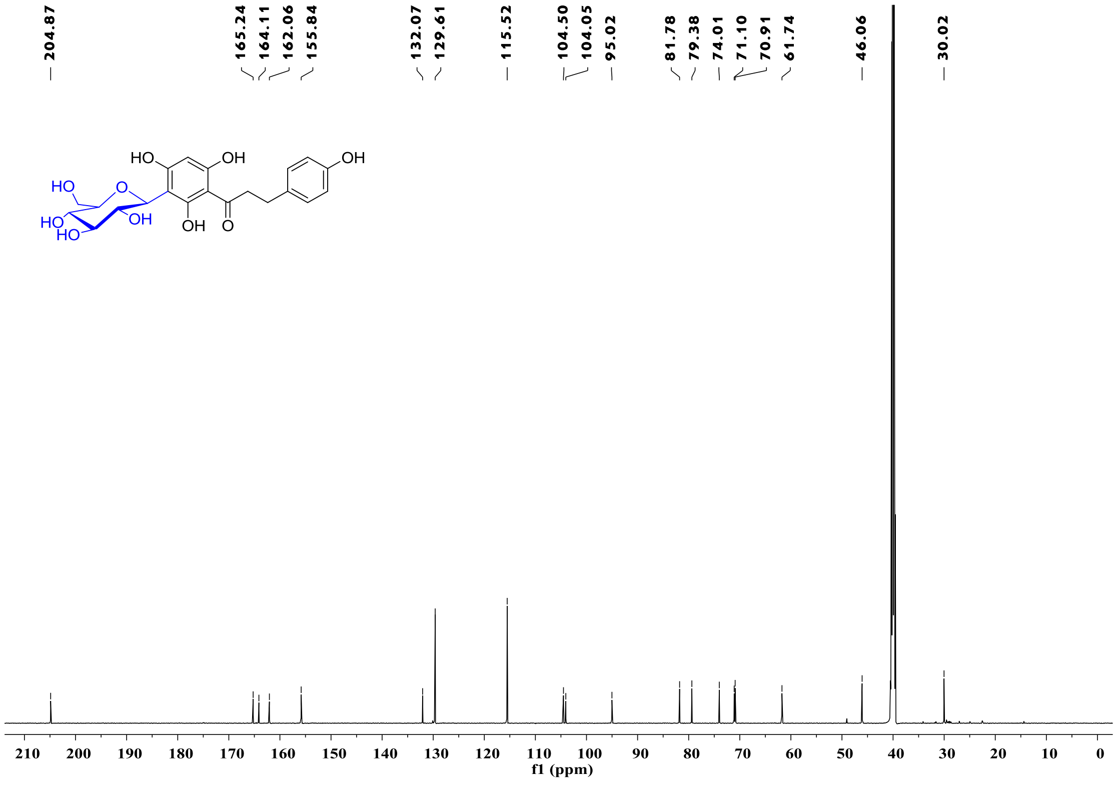

Supplementary Figure 68 ^{13}C NMR spectrum of **17a** (DMSO-d_6, 150 MHz)

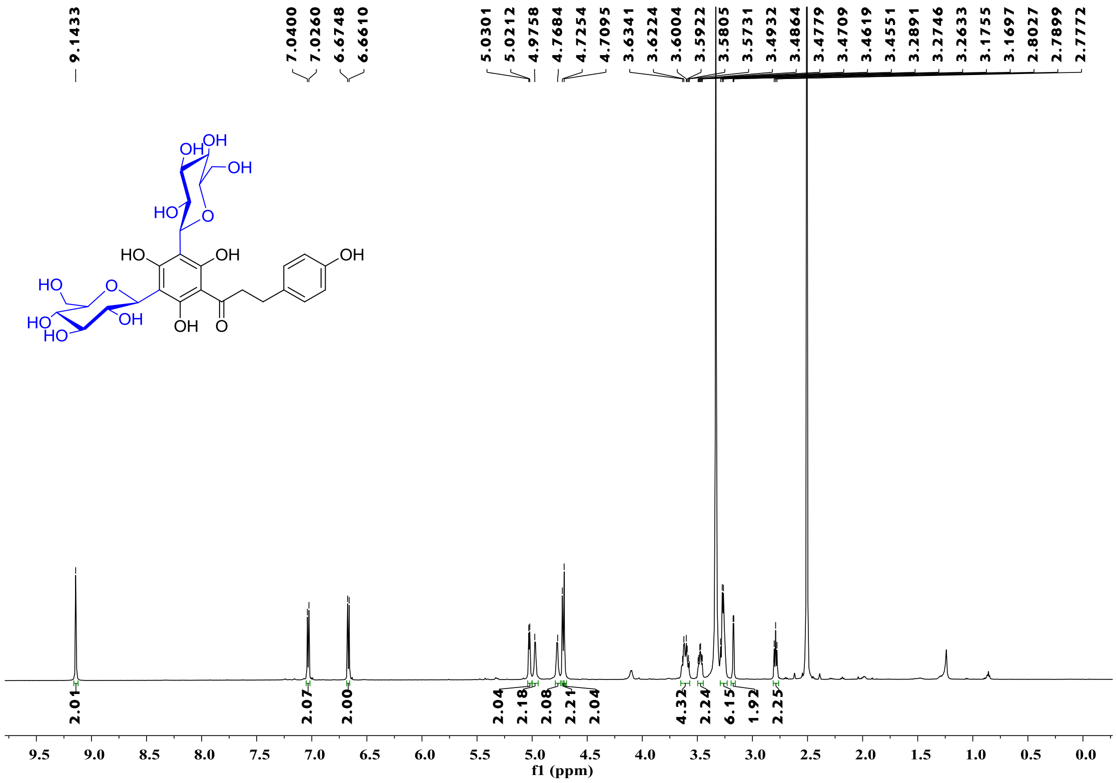

Supplementary Figure 69 ^{1}H NMR spectrum of **17aa** (DMSO-d_6, 600 MHz)

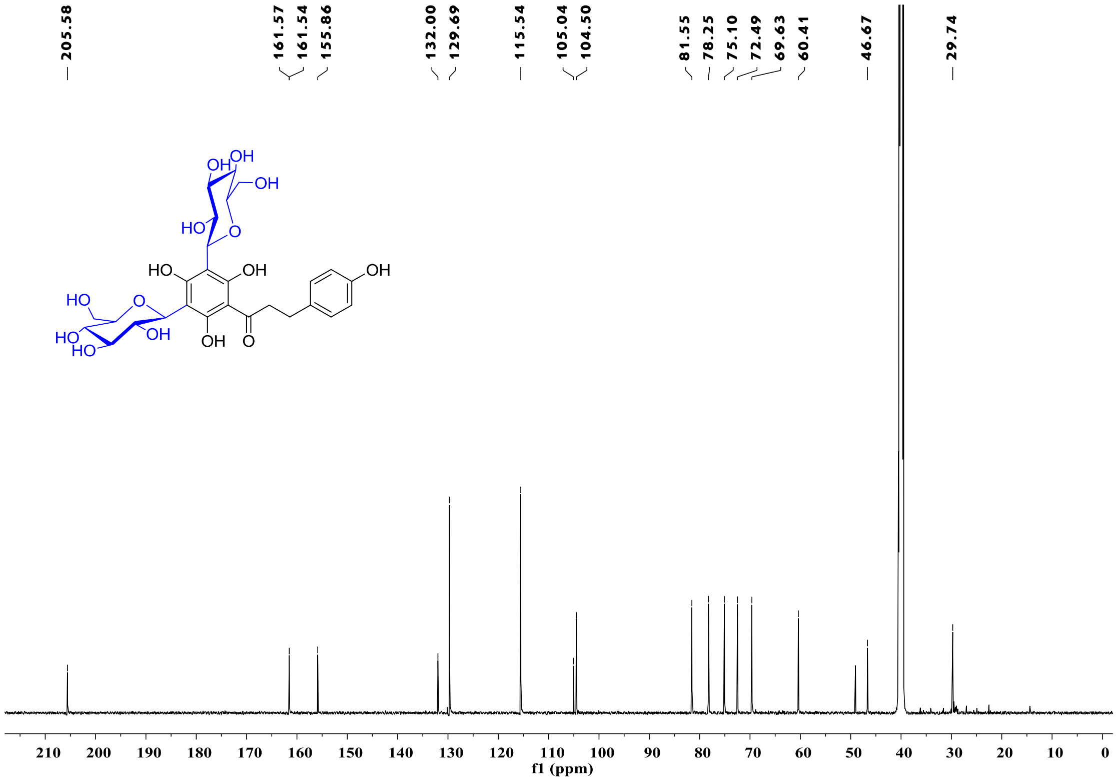

Supplementary Figure 70 ^{13}C NMR spectrum of 1**7aa** (DMSO-d_6, 150 MHz)

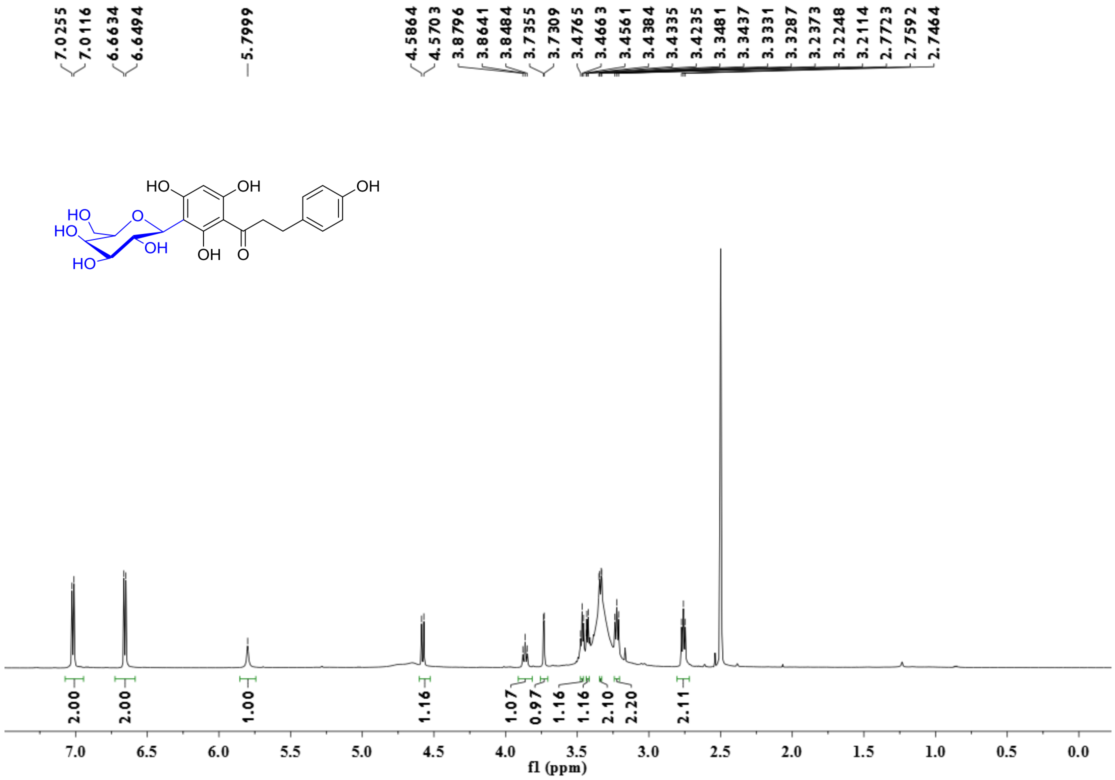

Supplementary Figure 71 ^{1}H NMR spectrum of **17b** (DMSO-d_6, 600 MHz)

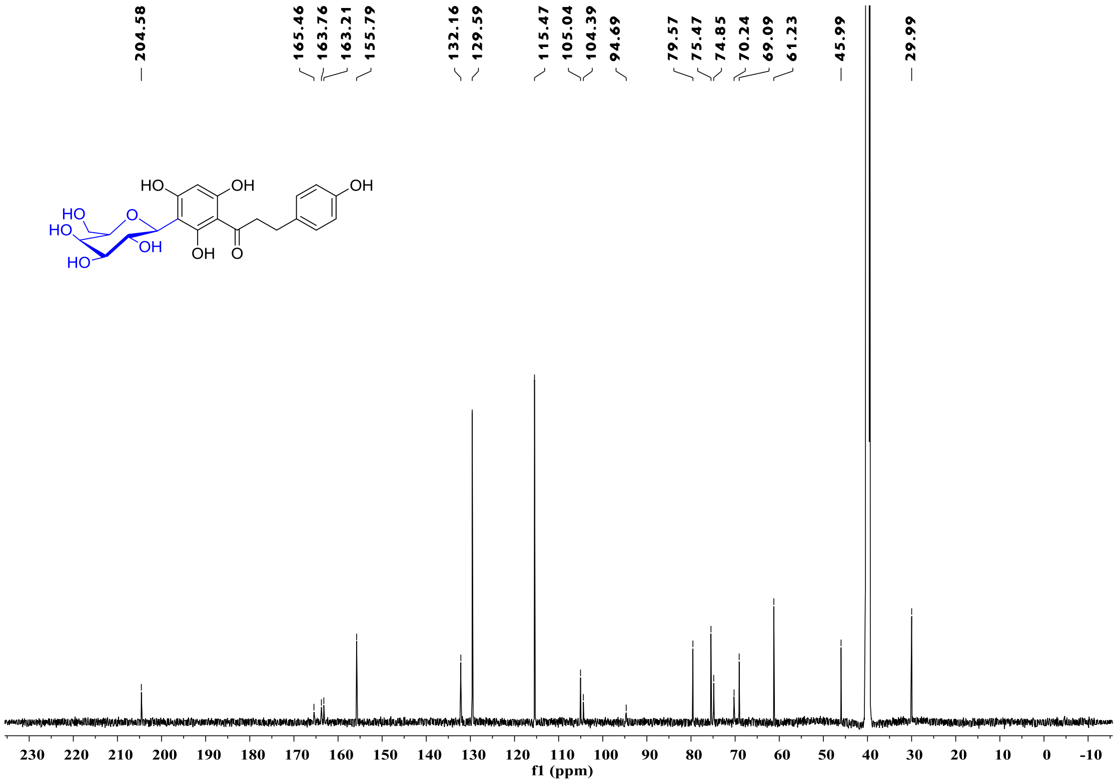

Supplementary Figure 72 ^{13}C NMR spectrum of 1**7b** (DMSO-d_6, 150 MHz)

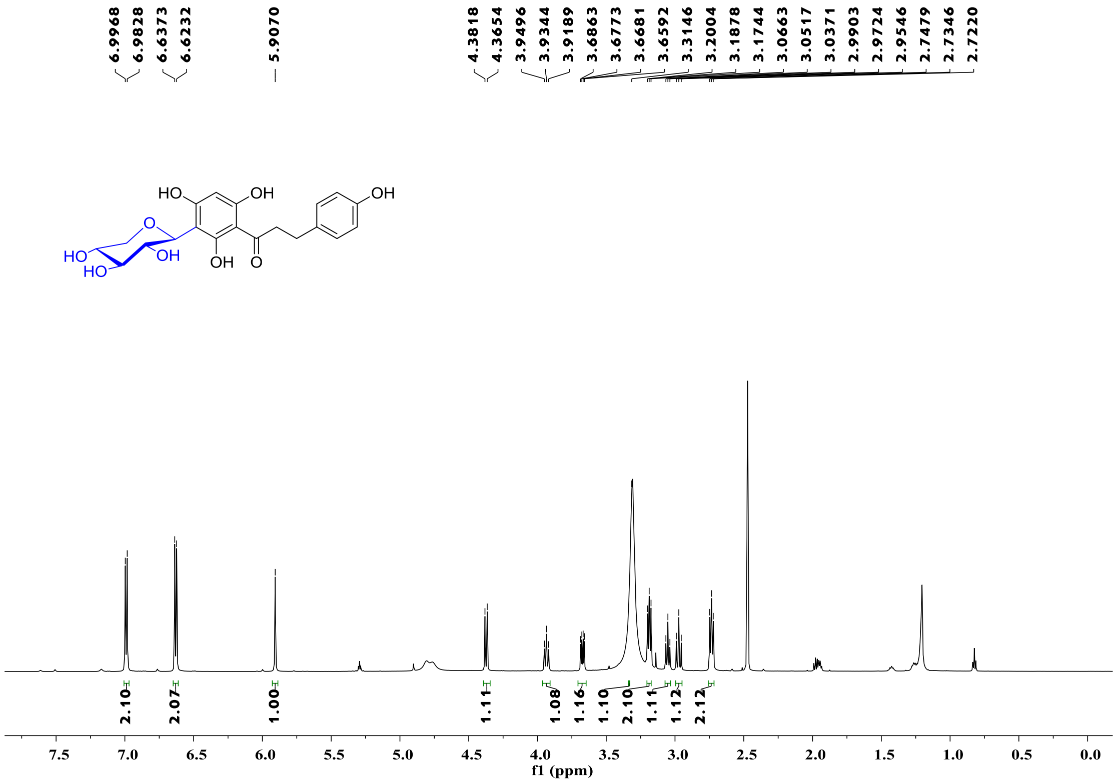

Supplementary Figure 73 ^{1}H NMR spectrum of **17c** (DMSO-d_6, 600 MHz)

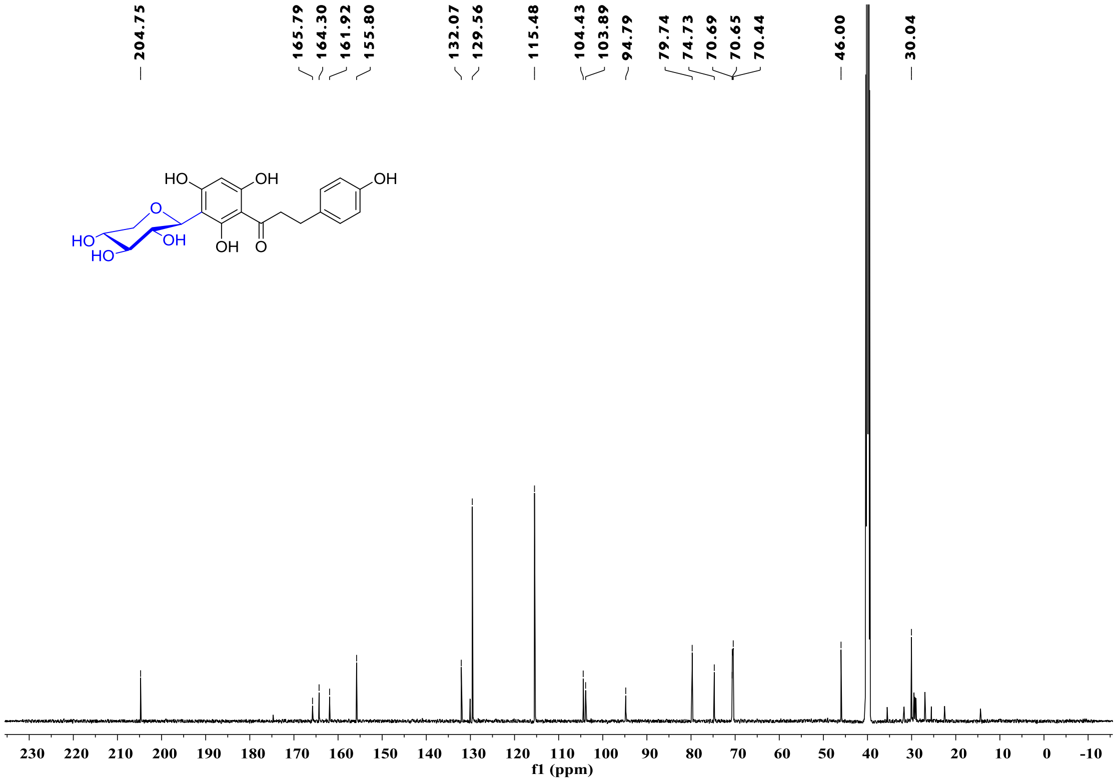

Supplementary Figure 74 ^{13}C NMR spectrum of 1**7c** (DMSO-d_6, 150 MHz)

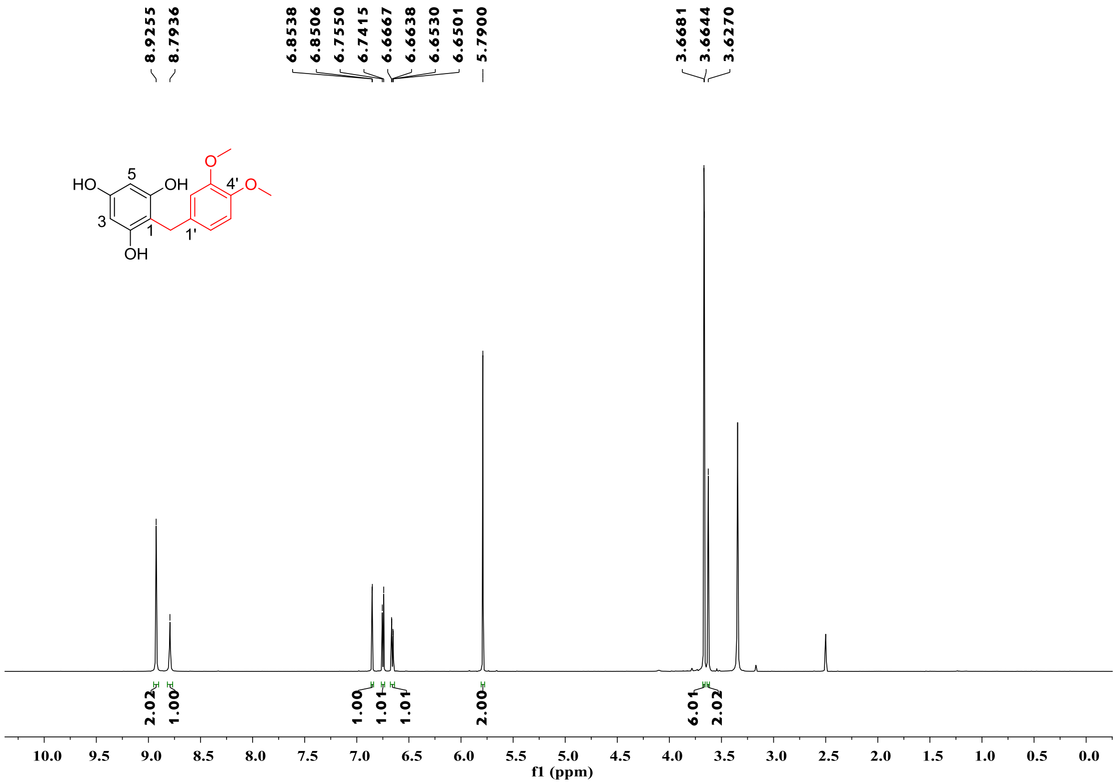

Supplementary Figure 75 ^{1}H NMR spectrum of **20** (DMSO-d_6, 600 MHz)

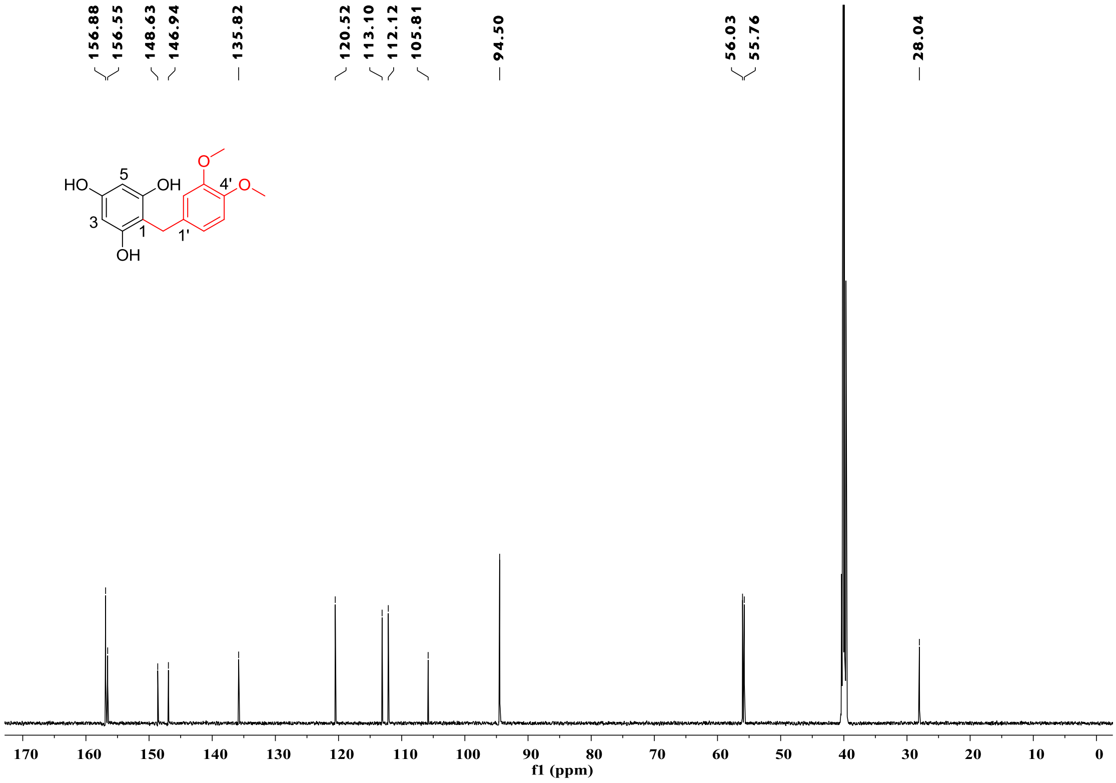

Supplementary Figure 76 ^{13}C NMR spectrum of **20** (DMSO-d_6, 150 MHz)

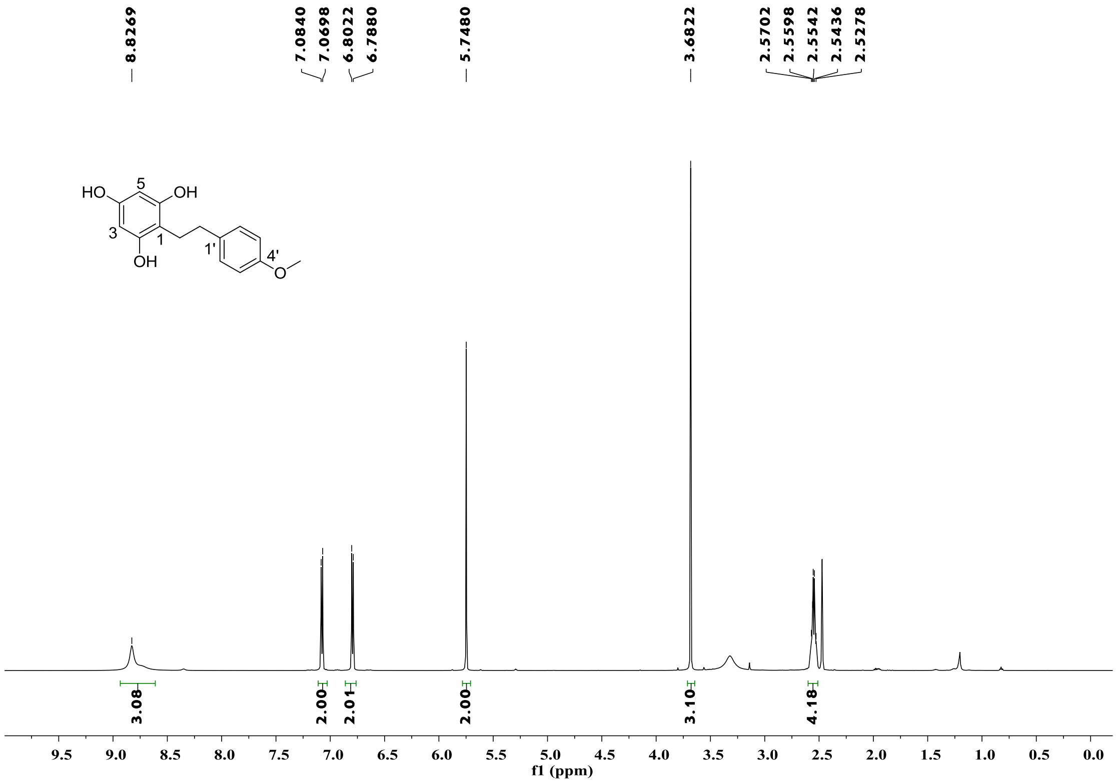

Supplementary Figure 77 ^{1}H NMR spectrum of **21** (DMSO-d_6, 600 MHz)

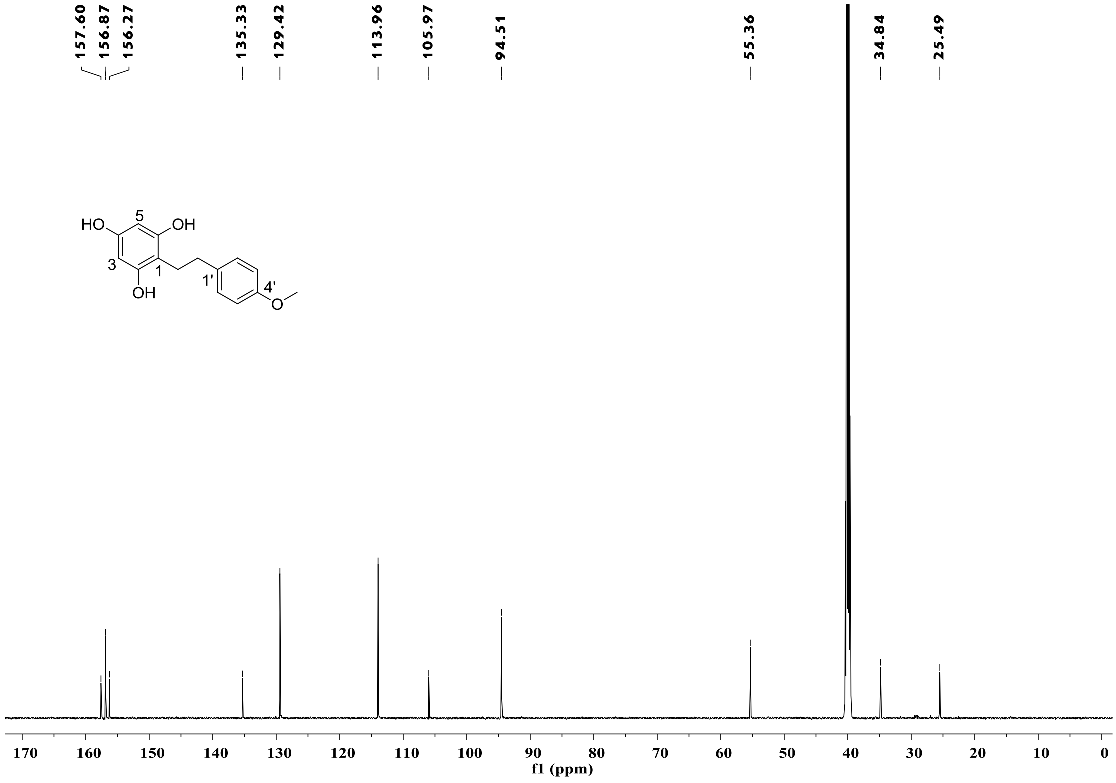

Supplementary Figure 78 ^{13}C NMR spectrum of **21** (DMSO-d_6, 150 MHz)

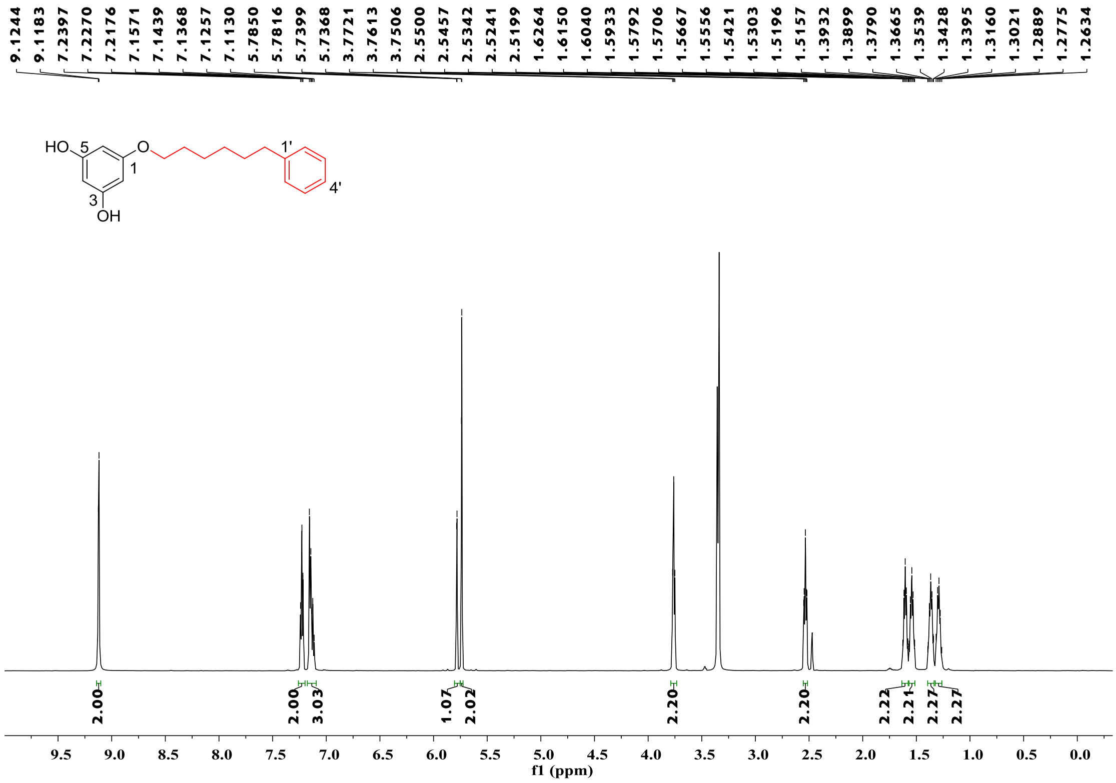

Supplementary Figure 79 ^{1}H NMR spectrum of **22** (DMSO-d_6, 600 MHz)

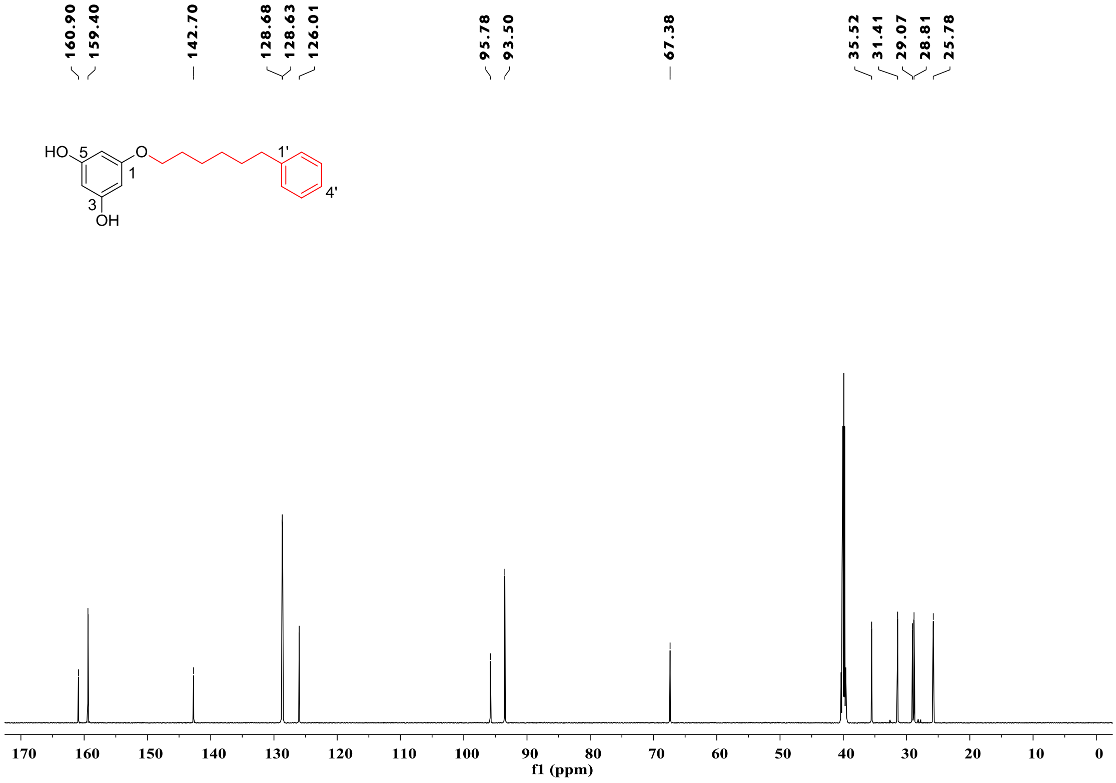

Supplementary Figure 80 ^{13}C NMR spectrum of **22** (DMSO-d_6, 150 MHz)

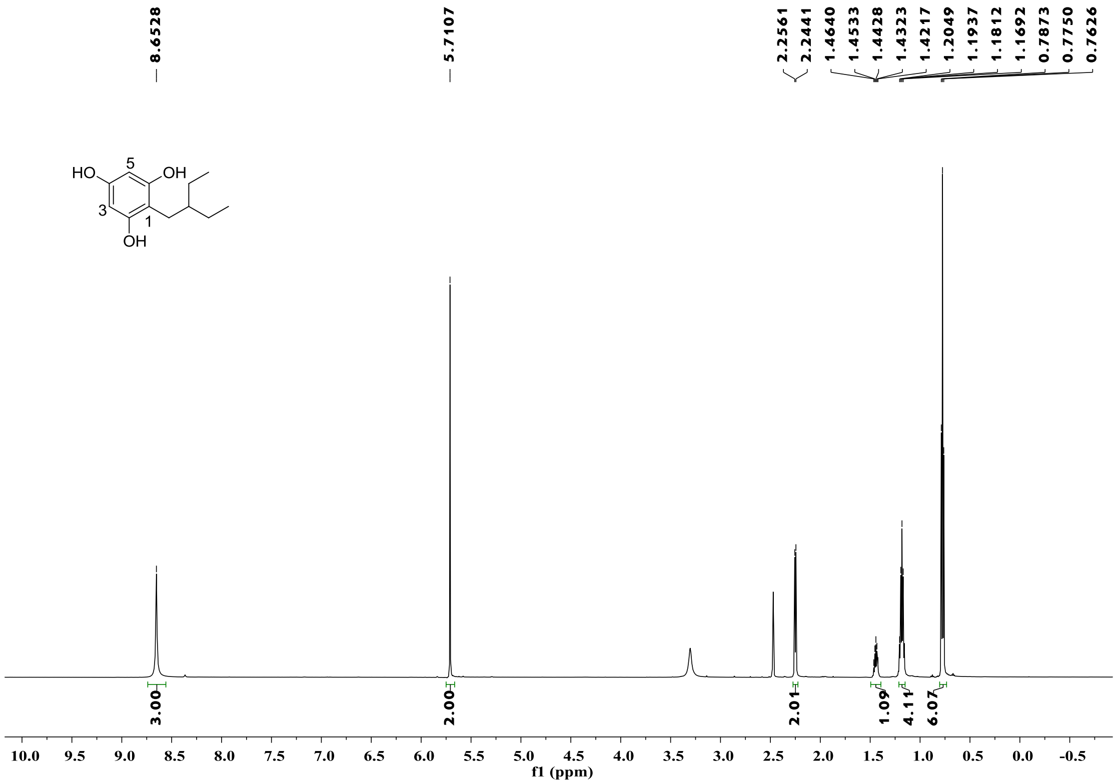

Supplementary Figure 81 ^{1}H NMR spectrum of **31** (DMSO-d_6, 600 MHz)

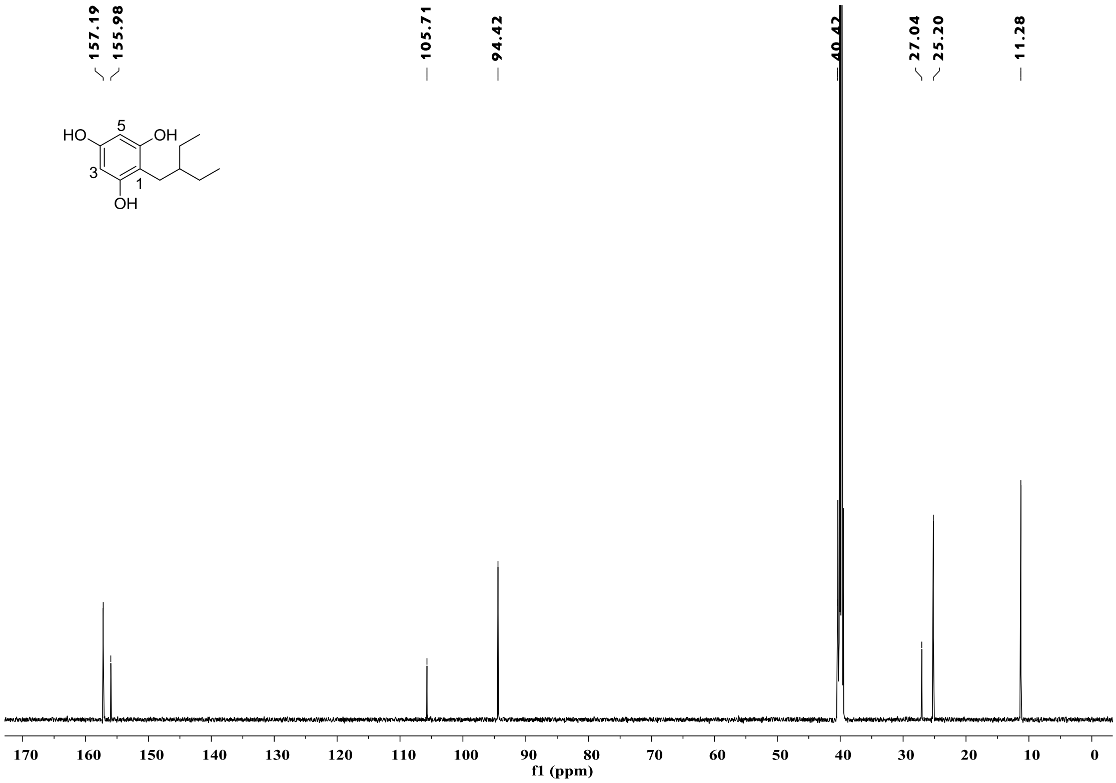

Supplementary Figure 82 ^{13}C NMR spectrum of **31** (DMSO-d_6, 150 MHz)

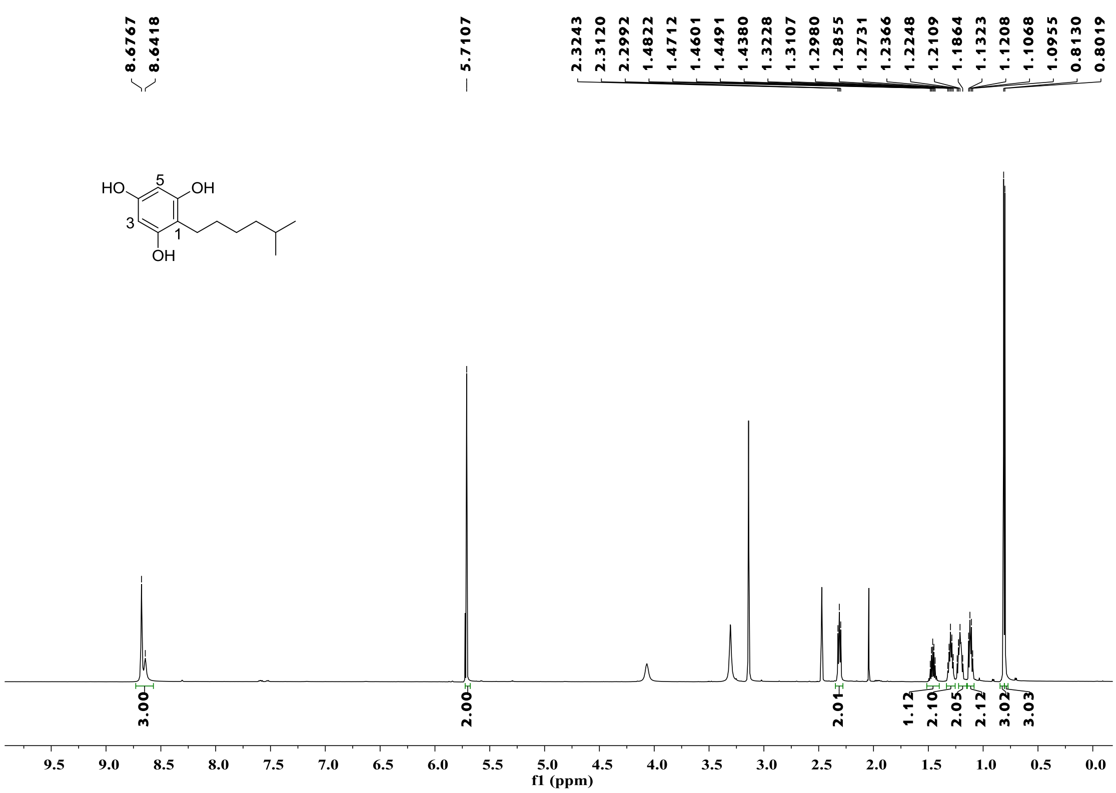

Supplementary Figure 83 ^{1}H NMR spectrum of **32** (DMSO-d_6, 600 MHz)

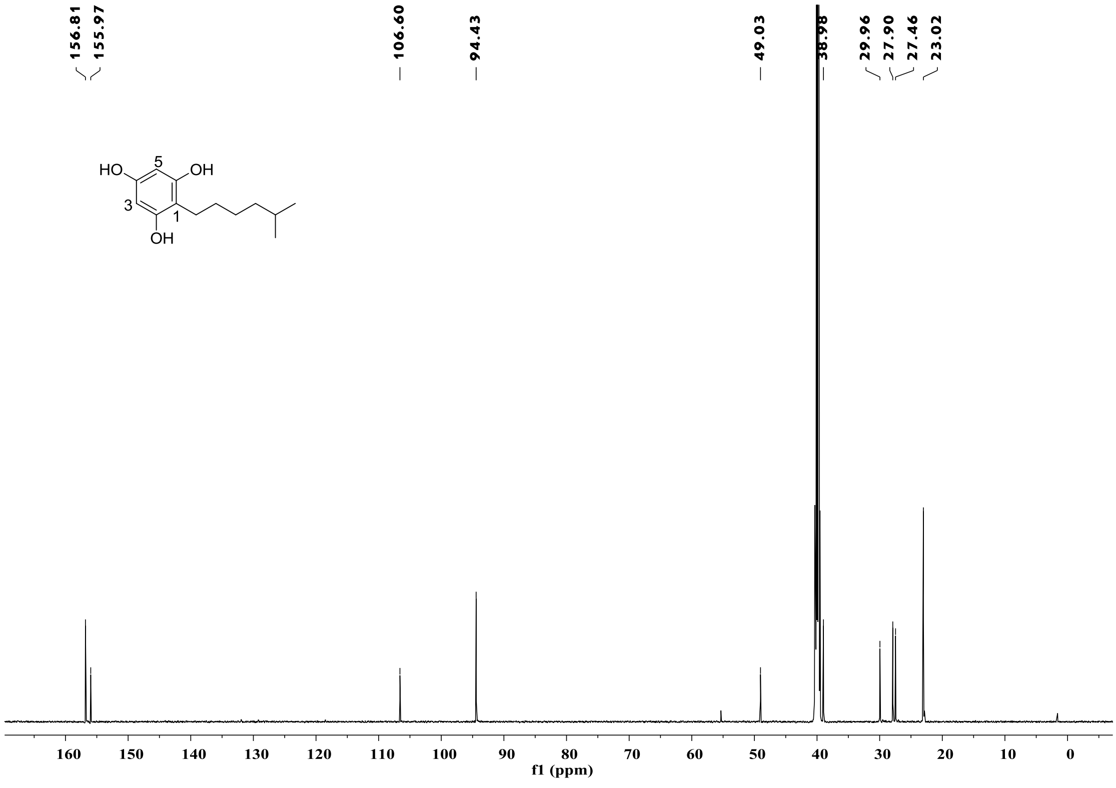

Supplementary Figure 84 ^{13}C NMR spectrum of **32** (DMSO-d_6, 150 MHz)

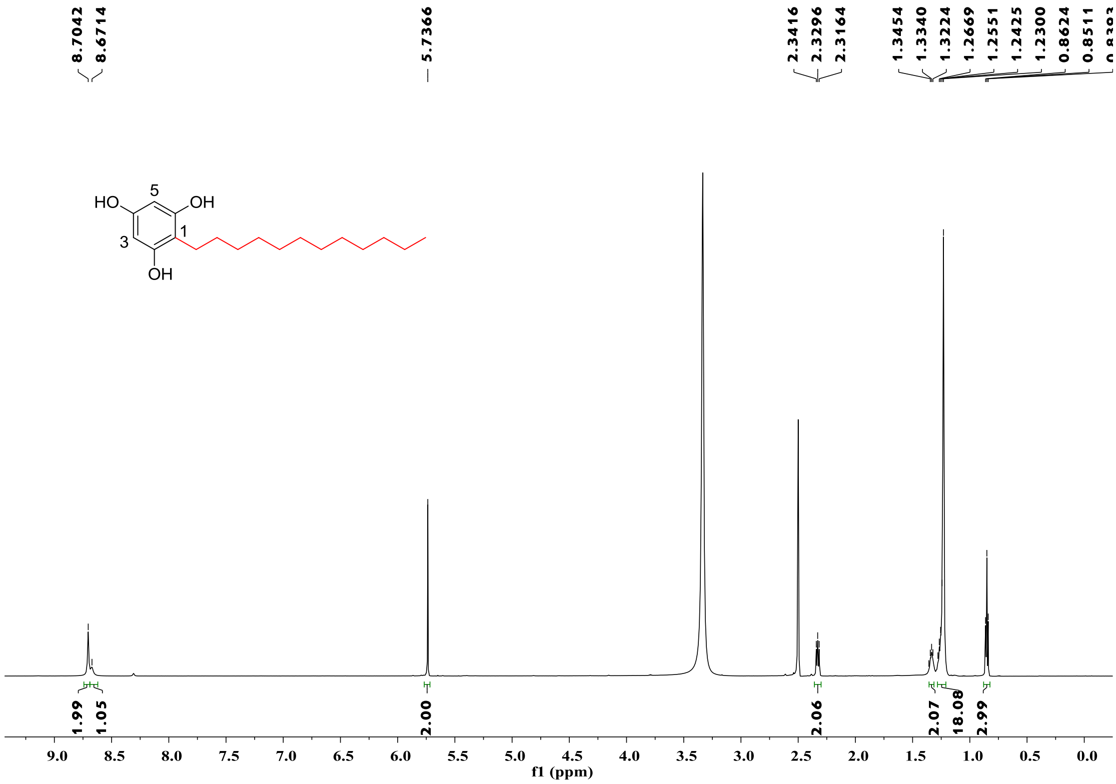

Supplementary Figure 85 ^{1}H NMR spectrum of **33** (DMSO-d_6, 600 MHz)

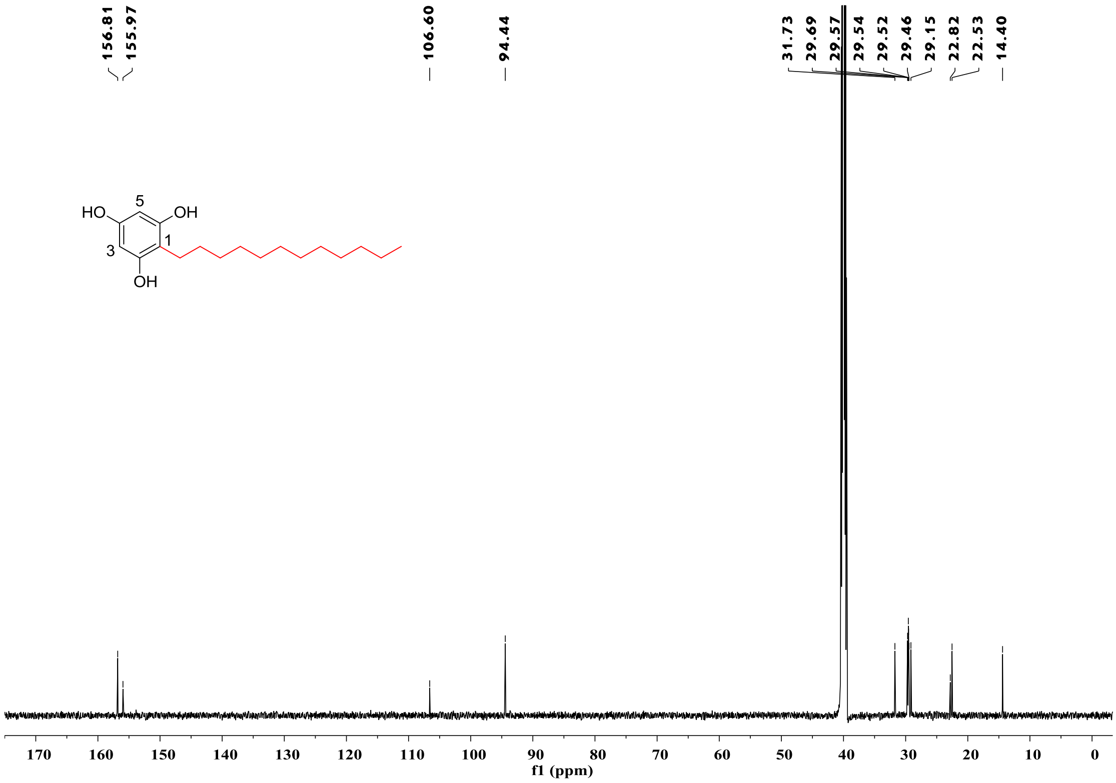

Supplementary Figure 86 ^{13}C NMR spectrum of **33** (DMSO-d_6, 150 MHz)

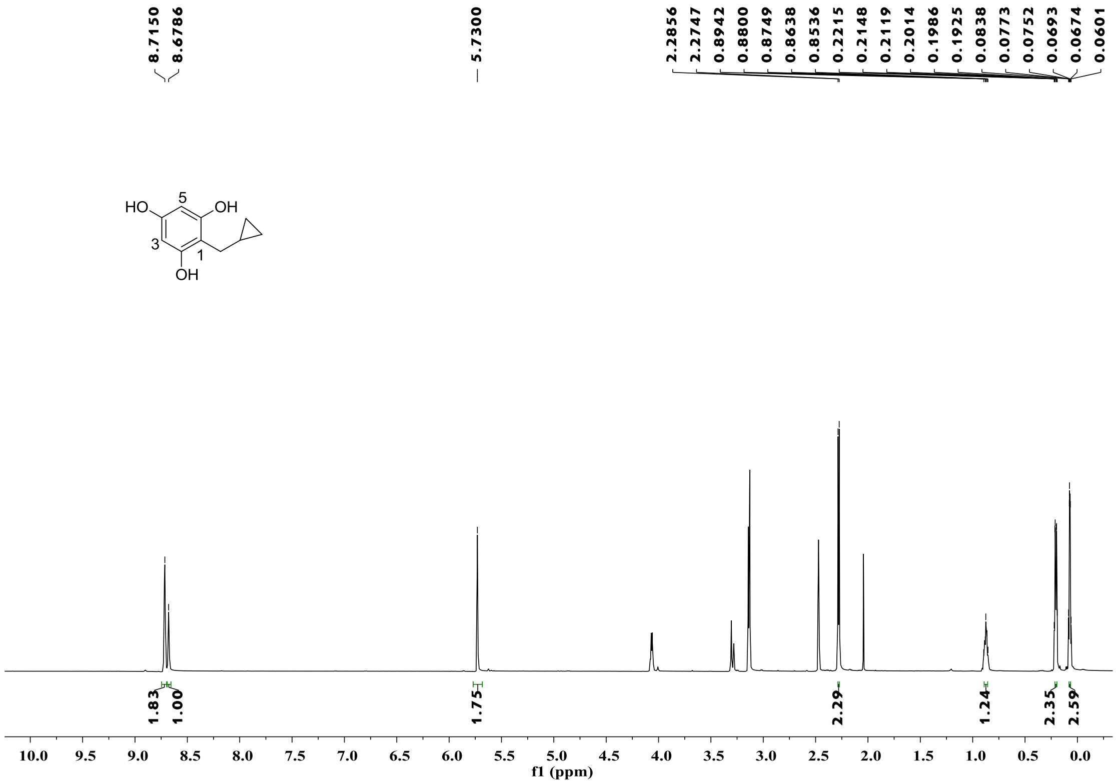

Supplementary Figure 87 ^{1}H NMR spectrum of **34** (DMSO-d_6, 600 MHz)

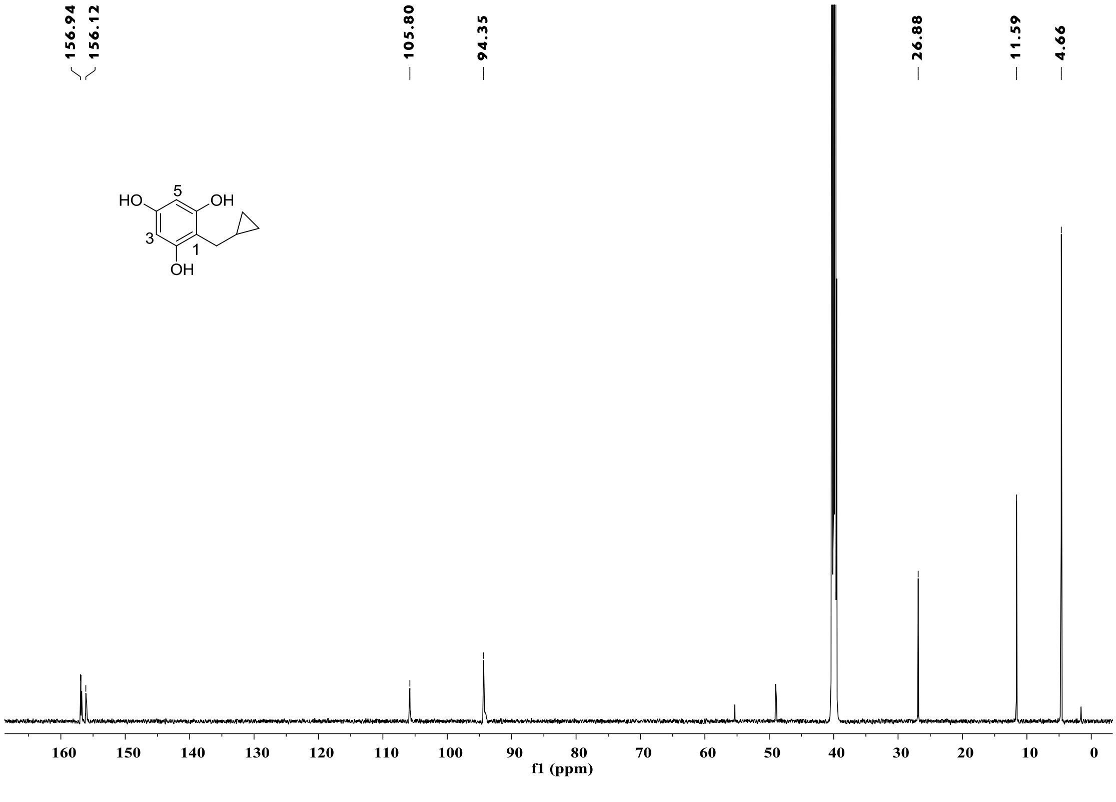

Supplementary Figure 88 ^{13}C NMR spectrum of **34** (DMSO-d_6, 150 MHz)

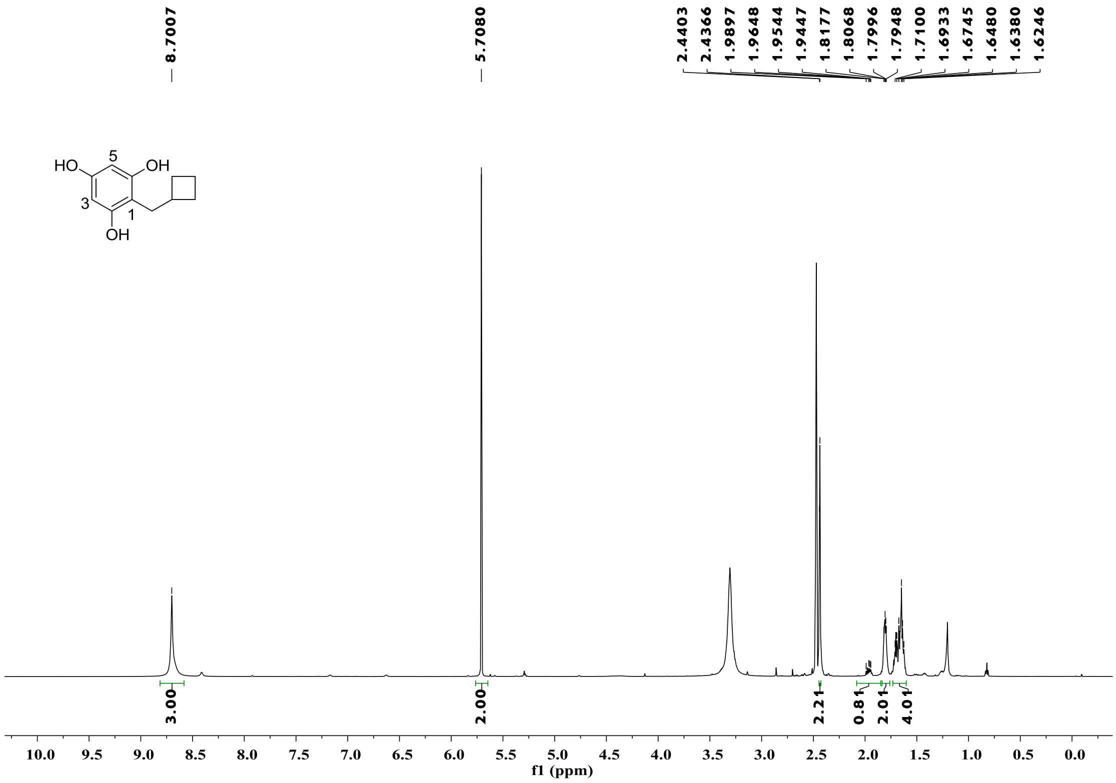

Supplementary Figure 89 ^{1}H NMR spectrum of **35** (DMSO-d_6, 600 MHz)

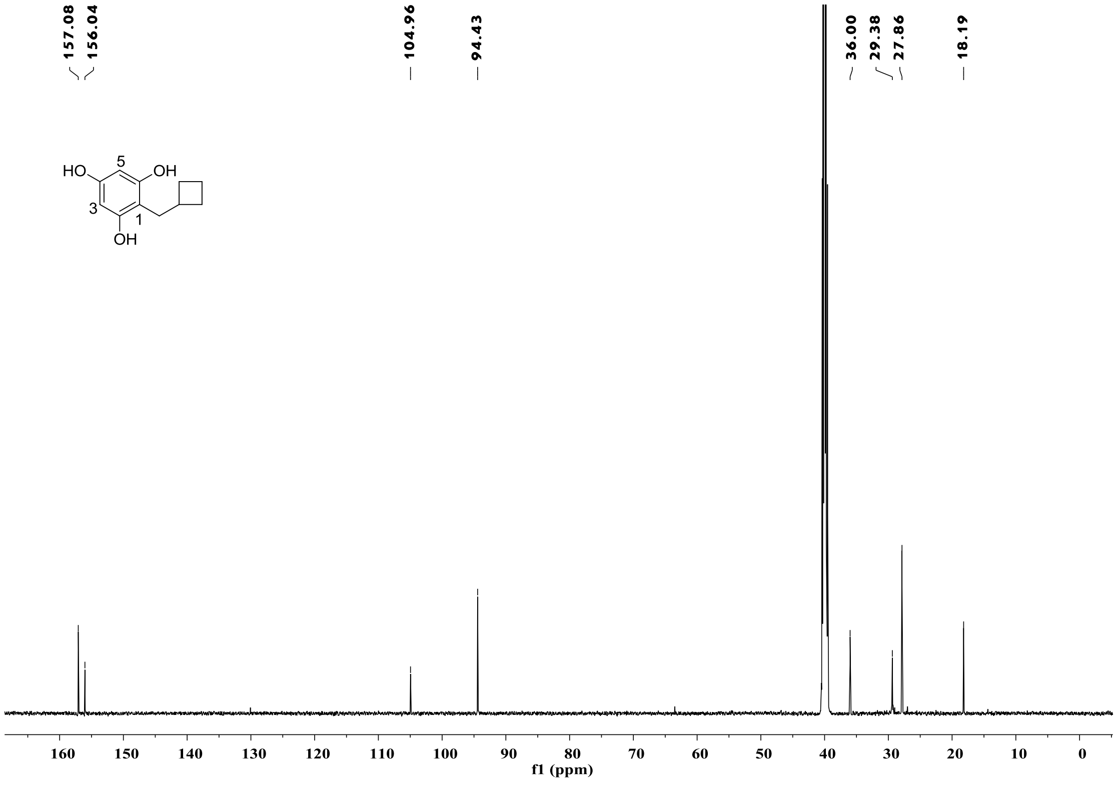

Supplementary Figure 90 ^{13}C NMR spectrum of **35** (DMSO-d_6, 150 MHz)

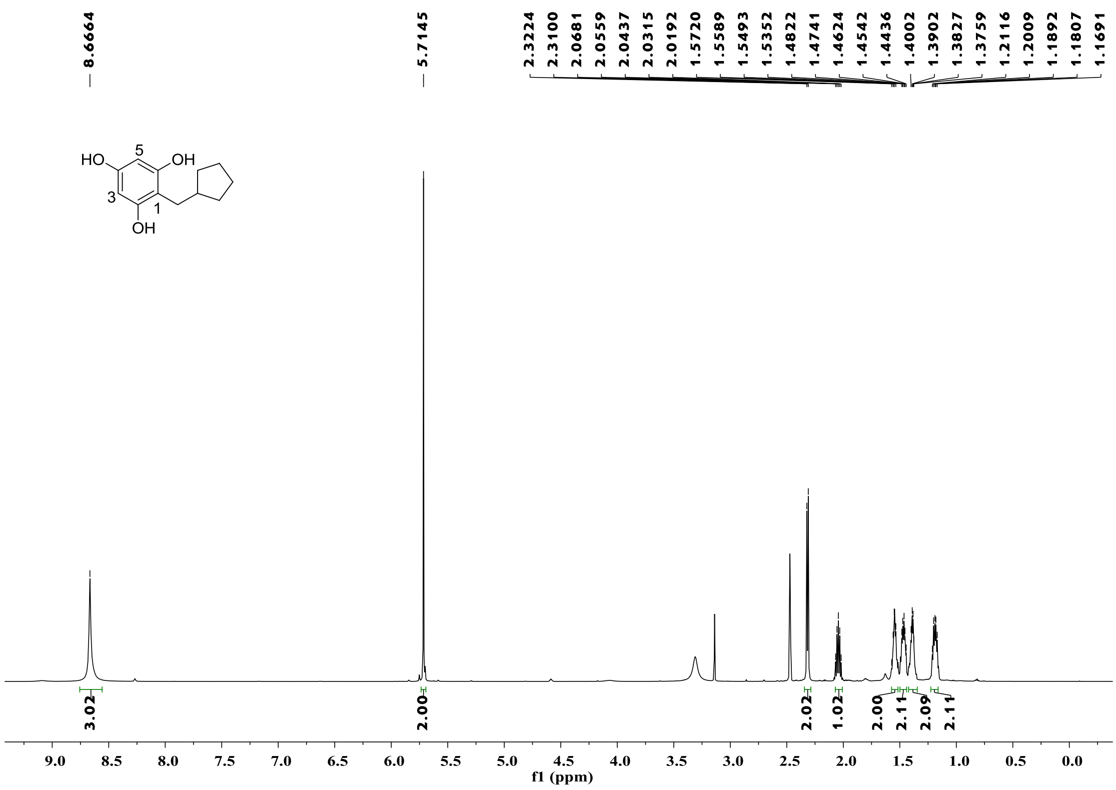

Supplementary Figure 91 ^{1}H NMR spectrum of **36** (DMSO-d_6, 600 MHz)

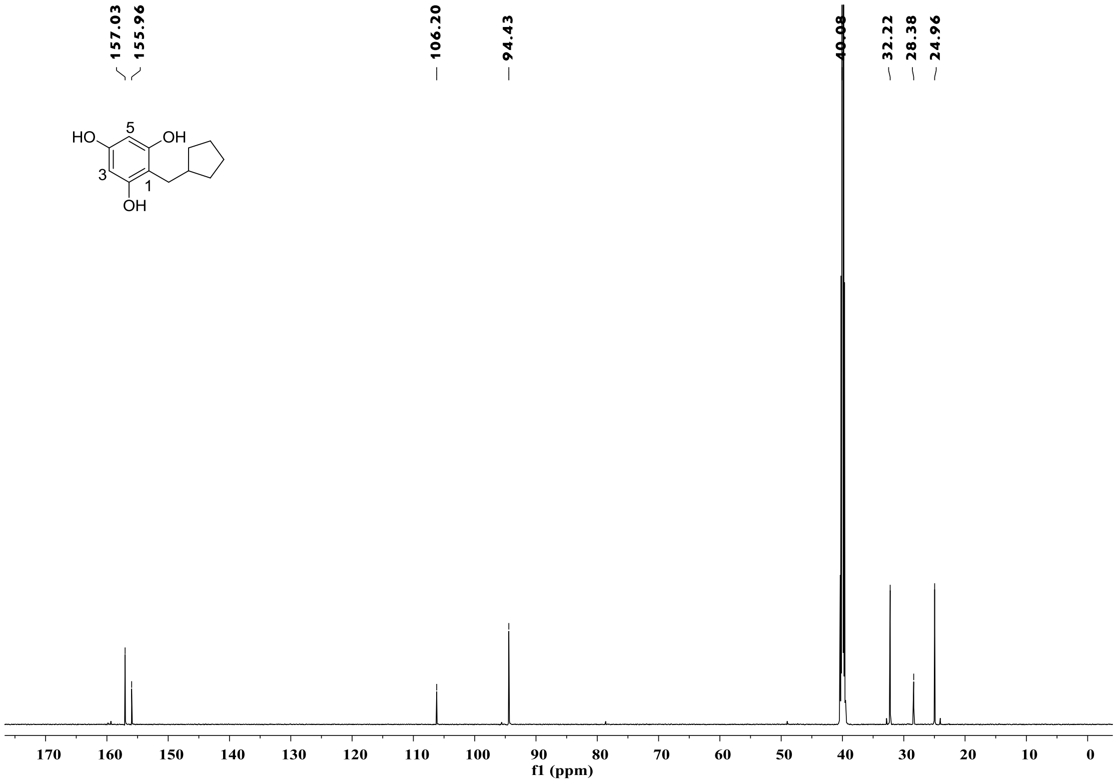

Supplementary Figure 92 ^{13}C NMR spectrum of **36** (DMSO-d_6, 150 MHz)

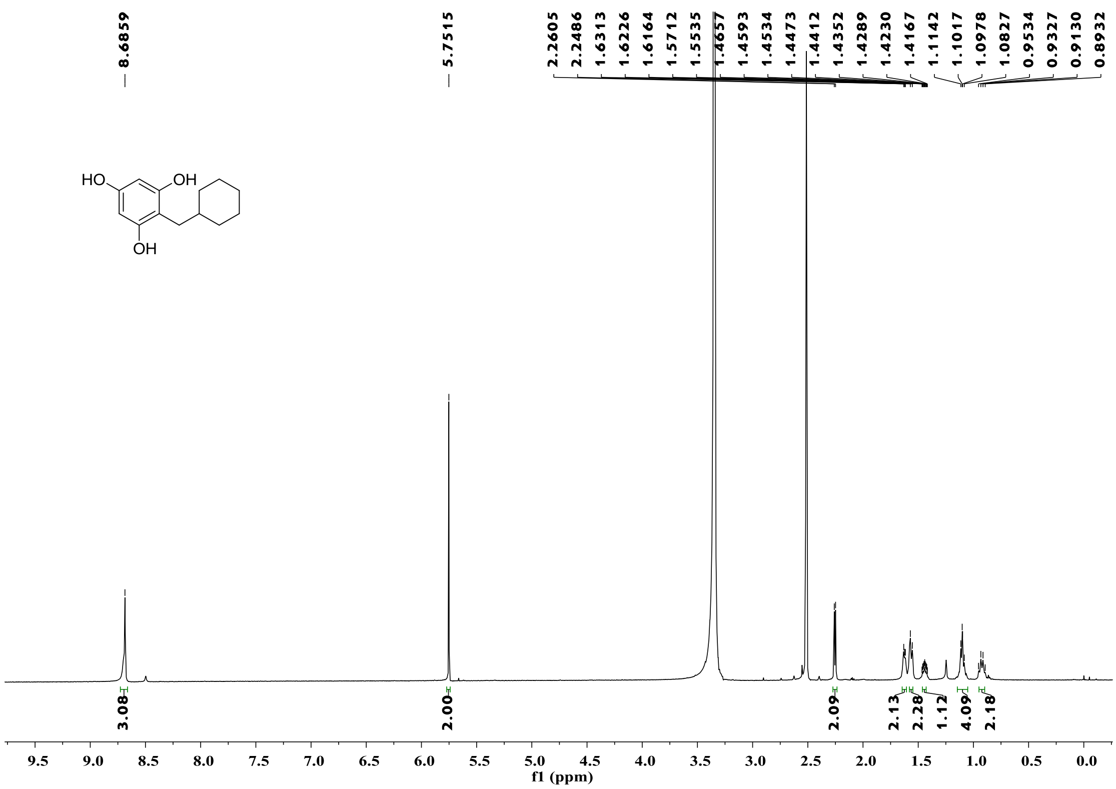

Supplementary Figure 93 ^{1}H NMR spectrum of **37** (DMSO-d_6, 600 MHz)

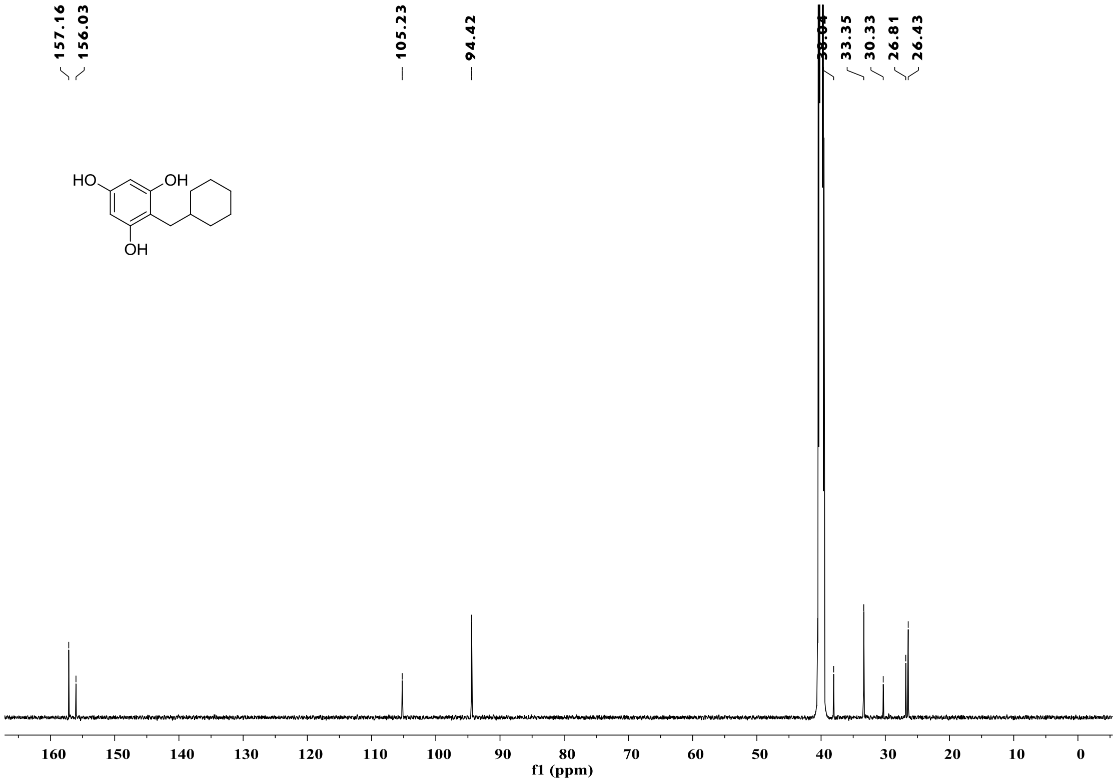

Supplementary Figure 94 ^{13}C NMR spectrum of **37** (DMSO-d_6, 150 MHz)

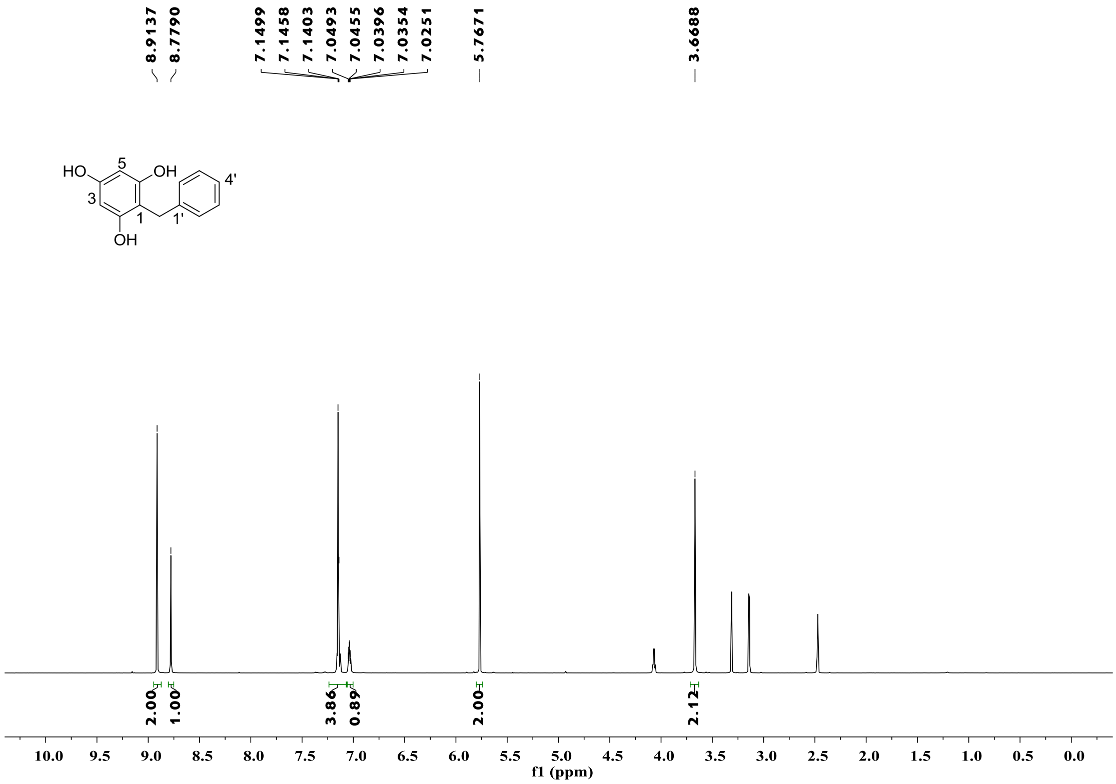

Supplementary Figure 95 ^{1}H NMR spectrum of **38** (DMSO-d_6, 600 MHz)

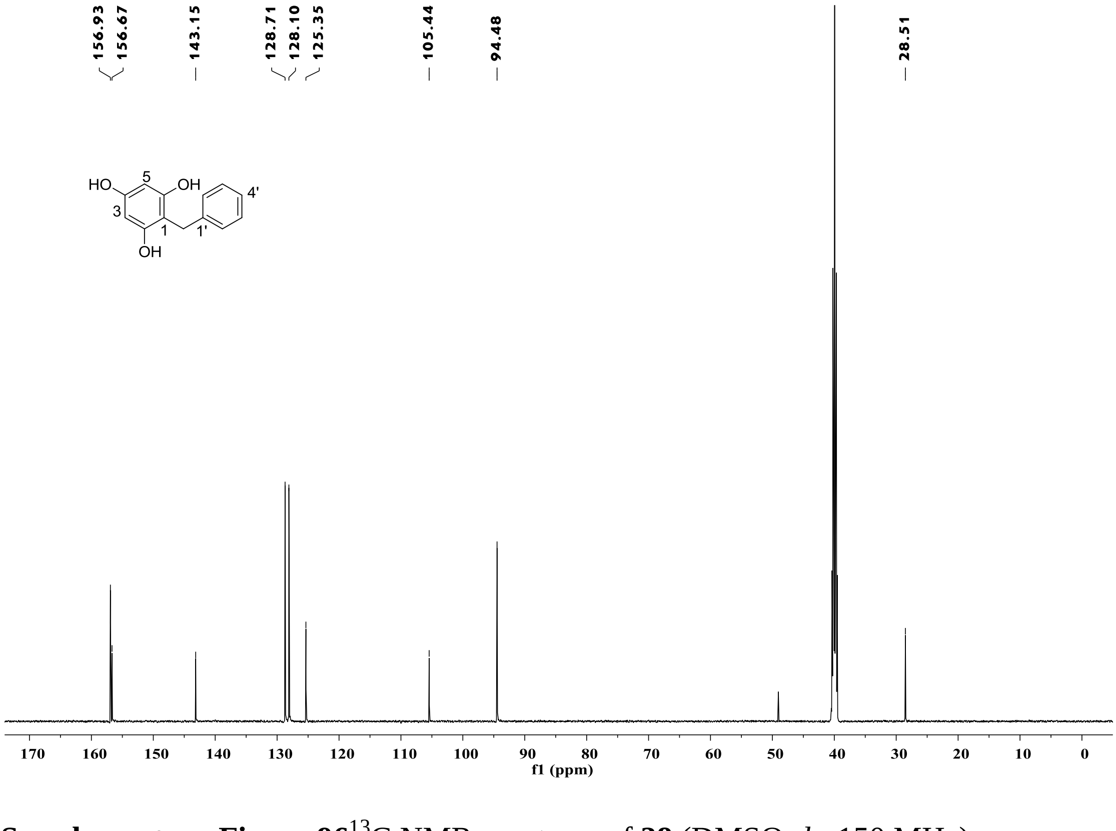

Supplementary Figure 96^{13}C NMR spectrum of **38** (DMSO-d_6, 150 MHz)

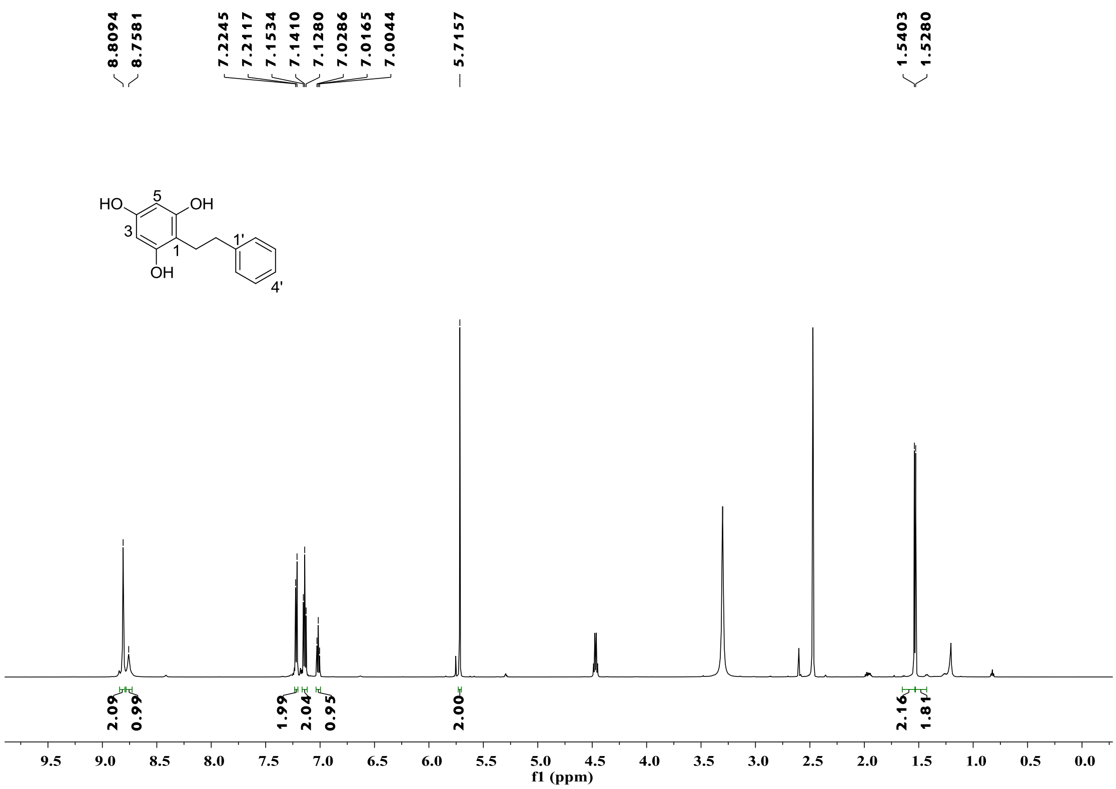

Supplementary Figure 97 ^{1}H NMR spectrum of **39** (DMSO-d_6, 600 MHz)

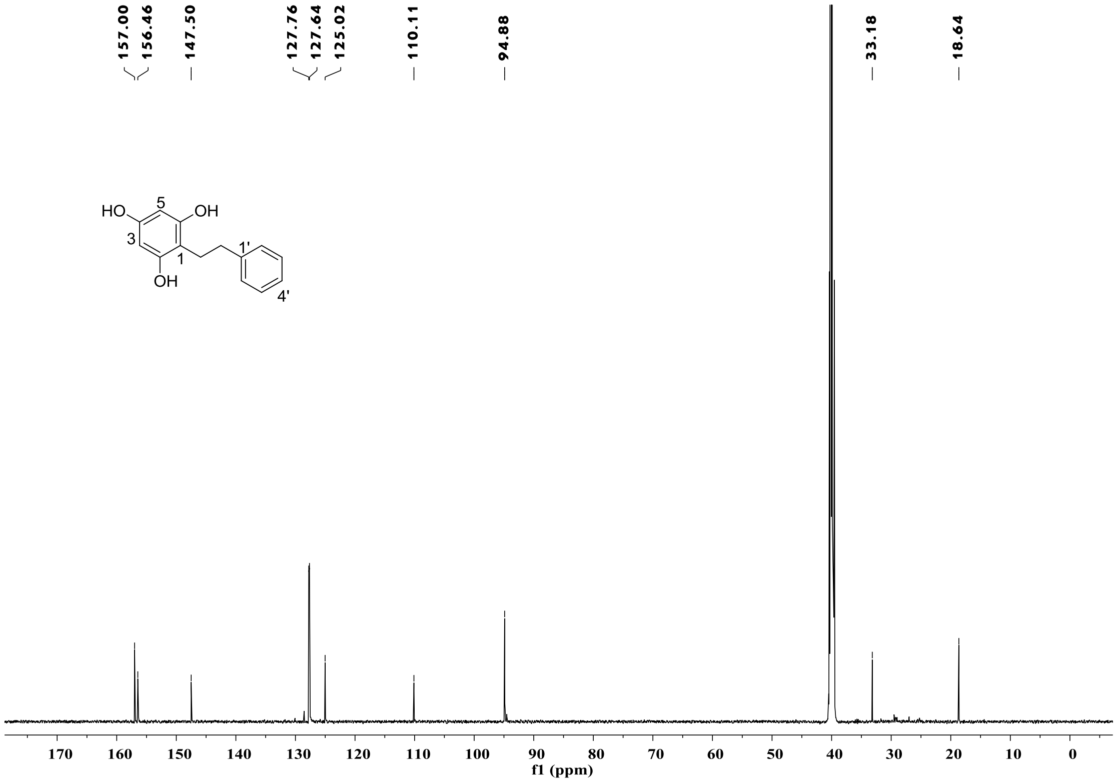

Supplementary Figure 98 ^{13}C NMR spectrum of **39** (DMSO-d_6, 150 MHz)

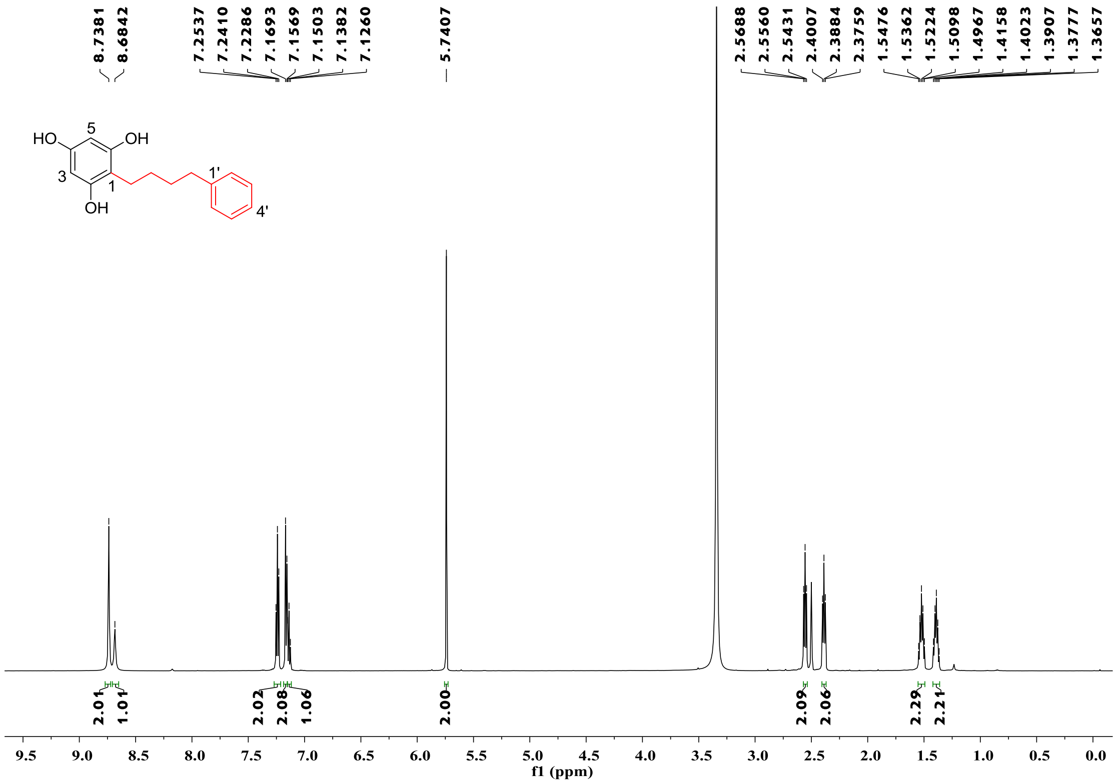

Supplementary Figure 99 ^{1}H NMR spectrum of **40** (DMSO-d_6, 600 MHz)

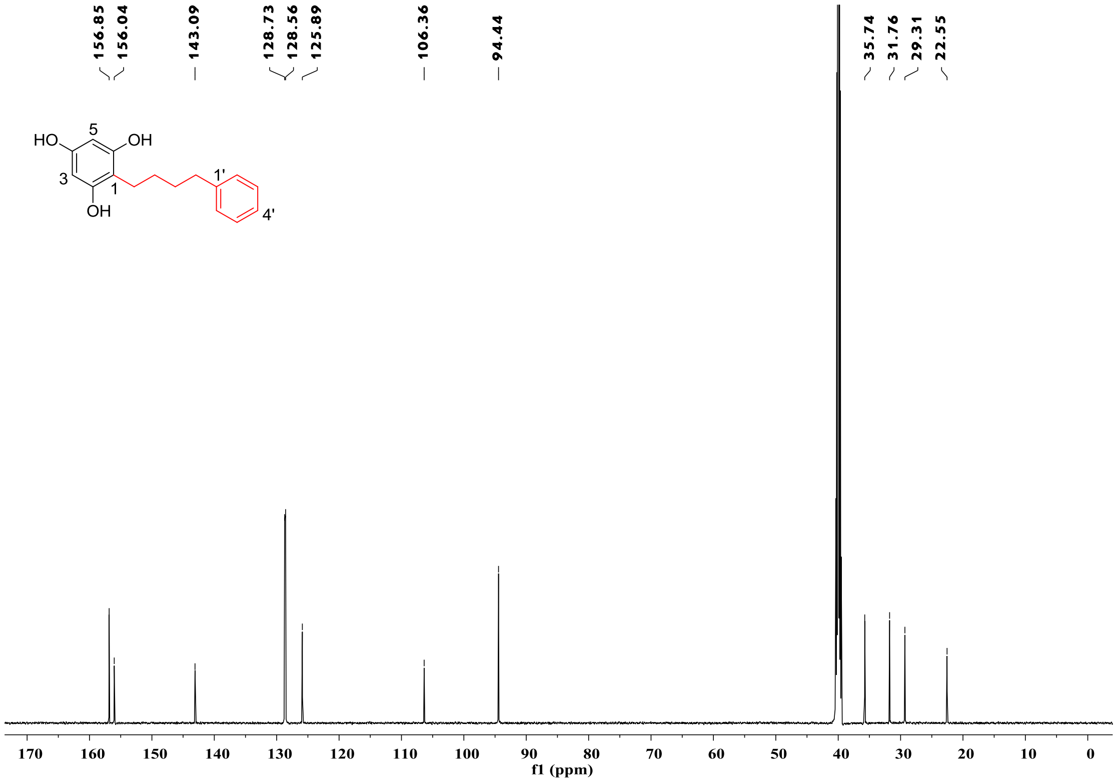

Supplementary Figure 100 ^{13}C NMR spectrum of **40** (DMSO-d_6, 150 MHz)

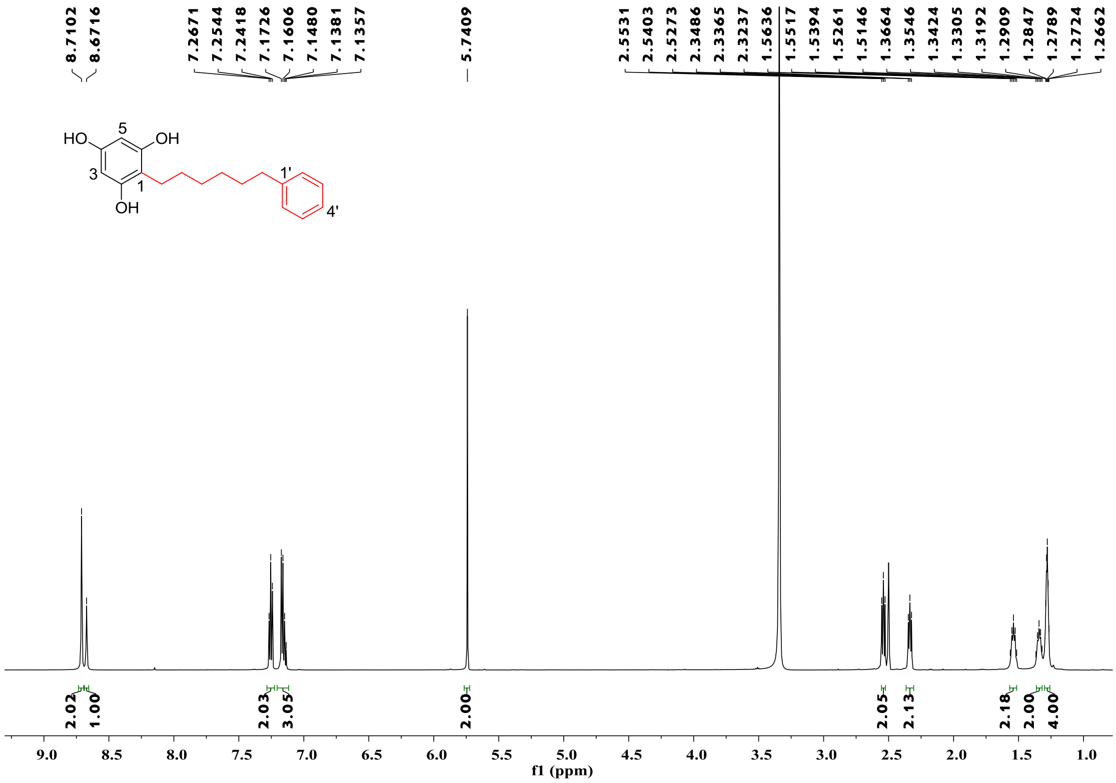

Supplementary Figure 101 ¹H NMR spectrum of **41** (DMSO-d_6, 600 MHz)

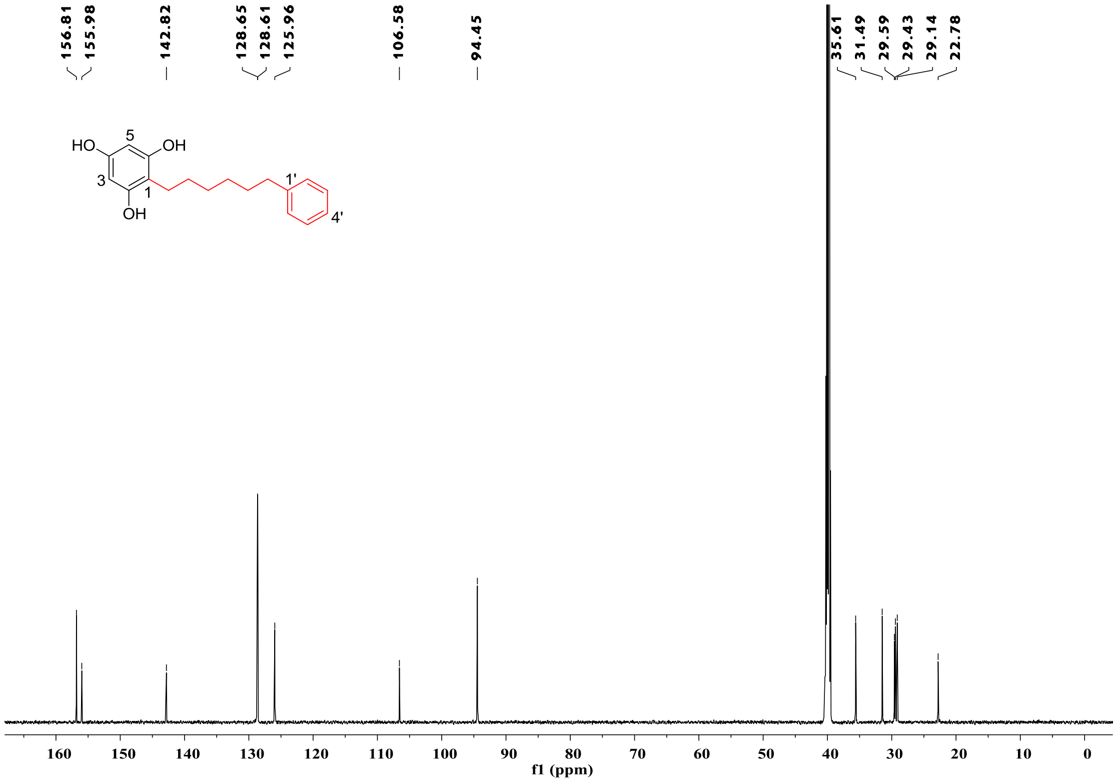

Supplementary Figure 102 ¹³C NMR spectrum of **41** (DMSO-d_6, 150 MHz)

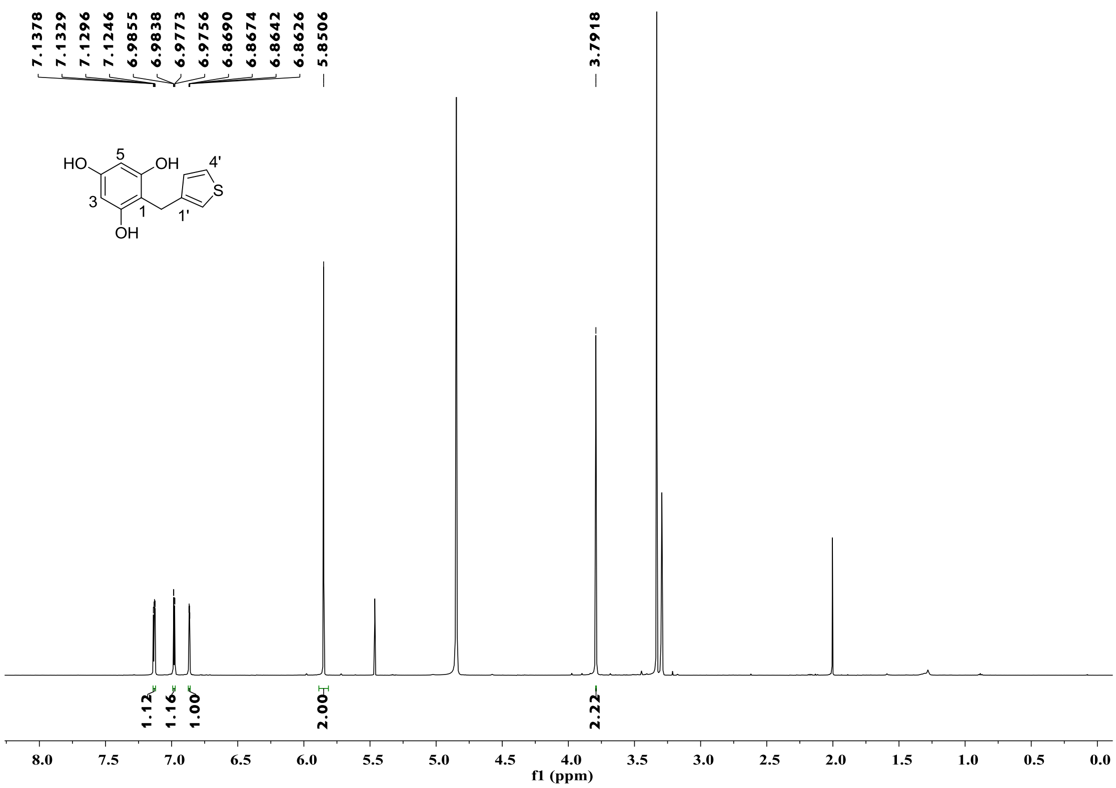

Supplementary Figure 103 ^{1}H NMR spectrum of **42** (Methanol-d_4, 600 MHz)

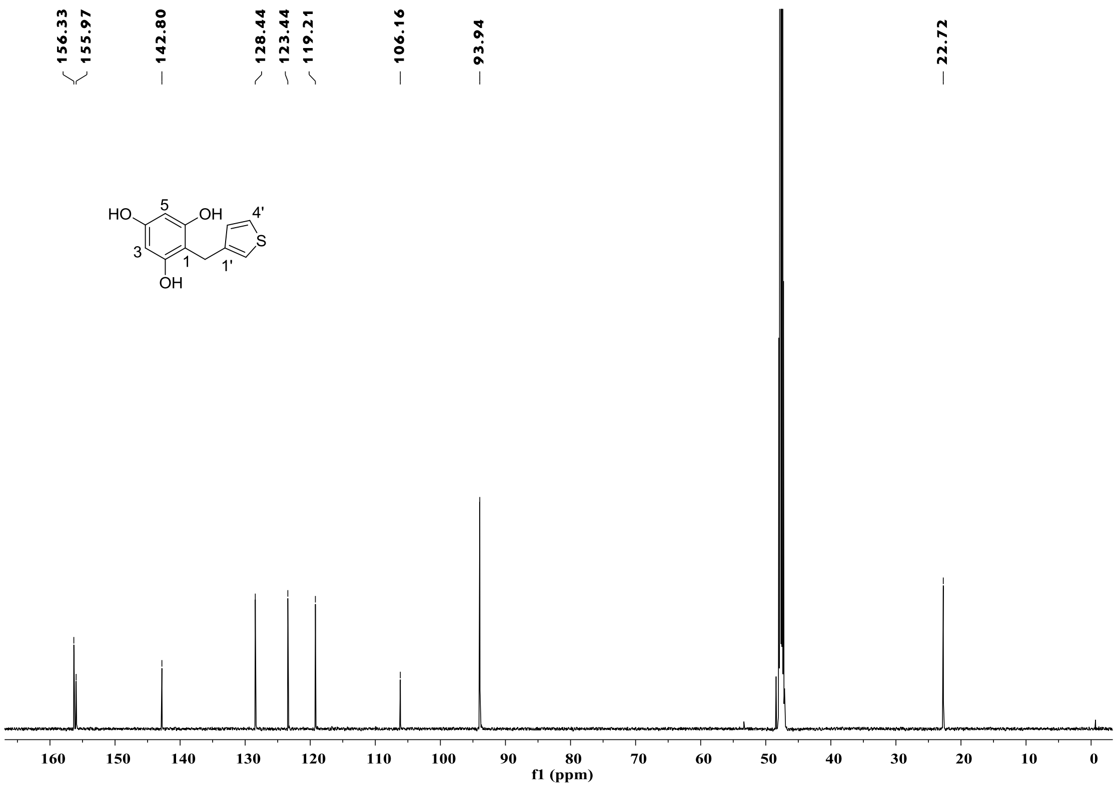

Supplementary Figure 104 ^{13}C NMR spectrum of **42** (Methanol-d_4, 150 MHz)

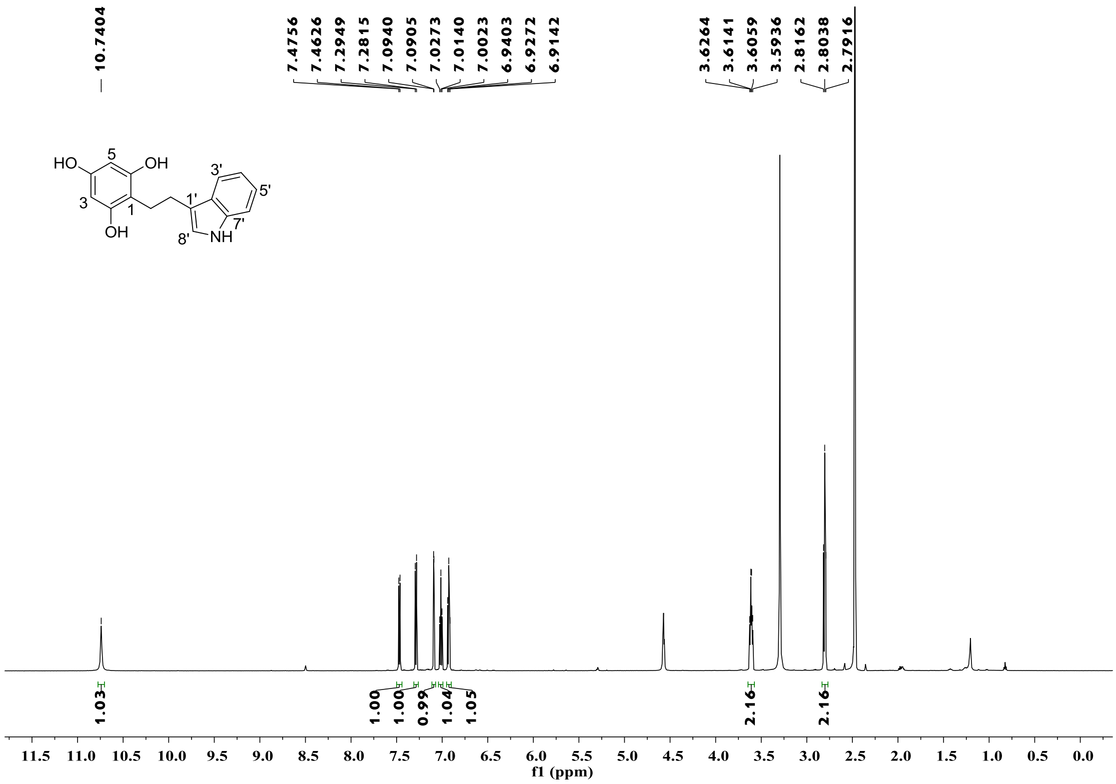

Supplementary Figure 105 ^{1}H NMR spectrum of **43** (DMSO-d_6, 600 MHz)

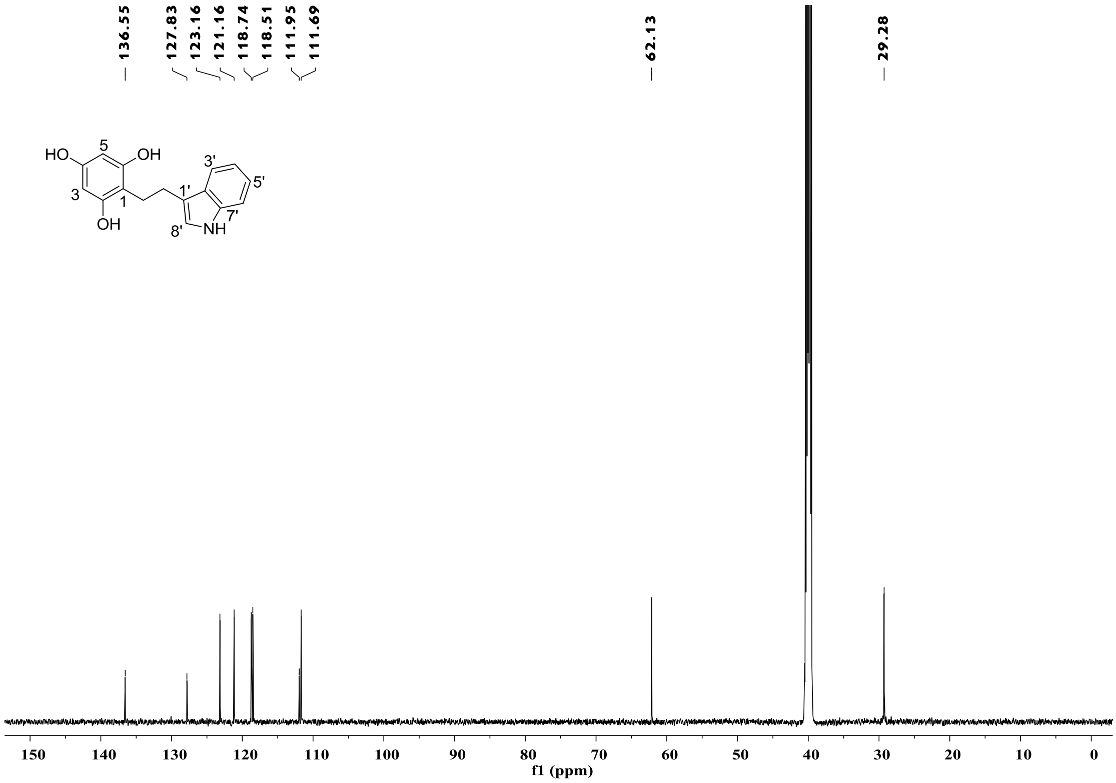

Supplementary Figure 106 ^{13}C NMR spectrum of **43** (DMSO-d_6, 150 MHz)

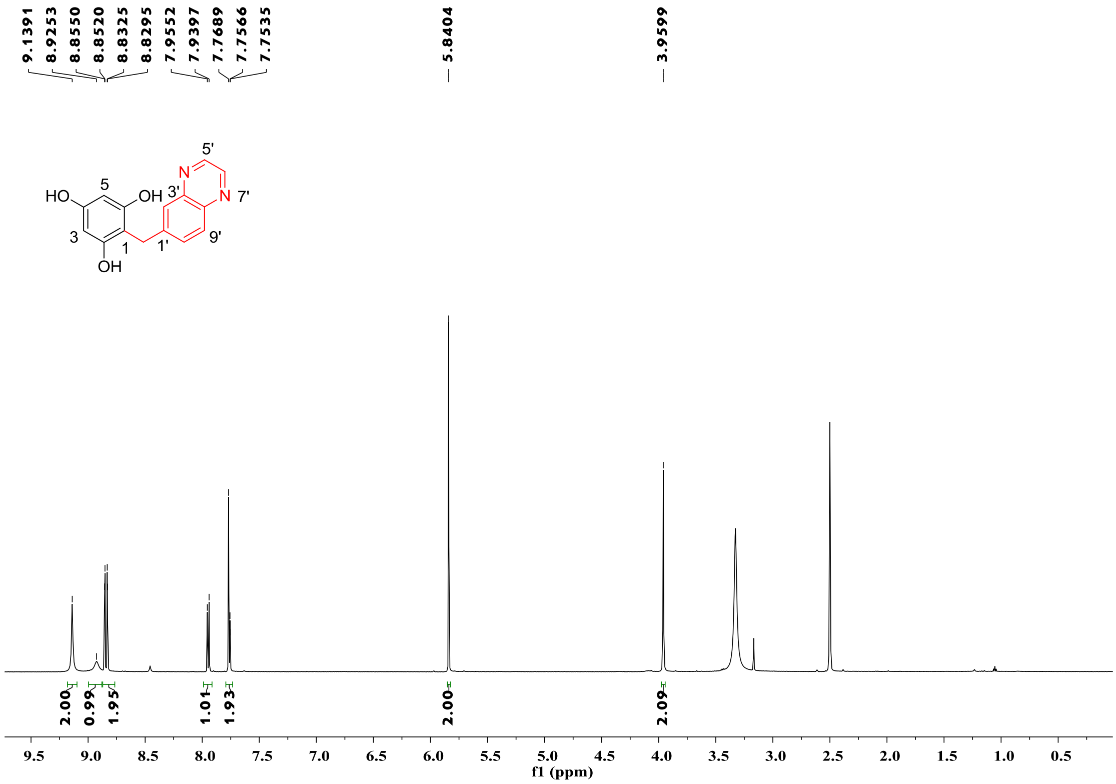

Supplementary Figure 107 ^{1}H NMR spectrum of **44** (DMSO-d_6, 600 MHz)

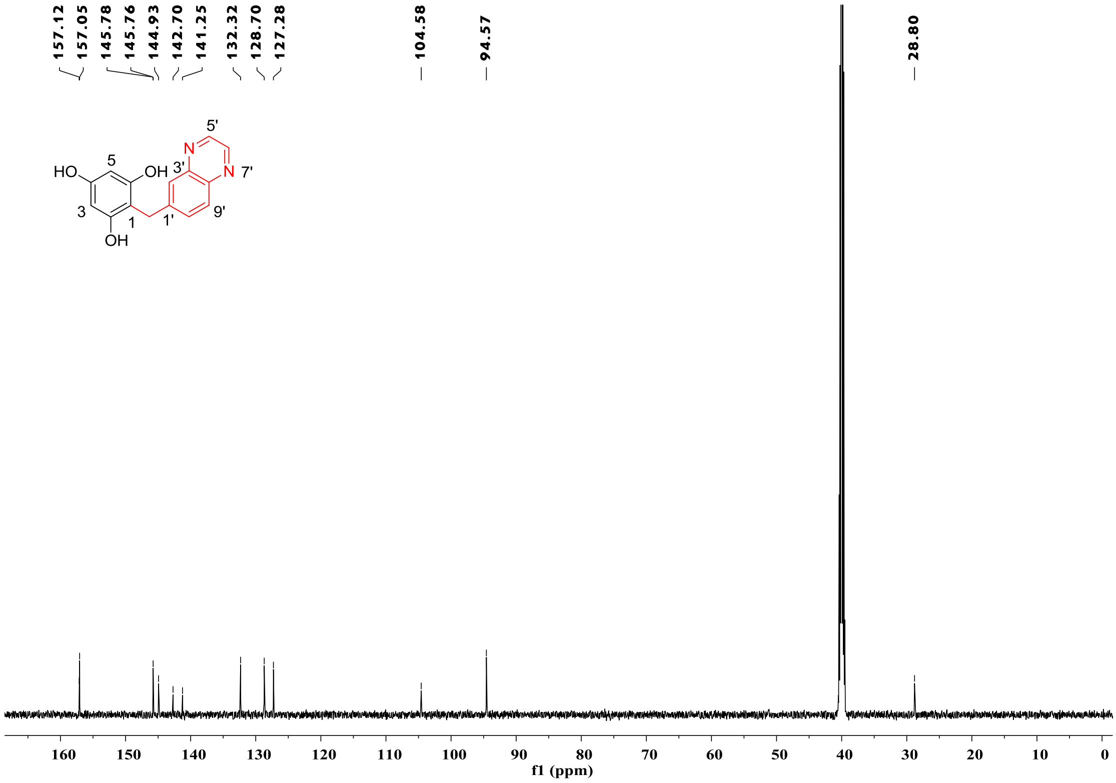

Supplementary Figure 108 ^{13}C NMR spectrum of **44** (DMSO-d_6, 150 MHz)

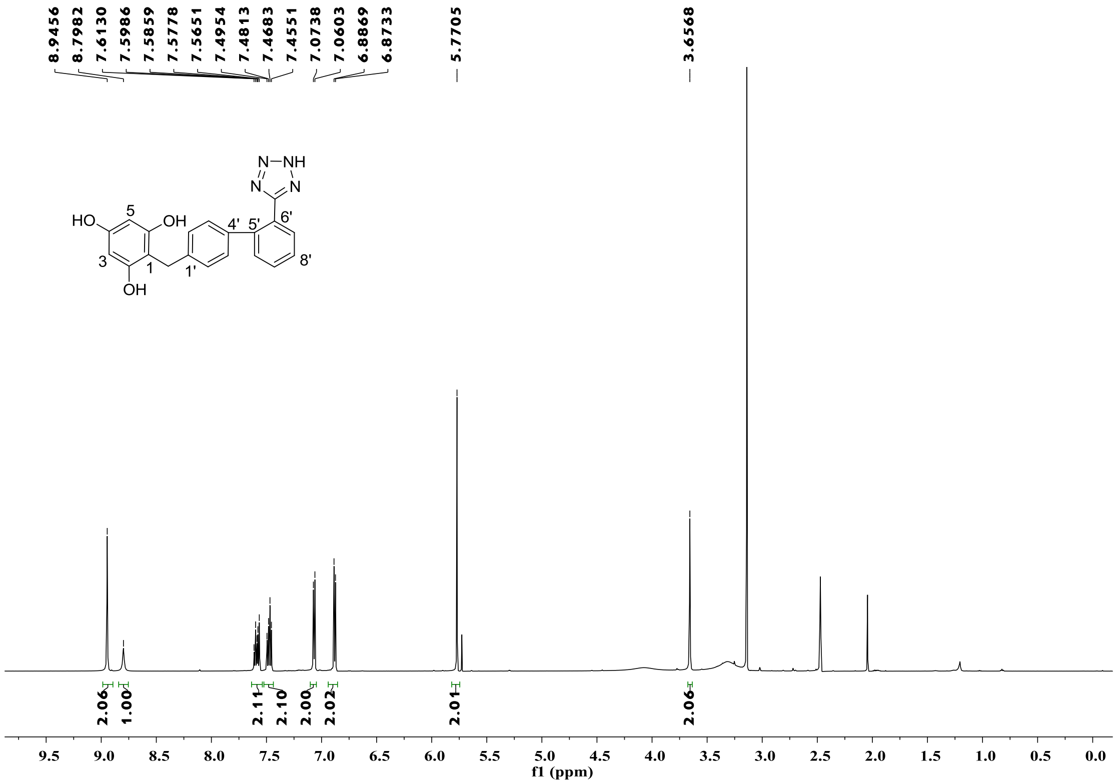

Supplementary Figure 109 ^{1}H NMR spectrum of **45** (DMSO-d_6, 600 MHz)

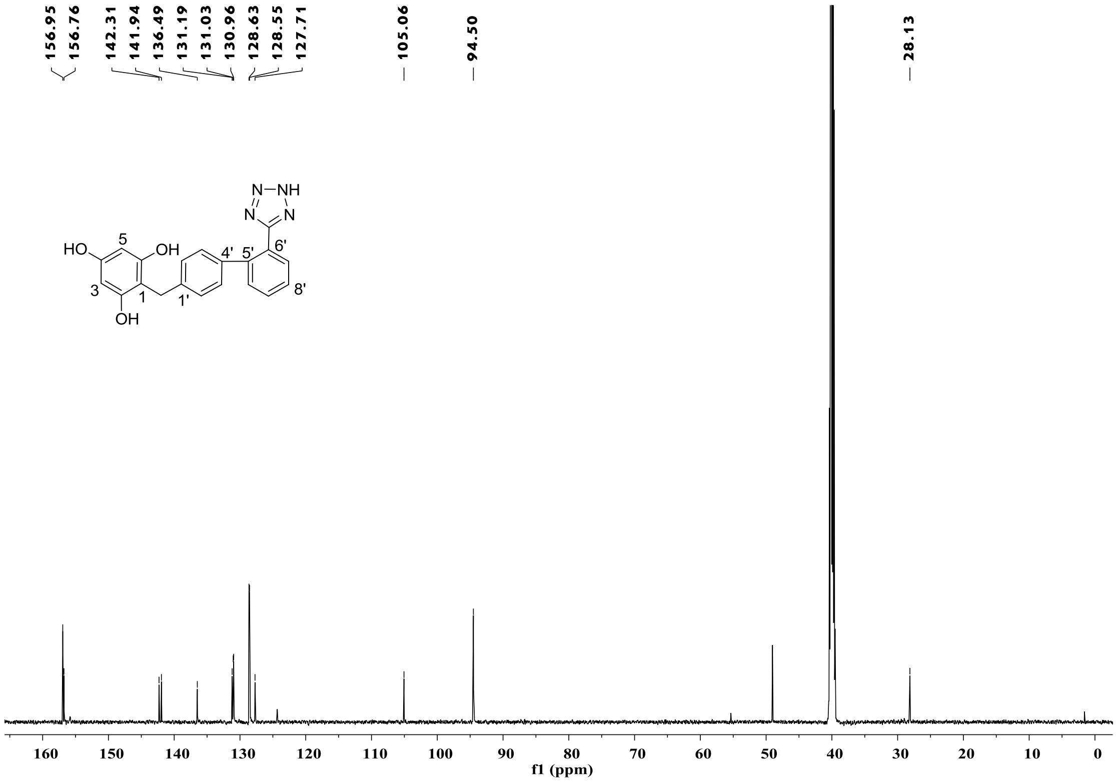

Supplementary Figure 110 ^{13}C NMR spectrum of **45** (DMSO-d_6, 150 MHz)

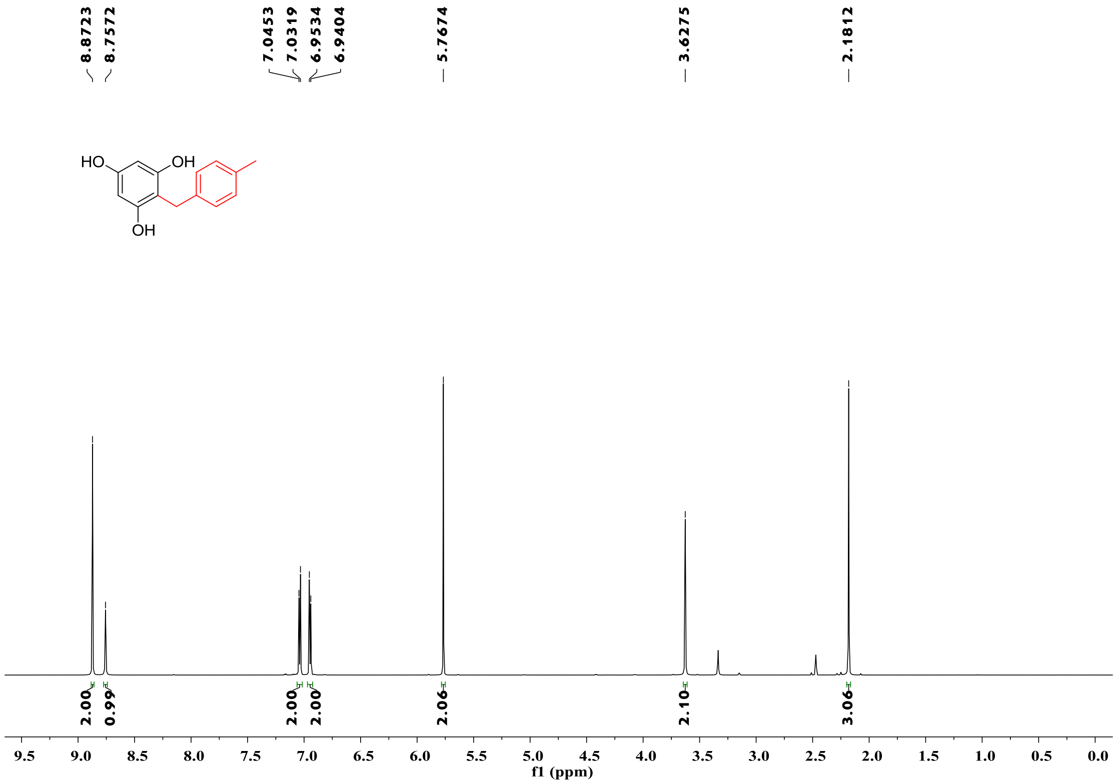

Supplementary Figure 111 ^{1}H NMR spectrum of **46** (DMSO-d_6, 600 MHz)

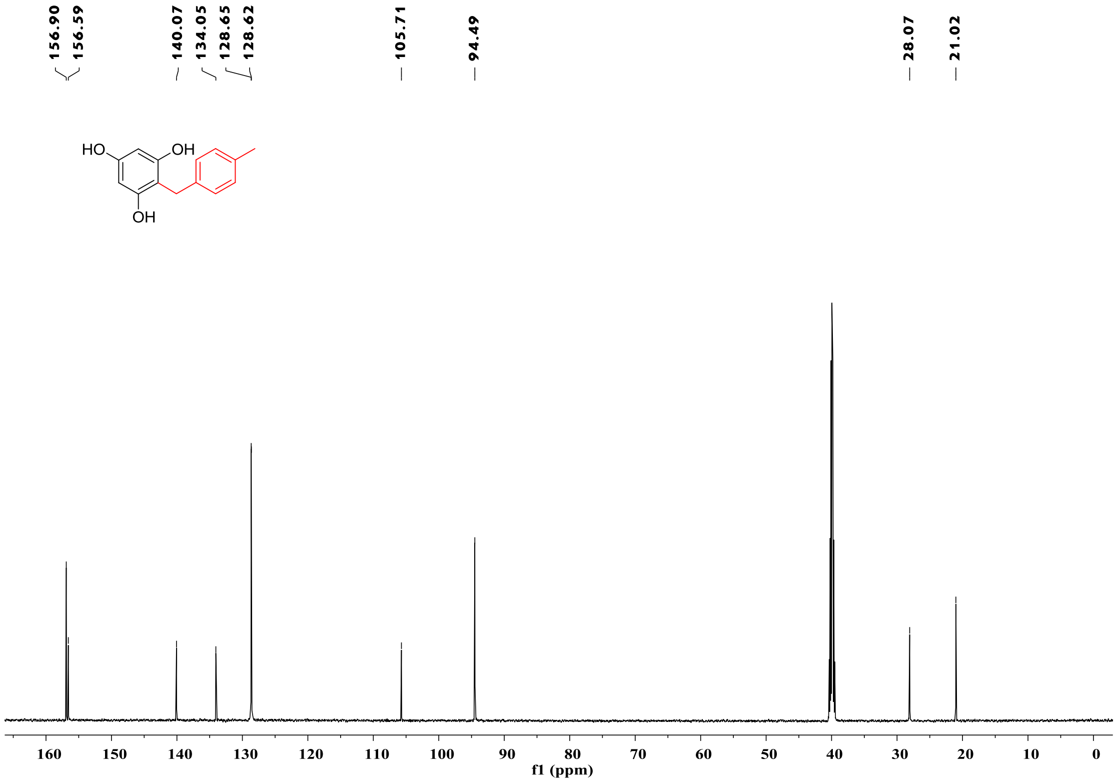

Supplementary Figure 112 ^{13}C NMR spectrum of **46** (DMSO-d_6, 150 MHz)

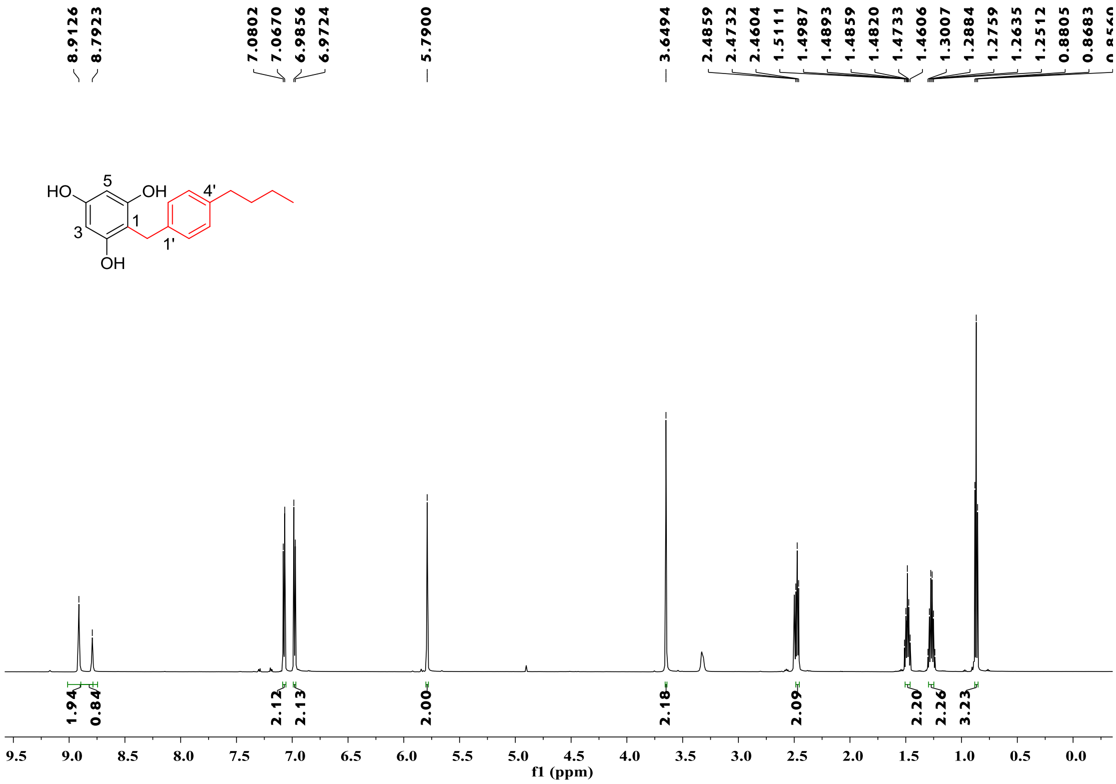

Supplementary Figure 113 ^{1}H NMR spectrum of **47** (DMSO-d_6, 600 MHz)

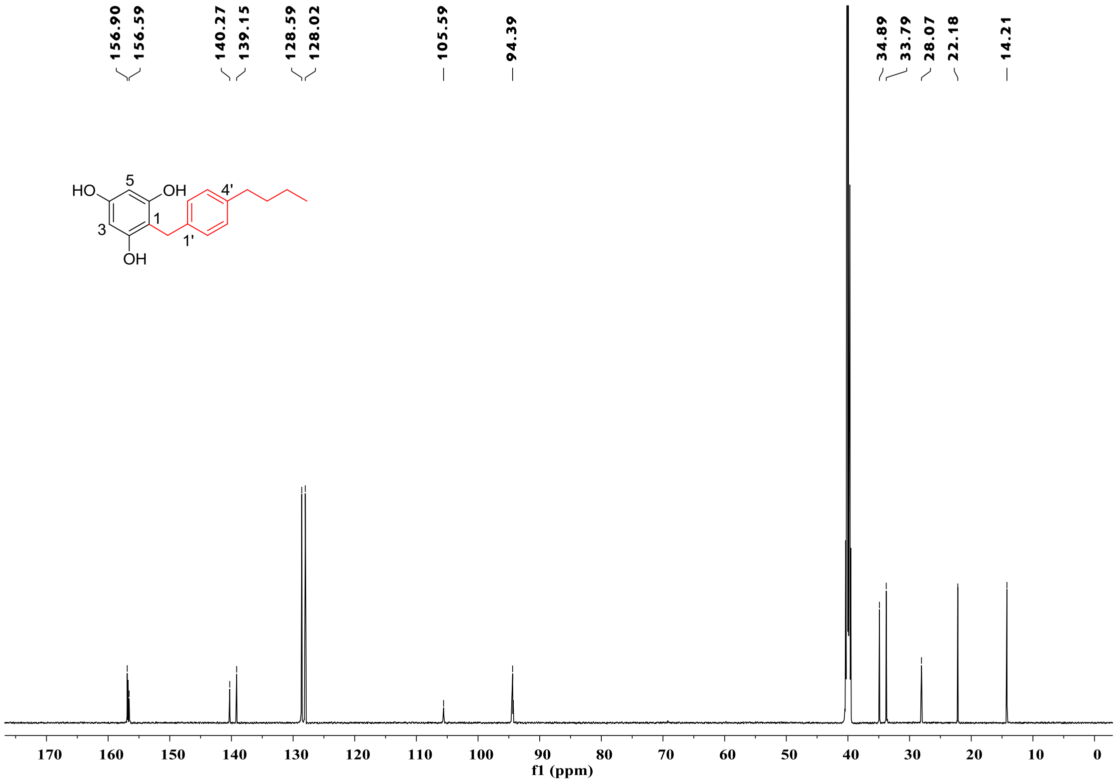

Supplementary Figure 114 ^{13}C NMR spectrum of **47** (DMSO-d_6, 150 MHz)

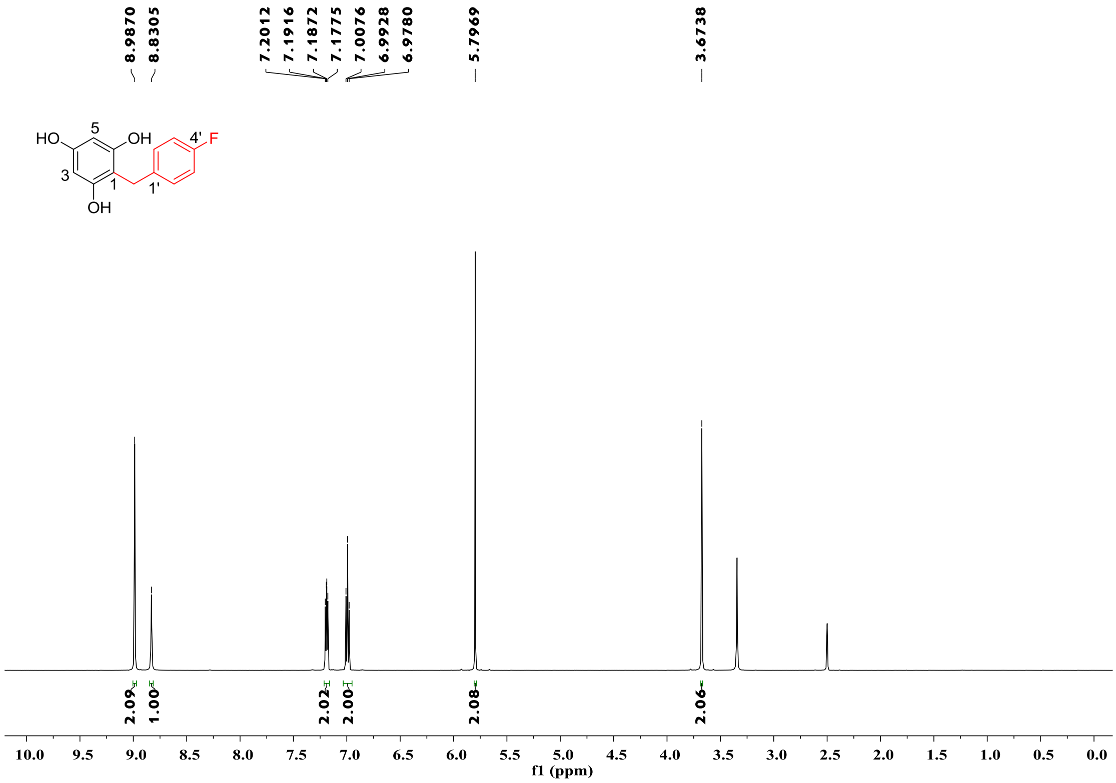

Supplementary Figure 115 ^{1}H NMR spectrum of **48** (DMSO-d_6, 600 MHz)

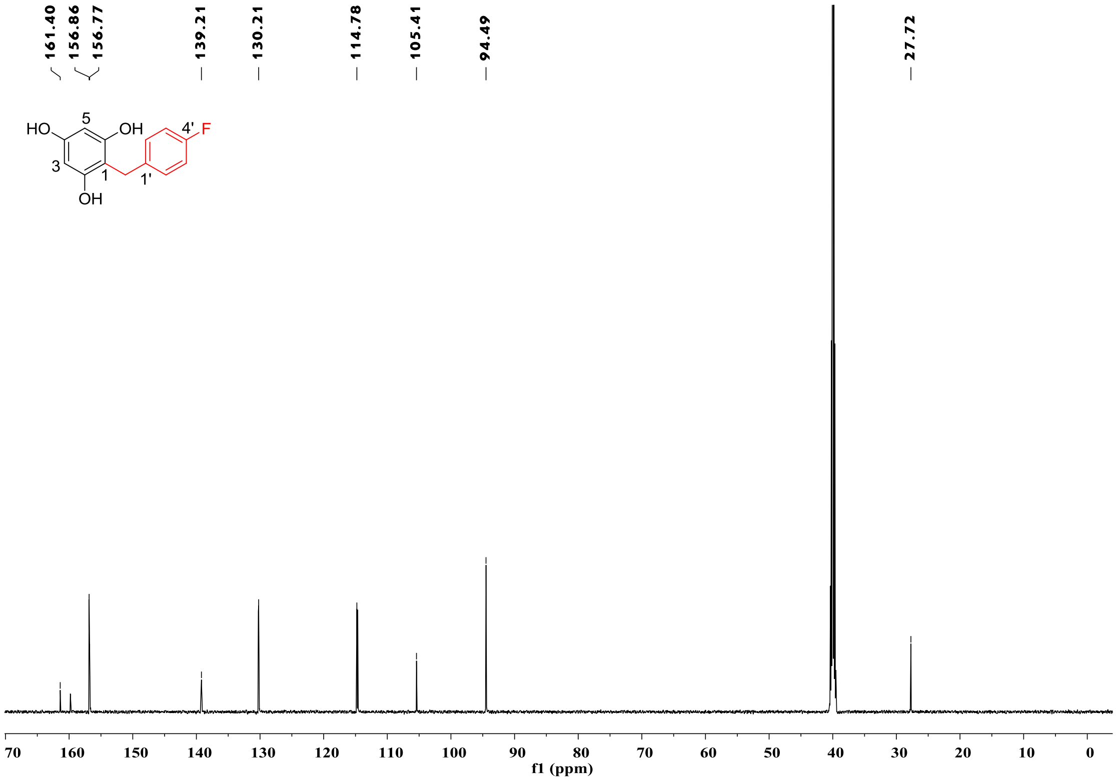

Supplementary Figure 116 ^{13}C NMR spectrum of **48** (DMSO-d_6, 150 MHz)

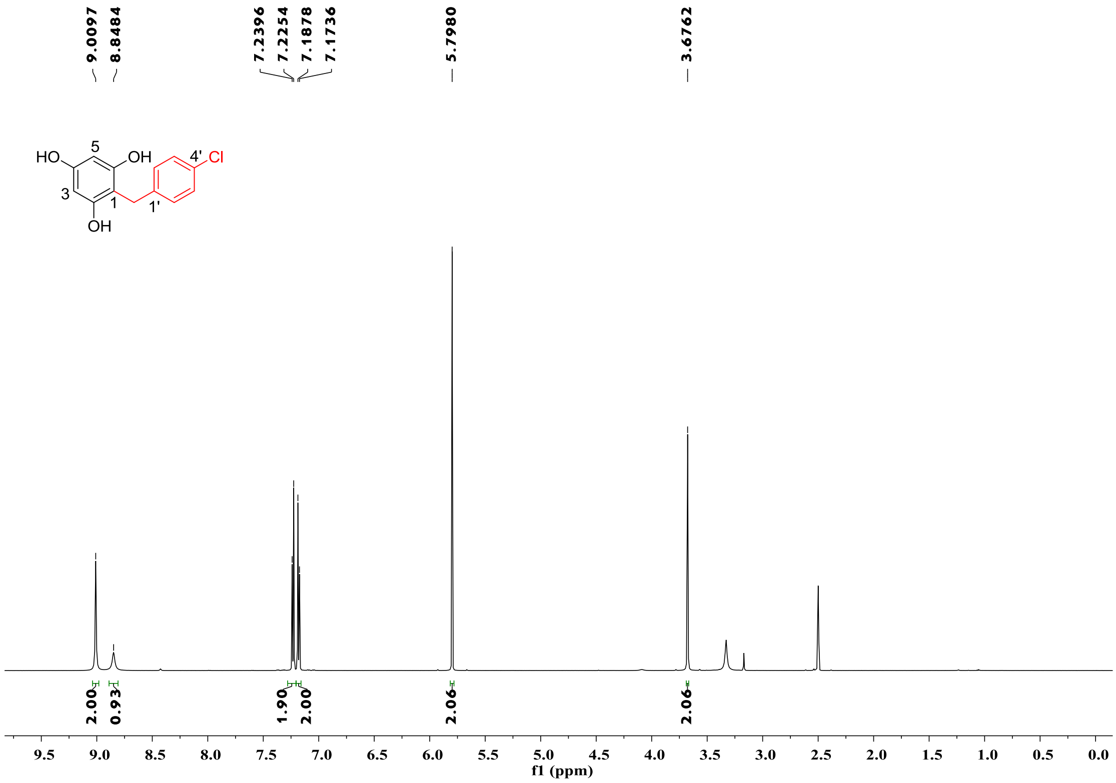

Supplementary Figure 117 ^{1}H NMR spectrum of **49** (DMSO-d_6, 600 MHz)

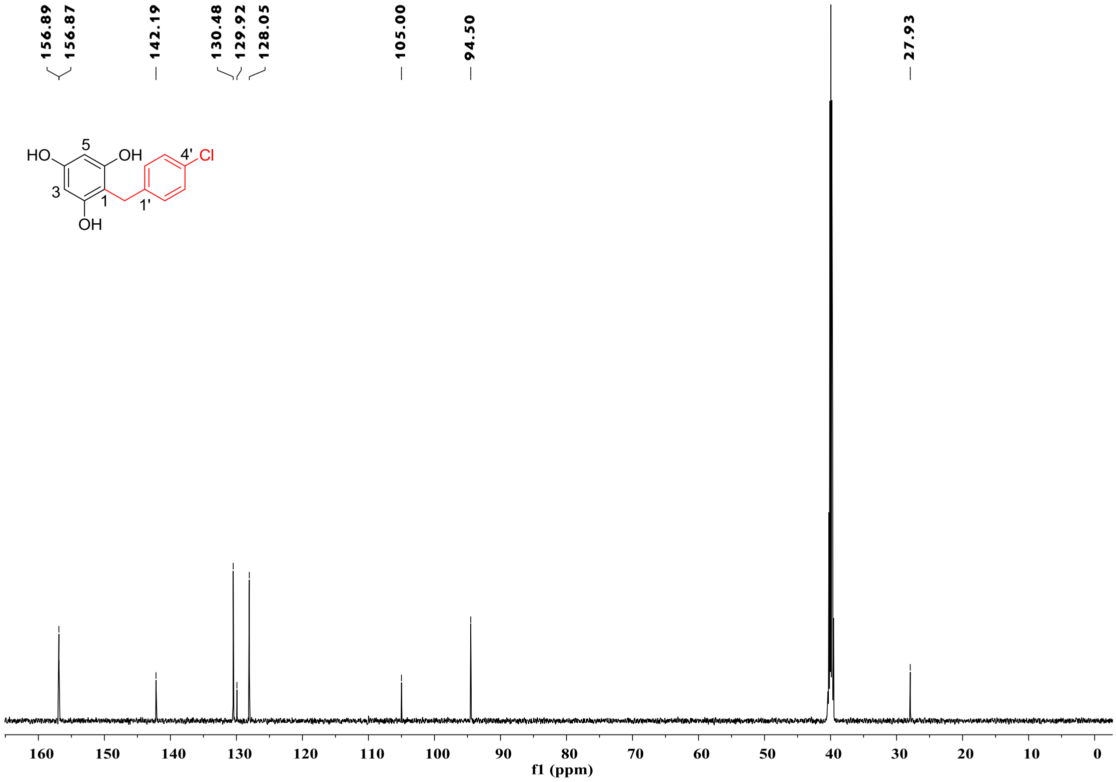

Supplementary Figure 118 ^{13}C NMR spectrum of **49** (DMSO-d_6, 150 MHz)

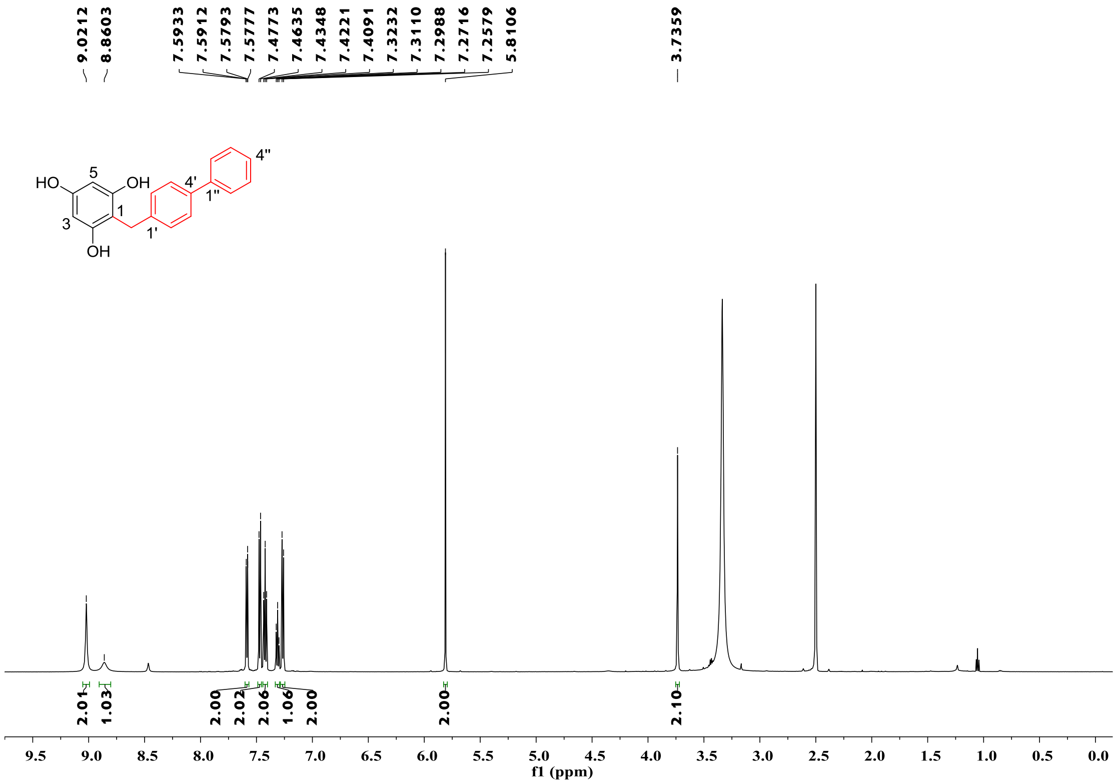

Supplementary Figure 119 ^{1}H NMR spectrum of **50** (DMSO-d_6, 600 MHz)

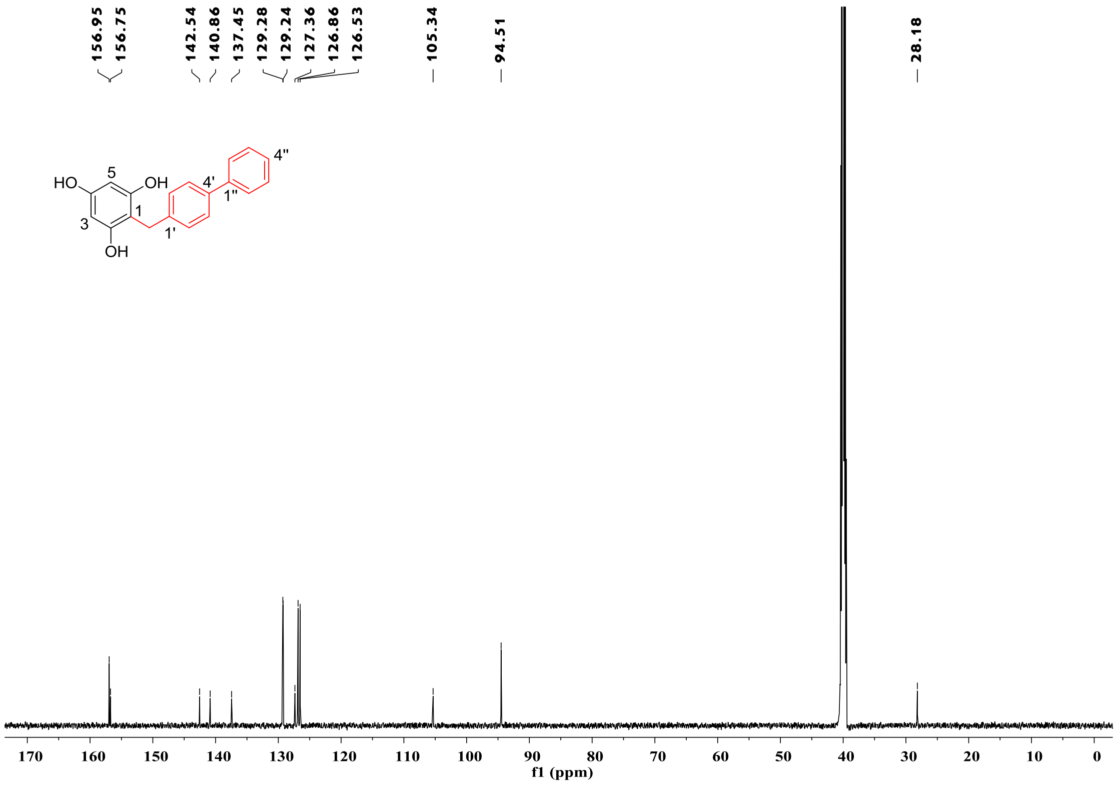

Supplementary Figure 120 ^{13}C NMR spectrum of **50** (DMSO-d_6, 150 MHz)

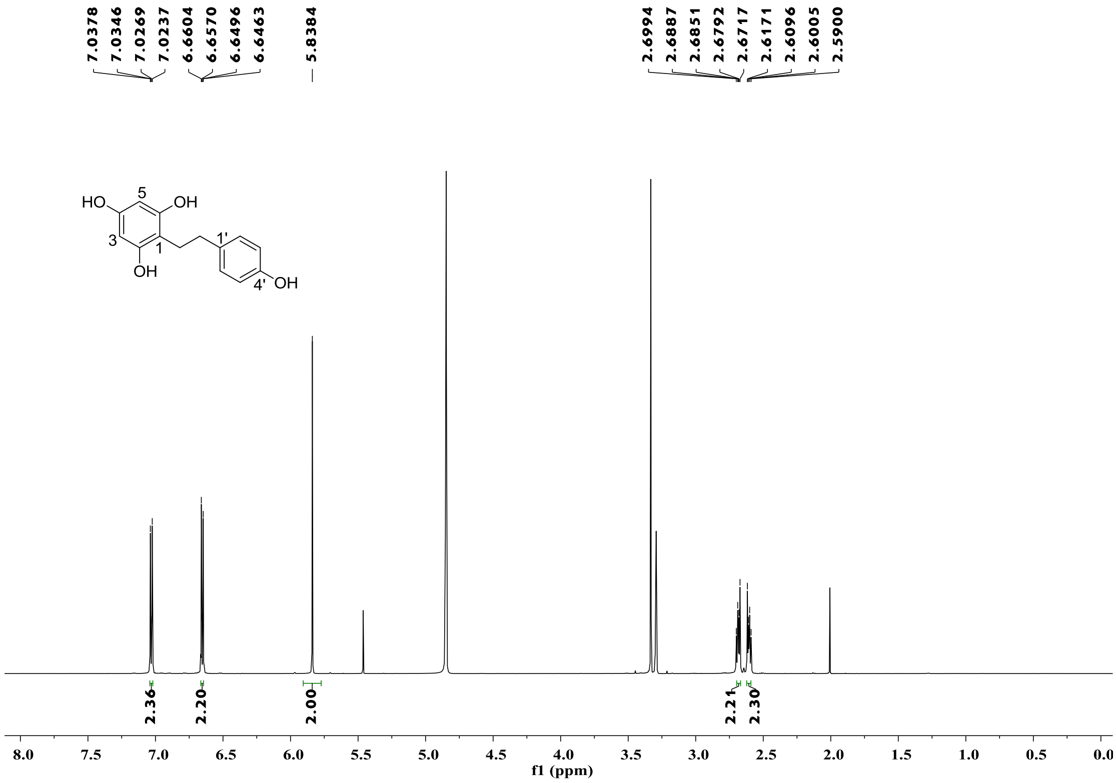

Supplementary Figure 121 ^{1}H NMR spectrum of **51** (Methanol-d_4, 600 MHz)

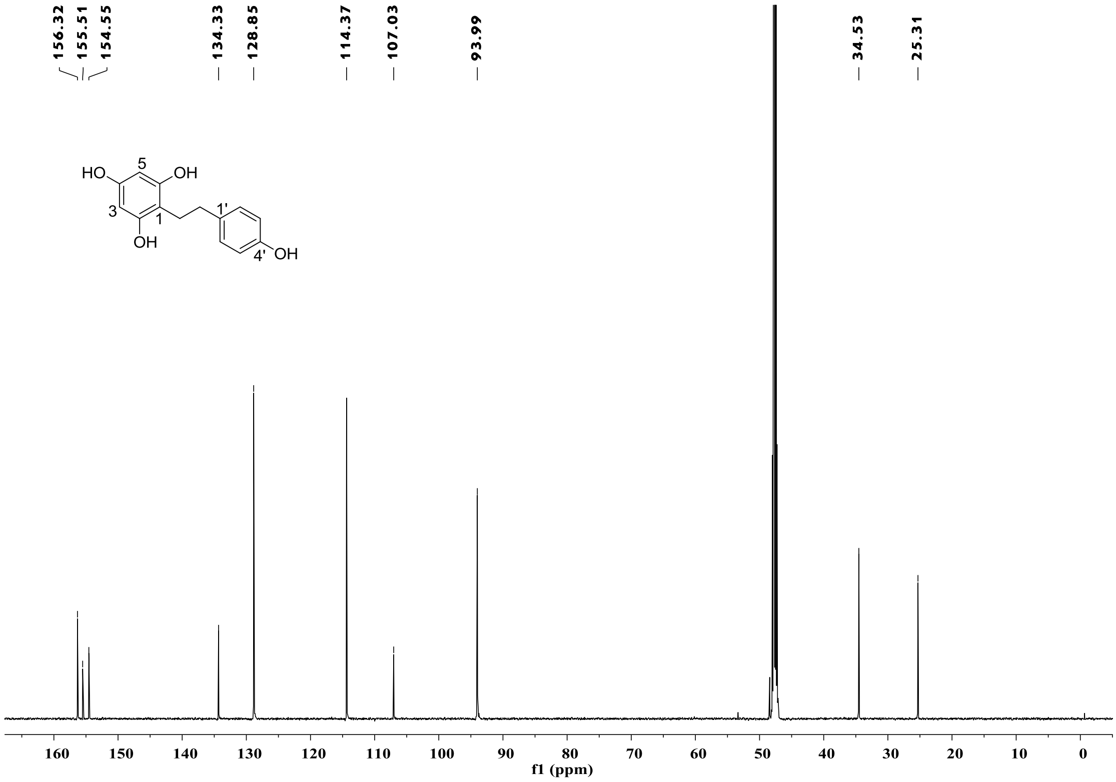

Supplementary Figure 122 ^{13}C NMR spectrum of **51** (Methanol-d_4, 150 MHz)

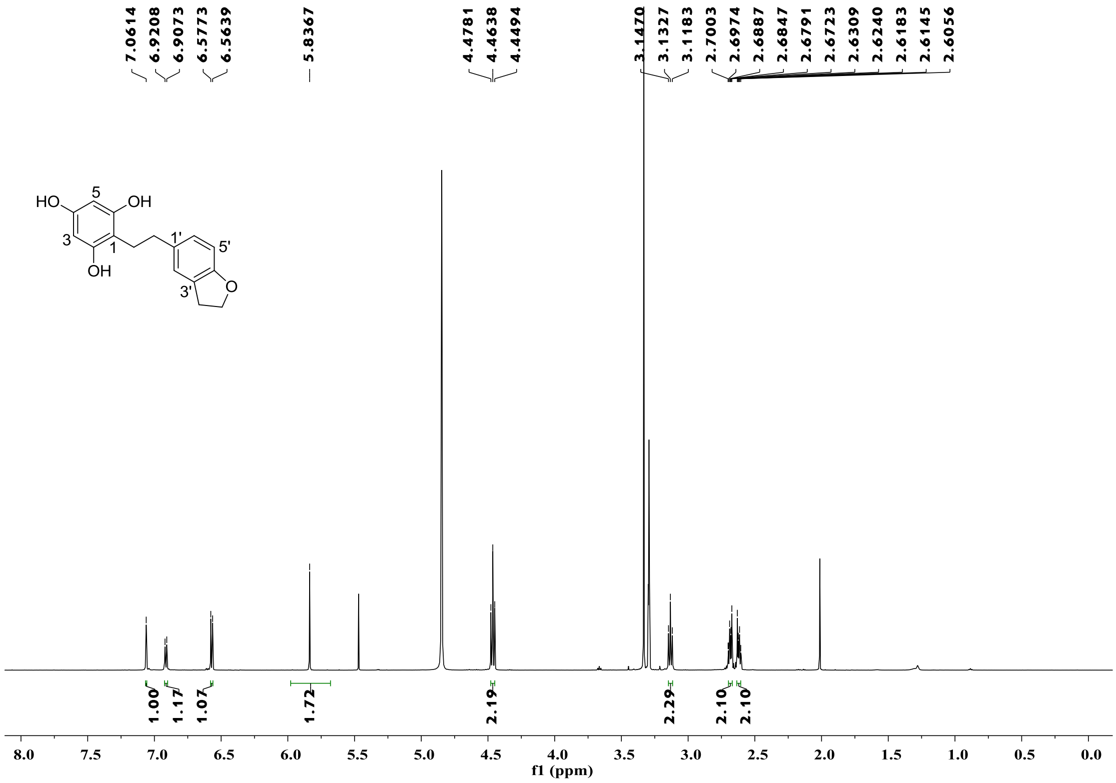

Supplementary Figure 123 ^{1}H NMR spectrum of **52** (Methanol-*d*$_4$, 600 MHz)

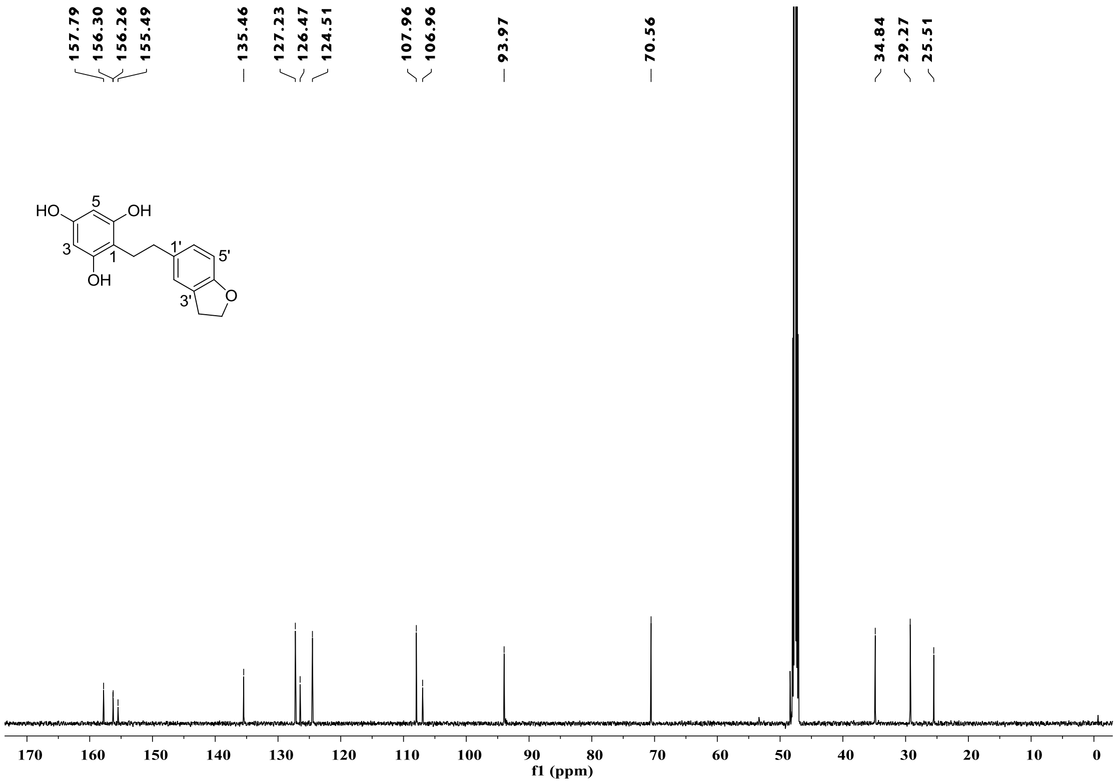

Supplementary Figure 124 ^{13}C NMR spectrum of **52** (Methanol-*d*$_4$, 150 MHz)

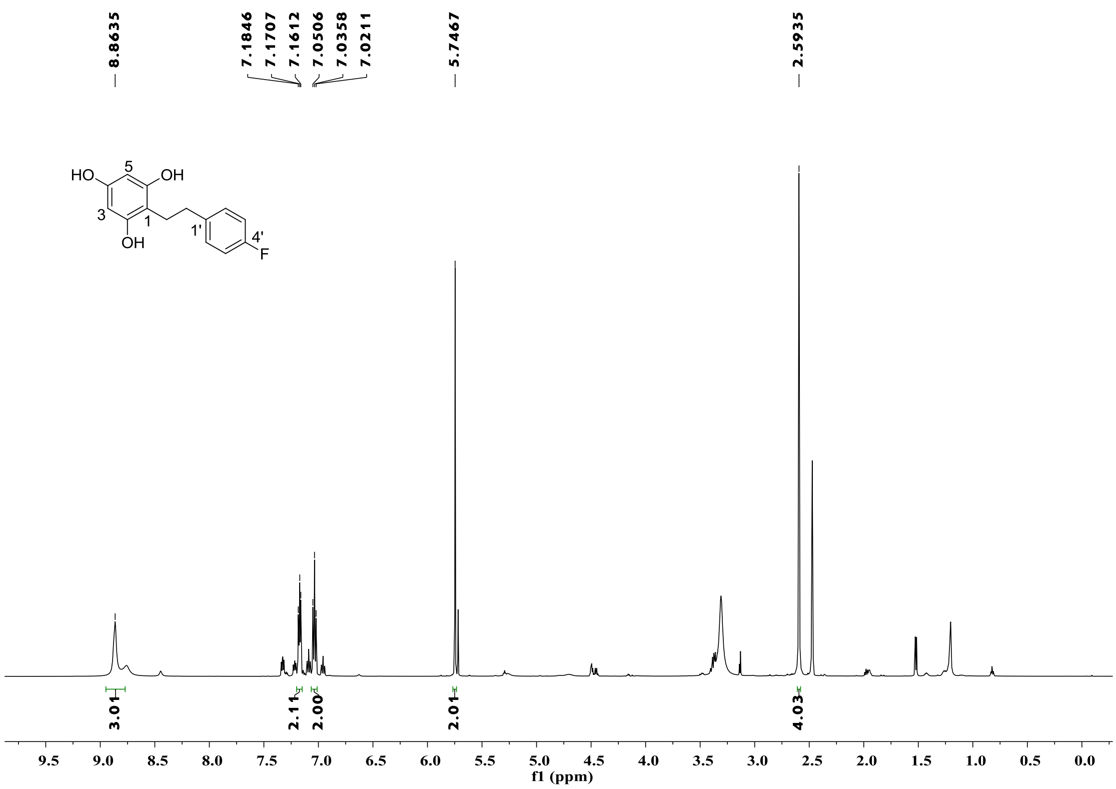

Supplementary Figure 125 ^{1}H NMR spectrum of **53** (DMSO-d_6, 600 MHz)

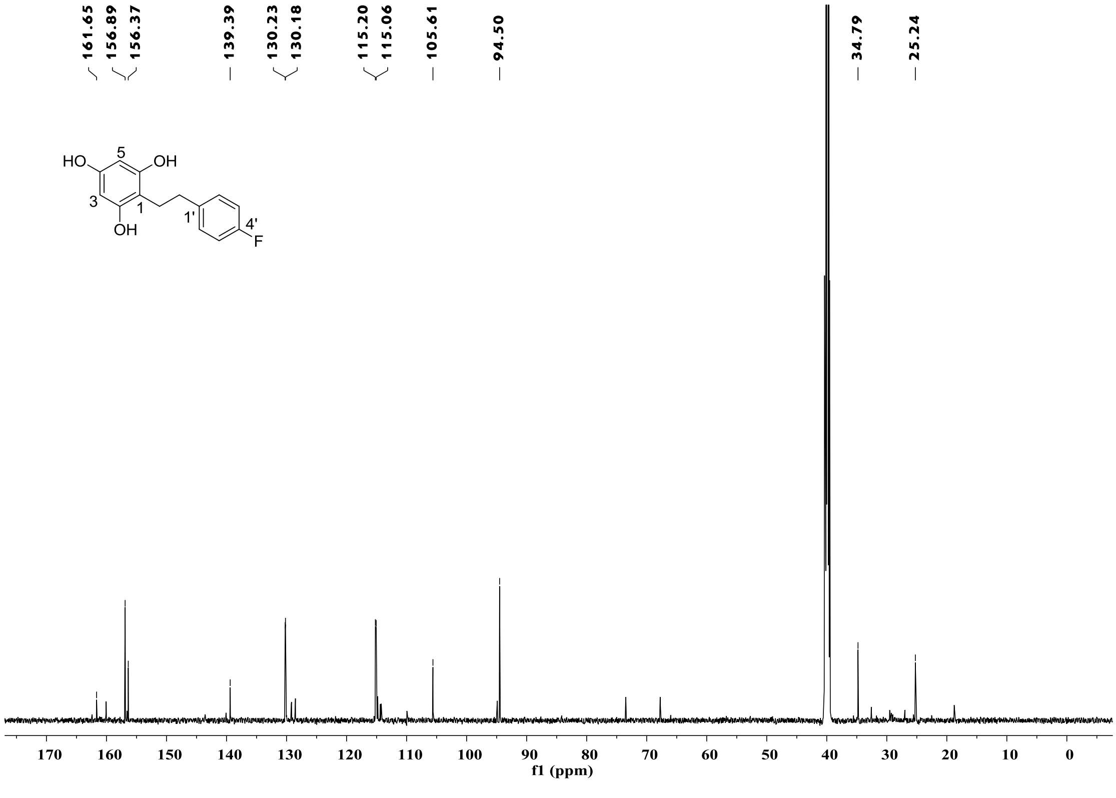

Supplementary Figure 126 ^{13}C NMR spectrum of **53** (DMSO-d_6, 150 MHz)

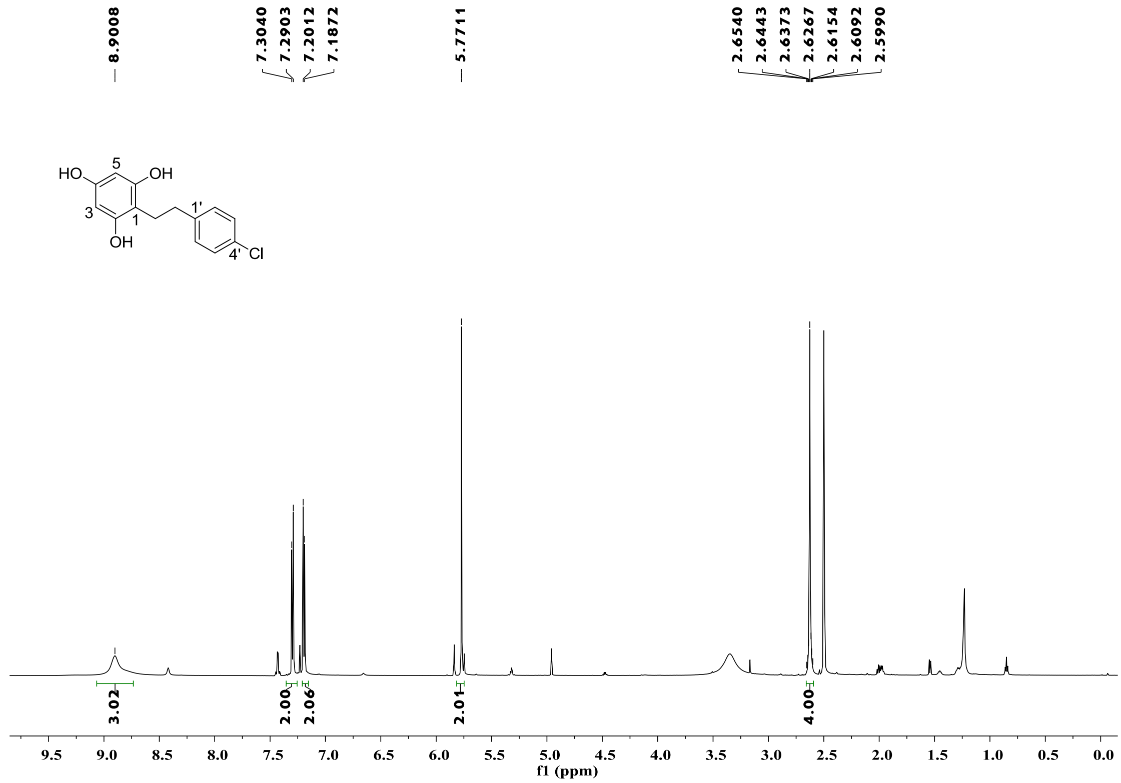

Supplementary Figure 127 ^{1}H NMR spectrum of **54** (DMSO-d_6, 600 MHz)

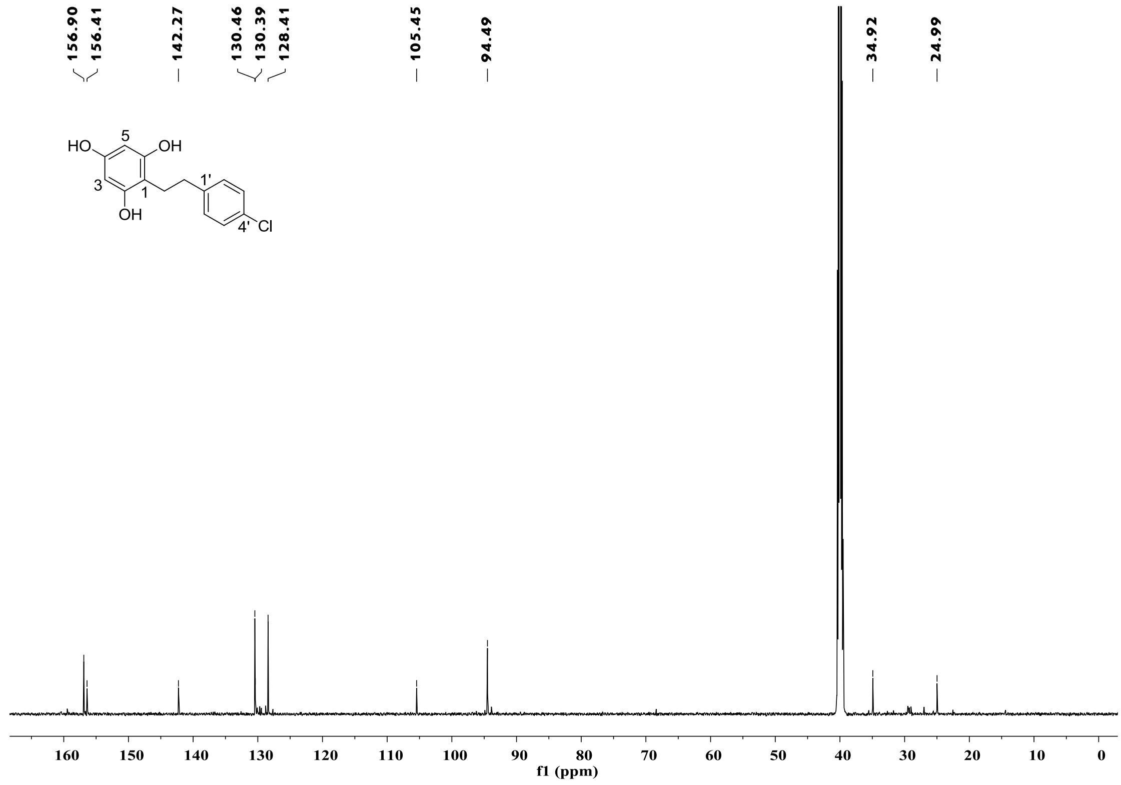

Supplementary Figure 128 ^{13}C NMR spectrum of **54** (DMSO-d_6, 150 MHz)

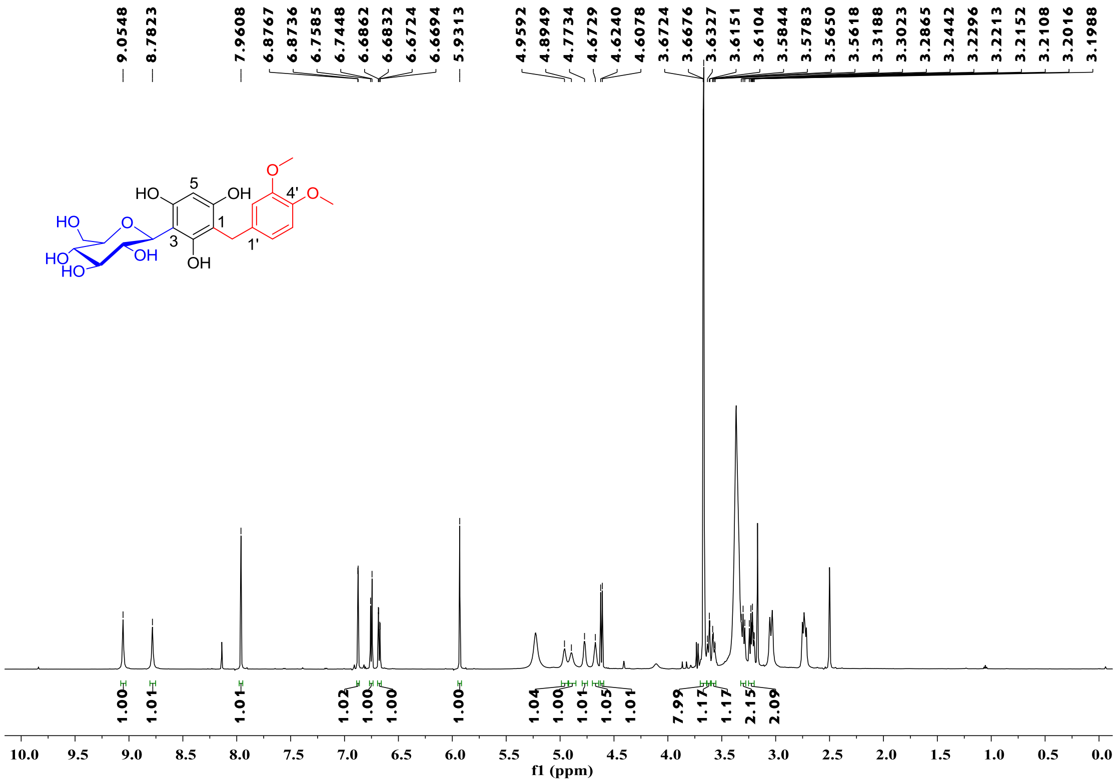

Supplementary Figure 129 ^{1}H NMR spectrum of **20a** (DMSO-d_6, 600 MHz)

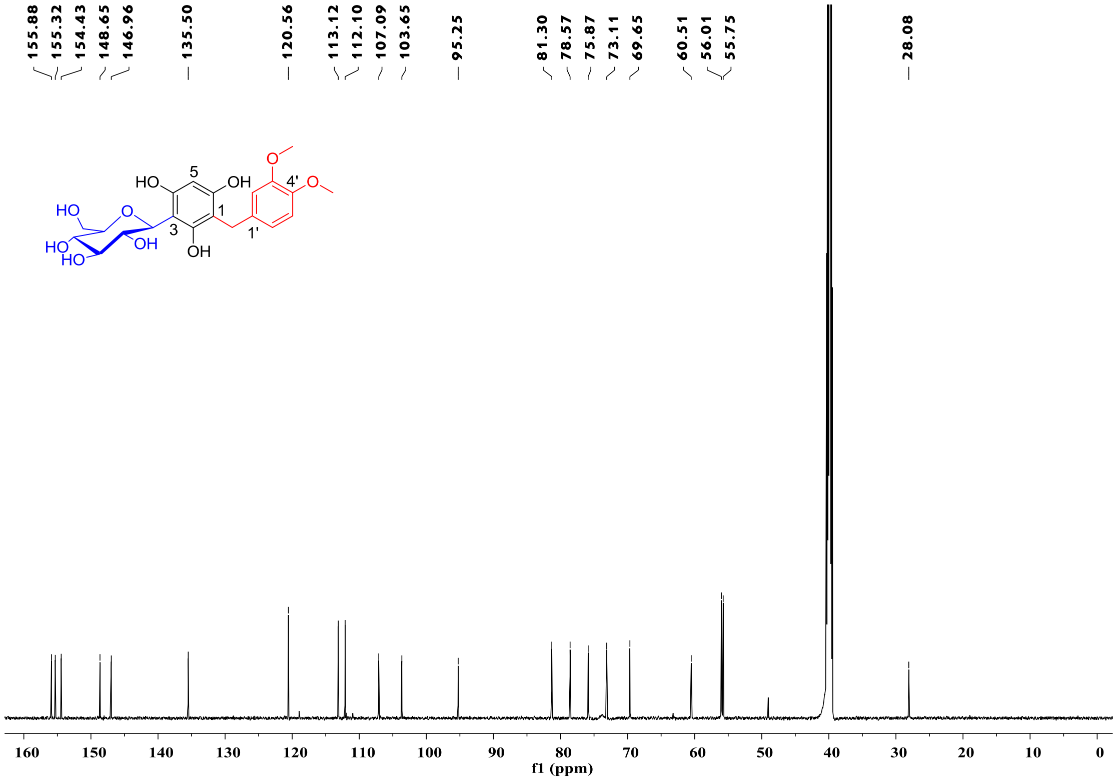

Supplementary Figure 130 ^{13}C NMR spectrum of **20a** (DMSO-d_6, 150 MHz)

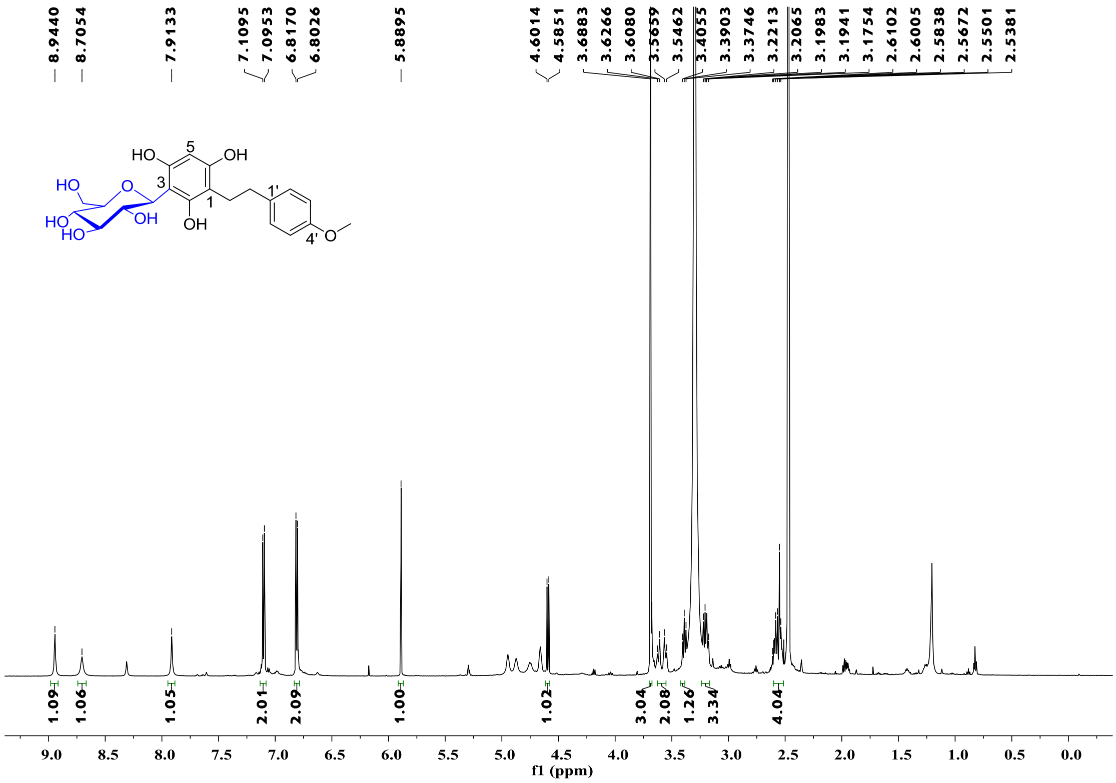

Supplementary Figure 131 ^{1}H NMR spectrum of **21a** (DMSO-d_6, 600 MHz)

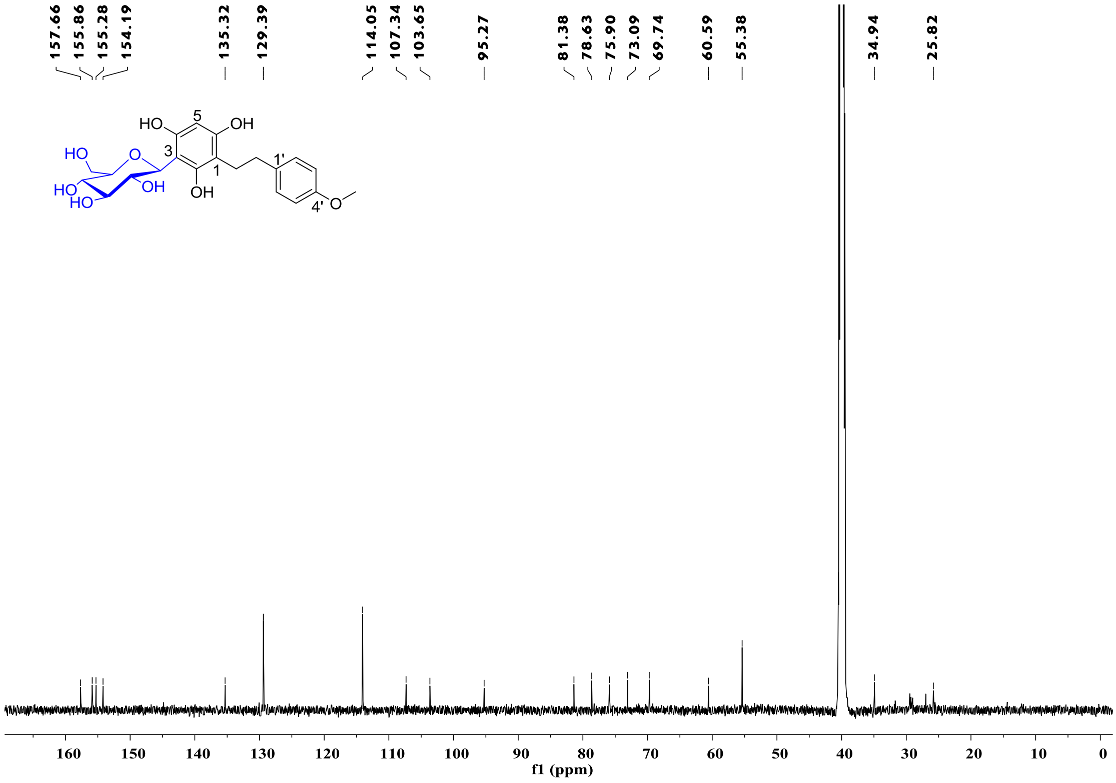

Supplementary Figure 132 ^{13}C NMR spectrum of **21a** (DMSO-d_6, 150 MHz)

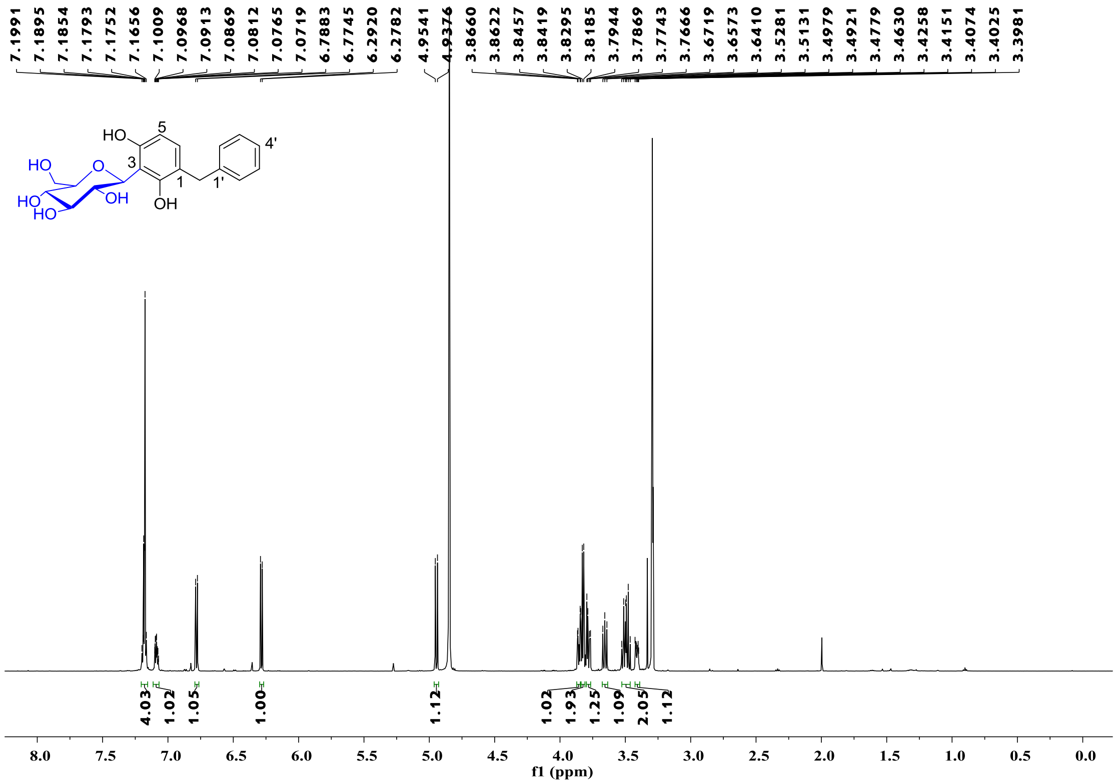

Supplementary Figure 133 ^{1}H NMR spectrum of **28a** (Methanol-d_4, 600 MHz)

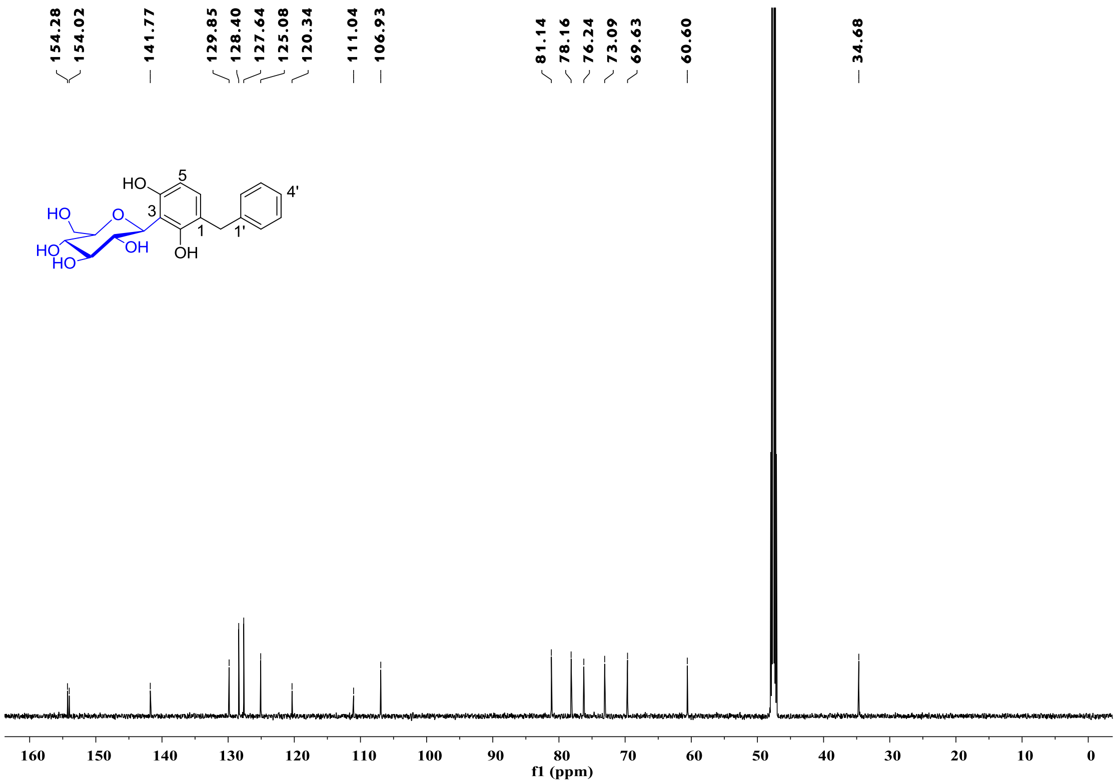

Supplementary Figure 134 ^{13}C NMR spectrum of **28a** (Methanol-d_4, 150 MHz)

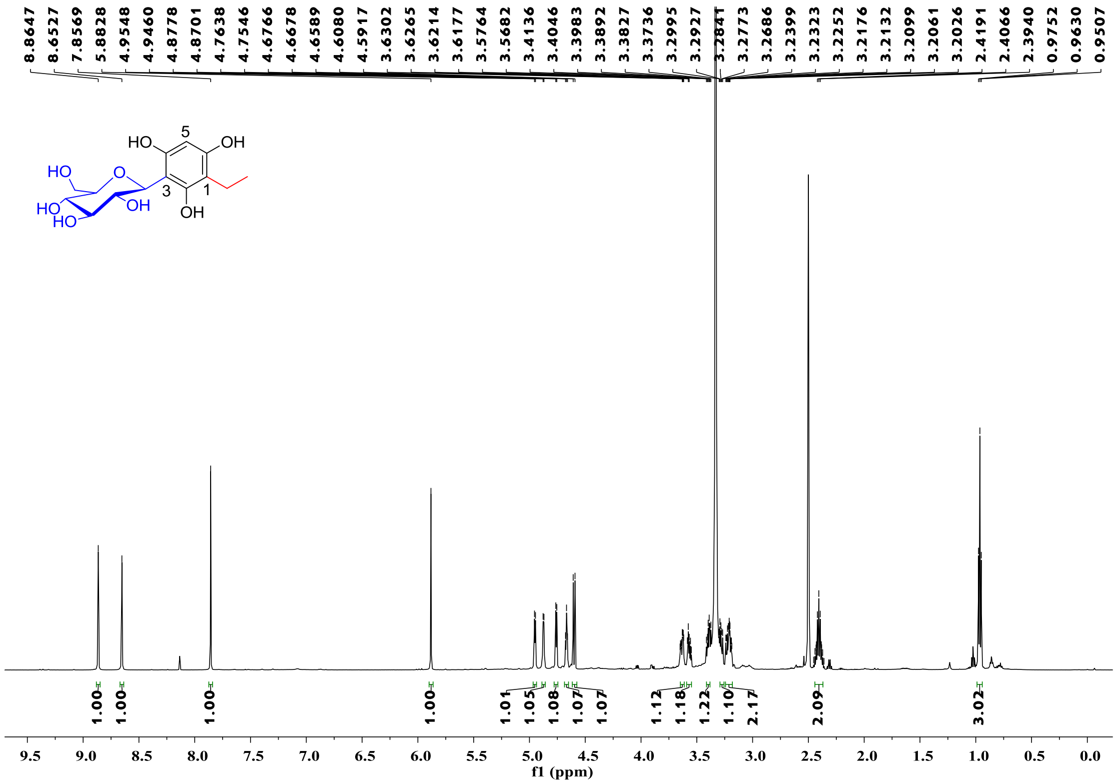

Supplementary Figure 135 ^{1}H NMR spectrum of **30a** (DMSO-d_6, 600 MHz)

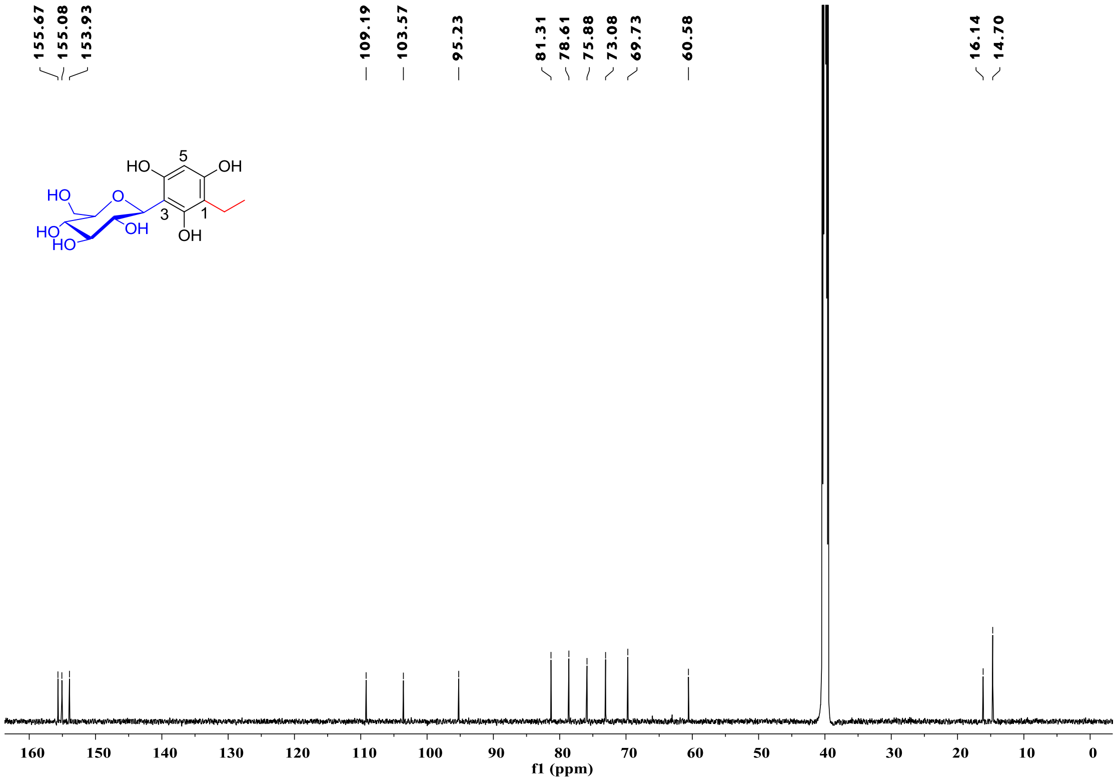

Supplementary Figure 136 ^{13}C NMR spectrum of **30a** (DMSO-d_6, 150 MHz)

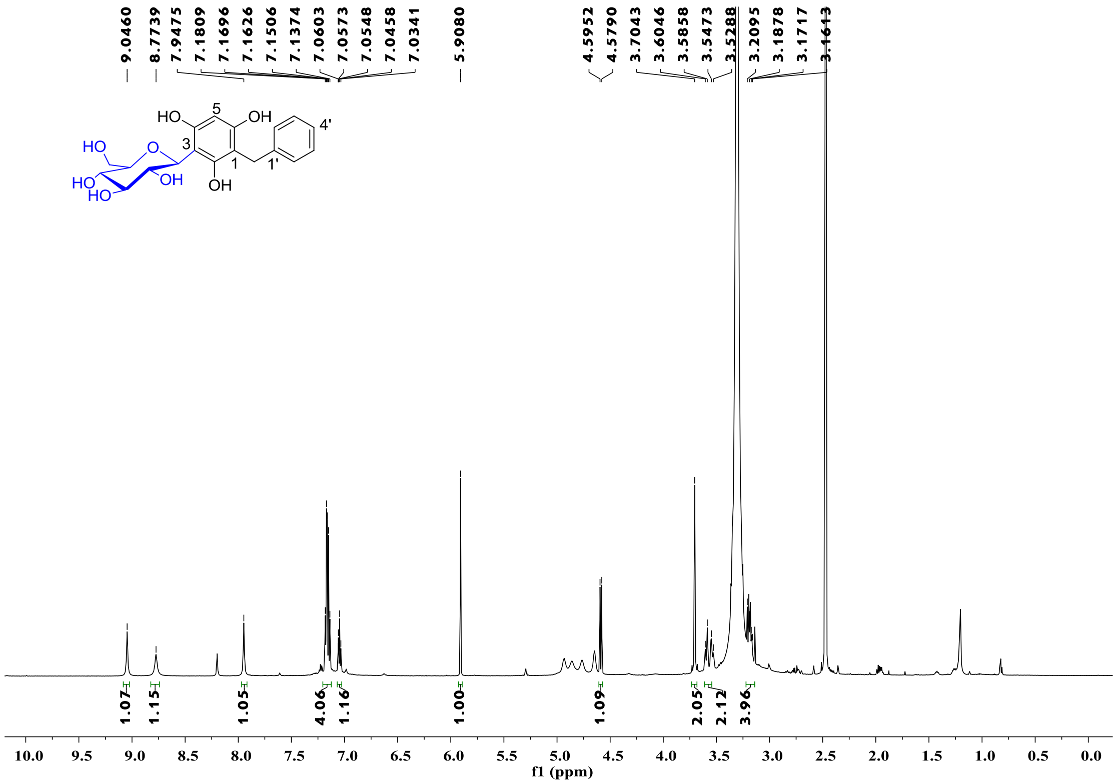

Supplementary Figure 137 ^{1}H NMR spectrum of **38a** (DMSO-d_6, 600 MHz)

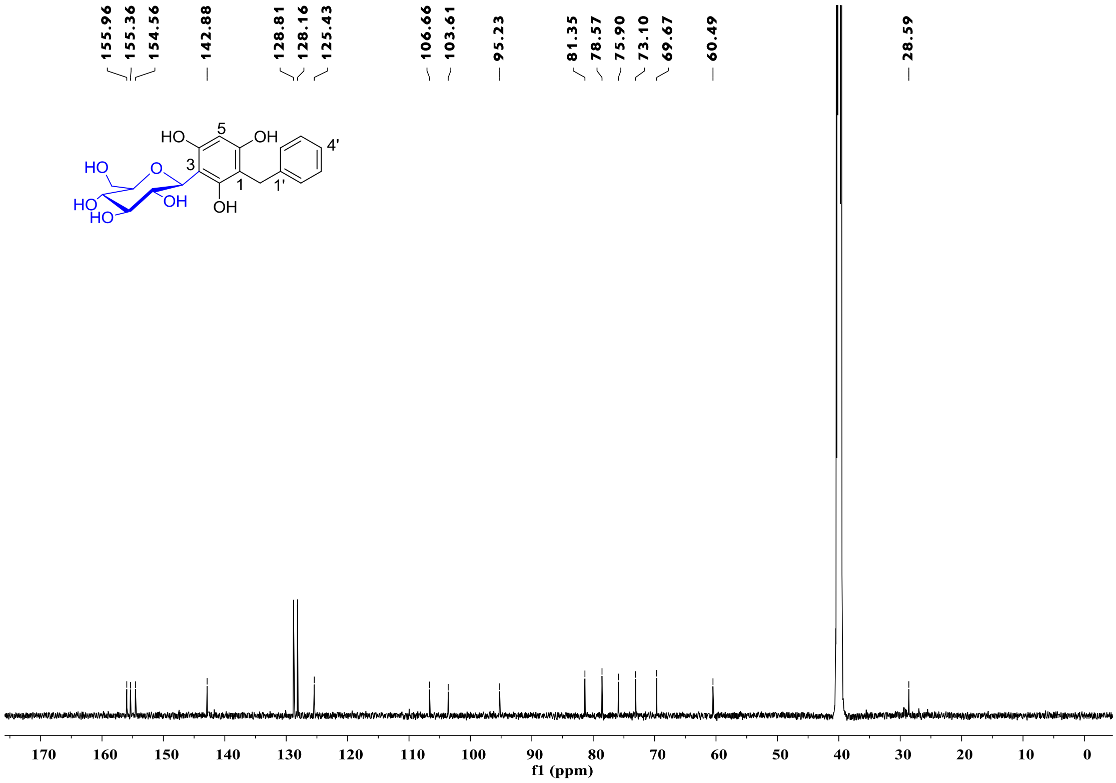

Supplementary Figure 138 ^{13}C NMR spectrum of **38a** (DMSO-d_6, 150 MHz)

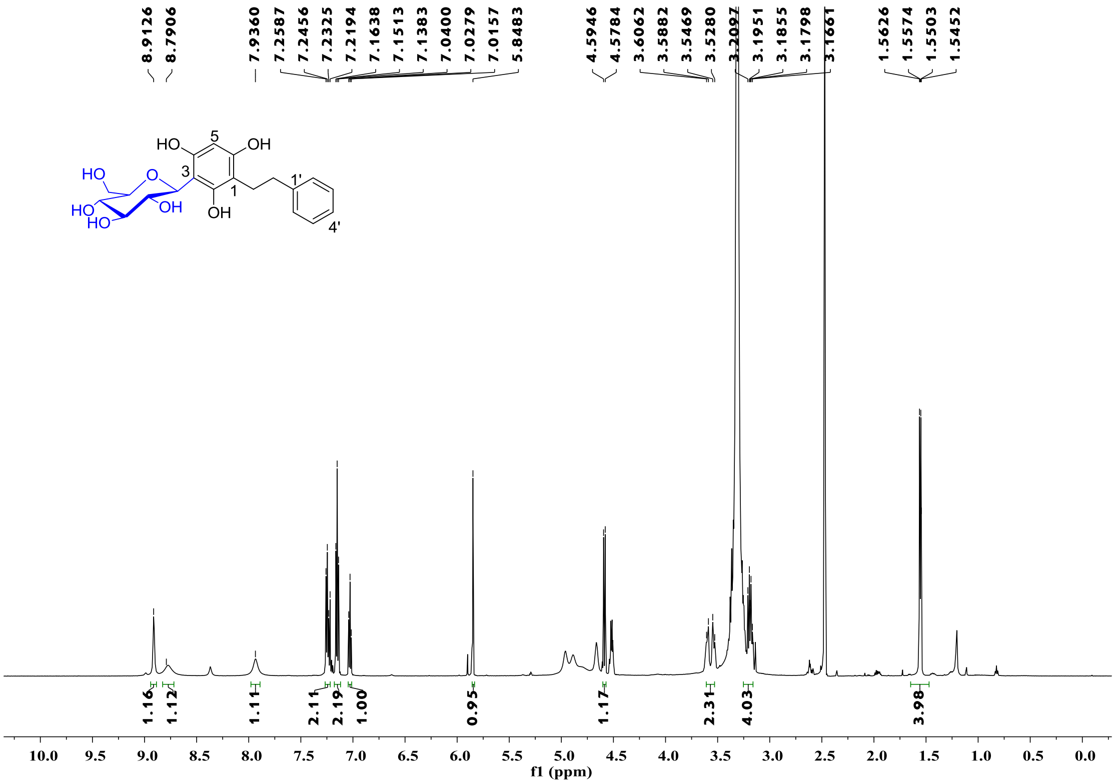

Supplementary Figure 139 ^{1}H NMR spectrum of **39a** (DMSO-d_6, 600 MHz)

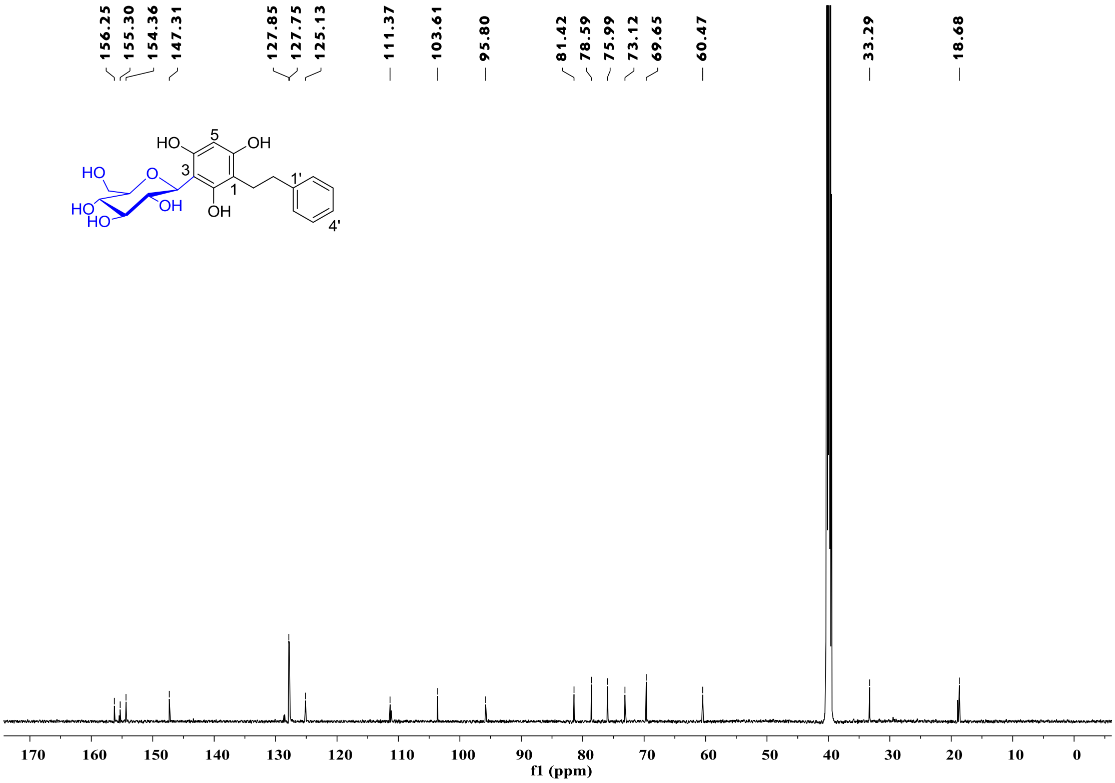

Supplementary Figure 140 ^{13}C NMR spectrum of **39a** (DMSO-d_6, 150 MHz)

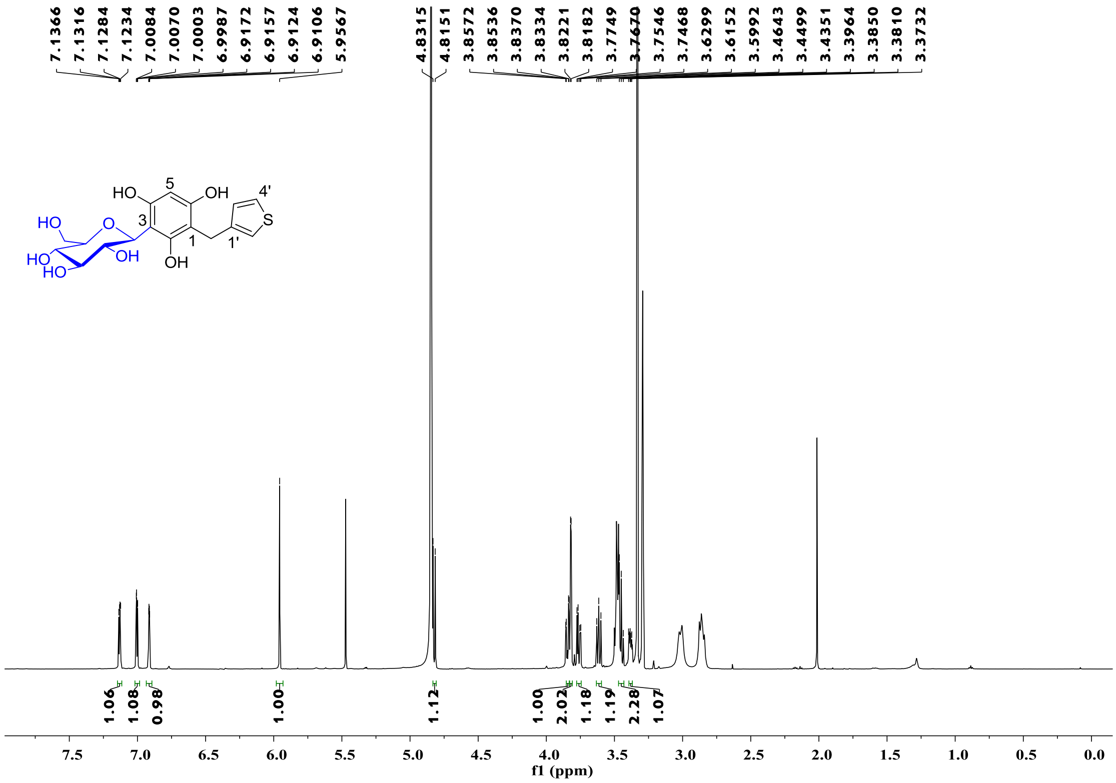

Supplementary Figure 141 ^{1}H NMR spectrum of **42a** (DMSO-d_6, 600 MHz)

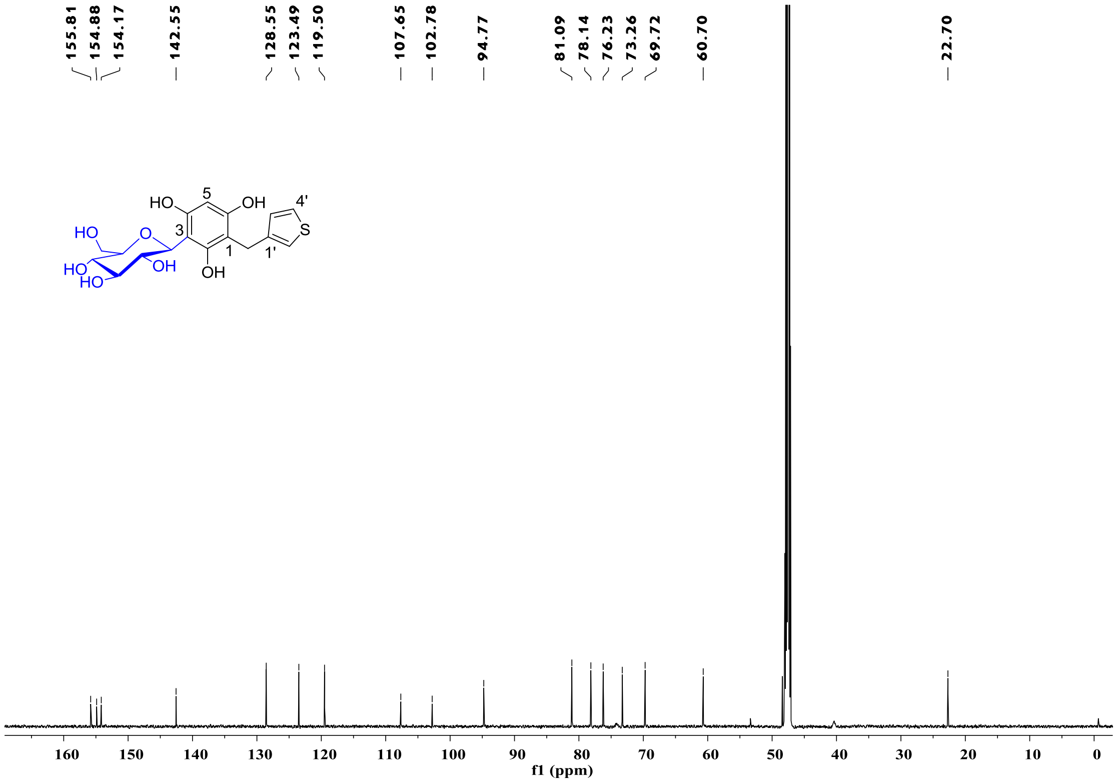

Supplementary Figure 142 ^{13}C NMR spectrum of **42a** (DMSO-d_6, 150 MHz)

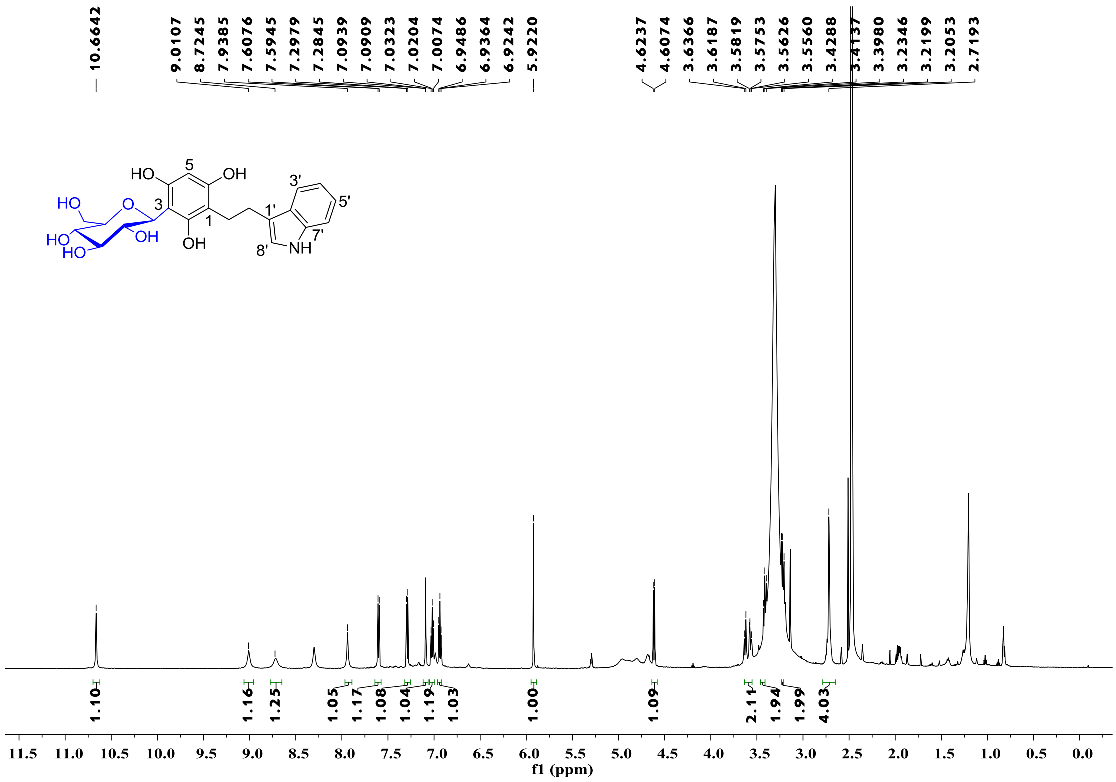

Supplementary Figure 143 ^{1}H NMR spectrum of **43a** (DMSO-d_6, 600 MHz)

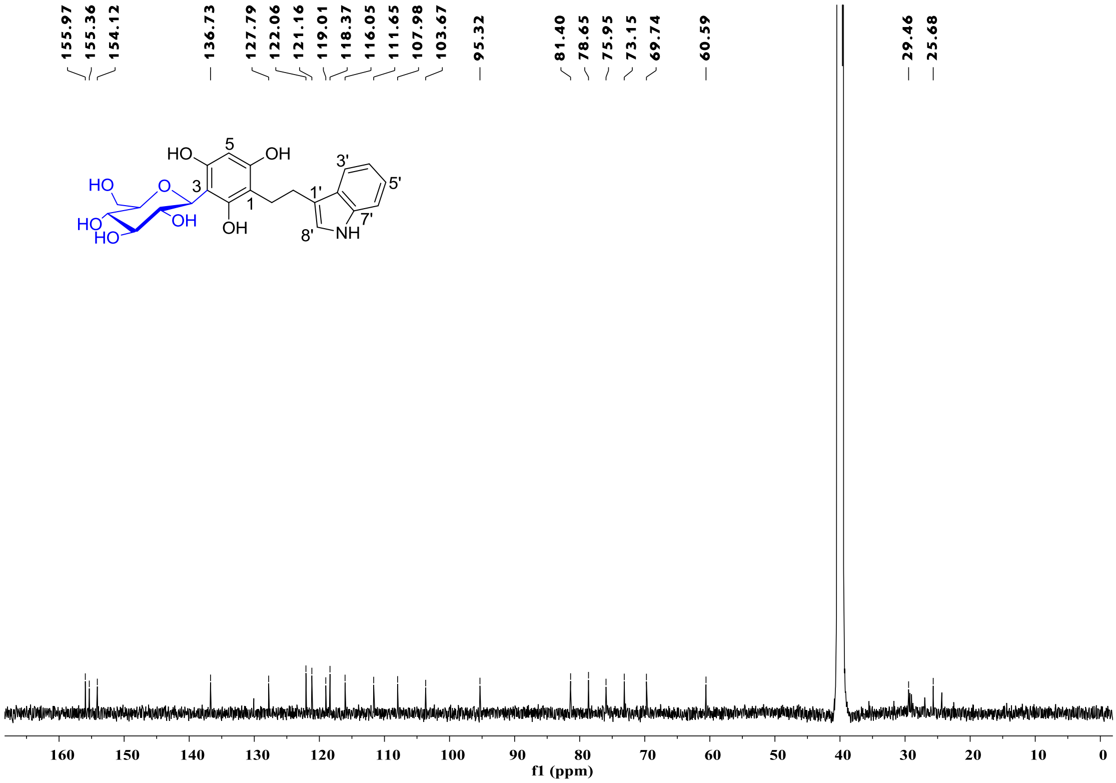

Supplementary Figure 144 ^{13}C NMR spectrum of **43a** (DMSO-d_6, 150 MHz)

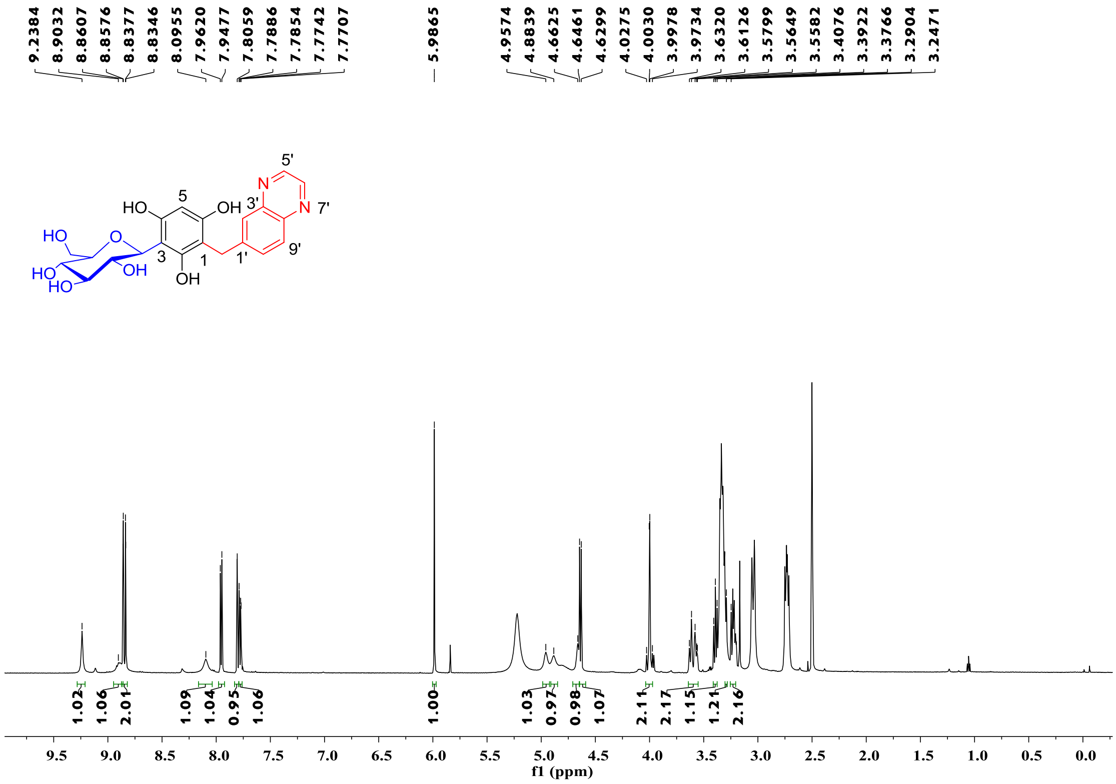

Supplementary Figure 145 ^{1}H NMR spectrum of **44a** (DMSO-d_6, 600 MHz)

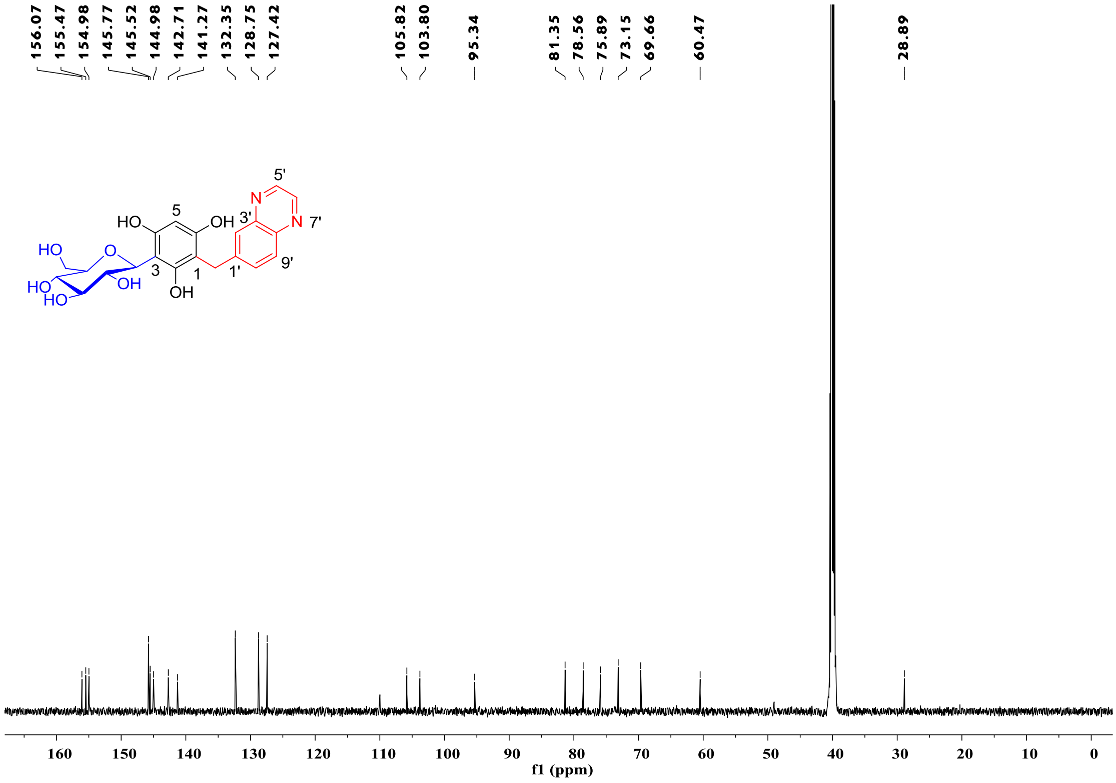

Supplementary Figure 146 ^{13}C NMR spectrum of **44a** (DMSO-d_6, 150 MHz)

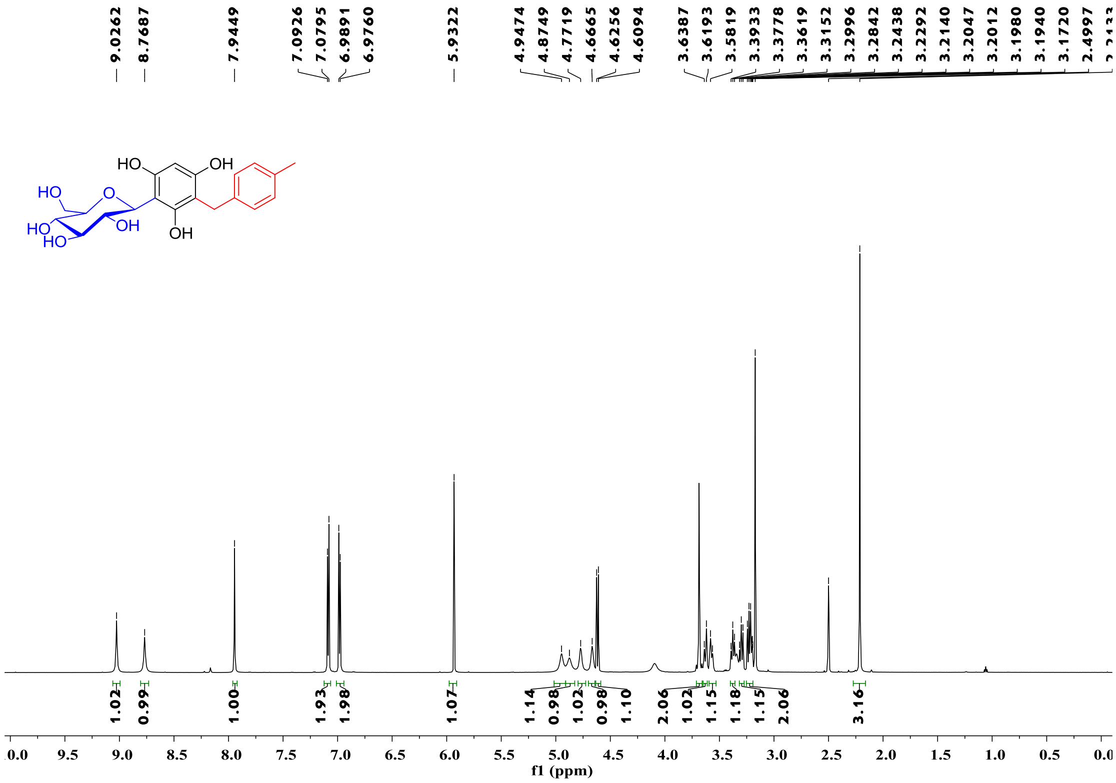

Supplementary Figure 147 ^{1}H NMR spectrum of **46a** (DMSO-d_6, 600 MHz)

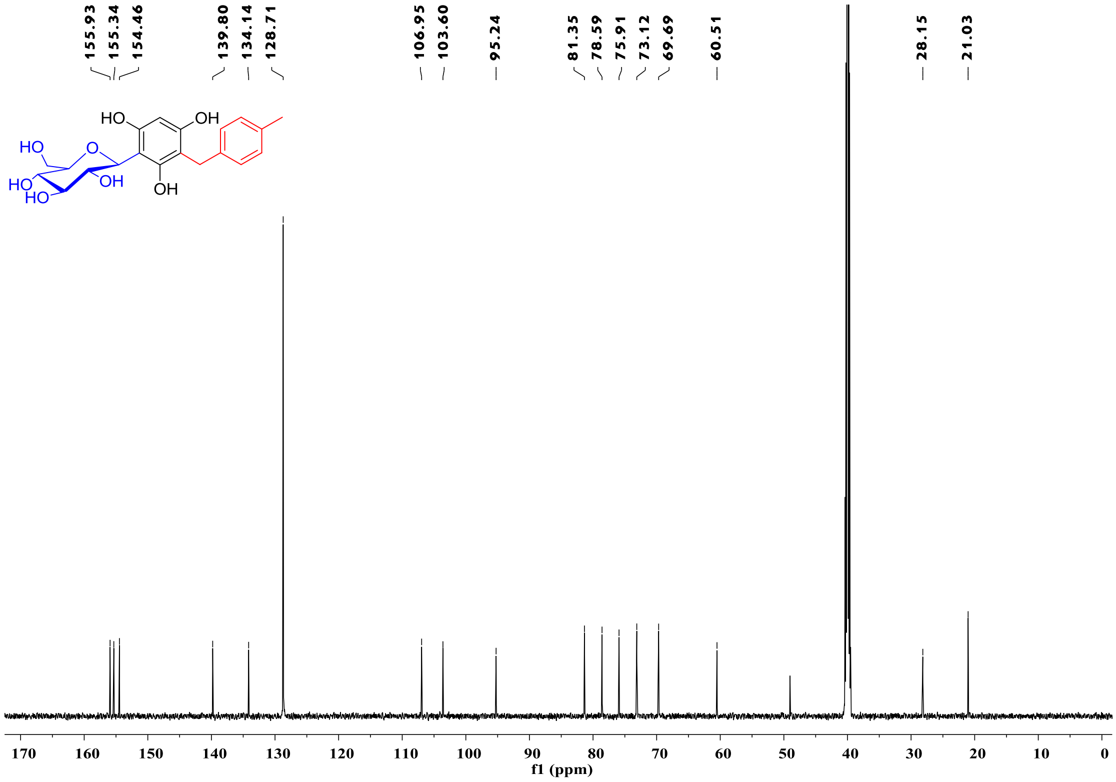

Supplementary Figure 148 ^{13}C NMR spectrum of **46a** (DMSO-d_6, 150 MHz)

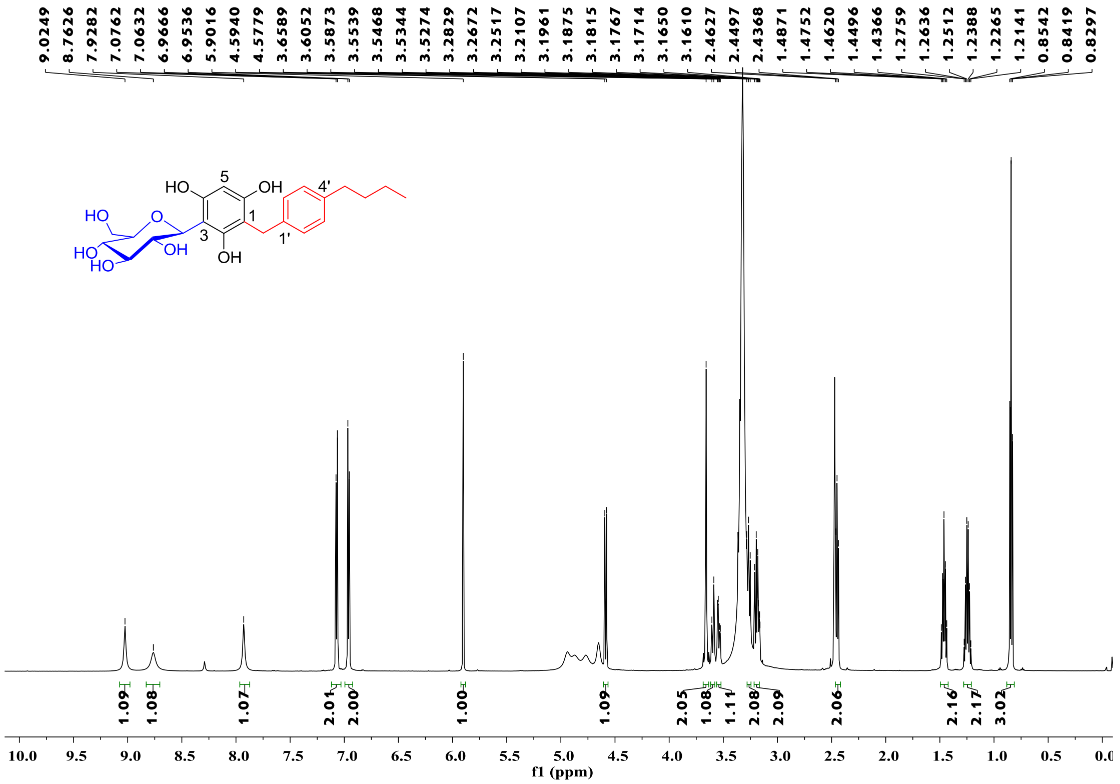

Supplementary Figure 149 ^{1}H NMR spectrum of **47a** (DMSO-d_6, 600 MHz)

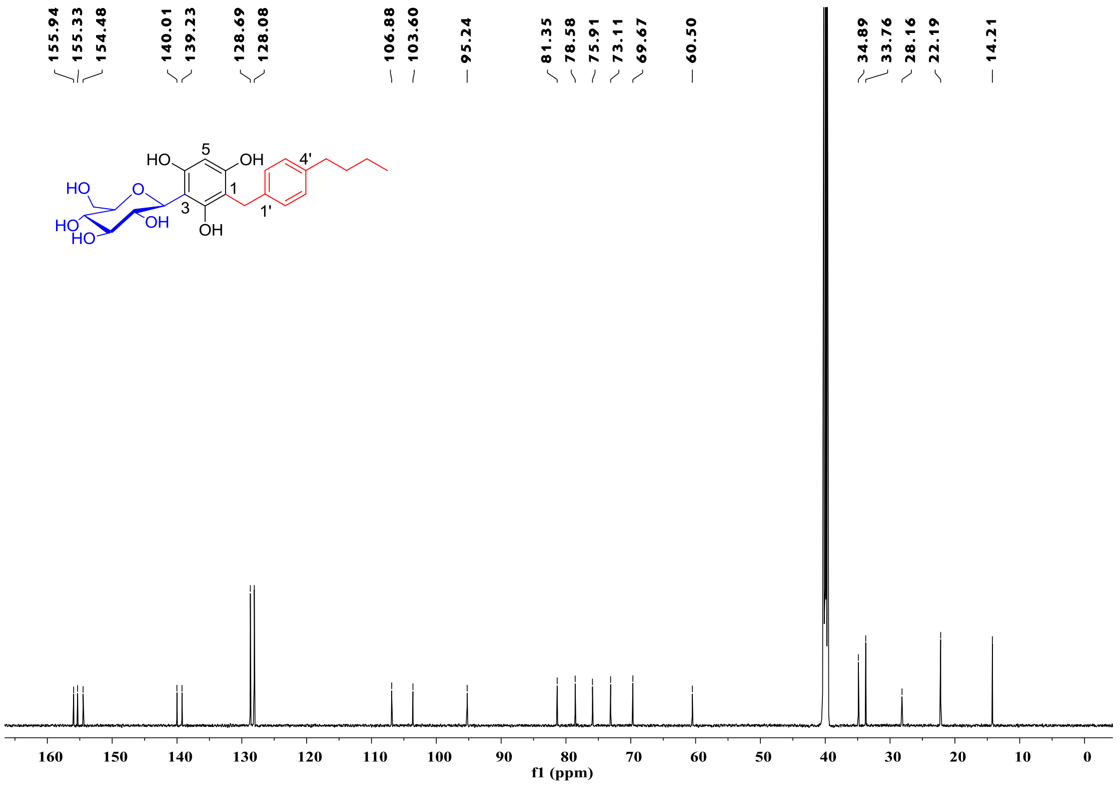

Supplementary Figure 150 ^{13}C NMR spectrum of **47a** (DMSO-d_6, 150 MHz)

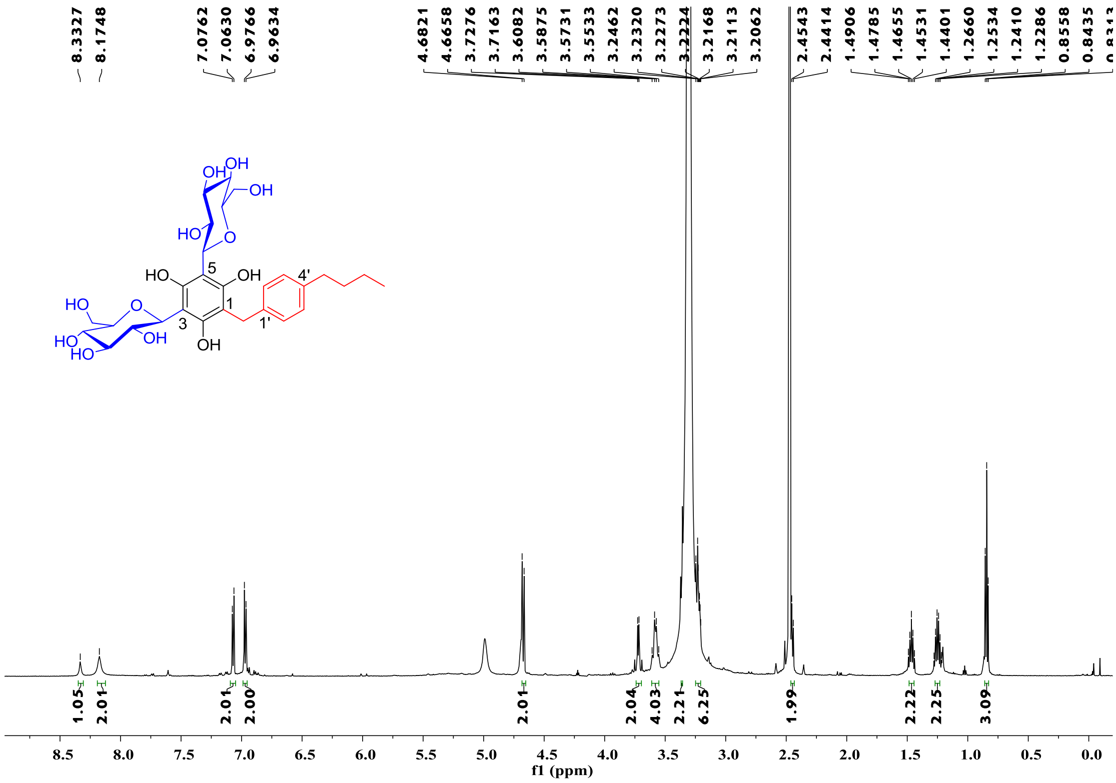

Supplementary Figure 151 ^{1}H NMR spectrum of **47aa** (DMSO-d_6, 600 MHz)

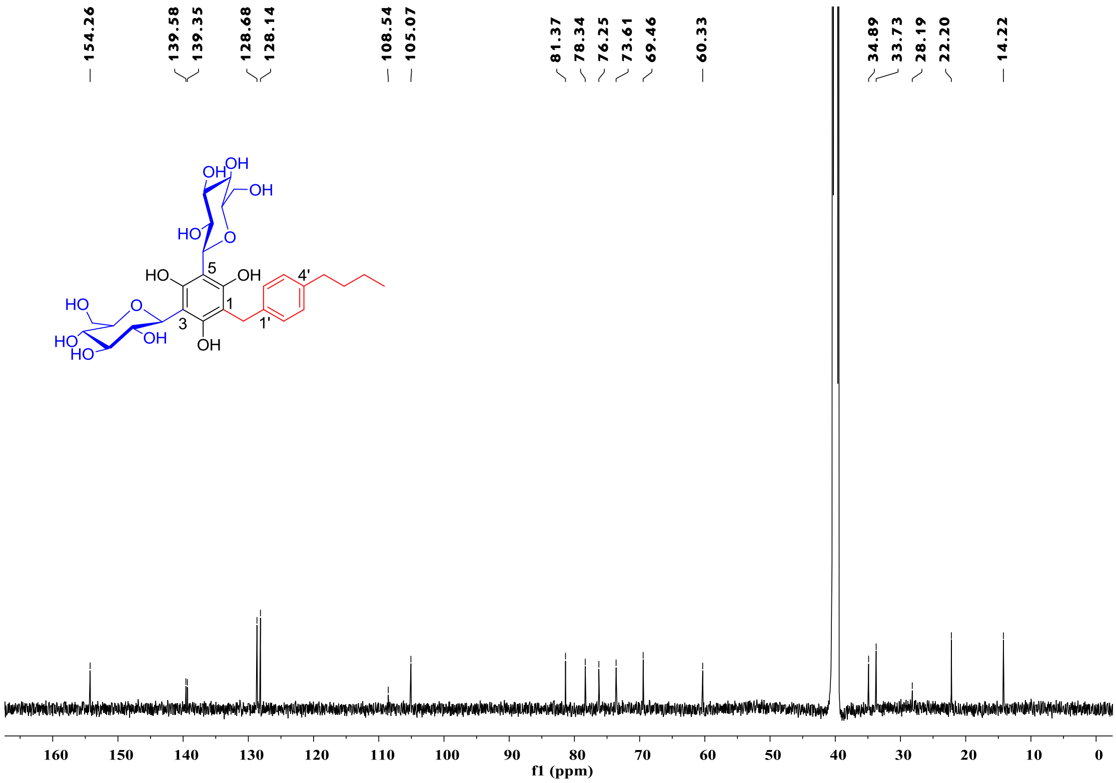

Supplementary Figure 152 ^{13}C NMR spectrum of **47aa** (DMSO-d_6, 150 MHz)

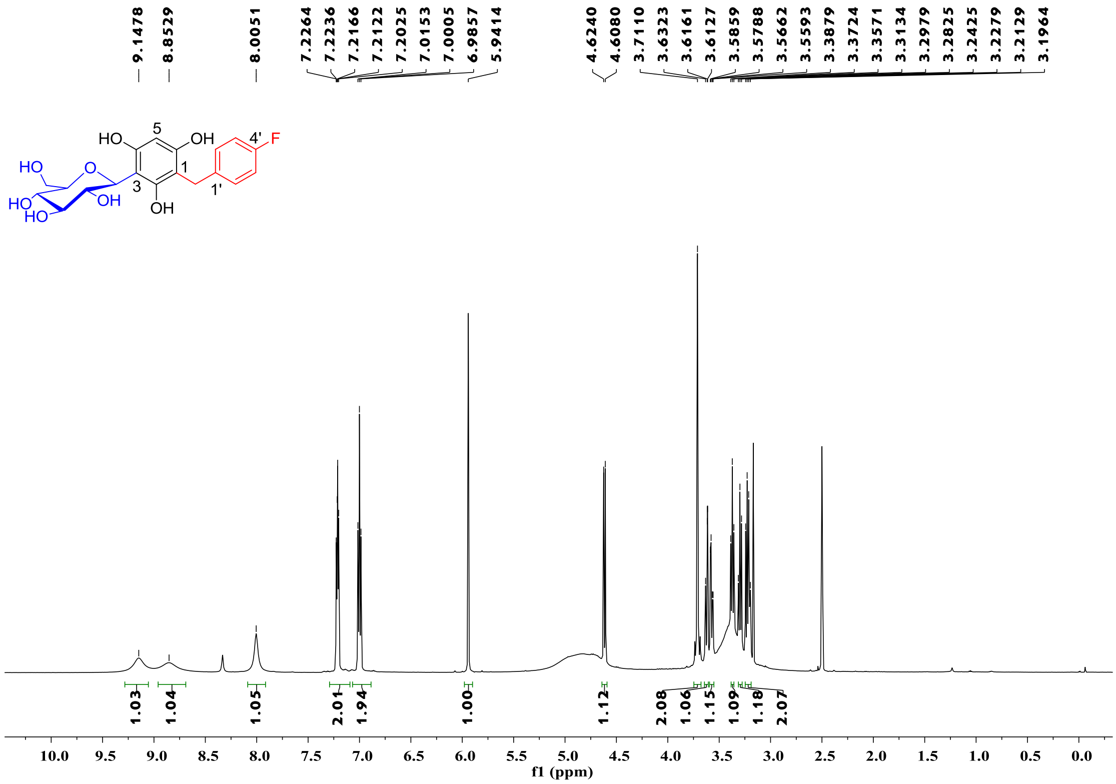

Supplementary Figure 153 ^{1}H NMR spectrum of **48a** (DMSO-d_6, 600 MHz)

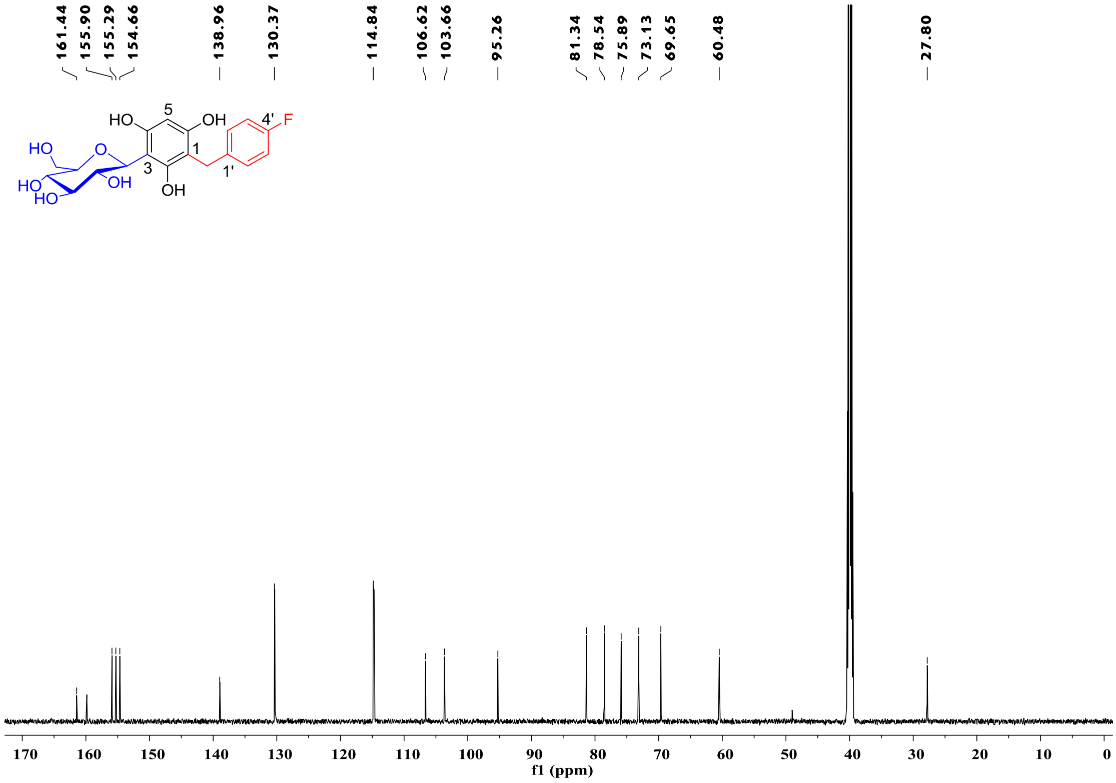

Supplementary Figure 154 ^{13}C NMR spectrum of **48a** (DMSO-d_6, 150 MHz)

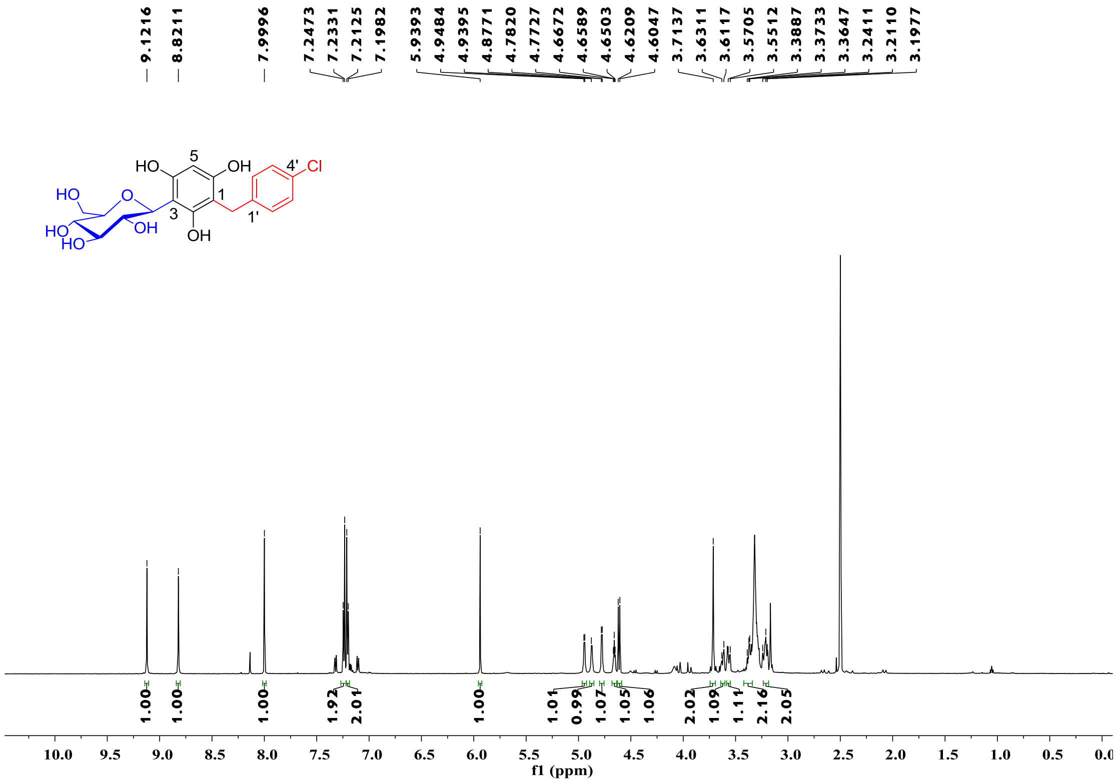

Supplementary Figure 155 ^{1}H NMR spectrum of **49a** (DMSO-d_6, 600 MHz)

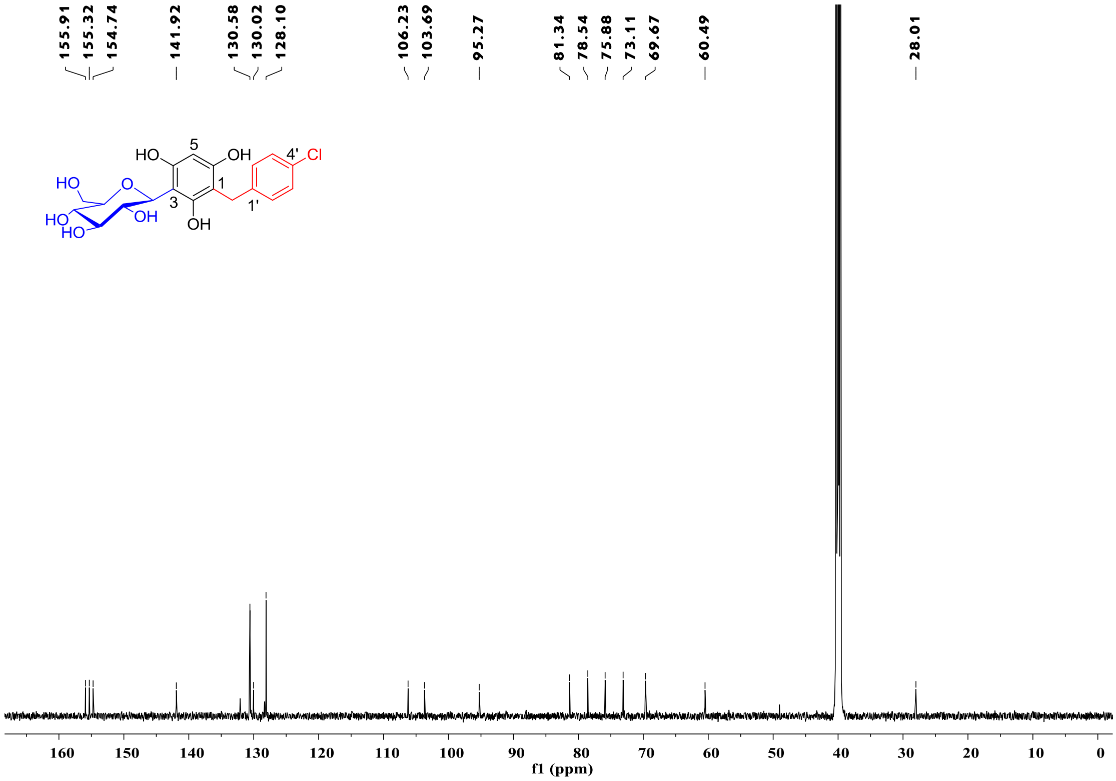

Supplementary Figure 156 ^{13}C NMR spectrum of **49a** (DMSO-d_6, 150 MHz)

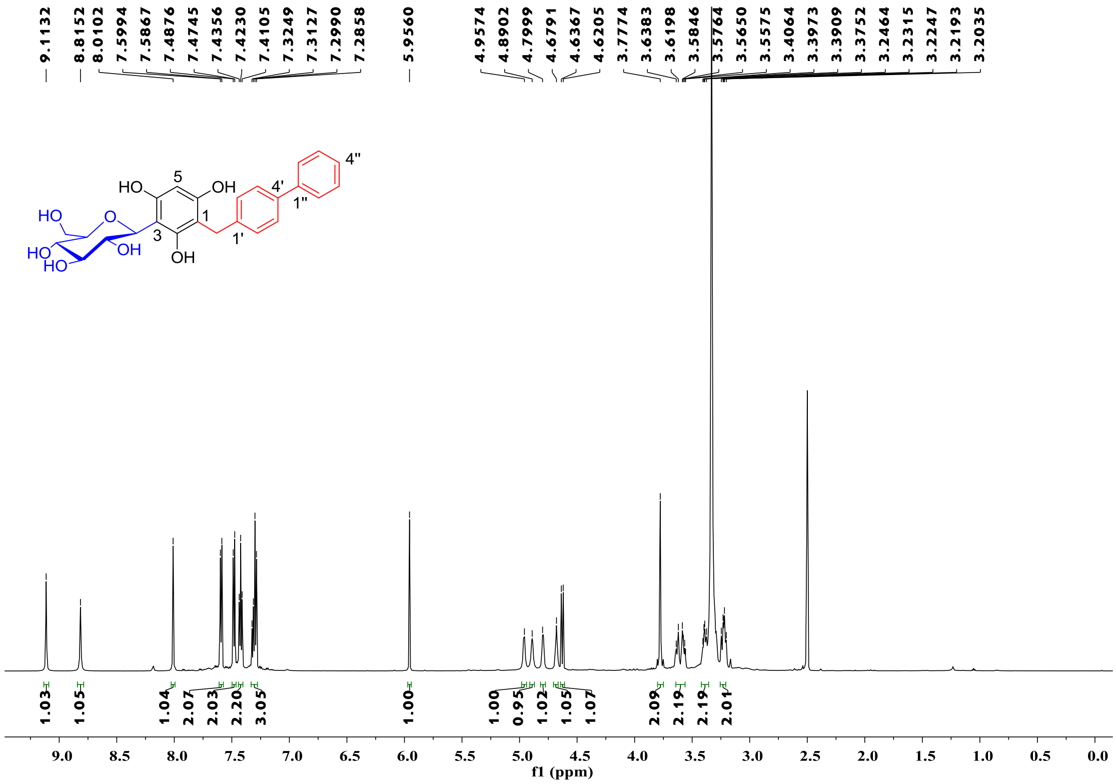

Supplementary Figure 157 ^{1}H NMR spectrum of **50a** (DMSO-d_6, 600 MHz)

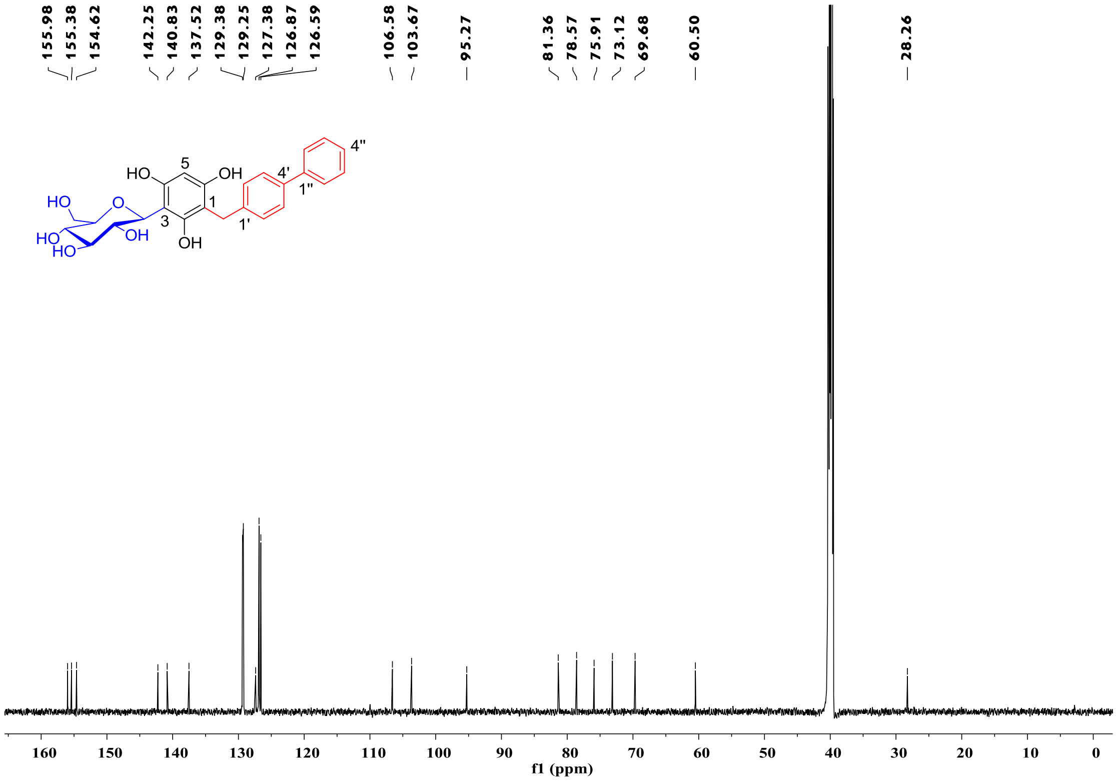

Supplementary Figure 158 ^{13}C NMR spectrum of **50a** (DMSO-d_6, 150 MHz)

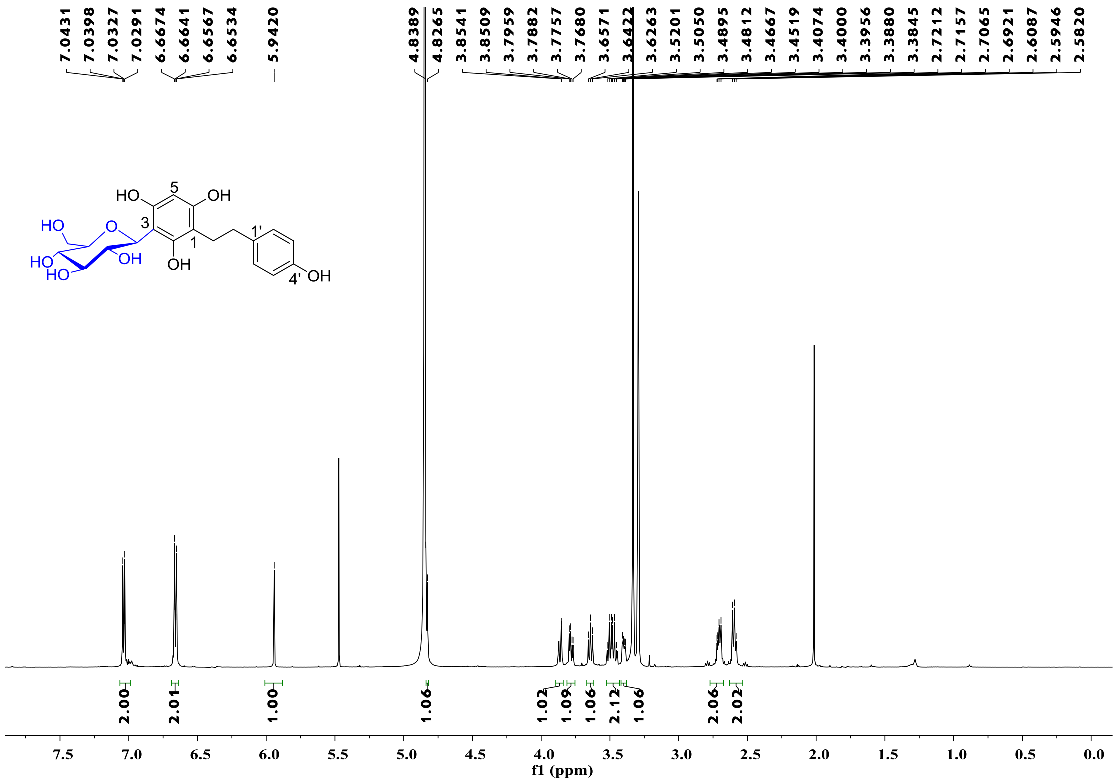

Supplementary Figure 159 ^{1}H NMR spectrum of **51a** (Methanol-d_4, 600 MHz)

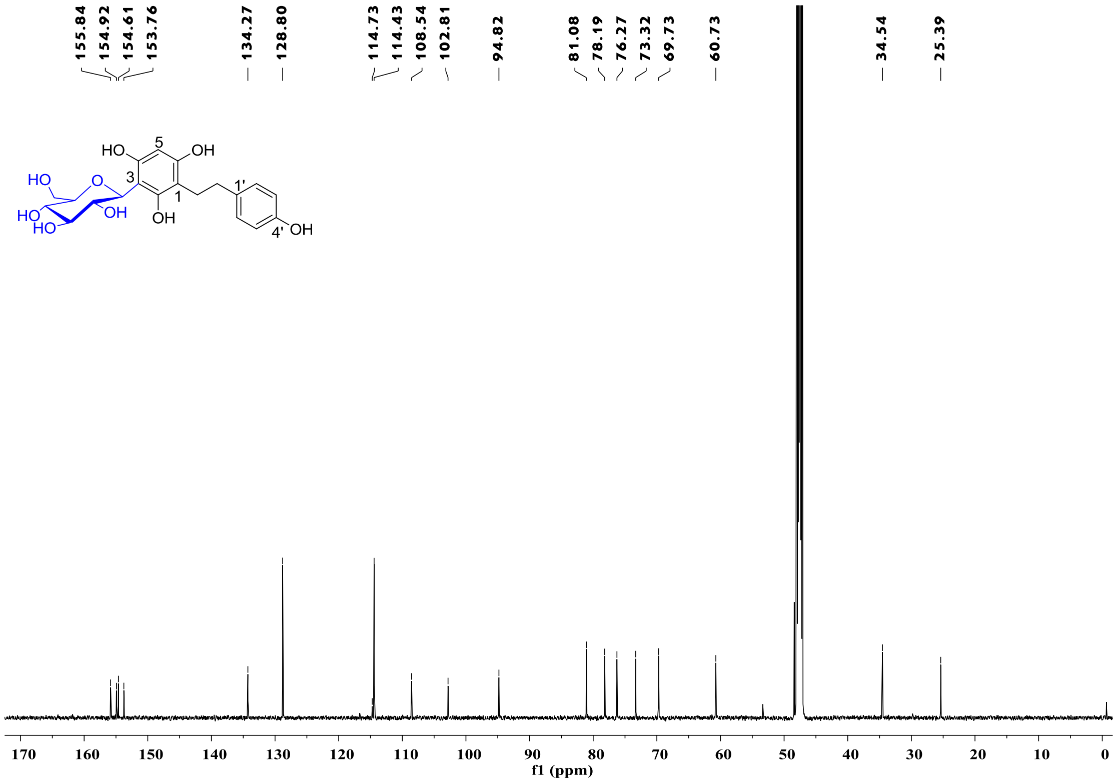

Supplementary Figure 160 ^{1}H NMR spectrum of **51a** (Methanol-d_4, 150 MHz)

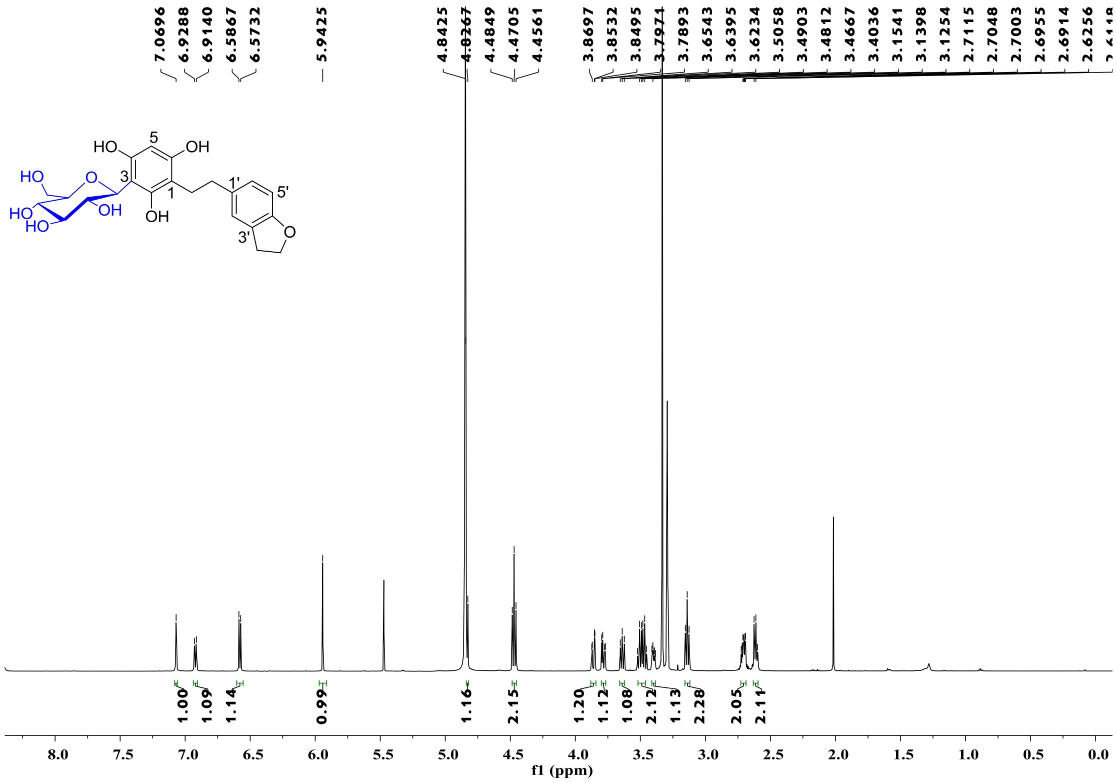

Supplementary Figure 161 ^{1}H NMR spectrum of **52a** (DMSO-d_6, 600 MHz)

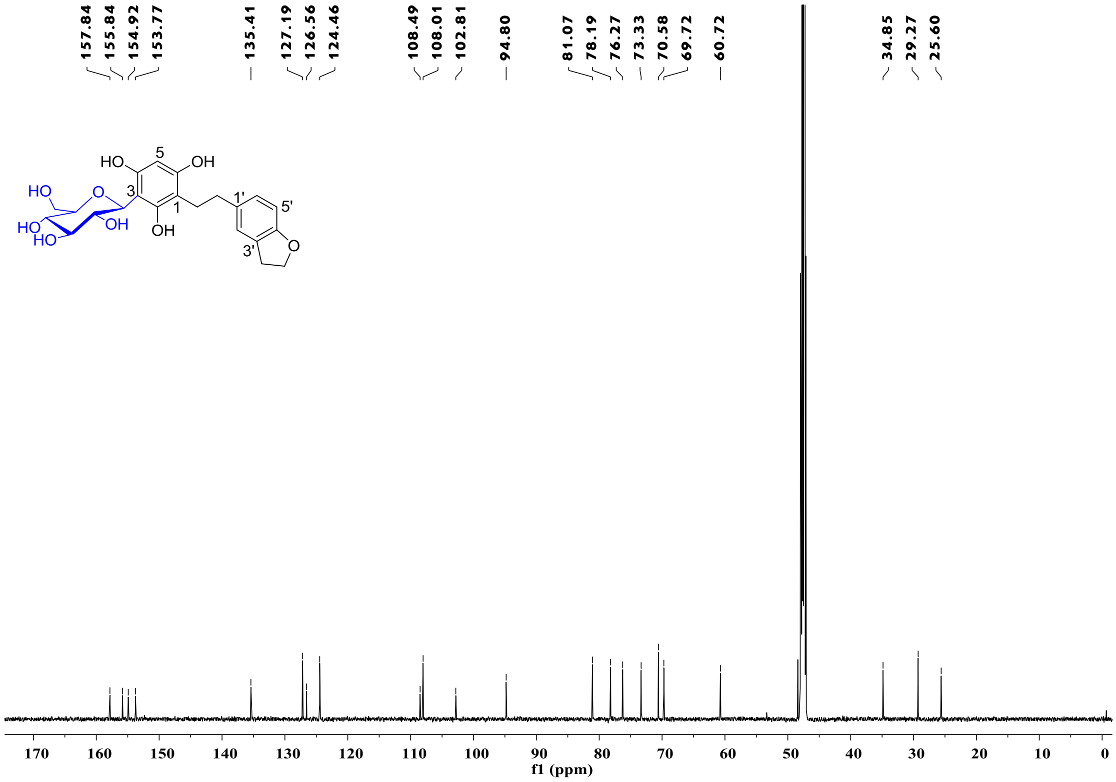

Supplementary Figure 162 ^{13}C NMR spectrum of **52a** (DMSO-d_6, 150 MHz)

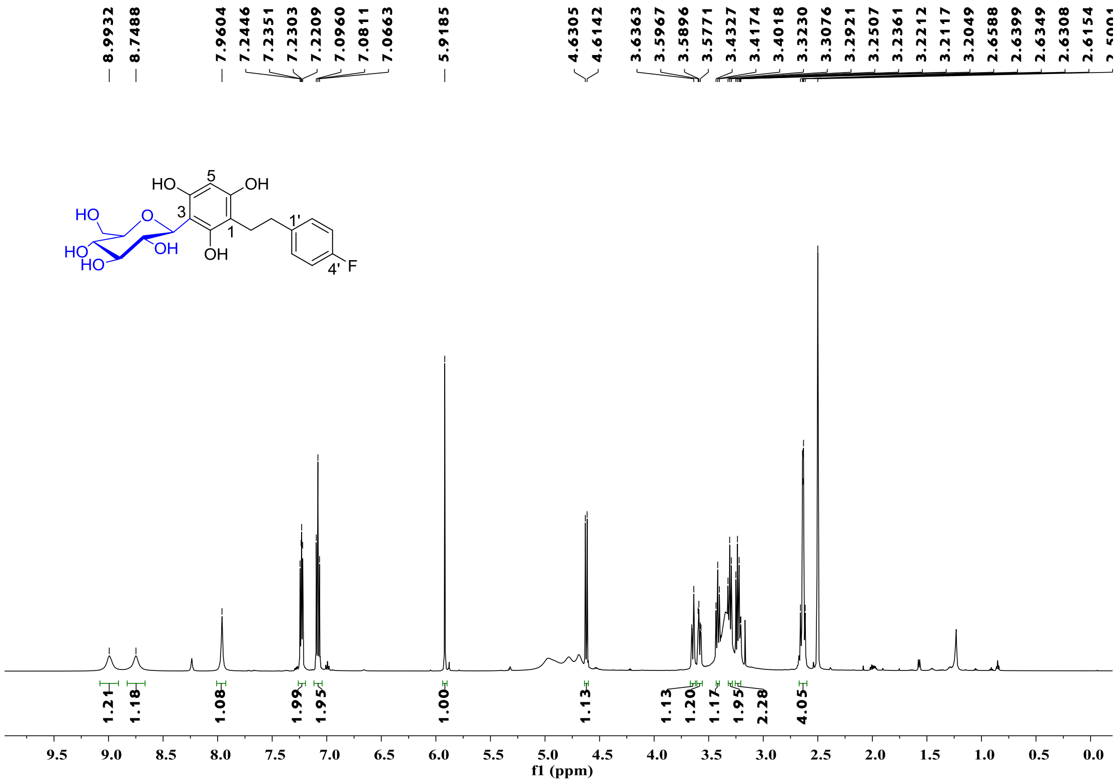

Supplementary Figure 163 ^{1}H NMR spectrum of **53a** (DMSO-d_6, 600 MHz)

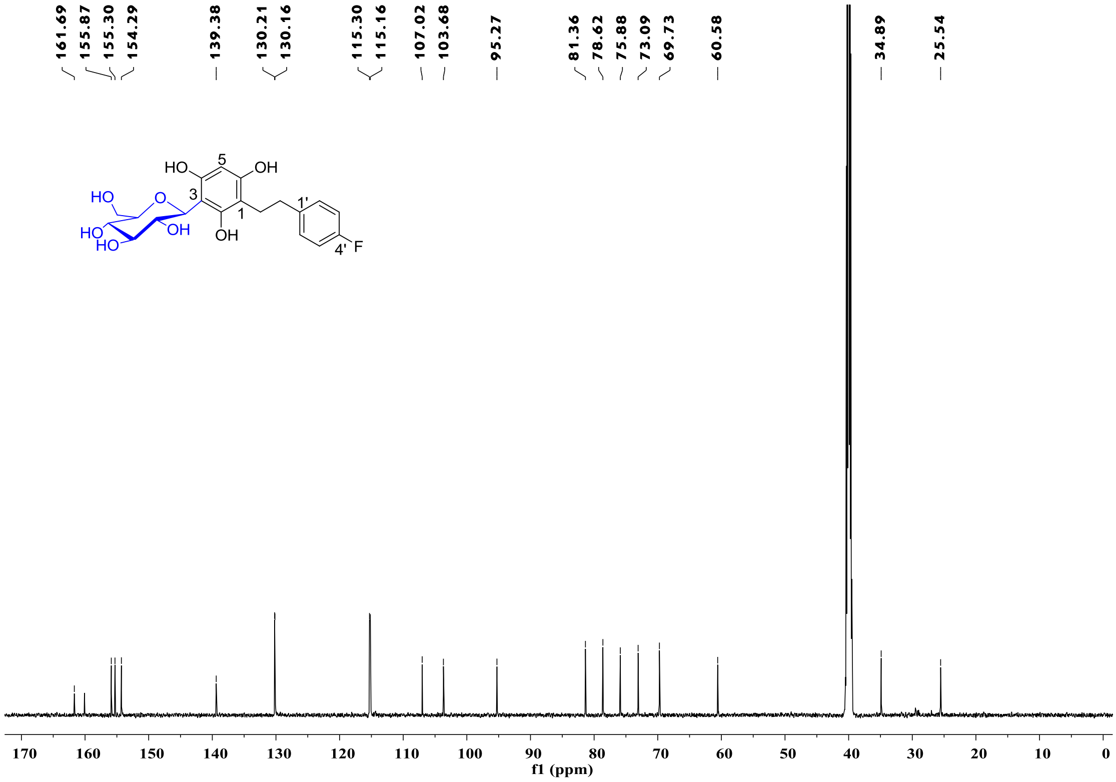

Supplementary Figure 164 ^{13}C NMR spectrum of **53a** (DMSO-d_6, 150 MHz)

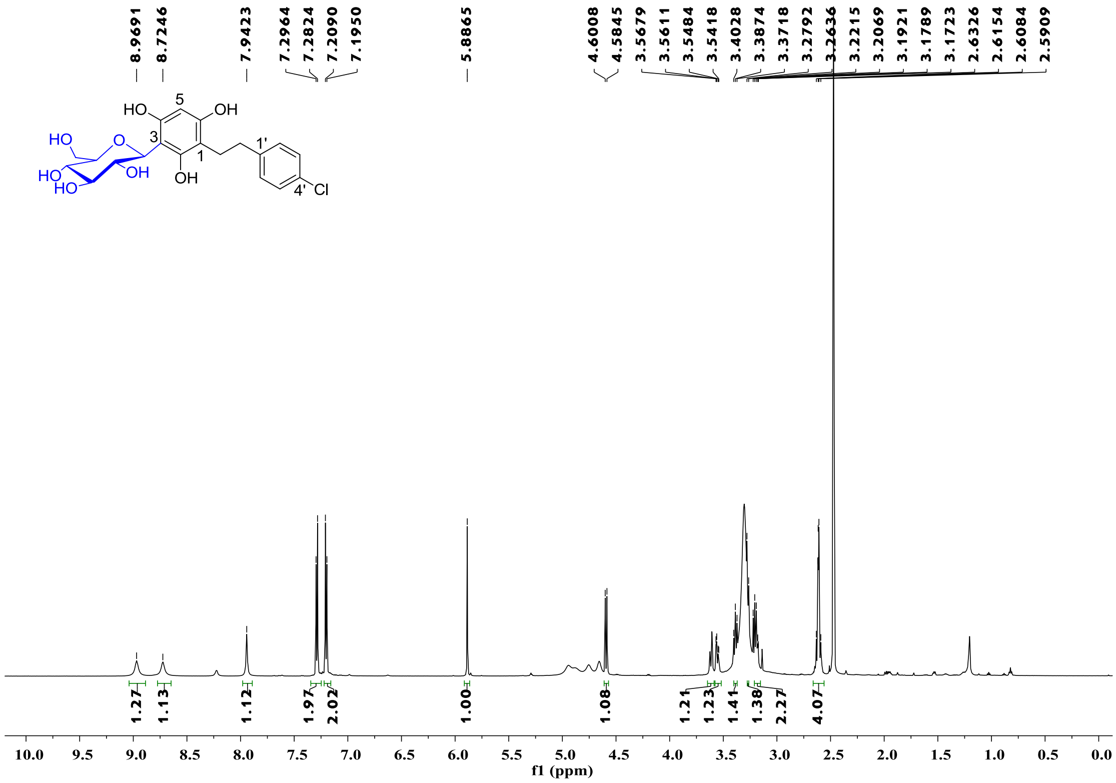

Supplementary Figure 165 ^{1}H NMR spectrum of **54a** (DMSO-d_6, 600 MHz)

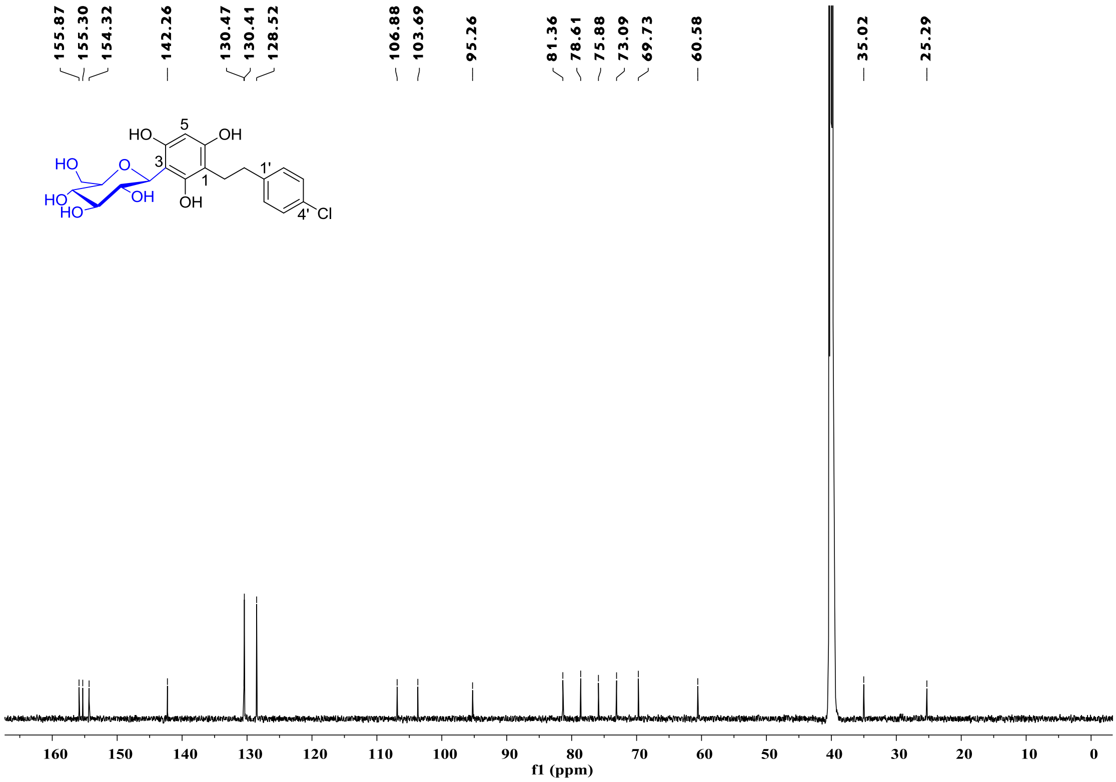

Supplementary Figure 166 ^{13}C NMR spectrum of **54a** (DMSO-d_6, 150 MHz)

Supplementary References

1. Gao Q, Wang Z, Liu Z, et al. A cell-based high-throughput approach to identify inhibitors of influenza A virus. *Acta Pharmaceutica Sinica B*, 2014, **4**, 301.

2. Wang M, Zhang G, Wang Y, et al. Design, synthesis and anti-influenza A virus activity of novel 2, 4-disubstituted quinazoline derivatives. *Bioorganic & Medicinal Chemistry Letters*, 2020, **30**, 127143.
