# Supplementary material for: Exploring and applying the substrate promiscuity of a C-glycosyltransferase in the chemo-enzymatic synthesis of bioactive C-glycosides
Source: Nat Commun. 2020 Oct 14;11:5162. doi: 10.1038/s41467-020-18990-9 (PMC7558026; doi:10.1038/s41467-020-18990-9)
Supplement: Supplementary file 3 — Reporting Summary [file 41467_2020_18990_MOESM3_ESM.pdf]

nature research

Corresponding author(s): Kebo Xie, Fei Ye, Jungui Dai

Last updated by author(s): Sep 19, 2020

Reporting Summary

Nature Research wishes to improve the reproducibility of the work that we publish. This form provides structure for consistency and transparency in reporting. For further information on Nature Research policies, see our Editorial Policies and the Editorial Policy Checklist.

Statistics

For all statistical analyses, confirm that the following items are present in the figure legend, table legend, main text, or Methods section.

n/a	Confirmed	
☐	☒	The exact sample size (n) for each experimental group/condition, given as a discrete number and unit of measurement
☒	☐	A statement on whether measurements were taken from distinct samples or whether the same sample was measured repeatedly
☐	☒	The statistical test(s) used AND whether they are one- or two-sided<br>*Only common tests should be described solely by name; describe more complex techniques in the Methods section.*
☒	☐	A description of all covariates tested
☒	☐	A description of any assumptions or corrections, such as tests of normality and adjustment for multiple comparisons
☐	☒	A full description of the statistical parameters including central tendency (e.g. means) or other basic estimates (e.g. regression coefficient) AND variation (e.g. standard deviation) or associated estimates of uncertainty (e.g. confidence intervals)
☐	☒	For null hypothesis testing, the test statistic (e.g. F, t, r) with confidence intervals, effect sizes, degrees of freedom and P value noted<br>*Give P values as exact values whenever suitable.*
☒	☐	For Bayesian analysis, information on the choice of priors and Markov chain Monte Carlo settings
☒	☐	For hierarchical and complex designs, identification of the appropriate level for tests and full reporting of outcomes
☒	☐	Estimates of effect sizes (e.g. Cohen's d, Pearson's r), indicating how they were calculated

Our web collection on statistics for biologists contains articles on many of the points above.

Software and code

Policy information about availability of computer code

Data collection	AutoDockTools-1.5.6
Data analysis	DNAMAN Version 7, MEGA5, MestReNova 11.0, Primer Premier 5, Origin 2018, ChemDraw 19.0, SWISS MODEL (http://www.swissmodel.expasy.org/), PyMOL 0.99rc6 and GraphPad Prism 5

For manuscripts utilizing custom algorithms or software that are central to the research but not yet described in published literature, software must be made available to editors and reviewers. We strongly encourage code deposition in a community repository (e.g. GitHub). See the Nature Research guidelines for submitting code & software for further information.

Data

Policy information about availability of data

All manuscripts must include a data availability statement. This statement should provide the following information, where applicable:

- Accession codes, unique identifiers, or web links for publicly available datasets
- A list of figures that have associated raw data
- A description of any restrictions on data availability

GenBank accession number of AbCGT is MN747045

Field-specific reporting

Life sciences study design

All studies must disclose on these points even when the disclosure is negative.

Sample size	No statistical method was used to predetermine sample size. To determine the enzymatic activities, conversion rates, Km, kcat of AbCGT and its variants, 3 biological replicates (n=3)were used in the experiments and this sample size is sufficient for a statistical analysis. For the bioactivity assays in vivo the sample size of n=4-8 was selected in this study. The sample size is sufficient to generate statistically significant results.
Data exclusions	No data were excluded from this study.
Replication	All experiments were repeated independently and showed similar results. Number of repeats is given in the figure legends and table notes.
Randomization	To establish Type 1 diabetes mellitus (T1DM) mice, the mice were randomized before injection of alloxan tetrahydrate. To evaluate the hypoglycemic effect of 46a in type 2 diabetes mellitus (T2DM), model mice were randomly divided into the three groups.
Blinding	The experiments are unblinded because these analyses were performed using quantifiable parameters and calculated by established software.

Reporting for specific materials, systems and methods

We require information from authors about some types of materials, experimental systems and methods used in many studies. Here, indicate whether each material, system or method listed is relevant to your study. If you are not sure if a list item applies to your research, read the appropriate section before selecting a response.

Materials & experimental systems

n/a	Involved in the study	
☒	☐	Antibodies
☒	☐	Eukaryotic cell lines
☒	☐	Palaeontology and archaeology
☐	☒	Animals and other organisms
☒	☐	Human research participants
☒	☐	Clinical data
☒	☐	Dual use research of concern

Methods

n/a	Involved in the study	
☒	☐	ChIP-seq
☒	☐	Flow cytometry
☒	☐	MRI-based neuroimaging

Animals and other organisms

Policy information about studies involving animals; ARRIVE guidelines recommended for reporting animal research

Laboratory animals	Male ICR mice were obtained from Beijing Vital River Laboratory Animal Technology Co., Ltd, China. Type 1 diabetes mellitus (T1DM) mice were established by intravenous injection of alloxan tetrahydrate. Female KKAy mice were obtained from the Animal Center of the Institute of Laboratory Animal Sciences, Chinese Academy of Medical Sciences and Peking Union Medical College. Mice were housed in grommet cages (four mice per cage) under the conditions of temperature 21–23 °C, humidity 40%–60%, 12 h light/dark cycle, and ad libitum access to water and chow diet. Male ICR mice for OGTT and T1DM are 6-7 weeks old; Female KKAy mice are 18 weeks old.
Wild animals	The study did not involve wild animals.
Field-collected samples	The study did not involve samples collected from the field.
Ethics oversight	We have complied with all relevant ethical regulations, and all experimental procedures were approved by the Institutional Animal Care and Use Committee of the Institute of Materia Medica, Chinese Academy of Medical Sciences.

Note that full information on the approval of the study protocol must also be provided in the manuscript.
